# Clinical usability of an explainable AI decision support tool and evaluation of multimodal models in NSCLC

**DOI:** 10.1038/s41591-026-04488-2

**Published:** 2026-09-13

**Authors:** Arsela Prelaj, Vanja Miskovic, Matteo Sacco, Alberto Ferrarin, Cristina Maria Licciardello, Leonardo Provenzano, Margherita Favali, Ludovica Lerma, Aleksandra Zec, Andrea Spagnoletti, Monica Ganzinelli, Daniele Lorenzini, Beshoy Guirges, Luca Invernizzi, Cecilia Silvestri, Laura Mazzeo, Marco Meazza Prina, Giulia Corrao, Margherita Ruggirello, Andra Diana Dumitrascu, Rosa Maria Di Mauro, Dario Monzani, Gabriella Pravettoni, Michele Zanitti, Davide Macocchi, Moreno Bruno Marino, Chiara Cavalli, Rebecca Romanò, Claudia Giani, Samuel G. Armato, Alessandra Esposito, Christine M. Bestvina, Maria Spector, Bogot R. Naama, Reham Basheer, Adi Lahiani Hafzadi, Laila Roisman, Iris Watermann, Marlen Szewczyk, Till Olchers, Heinz Richter, Constantin Blanke-Roeser, Costanza Siniscalchi, Anna Di Lello, Teresa Arangoa, Valentina Bartolomeo, Nikolaos Spathas, Evangelos Sarris, Elena Fountzilas, Aina Arbusà Roca, Rocio Caro-Consuegra, Patricia Iranzo, Melissa Fernández-Pinto, Jose Rodríguez-Morató, Luca Agnelli, Mario Occhipinti, Marta Brambilla, Teresa Beninato, Claudia Proto, Sokol Kosta, Michele Pio Di Palma, Eliana Rulli, Stefan Steurer, Ronald Simon, Michael Willis, Giancarlo Pruneri, Filippo De Braud, Marcello Restelli, Enriqueta Felip, Nir Peled, Alexander T. Pearson, Helena Linardou, Martin Reck, Giuseppe Lo Russo, Francesco Trovò, Alessandra Laura Giulia Pedrocchi, Marina Chiara Garassino

**Affiliations:** 1 Medical Oncology Department, Fondazione IRCCS Istituto Nazionale dei Tumori di Milano, Milan, Italy; 2 Department of Electronics, Information, and Bioengineering, Politecnico di Milano, Milan, Italy; 3 Department of Medicine, Section of Hematology Oncology, Thoracic Oncology Program, The University of Chicago, Chicago, IL USA; 4 Department of Diagnostic Innovation, Fondazione IRCCS Istituto Nazionale dei Tumori di Milano, Milan, Italy; 5 Applied Research Division for Cognitive and Psychological Science, IEO European Institute of Oncology IRCCS, Milan, Italy; 6 Department of Psychology, Educational Science and Human Movement, University of Palermo, Palermo, Italy; 7 WeSearch Lab - Laboratory of Behavioral Observation and Research on Human Development, University of Palermo, Palermo, Italy; 8 Department of Oncology and Hemato-oncology, University of Milan, Milan, Italy; 9 Department of Electronic Systems, Aalborg University, Copenhagen, Denmark; 10 ML cube, Milan, Italy; 11 Department of Radiology, The University of Chicago, Chicago, IL USA; 12 Shaare Zedek Medical Center, Jerusalem, Israel; 13 LungenClinic Grosshansdorf, Airway Research Center North, German Center for Lung Research, Grosshansdorf, Germany; 14 Department of Medical Oncology, Hospital Universitario de La Princesa, Madrid, Spain; 15 Radiation Oncology, Fondazione IRCCS Istituto Nazionale dei Tumori, Milan, Italy; 16 4th Oncology Department & Comprehensive Clinical Trials Center, Metropolitan Hospital, Athens, Greece; 17 Department of Medical Oncology, St. Luke’s Clinic, Thessaloniki, Greece; 18 Thoracic Tumors Group, Vall d’Hebron Institute of Oncology (VHIO), Vall d’Hebron Barcelona Hospital Campus, Barcelona, Spain; 19 Medica Scientia Innovation Research (MEDSIR), Barcelona, Spain; 20 Methodology for Clinical Research Laboratory, Istituto di Ricerche Farmacologiche Mario Negri IRCCS, Milan, Italy; 21 Institute for Pathology, University Medical Center Hamburg-Eppendorf, Hamburg, Germany; 22 The Swedish Institute for Health Economics, Lund, Sweden; 23 Chan Zuckerberg Biohub Chicago, LLC, Chicago, IL USA; 24 Molecular Immunology Unit, Department of Experimental Oncology, Fondazione IRCCS Istituto Nazionale dei Tumori di Milano, Milan, Italy; 25 Catholic University of Leuven, Leuven, Belgium; 26 Lung Cancer Europe (LUCE), Bern, Switzerland; 27 European Interdisciplinary Society for AI in Cancer Research (ESAC), Milan, Italy

**Keywords:** Predictive markers, Prognostic markers

## Abstract

Despite a decade in, immunotherapy (IO) treatment selection in non-small cell lung cancer (NSCLC) remains largely guided by subgroup analyses and imperfect programmed death ligand 1 (PD-L1) and clinical scores. To our knowledge, I^3^LUNG (NCT05537922) is currently the largest international, real-world, multimodal, artificial intelligence (AI)-based study, enrolling 2,396 patients. We integrated real-world clinical and blood (CB) data, computed tomography (CT) images, digital pathology (DP), and genomics into machine learning early fusion (MLEF) and deep learning intermediate fusion (DLIF) models. Machine learning (ML) and deep learning (DL) CB-only models achieved consistent performance across outcomes with area under the curve (AUC) up to 0.77 in the test (TEST) set. Performance drop in external validation (EXVAL) likely reflects population differences (AUC range: 0.55–0.72). AI models significantly surpassed PD-L1, Eastern Cooperative Oncology Group performance status (ECOG PS), neutrophil-to-lymphocyte ratio (NLR), lactate dehydrogenase (LDH) and Lung Immune Prognostic Index (LIPI) score in the independent TEST set. The clinical usability study showed that lung expert and nonexpert physicians improved their prediction with the explainable AI (XAI) ML CB-only based tool. Although multimodal integration with MLEF (CB+CT+DP) was associated with higher performance, its incremental benefit remains uncertain, not translated in TEST and EXVAL. The I^3^LUNG project is a pioneering framework showing the clinical usefulness of AI tools. A prospective validation of the decision support system (both CB and multimodal) is currently undergoing in more than 2,000 patients.

## Abstract (web summary)

In a large international real-world study of non-small cell lung cancer, a multimodal explainable AI model outperformed established biomarkers for immunotherapy outcome prediction and improved physician decision-making.

## Main

IO targeting programmed cell death protein 1 (PD-1)^1–3^, PD-L1 (ref. ^4^) and cytotoxic T-lymphocyte-associated protein 4 (ref. ^5^) has transformed metastatic NSCLC care, achieving long-term benefit in 20–30% of patients^6^. IO alone or in combination with chemotherapy (IO/CHT) currently forms the backbone of advanced non-oncogene-addicted NSCLC treatment; however, most patients face primary (5–20%) or secondary (60–85%) resistance^7^. This underscores the need for robust predictive biomarkers to identify likely IO responders at diagnosis, avoiding unnecessary toxicity and unnecessary cost. Despite its limited predictive power, PD-L1 remains the only clinically approved biomarker^8^.

Beyond traditional methods, AI-based approaches leverage large-scale, complex data to enable individualized IO outcome prediction. Although unimodal studies (for example, genomics^9^, radiomics^10^ and DP^11^) show promise, they remain limited by size, scope and single-center design^12^. Two recent studies demonstrated the potential of multimodal integration for predicting IO response in NSCLC. Vanguri et al.^13^ combined CT, histopathology and genomics, and Captier et al.^14^ integrated positron emission tomography (PET), pathology, transcriptomics and clinical data. Although both models outperformed traditional biomarkers, they were limited by small cohorts (approximately 250–300 patients), with only approximately 80 patients having complete multimodal data.

**Extended Data Fig. 1:**
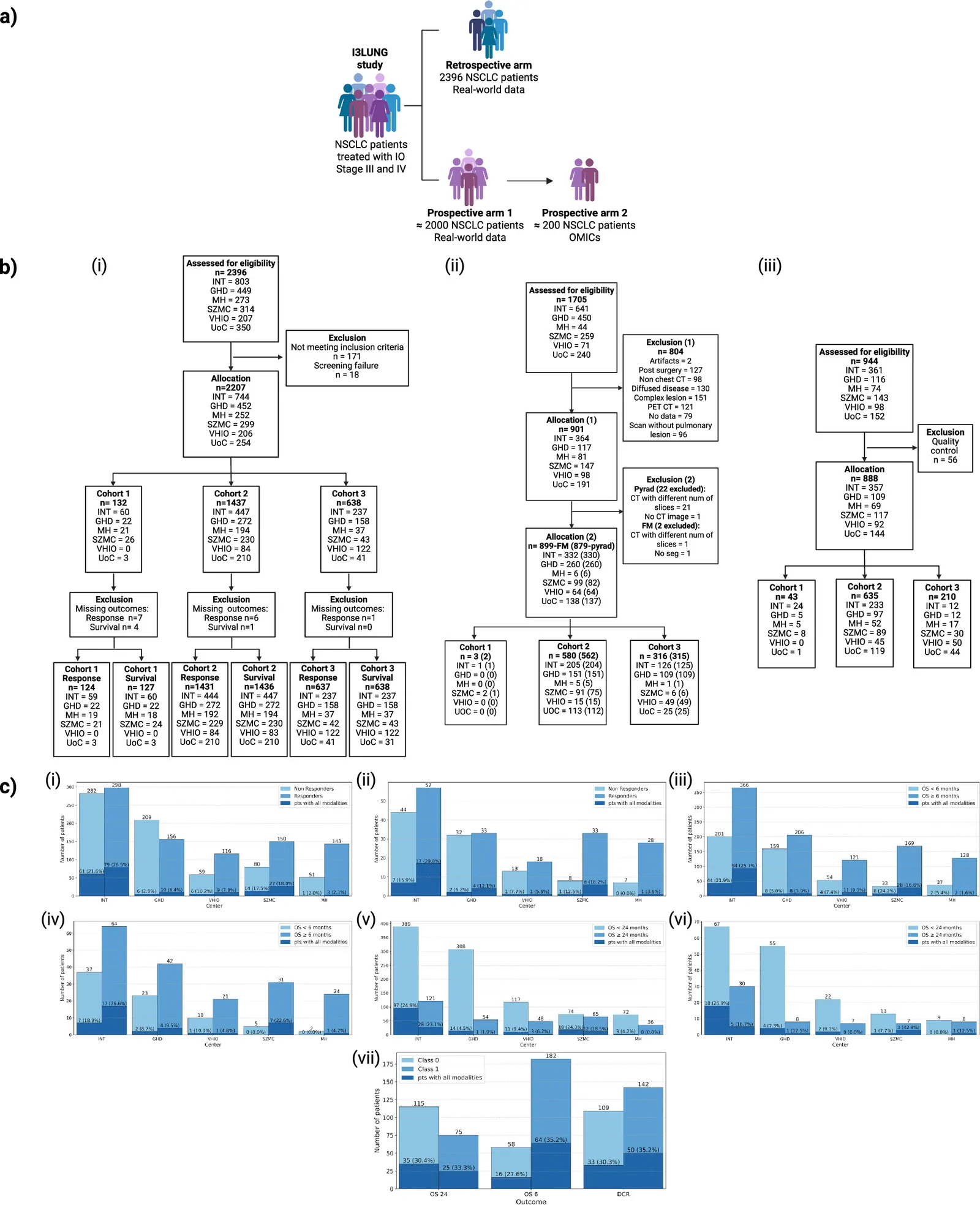
Cohort overview and data analysis. **a**) I3LUNG cohort design; **b**) Consort flow diagram for (i) clinical data, (ii) CT images, and (iii) DP; **c**) Distribution of pts across centres by outcomes: DCR in TRAIN (i) and TEST (ii), OS6 in TRAIN (iii) and TEST (iv), OS24 in TRAIN (v) and TEST (vi), and EXVAL (vii). *Created in BioRender. PRELAJ*, *A*. (2026) https://BioRender.com/ulcxt65.

To support individualized IO decisions, the I^3^LUNG (NCT05537922) project^15^ was launched under the Horizon Europe 2021–2027 framework, as an international, multicenter study. It includes retrospective/prospective data to develop and validate AI-based physician decision support systems (PDSSs) for optimizing IO treatment selection in advanced NSCLC. Although prospective patient enrollment is ongoing, the present study focuses on the retrospective arm of the I^3^LUNG project (Extended Data Fig. 1a). Here we describe the multimodal data collection pipeline and present, to our knowledge, the first set of AI-based PDSSs developed using data from patients with advanced NSCLC treated with IO or IO/CHT between September 2012 and October 2023. These patients were enrolled across six clinical centers, including four in the European Union (Istituto Nazionale dei Tumori (INT-Italy), Metropolitan Hospital (MH-Greece), LungenClinic Grosshansdorf (GHD-Germany) and Vall d’Hebron Institute of Oncology (VHIO-Spain)) and two outside the European Union (University of Chicago (UOC) (UOC-USA) and Shaare Zedek Medical Center (SZMC-Israel)). We introduce a CB and multimodal framework for personalized treatment selection comprising foundation-model-based extraction from medical images, comparative evaluation of early fusion and intermediate fusion models, explainability and fairness analyses and a clinical usability study assessing real-world utility.

## Results

### Overview of the study design

**Fig. 1:**
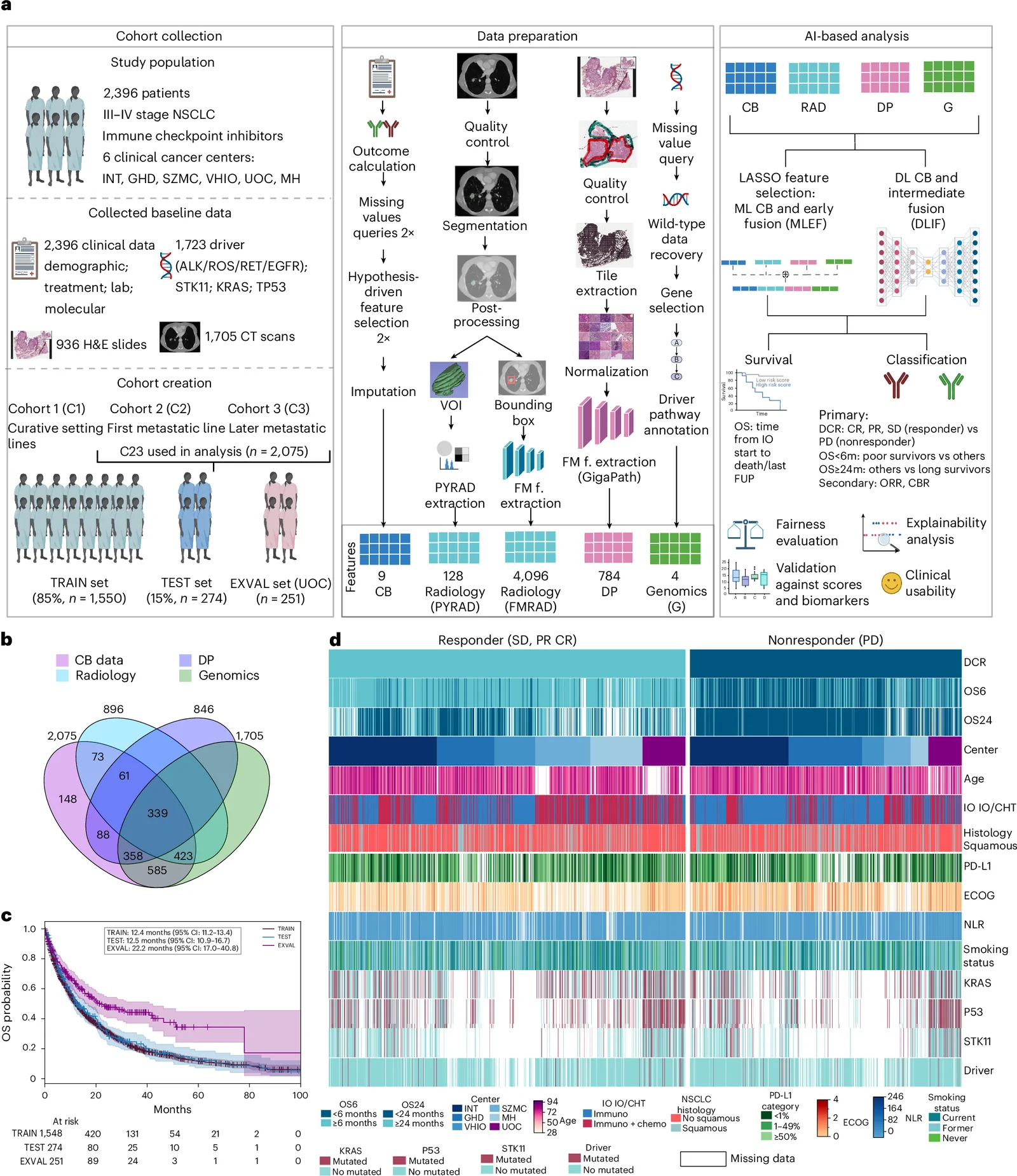
Overview of the study and dataset description. **a**, Study overview. Cohort collection: four data modalities from patients with NSCLC included in the overall study cohort (left); data preparation: showing different workflows for data preprocessing and feature extraction for each modality, including CB, radiology (two pipelines), DP and genomics (middle); and AI-based analysis: overview of two different approaches—ML: CB-only and multimodal MLEF and DL: CB-only and multimodal DLIF—and performed analyses (right). **b**, Cohort C23 modality overview (*n* = 2,075 patients); 339 patients had all 4 modalities available. **c**, Kaplan–Meier survival curves for TRAIN, TEST and EXVAL (UOC), using OS as the endpoint. **d**, Multimodal cohort heatmap listing characteristics for *n* = 2,075 patients. White indicates missing information. VOI, volume of interest; FM f., foundation model features; FUP, follow-up; immuno, immunotherapy; chemo, chemotherapy. Figure created in BioRender; Prelaj, A. https://biorender.com/ulcxt65 (2026).

The overview of this study is shown in Fig. 1a. As of 15 January 2025, 2,396 patients with stage IIIC–IVB advanced NSCLC treated with IO or IO/CHT were enrolled from the six clinical centers. The dataset included CB data (sex, ECOG PS, smoking status, PD-L1 expression, metastatic sites (bone, liver, brain), NLR and LDH) (CB, *n* = 2,396); most commonly performed genomic analyses (*KRAS*, *TP53*, *STK11*, *ALK*, *EGFR*, *RET* and *ROS1*; *n* = 1,723) (Supplementary Information 1, Tables 1 and 2); CT scans (*n* = 1,705) analyzed using PyRadiomics (PYRAD) and foundation models (FMRAD); and histopathology slides (DP, *n* = 936) analyzed using foundation models. The CONSORT flow for all data modalities is described in Extended Data Fig. 1b; additional data analysis is shown in Supplementary Information 1 (Tables 3–36). Patients were allocated into three cohorts: C1, patients with stage III NSCLC who received IO as maintenance treatment after chemoradiation therapy; C2, patients with stage IV NSCLC who received IO as first-line treatment for metastatic disease; and C3, patients with stage IV NSCLC who received IO as second-line or further-line therapy. Additional non-IO cohorts have been included to test the predictive role of the ML CB-only models—in particular, EGFR-mutant cohorts treated with EGFR-tyrosine kinase inhibitors (TKIs) (C-EGFR-INT, *n* = 132; C-EGFR-UOC, *n* = 139) and a stage III cohort treated with CHT and surgery at INT center (C-StageIII-CHT, *n* = 91). Additionally, we collected data from C3 that received first-line CHT at INT (C-LineI-CHT, *n* = 208). In this analysis, we used CB features collected at baseline CHT, whereas the original I^3^LUNG combined C2 and C3 (C23) models were developed using baseline features at IO start (details in Methods). Statistical analysis related to the difference between IO and non-IO cohorts are reported in Supplementary Information 1 (Tables 29–36). Clinical endpoints included real-world overall survival (OS), survival status at 6 months (OS6) and 24 months (OS24) and disease control rate (DCR). Secondary endpoints included clinical benefit rate (CBR) and overall response rate (ORR) (definitions in Methods).

The main predictive AI model was built using the combined cohort C23 (C2, *n* = 1,437, C3, *n* = 638; *n* = 2,075). Patients from UOC served as an EXVAL (*n* = 251) cohort to assess generalizability. The remaining 1,824 patients were split into a training set (TRAIN; 85%, *n* = 1,550) with five-fold cross-validation (CV, using centers as folds) and a TEST set (15%, *n* = 274). Subgroup analyses were conducted by training and evaluating additional submodels using clinically relevant subgroups as input: C2 only; PD-L1 ≥ 50%, 1–49% and <1%; patients who received IO as monotherapy; patients who received IO/CHT; patients with squamous cell carcinoma and adenocarcinoma histology; and single centers.

We used two AI approaches for CB model development and multimodal integration: MLEF and DLIF models. Survival models, including Cox, were built for OS outcome predictions, and classification models were used for OS6, OS24 and DCR predictions. Finally, model post hoc explainability (XAI) analysis, fairness evaluation and a clinical usability study were performed on the TEST to assess the clinical relevance and trustworthiness of the models.

### Clinical characteristics of patients included in the analysis and multimodal data description

For C23, CB and both image modalities were available for 400 patients, whereas 339 patients had data from all four modalities (Fig. 1b). Table 1 shows patient characteristics. Median OS was 12.4 months (95% CI: 11.2–13.4) for TRAIN, 12.5 months (95% CI: 10.9–16.7) for TEST and 22.2 months (95% CI: 17.0–40.8) for EXVAL. At data cutoff, 9.3% of patients remained on treatment, and 29.8% of patients were alive (Fig. 1c). Figure 1d presents the heatmap of the main baseline characteristics, single biomarkers and outcomes. Detailed information related to descriptive analysis across centers, including genomics information, and differences among patients with available unimodal, bimodal and multimodal can be found in Supplementary Information 1 (Tables 3–28).

**Table 1:** Patient characteristics of datasets used in the study: TRAIN, TEST and EXVAL

	TRAIN set (*n* = 1,550)	TEST set (*n* = 274)	EXVAL set (*n* = 251)	Clinical usability set^a^ (*n* = 100)
Age, median (range)	68 (28–94)	67 (30–87)	69 (36–92)	67 (30–87)
Sex, *n* (%)				
Male	1,013 (65.4)	175 (63.9)	103 (41.0)	60 (60.0)
Female	537 (34.6)	99 (36.1)	148 (59.0)	40 (40.0)
NA	0 (0.0)	0 (0.0)	0 (0.0)	0 (0.0)
ECOG PS, *n* (%)				
ECOG 0	470 (30.3)	90 (32.8)	6 (2.4)	27 (27.0)
ECOG 1	725 (46.8)	115 (42)	197 (78.5)	48 (48.0)
ECOG ≥2	194 (12.5)	43 (15.7)	45 (17.9)	16 (16.0)
NA	161 (10.4)	26 (9.5)	3 (1.2)	9 (9.0)
Histology, *n* (%)				
Adenocarcinoma	1,005 (64.8)	178 (65.0)	207 (82.5)	74 (74.0)
Squamous	367 (23.7)	70 (25.6)	27 (10.8)	17 (17.0)
Other	162 (10.5)	23 (8.4)	13 (5.2)	9 (9.0)
NA	16 (1.0)	3 (1.0)	4 (1.5)	0 (0.0)
Smoking status, *n* (%)				
Current	537 (34.6)	101 (36.9)	39 (15.5)	27 (27.0)
Former	754 (48.6)	133 (48.5)	189 (75.3)	58 (58.0)
Never	117 (7.6)	19 (6.9)	23 (9.2)	6 (6.0)
NA	142 (9.2)	21 (7.7)	0 (0.0)	9 (9.0)
PD-L1 expression, *n* (%)				
<1%	428 (27.6)	75 (27.4)	101 (40.2)	30 (30.0)
1–49%	383 (24.7)	74 (27)	50 (19.9)	28 (28.0)
≥50%	424 (27.4)	66 (24.1)	69 (27.5)	30 (30.0)
NA	315 (20.3)	59 (21.5)	31 (12.4)	12 (12.0)
Therapy type, *n* (%)				
IO	904 (58.3)	161 (58.8)	96 (38.2)	50 (50.0)
IO/CHT	646 (41.7)	113 (41.2)	155 (61.8)	50 (50.0)
NA	0 (0.0)	0 (0.0)	0 (0.0)	0 (0.0)
Therapy line, *n* (%)				
1 (C2)	1,046 (67.5)	181 (66.1)	210 (83.7)	96 (96.0)
2 (C3)	344 (22.2)	66 (24.1)	36 (14.3)	4 (4.0)
≥3 (C3)	160 (10.3)	27 (9.8)	5 (2.0)	0 (0.0)
NA	0 (0.0)	0 (0.0)	0 (0.0)	0 (0.0)
Driver (EGFR/ALK/RET/ROS1), *n* (%)				
Altered	31 (2.0)	7 (2.6)	16 (6.4)	1 (1.0)
Not altered	1,193 (77.0)	208 (75.9)	220 (87.6)	79 (79.0)
NA	326 (21.0)	59 (21.5)	15 (6.0)	20 (20.0)
EGFR, *n* (%)				
Altered	24 (1.5)	3 (1.1)	9 (3.6)	0 (0.0)
Not altered	1,179 (76.1)	206 (75.2)	220 (87.6)	79 (79.0)
NA	347 (22.4)	65 (23.7)	22 (8.8)	21 (21.0)
KRAS, *n* (%)				
Altered	268 (17.3)	53 (19.3)	89 (35.5)	26 (26.0)
Not altered	379 (24.5)	60 (21.9)	131 (52.2)	26 (26.0)
NA	903 (58.2)	161 (58.8)	31 (12.3)	48 (48.0)
STK11, *n* (%)				
Altered	61 (3.9)	8 (2.9)	50 (19.9)	7 (7.0)
Not altered	321 (20.7)	57 (20.8)	170 (67.7)	30 (30.0)
NA	1,168 (75.4)	209 (76.3)	31 (12.4)	63 (63.0)
KEAP1, *n* (%)				
Altered	17 (1.1)	0 (0.0)	4 (1.6)	1 (1.0)
Not altered	446 (28.8)	78 (28.5)	33 (13.1)	21 (21.0)
NA	1,087 (70.1)	196 (71.5)	214 (85.3)	78 (78.0)
Best response, *n* (%)				
PD	681 (43.9)	104 (38.0)	109 (43.4)	35 (35.0)
SD	391 (25.2)	74 (27.0)	67 (26.7)	31 (31)
PR	429 (27.7)	84 (30.6)	69 (27.5)	29 (29.0)
CR	43 (2.8)	11 (4.0)	6 (2.4)	5 (5.0)
NA	6 (0.4)	1 (0.4)	0 (0.0)	0 (0.0)
DCR, *n* (%)				
Yes	863 (55.7)	169 (61.9)	142 (56.6)	65 (65.0)
No	681 (43.9)	104 (38.1)	109 (43.4)	35 (35.0)
NA	6 (0.4)	1 (0.4)	0 (0.0)	0 (0.0)
ORR, *n* (%)				
Yes	472 (30.5)	95 (34.6)	75 (29.9)	66 (66.0)
No	1,072 (69.1)	178 (65.0)	176 (70.1)	34 (34.0)
NA	6 (0.4)	1 (0.4)	0 (0.0)	0 (0.0)
CBR, *n* (%)				
Yes	700 (45.2)	138 (50.3)	127 (50.6)	56 (56.0)
No	842 (54.3)	135 (49.3)	124 (49.4)	44 (44.0)
NA	8 (0.5)	1 (0.4)	0 (0.0)	0 (0.0)
Survival months, median (95% CI)				
OS	12.4 (11.2–13.4)	12.5 (10.9–16.7)	22.2 (17.0–40.8)	13.6 (11.1–20.9)
PFS	5.0 (4.5–5.4)	5.1 (4.3–5.8)	8.0 (6.0–11.1)	6.1 (4.2–9.8)
Median survival follow-up (95% CI) in months				
OS	38.4 (0.0–109.0)	33.2 (0.2–114.8)	28.9 (0.1–102.4)	39.7 (0.2–63.6)

^a^Clinical usability set is composed of subsets of TEST (*n* = 80) and EXVAL (*n* = 20).

To develop predictive tools, we trained and evaluated MLEF and DLIF using multiple modality combinations and different clinically uniform patient subgroups (full list of analyses in Supplementary Information 2, Table 1).

### ML-based CB-only models provide robust IO outcome prediction

**Fig. 2:**
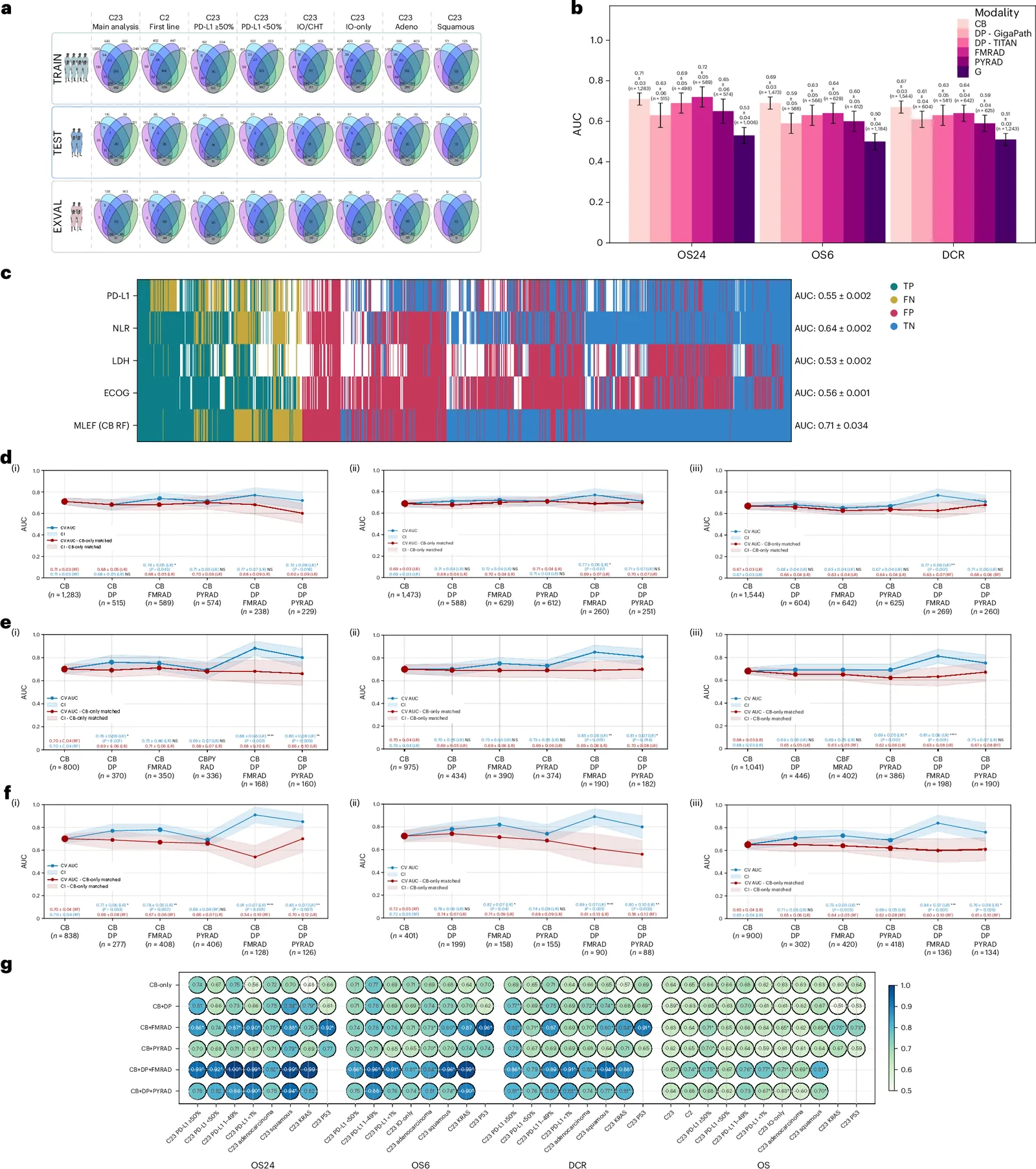
MLEF framework. **a**, MLEF subanalysis with the number of patients in TRAIN, TEST and EXVAL. **b**, Unimodal MLEF AUC performance across outcomes in CV. **c**, Patient-level comparison of MLEF and single biomarkers (C23) in CV for predicting long responders (OS24). Columns represent patients ordered from left (correctly predicted positives) to right (correctly predicted negatives), with the middle section highlighting patients where models and biomarkers consistently struggled. **d**, Classification performance of MLEF unimodal, bimodal and multimodal models for three endpoints: (i) OS24; (ii) OS6; (iii) DCR, evaluated on the C23 cohort, CV. **e**, Classification performance of MLEF unimodal, bimodal and multimodal models for three endpoints: (i) OS24; (ii) OS6; (iii) DCR, evaluated on the C2 cohort, CV. **f**, Selected MLEF subanalyses for classification: (i) OS24 in the IO-only cohort; (ii) OS6 in the PD-L1 ≥ 50% cohort; (iii) DCR in the IO-only cohort. **g**, CV AUC performance of MLEF classifier; OS24, OS6, DCR and Cox-MLEF, C-index across remaining subgroup analysis; circle color indicates the model’s performance; star (*) indicates whether there is a statistically significant difference between multimodal and CB-only models on the same population. For **b** and **d**–**f**, AUC values are shown as point estimates with 95% CIs calculated using DeLong’s method; shaded areas and error bars represent 95% CIs. For **d**–**f**, circle size indicates the number of patients in the TRAIN set. Statistical comparisons were performed between multimodal model performance (blue) and CB-only model on the same dataset (CB-only matched, red), using DeLong’s test with two-sided *z*-test *P* values. NS, not significant; *P ≤ 0.05; **P ≤ 0.01; ***P ≤ 0.001; ****P ≤ 0.0001. Adeno, adenocarcinoma; FN, false negative; FP, false positive; TN, true negative; TP, true positive; G, genomics; LR, logistic regression; RF, random forest. Figure created in BioRender; Prelaj, A. https://biorender.com/ulcxt65 (2026).

Because the MLEF workflow requires complete data, patients with missing modalities were excluded, resulting in varying sample sizes for each analysis (Fig. 2a). To ensure a fair comparison, we compared them with the corresponding CB-only models on the same subset of patients (CB-only matched). Figure 2b shows the unimodal contribution of each modality, with FMRAD providing the greatest contribution. In general, the main analysis for C23 CB-only models provided robust baseline performance across endpoints in CV (Fig. 2b), achieving AUC of 0.74 (*n* = 226; 95% CI: 0.67–0.81) in TEST and 0.60 (*n* = 190; 95% CI: 0.52–0.68) in EXVAL for OS24; AUC of 0.69 (*n* = 259; 95% CI: 0.62–0.76) in TEST and 0.64 (*n* = 240; 95% CI: 0.56–0.72) in EXVAL for OS6; and AUC of 0.71 (*n* = 273; 95% CI: 0.65–0.77) in TEST and 0.55 (*n* = 251; 95% CI: 0.48–0.62) in EXVAL for DCR. Patient case-level comparisons for OS24 in CV (Fig. 2c) demonstrated that the ML CB-only model provided a rate of correct predictions more balanced across the two classes as compared to single biomarkers.

### Multimodal integration showed exploratory gains with MLEF

Multimodal MLEF models were compared on the CV, due to the small TEST size. Specific combinations of modalities were associated with higher performance in CV across classification endpoints (Fig. 2d(i)–(iii)), particularly when CB was combined with both pathology and radiology images. In the C23 cohort, for OS24 prediction, CB+DP+PYRAD showed improved CV performance compared to CB-only matched models (AUC 0.72 (95% CI: 0.64–0.80) versus AUC 0.60 (95% CI: 0.51–0.69), *P* = 0.0158; Fig. 2d(i)). Similarly, with FMRAD features instead of PYRAD, multimodal models achieved higher performance than CB-only (DCR AUC 0.77 (95% CI: 0.71–0.83) versus 0.63 (95% CI: 0.56–0.70), *P* = 0.0014; Fig. 2d(iii)). Multimodal combinations were also associated with higher CV AUC values compared to matched CB-only models in first-line IO (Fig. 2e(i)–(iii))—for example, OS24: 0.88 (95% CI: 0.82–0.94) versus 0.68 (95% CI: 0.58–0.78), *P* ≤ 0.0001, Fig. 2e(i); DCR: 0.81 (95% CI: 0.75–0.87) versus 0.63 (95% CI: 0.55–0.71), *P* ≤ 0.0001, Fig. 2e(iii). Full analyses for unimodal and multimodal for ORR and CBR are reported in Supplementary Information 2, Figs. 1 and 2 (AUC results) and Supplementary Information 3 (all metrics results). Detailed sensitivity analyses related to multimodal models are in Supplementary Information 2, Tables 2 and 3. Further analysis using a new foundation model to extract DP features yielded similar results in multimodal settings (Supplementary Information 2, Figs. 3–5). Overall, multimodal model improvements were not consistently reproduced in the independent TEST cohort and were variably replicated in EXVAL (Supplementary Information 3).

### Survival analysis achieved consistent prediction across cohorts

**Extended Data Fig. 2:**
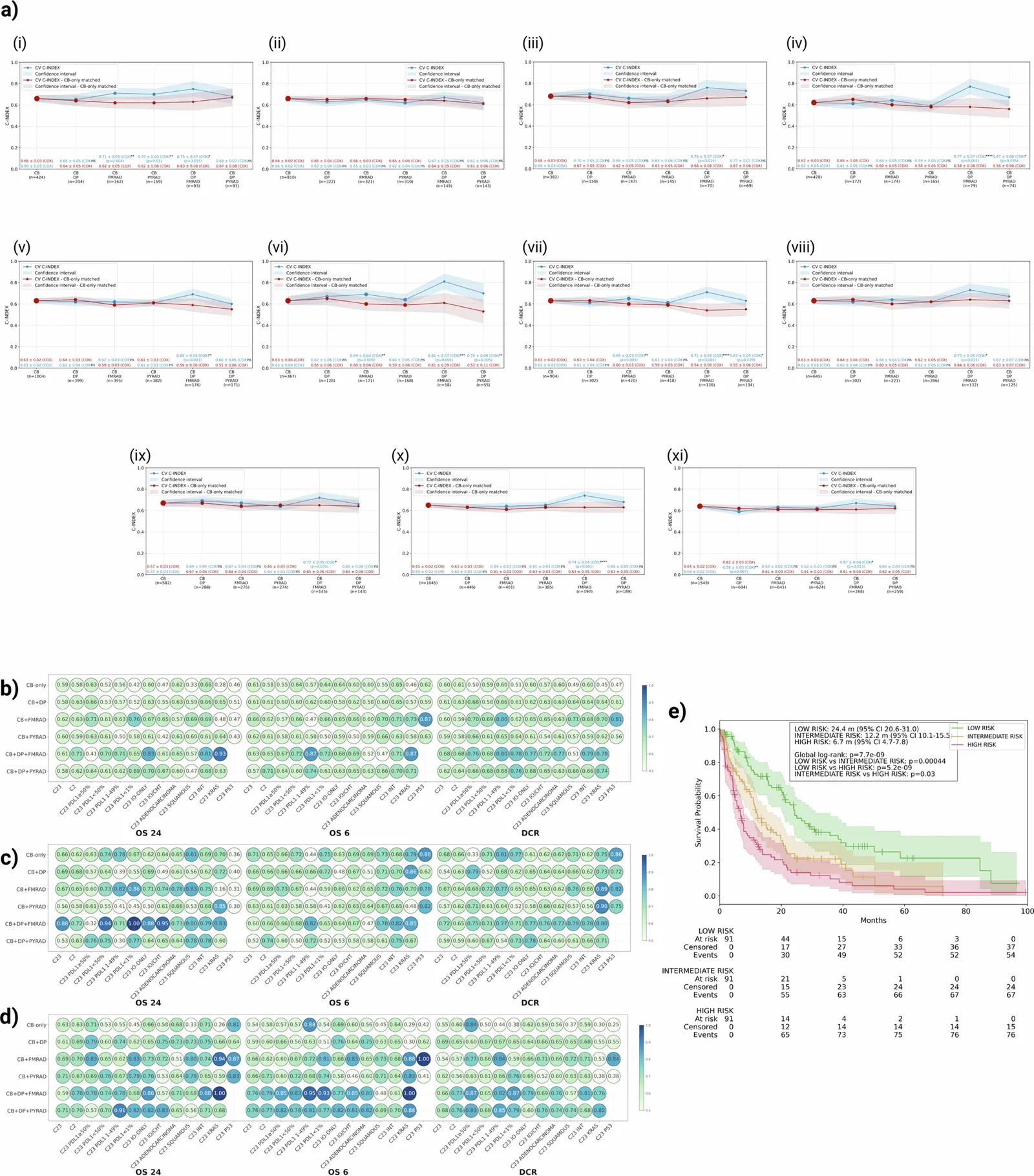
MLEF Framework. **a**) CV c-index for OS survival analysis results (i) C23 PDL1≥50%, (ii) C23 PDL1<50%, (iii) C23 PDL1 1-49%, (iv) C23 PDL1 <1%, (v) C23 adeno, (vi) C23 squamous, (vii) C23 IO-only, (viii) C23 IO/CHT, (ix) C23 INT, (x) C2, (xi) C23. C-index values are shown with 95% confidence intervals, represented by the shaded areas and calculated by bootstrapping. C-index differences were tested using bootstrapping (n=1000) with two-sided p-values; **b**) CV F1 performance of MLEF classifier (OS24, OS6, DCR) across all subgroup analysis; **c**) CV Sensitivity performance of MLEF classifier (OS24, OS6, DCR), circle color indicates the model’s performance; **d**) CV Specificity performance of MLEF classifier (OS24, OS6, DCR) across all subgroup analysis, circle color indicates the model’s performance; **e**) Cox-MLEF stratification of full TEST set. *Created in BioRender. PRELAJ*, *A*. (*2026*) https://BioRender.com/ulcxt65.

For the combined C23 analysis, the CB-only model achieved a C-index of 0.64 (95% CI: 0.62–0.66) in CV, 0.66 (95% CI: 0.62–0.70) in TEST and 0.65 (95% CI: 0.60–0.70) in EXVAL. Similar results were achieved in the C2 analysis. In both cohorts, the multimodal model with CB+DP+FMRAD outperformed the CB-only matched model in CV (C2: 0.74 (95% CI: 0.70–0.78) versus 0.63 (95% CI: 0.58–0.68), *P* = 0.0004). Full C-index results are reported in Extended Data Fig. 2a.

### Multimodal approaches gain performance in NSCLC-specific subgroups

**Extended Data Fig. 3:**
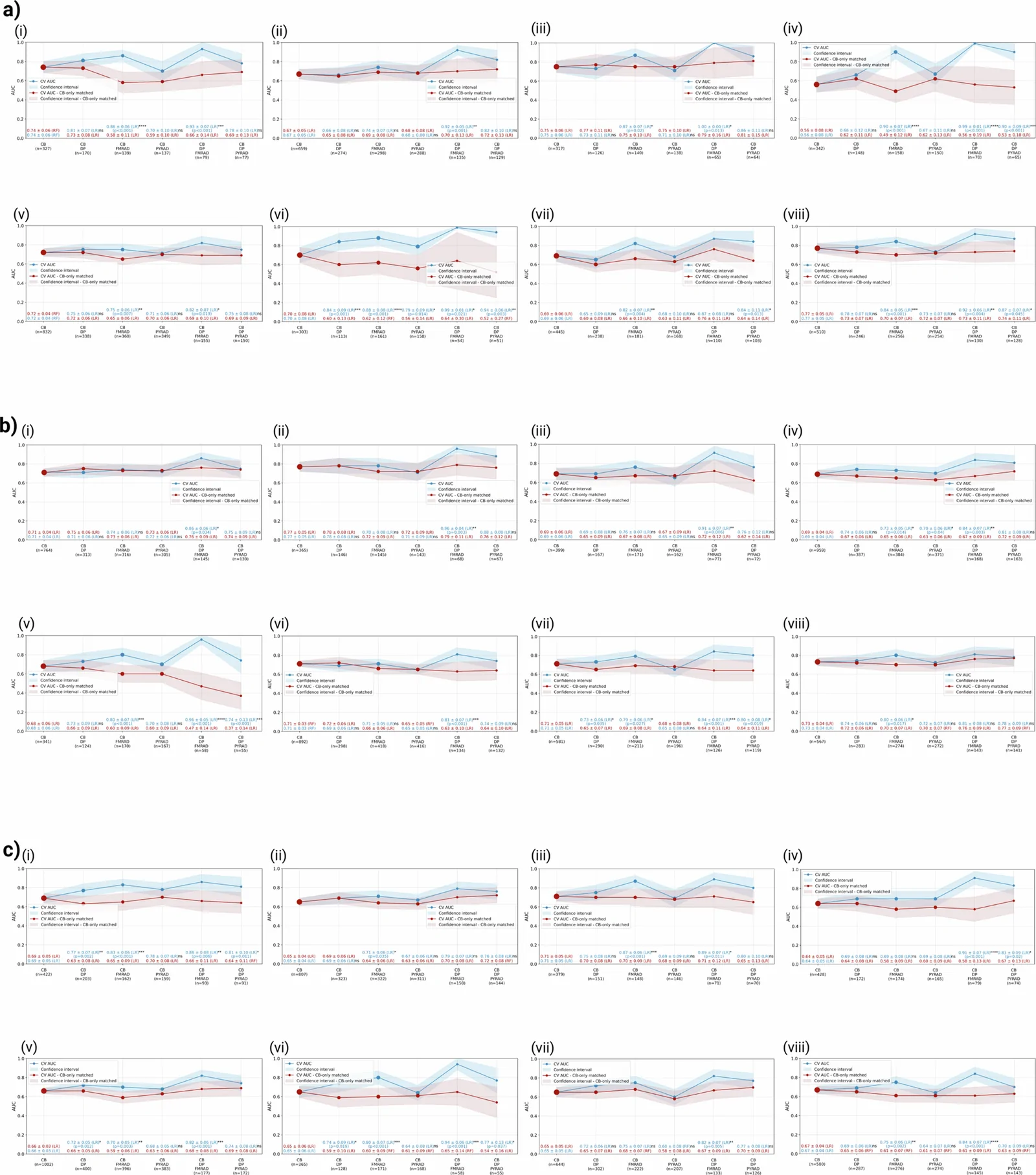
MLEF Framework. **a**) CV AUC for OS24 (i) C23 PDL1≥50%, (ii) C23 PDL1<50%, (iii) C23 PDL1 1-49%, (iv) C23 PDL1 <1%, (v) C23 adeno, (vi) C23 squamous, (vii) C23 IO/CHT, (viii) C23 INT; **b**) CV AUC for OS6 (i) C23 PDL1<50%, (ii) C23 PDL1 1-49%, (iii) C23 PDL1 <1%, (iv) C23 adeno, (v) C23 squamous, (vi) C23 IO-only, (vii) C23 IO/CHT, (viii) C23 INT; **c**) CV AUC for DCR (i) C23 PDL1≥50%, (ii) C23 PDL1<50%, (iii) C23 PDL1 1-49%, (iv) C23 PDL1 <1%, (v) C23 adeno, (vi) C23 squamous, (vii) C23 IO/CHT, (viii) C23 INT; AUCs shown with 95% confidence intervals, represented by shaded areas and calculated using DeLong’s method; Circle size indicates training set size; Multimodal (blue) and CB-only matched (red) models were compared using DeLong’s two-sided z-test. n.s., not significant; *p≤0.05; **p≤0.01; ***p≤0.001; ****p≤0.0001. *Created in BioRender. PRELAJ*, *A*. (2026) https://BioRender.com/ulcxt65.

For OS24, multimodal approaches outperformed CB-only matched models in IO-only patients (AUC 0.91 versus 0.54, *P* ≤ 0.0001; Fig. 2f(i)) and other subgroups (Extended Data Fig. 3a). Similar trends were observed for OS6 (for example, PD-L1 ≥ 50%, AUC 0.89 versus 0.61, *P* ≤ 0.0001; Fig. 2f(ii)) and DCR (for example, IO-only, AUC 0.84 versus 0.60, *P* = 0.00011; Fig. 2f(iii)). For survival models, improvements were seen in PD-L1 ≥ 50%, squamous and IO-only groups. A summary of CV AUCs across all endpoints and remaining subgroups is shown in Fig. 2g, with full metrics reported in Extended Data Fig. 3 and Supplementary Information 3. Detailed subgroup genomic analyses (KRAS, P53 and STK11) are in Supplementary Information 2, Table 4 and Fig. 6.

### DL models performed similarly to ML for CB-only models with no added benefit from multimodal integration

**Fig. 3:**
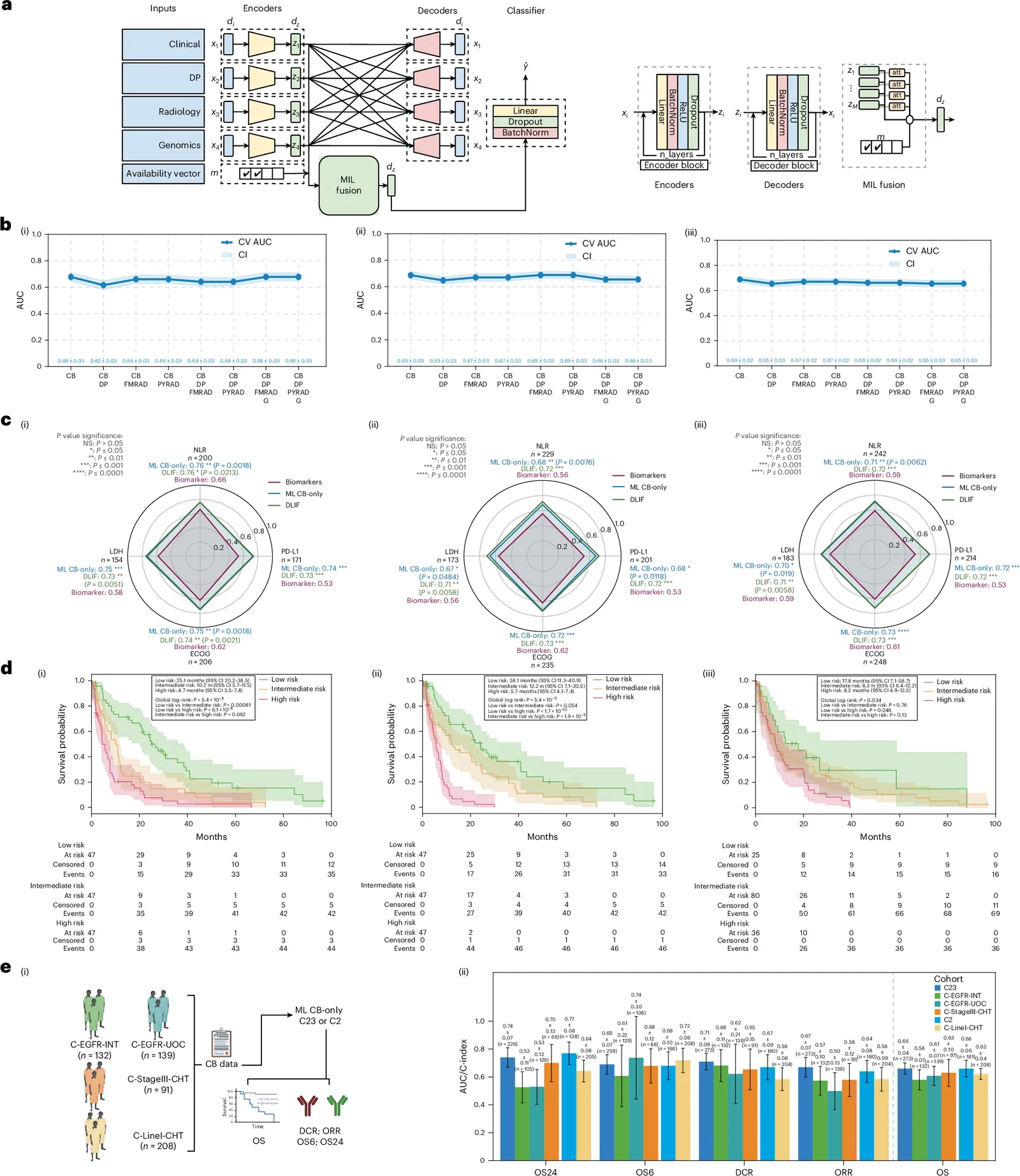
DLIF performance and single-biomarker comparison. **a**, Schematic overview of the DLIF architecture designed to handle missing modalities. **b**, Classification performance of C23 unimodal, bimodal and multimodal DLIF models for (i) OS24, (ii) OS6 and (iii) DCR on CV; AUC values were calculated using DeLong’s method; shaded areas represent 95% CIs. **c**, Direct comparison among MLEF CB-only, DLIF (best-performing multimodal model on CV) and single biomarkers for (i) OS24 (DLIF CB±FMRAD), (ii) OS6 (DL CB) and (iii) DCR (CB DP+FMRAD) in the C23 cohort on TEST; performance was assessed using AUC and tested with DeLong’s method; *P* values were calculated using a two-sided *z*-test. **d**, Kaplan–Meier curves of low-risk, intermediate-risk and high-risk patients: (i) separation by tertiles from Cox-ML on TEST LIPI subset (*n* = 141), (ii) separation by tertiles from Cox-DL on TEST LIPI subset (*n* = 141) and (iii) separation by LIPI score on TEST subset with LIPI available (*n* = 141). *P* values were calculated using the log-rank test. **e**, Predictive value of CB-only models: (i) non-IO cohorts (EGFR and CHT) and (ii) AUC/C-index bar plot for TEST in IO and non-IO cohorts. AUC values were calculated using DeLong’s method; error bars represent 95% CIs. Figure created in BioRender; Prelaj, A. https://biorender.com/ulcxt65 (2026).

**Fig. 4:**
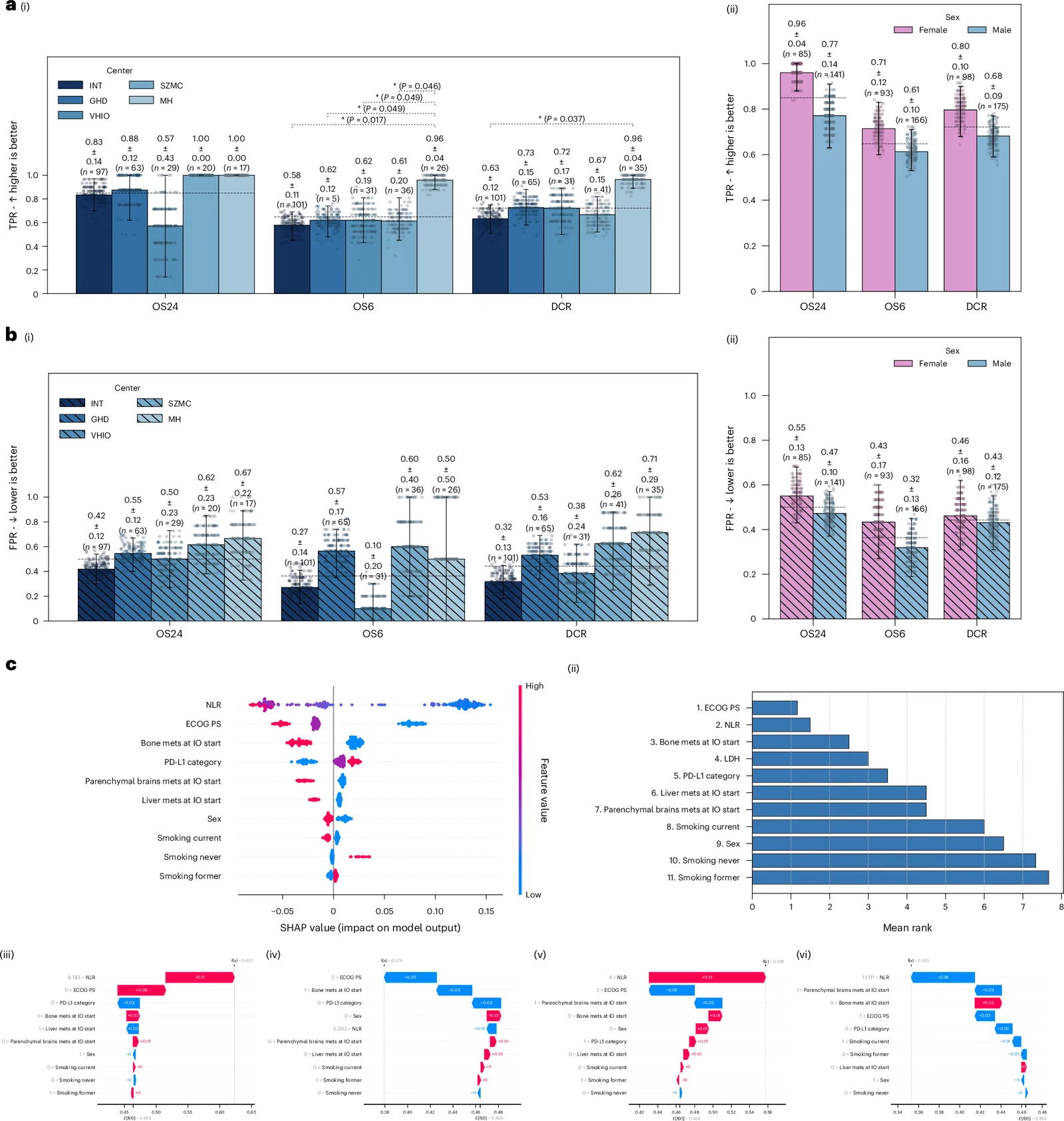
Fairness (equalized odds) and XAI analysis of CB-only ML models. **a**, Sensitivity (TPR) in TEST across protected attributes: (i) center and (ii) sex; black dashed line indicates TPR value across full TEST set. Error bars represent 95% CIs calculated using bootstrapping. *P* values were calculated using a permutation test with 1,000 iterations and a two-sided test for comparison between the 2 groups. *P ≤ 0.05, **P ≤ 0.01, ***P ≤ 0.001, ****P ≤ 0.0001. **b**, FPR in TEST across protected attributes: (i) center and (ii) sex; black dashed line indicates FPR value across full TEST set. Error bars represent 95% CIs calculated using bootstrapping. *P* values were calculated using a permutation test with 1,000 iterations and a two-sided test for comparison between the 2 groups. *P ≤ 0.05, **P ≤ 0.01, ***P ≤ 0.001, ****P ≤ 0.0001. **c**, Post hoc global explainability for OS24 model: (i) global summary SHAP plot for TEST; (ii) mean-ranked feature importance based on SHAP global values across OS24, OS6 and DCR outcomes for TRAIN; local SHAP explanation for four patients from TEST, true positive (iii), true negative (iv), false positive (v) and false negative (vi). More detailed explanation about the graphs is given in Methods. Mets, metastasis. Figure created in BioRender; Prelaj, A. https://biorender.com/ulcxt65 (2026).

**Fig. 5:**
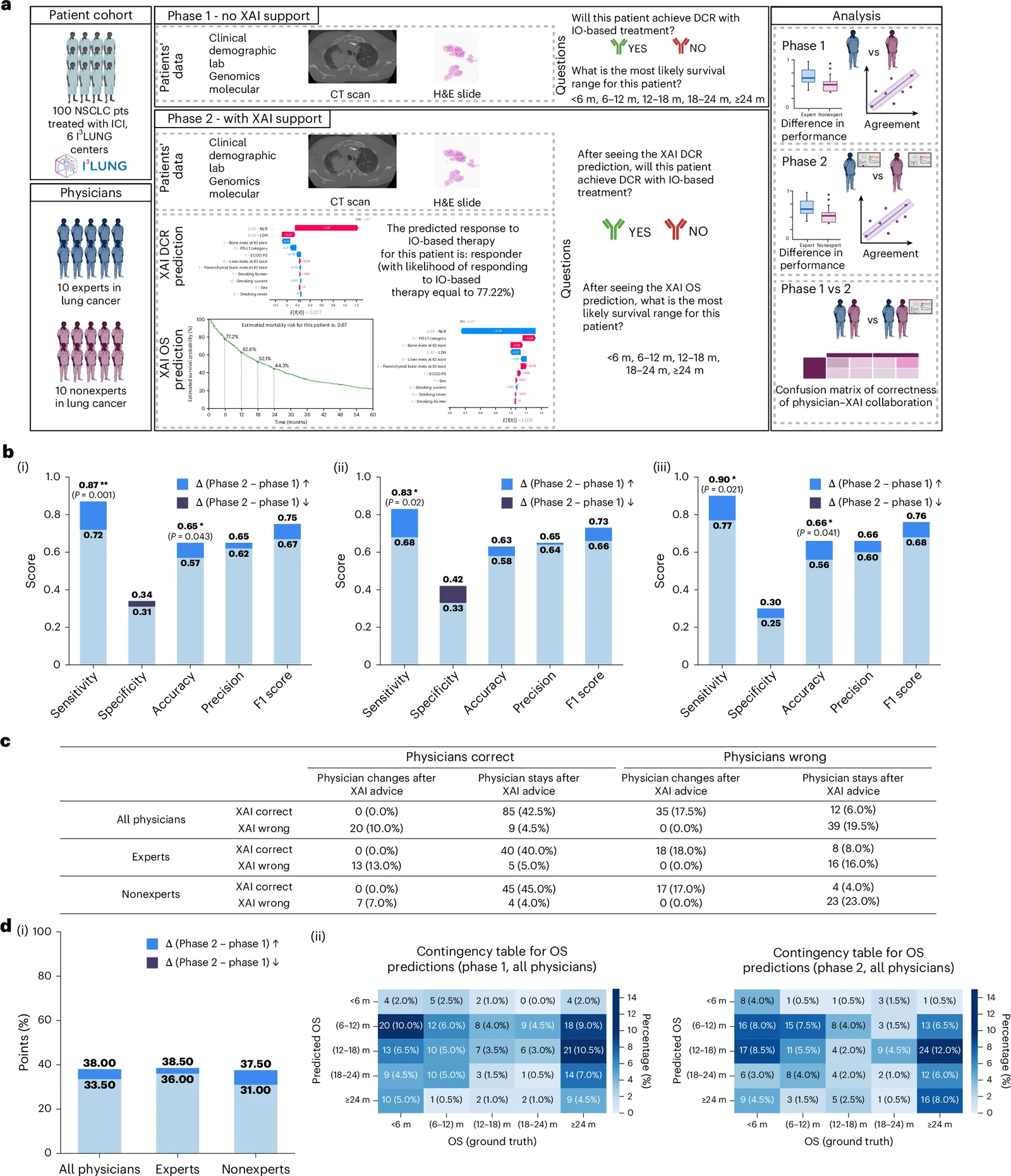
Clinical usability of ML CB-only models. **a**, Schematic overview of clinical usability study. We selected 100 patients (80 from TEST set and 20 from EXVAL set). Ten lung cancer experts and 10 nonexperts evaluated 10 patients each for 2 endpoints, DCR and OS, first in phase 1 using just CB data and images when available and in phase 2 using CB data, images when available and CB-only MLEF (logistic regression and Cox) output together with global and local explainability for each patient. We analyzed the difference in performance in phase 1 and phase 2 among experts and nonexperts, their agreement and, finally, the difference in performance in phase 1 versus phase 2. **b**, Metrics summary for phase 1 versus phase 2 for DCR prediction for (i) all physicians, (ii) experts and (iii) nonexperts, with *P* values calculated using two-sided McNemar’s test. *P ≤ 0.05, **P ≤ 0.01, ***P ≤ 0.001, ****P ≤ 0.0001. Statistical test for F1 was not performed. **c**, Confusion matrix of correctness of human–XAI collaboration in phase 2 for DCR, illustrating whether physicians changed or maintained their prediction after XAI advice. **d**, Comparison of phase 1 versus phase 2 for predicting OS range: (i) total score for OS predictions for phase 1 and phase 2 according to physicians’ expertise and (ii) contingency table for OS predictions in phase 1 (left) and phase 2 (right) for all physicians. ICI, immune checkpoint inhibitor; pts, patients. Figure created in BioRender; Prelaj, A. https://biorender.com/ulcxt65 (2026).

**Extended Data Fig. 4:**
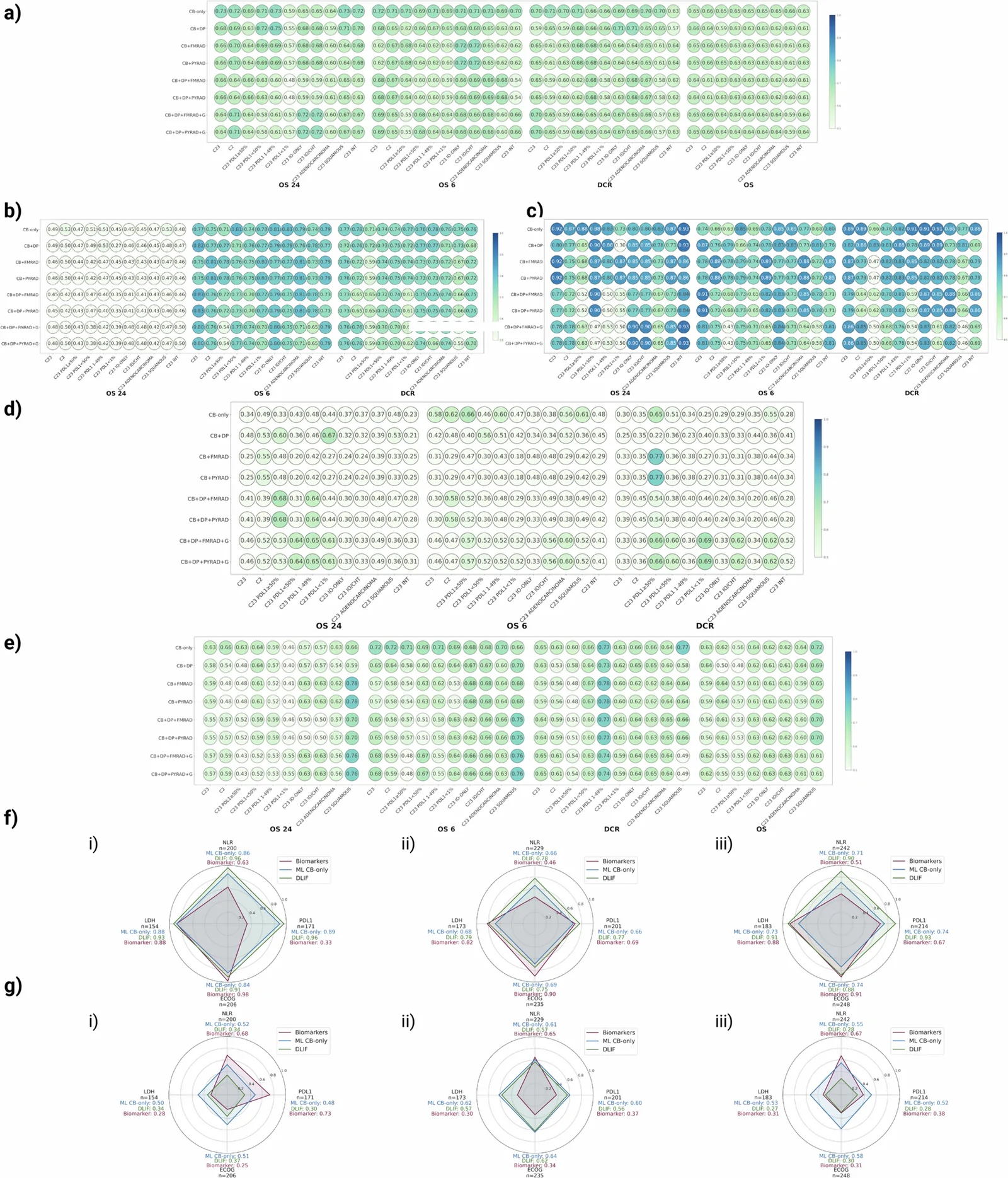
DLIF Framework. **a**) TEST AUC of DLIF classifier and C-index of Cox-DLIF, **b**) TEST F1 of DLIF classifier, **c**) TEST Sensitivity of DLIF classifier, **d**) TEST Specificity of DLIF classifier, **e**)EXVAL AUC of DLIF classifier **f**) Direct comparison between MLEF, DLIF and single biomarkers for (i) OS24, (ii) OS6, and (iii) DCR in the C23 cohort on TEST; performance assessed using Sensitivity **g**) Direct comparison between MLEF, DLIF and single biomarkers for (i) OS24, (ii) OS6, and (iii) DCR in the C23 cohort on TEST; performance assessed using Specificity. *Created in BioRender. PRELAJ*, *A*. (2026) https://BioRender.com/ulcxt65.

DL CB-only models (Fig. 3a) achieved similar performance to ML: with AUC 0.73 in TEST (95% CI: 0.66–0.80) and 0.63 (95% CI: 0.55–0.71) in EXVAL for OS24; AUC 0.72 (95% CI: 0.65–0.79) in TEST and 0.72 (95% CI: 0.64–0.80) in EXVAL for OS6; AUC 0.70 (95% CI: 0.64–0.76) in TEST and 0.65 (95% CI: 0.58–0.72) in EXVAL for DCR (Fig. 3b(i)–(iii)). Regardless of the outcome, the DLIF approach did not benefit from adding other modalities. This held across endpoints and in subgroup analyses, including first-line treatment (Extended Data Fig. 4a–e). Similarly, DL survival analysis showed no benefit from multimodal integration (Extended Data Fig. 4a,e). Additionally, we tested two other DL alternative fusion approaches, one of them with late fusion architecture^13,16^, achieving similar results compared to DLIF (Supplementary Information 4, Section 1, Figs. 1–5). DLIF robustness across populations with different modality availability is reported in Supplementary Information 4, Section 2, Figs. 6–21.

### ML and DL CB-only models outperformed single biomarkers and better stratified patients than the LIPI score

In the TEST for the C23 without missing biomarker data for OS24, MLEF and DLIF CB-only models outperformed single biomarkers: PD-L1 (AUCs for ML versus DL versus single biomarker: 0.74 versus 0.73 versus 0.53), ECOG PS (0.75 versus 0.74 versus 0.62), LDH (0.75 versus 0.73 versus 0.58) and NLR (0.76 versus 0.76 versus 0.66), with *P*≤ 0.05 for all comparisons (Fig. 3c(i)). For OS6 and DCR, results are reported, respectively, in Fig. 3c(ii) and (iii).

However, the difference observed among AUC values for ML and DL models versus single biomarkers was not consistent across all other metrics (Extended Data Fig. 4f,g).

In the TEST subset with available LDH and NLR data (*n* = 141), both survival models effectively stratified risk groups. The ML model significantly separated low versus high (25.1 months versus 4.7 months, *P* = 6.1 × 10^−8^) and low versus intermediate (25.1 months versus 10.2 months, *P* = 0.00081) but not intermediate versus high (Fig. 3d(i)). The DL model showed significant separation for low versus high (24.1 months versus 5.7 months, *P* < 0.0001) and intermediate versus high (12.2 months versus 5.7 months, *P* < 0.0001) but not low versus intermediate (Fig. 3d(ii)). By contrast, LIPI only distinguished low versus high risk and failed in other comparisons (Fig. 3d(ii)). In the full TEST cohort (*n* = 273), ML-based analysis significantly separated all three risk groups (global *P* < 0.000001), supporting improved stratification over standard approaches (Extended Data Fig. 2). Cox-ML (C-index 0.65, 95% CI: 0.60–0.70) and Cox-DL (C-index 0.63, 95% CI: 0.58–0.68) outperformed LIPI^17^ (C-index 0.55, 95% CI: 0.50–0.60).

### ML models showed greater predictive performance in IO cohorts compared to CHT and EGFR cohorts

We evaluated the ML CB-only C23 model against multiple non-IO cohorts to assess its predictive value, including two independent EGFR-mutant cohorts (C-EGFR-INT and C-EGFR-UOC) and one CHT cohort (C-StageIII-CHT) (Fig. 3e(i) and Methods). Overall, across non-IO cohorts, the model showed less stable performance, particularly for response endpoints (ORR AUC: 0.57 ± 0.10 and 0.50 ± 0.13 in C-EGFR-INT and C-EGFR-UOC; ORR AUC 0.58 ± 0.12 in C-StageIII-CHT) (Fig. 3e(ii)).

Additionally, we tested the ML CB-only C2 model performance on patients from C3 who received first-line CHT from INT (C-LineI-CHT). In this setting, OS discrimination remained similar (AUC 0.72 ± 0.09 for OS6; AUC 0.64 ± 0.08 for OS24), potentially reflecting the impact of subsequent IO on OS while response endpoints remained lower (ORR/DCR AUC both 0.58), suggesting predicting capability of IO models (Supplementary Information 2, Table 5).

### Fairness evaluation of the ML CB-only models revealed variability in sensitivity across centers

**Extended Data Fig. 5:**
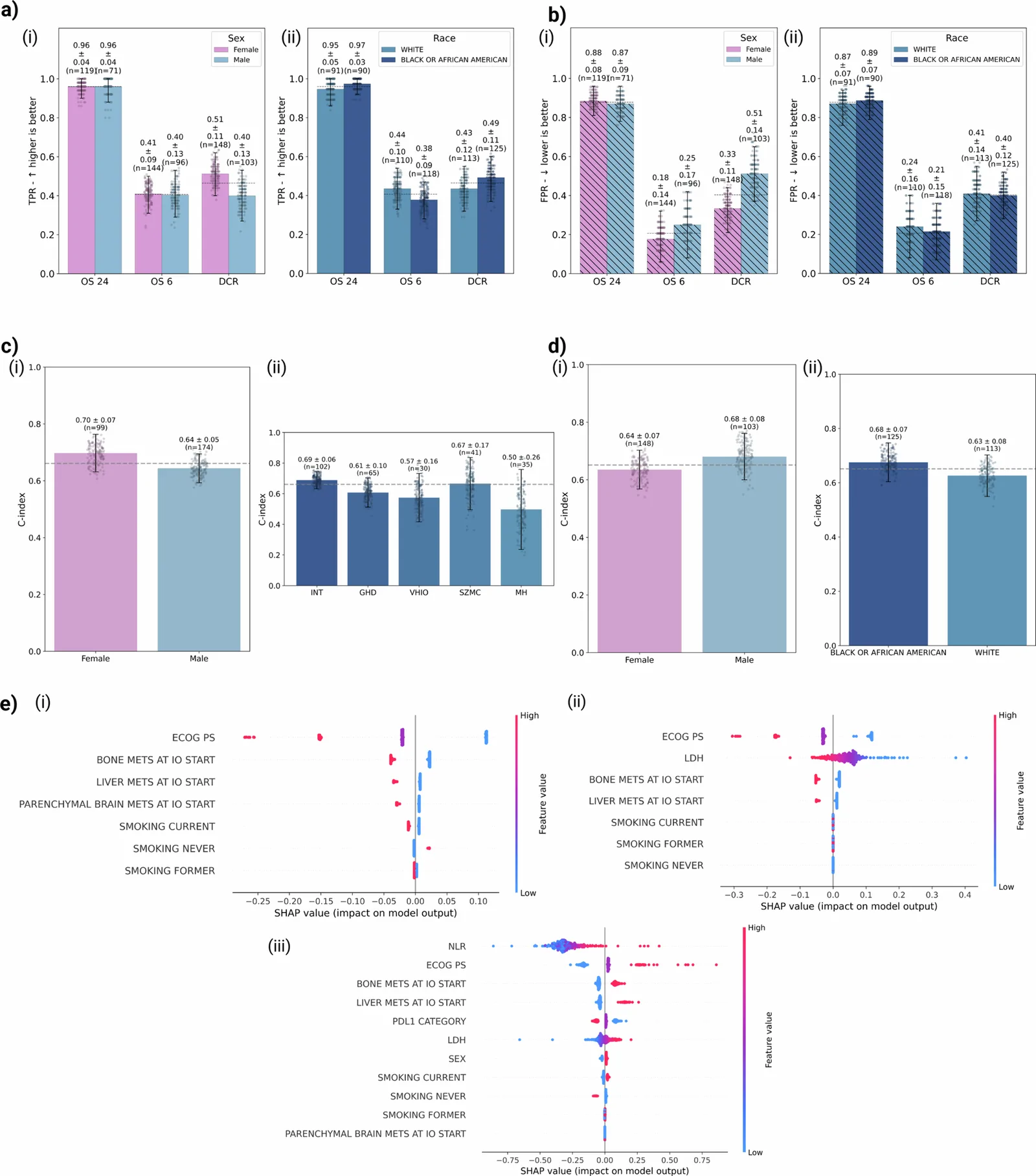
Fairness and post-hoc XAI analysis. **a**) EXVAL TPR across sex (i) and self-reported race (ii), **b**) EXVAL FPR across sex (i) and self-reported race (ii), **c**) TEST c-index across sex (i) and centers (ii), **d**) EXVAL c-index across (i) self-reported race and (ii) sex; **e**) global SHAP plots for OS6 (i), DCR (ii), and OS (iii). For panels a-d, error bars represent 95% confidence intervals calculated by bootstrapping. *Created in BioRender. PRELAJ*, *A*. (2026) https://BioRender.com/ulcxt65.

When using center as a protected attribute in TEST, the MH subgroup achieved significantly higher sensitivity for OS6 prediction (true-positive rate (TPR) = 0.96) compared to other centers, with *P* ≤ 0.05 (Fig. 4a(i)). For the DCR endpoint, a significant difference in sensitivity was observed between MH and INT (0.96 versus 0.63, *P* = 0.037; Fig. 4a(i)). By contrast, sensitivity was not statistically different when using sex as a protected attribute (Fig. 4a(ii)). For false-positive rate (FPR), no statistically significant differences were observed across centers or between sex (Fig. 4b(i),(ii); detailed analysis in Supplementary Information 5, Section 1, Tables 1–5 and Figs. 1–4). Fairness analysis in EXVAL showed that classification models performed equally across self-reported race and sex (Extended Data Fig. 5). For the OS survival model (Cox-ML), TEST C-index varied across centers; EXVAL C-index across self-reported races showed modest disparities when using Cox (Black/African American = 0.68, White = 0.63, without statistical significance) (Extended Data Fig. 5). Fairness analysis of the DL models is reported in Supplementary Information 5, Section 2, Fig. 5.

### XAI analysis for ML CB-only models aligns with established prognostic factors

Across endpoints, ECOG PS greater than 0 and the presence of bone, liver and brain metastases consistently emerged as strong negative prognostic factors for OS24 (Fig. 4c(i),(ii)). NLR was additionally confirmed as important for OS24 and OS analysis, and high LDH influenced DCR nonresponder predictions (Extended Data Fig. 5eii). Examples of local explainability graphs are presented for four patients from the TEST for OS24 in Fig. 4c(iii)–(vi). These types of graphs were further used for the clinical usability study (Fig. 5a).

### Physician–XAI collaboration improved predictions

To estimate the clinical benefit of the ML CB-only baseline models (logistic regression for DCR and Cox for OS, trained on all hypothesis-driven CB features), we conducted a clinical usability study. No multimodal models were used in this study. The study involved 20 physicians: 10 lung expert oncologists and 10 nonexperts in lung cancer (including resident doctors and general oncologists), in pairs. Each physician evaluated 10 real-world NSCLC cases from the TEST set, totaling 200 assessments. The detailed design is shown in Fig. 5a. Physicians were asked to estimate DCR (yes versus no) and OS (split into five categories: <6, 6–12, 12–18, 18–24 and ≥24 months) first without (phase 1) and then with (phase 2) support from the models and SHapley Additive exPlanations (SHAP)-based explainability (see Methods and Supplementary Information 6, Tables 1–40, for more details on design and full analysis).

For DCR prediction, physician–XAI collaboration improved sensitivity for all physicians from 0.72 (95% CI: 0.64–0.80) to 0.87 (95% CI: 0.79–0.92), *P* = 0.0011, and accuracy from 0.57 to 0.65, *P* = 0.0431, at the expense of slightly lower specificity (Fig. 5b(i)). Both experts and nonexperts benefited from the use of XAI (sensitivity from 0.68 to 0.83, *P* = 0.0201, Fig. 5b(ii), and from 0.77 to 0.90, *P* = 0.0209, Fig. 5b(iii), respectively). No statistical differences between groups were observed in either phase (Supplementary Information 6, Tables 28 and 34). Overall, XAI use was associated with a 37% increase in the correct DCR prediction among physicians (odds ratio = 1.37, *P* = 0.1). Similarly, non-statistically significant improvements of +53% and +23% correct DCR predictions for nonexperts and experts was observed, respectively. The agreement between experts and nonexperts improved from slight (*κ* = 0.11) to moderate (*κ* = 0.48) from phase 1 to phase 2. Overall, physicians frequently adopted correct XAI suggestions (74.5%), but experts were more likely to follow incorrect ones (72.2% versus 63.6% for lung experts and nonexperts, respectively) (Fig. 5c).

In the OS estimation task, XAI increased the probability of correct prediction by 36% across all physicians—14% for experts and 61% for nonexperts. A scoring system (see Methods for details) for correctness showed an increased total score for all physicians by 4.5%, for nonexperts by 6.5% and for experts by 2.5% (Fig. 5d(i)). In phase 1, 16.5% of the physicians’ evaluations were concordant with the true OS; such a proportion increased to 22.5% after using the XAI (Fig. 5d(ii)). Finally, agreement between the experts and nonexperts improved from slight to fair (*κ* from 0.14 to 0.34).

## Discussion

I^3^LUNG is, to our knowledge, the largest international, real-world multimodal study in NSCLC IO and AI, encompassing 2,396 patients from six centers in six countries, including 339 patients with complete data modalities and 400 patients with CB+RAD+DP. By combining CB, CT scans, histopathology slides and genomics with fairness audits, XAI and clinical usability, this work provides a comprehensive framework for evaluating AI-based decision support systems in immuno-oncology. Our analysis demonstrated that the AI-based predictive models using only routine CB data outperformed single biomarkers (PD-L1, ECOG PS, NLR and LDH) and the composite LIPI score. A notable finding is the robustness of CB-only models (both ML and DL) across cohorts and endpoints. Their stable performance in TEST (AUC up to 0.77 in C2) suggests that CB data capture much of the prognostic signal. The drop of the performance in the geographically distinct EXVAL cohort likely reflects differences in baseline characteristics, outcomes and treatment patterns. Such distributional shifts can influence discrimination metrics even when the underlying predictive relationships remain valid. A prospective multicenter study with predefined calibration will be essential to ensure model stability across heterogeneous real-world settings.

Although adding imaging modalities (DP and FMRAD/PYRAD) improved performance in the MLEF CV model (AUC up to 0.88), these gains were not consistently replicated in TEST and EXVAL, likely due to limited multimodal data. Complete-case analyses introduce potential selection bias, as these subsets may not fully represent the broader population. DL models require large datasets to capture crossmodal interactions, which may explain why DLIF performs similarly to CB-only models without added multimodal benefit. We tested an additional DL fusion model^16^, which handles missing modalities differently, and a late fusion strategy^13^. Results were similar, suggesting that the lack of clear gains was driven by incomplete or heterogeneous retrospective data rather than model architecture.

These results align with previous large-scale, CB-based efforts such as SCORPIO^18^, which achieved AUC values of 0.76 (OS) and 0.64–0.71 (clinical benefit) using CB from more than 9,000 patients to outperform traditional biomarkers (tumor mutational burden (TMB) and PD-L1). Our unimodal models based on FMRAD, DP or genomics alone performed similarly to other single-modality efforts in literature. For instance, Saad et al.^10^ reported a C-index of 0.75 using DEEP-CT, a DL model developed with 976 baseline chest CT scans, combined with clinical data. Rakaee et al.^11^ achieved an AUC of 0.75 internally and 0.66 in external validation with hematoxylin and eosin (H&E) slide-based prediction (*n* = 958). A great effort to overcome these challenges is represented by the LORIS model developed by Chang et al.^19^, a pan-cancer logistic regression framework integrating six clinical, TMB and pathologic features (AUCs up to 0.83 for IO response). The validation on multiple unseen datasets achieved an AUC of 0.74 for the NSCLC-specific model. However, compared to our study, the models did not perform a clinical usability study.

Few studies using multimodal data in NSCLC and IO have previously been published, and all of them include small cohorts and single-center designs^13,14^. For instance, Vanguri et al.^13^ developed DyAM, integrating CT radiomics, PD-L1 slides and genomics to predict IO response in NSCLC, achieving AUC of 0.80 (95% CI: 0.74–0.86). Although representing a pioneering effort in developing a rigorous methodology for handling missing data modalities, the study was limited by its modest sample size (*n* = 247; 81 fully multimodal) and single-center design. More recently, Captier et al.^14^ combined PET/CT, pathology, transcriptomics and clinical data from 317 patients with NSCLC (80 fully multimodal), achieving an AUC of 0.81 and a C-index of 0.75. In both studies, TEST or EXVAL cohorts were missing, limiting the generalizability and positioning I^3^LUNG as the largest multimodal study in NSCLC and IO.

Emerging work by Saad et al.^20^ extends the efforts toward identifying patients more likely to benefit from the addition of CHT to IO.

To assess treatment-specific predictive value from general prognostic outcomes, we evaluated the ML CB-only (C23 and C2) models in four additional non-IO cohorts (two CHT and two EGFR-mutant). The model discrimination was strongest and most stable in the IO-based test cohort, particularly for response endpoints, which may better reflect objective benefit from IO. These preliminary comparisons suggest that our models may capture IO-specific predictive patterns rather than purely baseline prognosis.

In our work, fairness audits across protected attributes revealed variability in sensitivity across centers, likely driven by heterogeneous data quality and TEST sample imbalances such as the higher TPR in the MH. ECOG PS (clinician dependent and maybe institution dependent) drove center-level variability, and a center prediction model showed low discrimination, suggesting minimal center-specific bias. Although differences in Cox-ML performance by self-reported race were not statistically significant, they underscore the need for fairness-aware calibration and site-specific validation before clinical deployment.

The clinical usability study demonstrated that XAI applied to CB-only models can support physician decision-making. Sensitivity for DCR prediction improved from 0.72 to 0.87 with XAI, with parallel gains in accuracy and F1 score. This sensitivity gain is clinically valuable, reducing missed prediction for patients likely to benefit from IO. OS prediction also improved modestly with XAI, with an overall gain of 36% among all physicians. Such improvements may be especially impactful in community settings lacking thoracic oncology expertise. From a clinical implementation perspective, the I^3^LUNG model may serve as a decision support tool for IO in advanced NSCLC. In particular, the model may help contextualize patients’ prognosis and treatment strategies in clinically relevant scenarios, such as identifying patients with PD-L1-high, who might benefit from IO alone and deescalation of CHT. Conversely, we can predict patients with poor outcomes (OS < 6 months) who may benefit from intensified strategies, enrollment in clinical trials or best supportive care.

This study has several limitations. First, the retrospective design and heterogeneity of real-world data may affect model performance. Second, the relatively small number of complete multimodal cases limits the reliability of multimodal analyses, particularly for DLIF. Third, more flexible architectures capable of handling incomplete data will be essential for clinical deployment compared to MLEF (for example, transformers, multimodal foundation models and agentic AI systems, such as MSK-CHORD^21^, MUSK^22^ and APOLLO^23^). Fourth, genomic data were sparse, and radiomic features were restricted to primary lesions, potentially missing relevant biological information. In addition, approximately 15% of used CT scans were not at baseline IO. Finally, EXVAL was limited to a single cohort with different baseline characteristics, highlighting the need for prospective validation in larger datasets.

In conclusion, I^3^LUNG demonstrates that curated real-world clinical data outperform traditional single biomarkers in predicting outcomes in NSCLC IO. Although retrospective, the clinical usability study provides a reproducible and structured reference framework for future evaluation of PDSS usefulness and XAI analysis in real-world clinical settings, aligning with the FUTURE-AI^24^ framework. Although multimodal integration remains promising, its added value needs to be validated. In fact, to bring the model into clinical practice, a clinical implementation pathway is underway, including an ongoing usability study 2.0 (for the multimodal model), a silent prospective validation in 2,000 patients and a planned pragmatic randomized trial to support regulatory approval, with deployment expected within 2–3 years.

## Methods

The I^3^LUNG project (NCT05537922) uses a two-phase approach to develop a medical device to predict IO efficacy in patients with NSCLC. This framework integrates multimodal clinical and multiomics data. Here we report the results from the retrospective phase, focused on developing a preliminary predictive model for IO outcomes that served as the foundation for the initial PDSS version.

### Patient enrollment

This study included retrospective data from 2,396 patients with stage IIIC–IVB NSCLC who received IO-based therapy between September 2012 and October 2023 across 6 international institutions: INT-Italy, GHD-Germany, MH-Greece, SZMC-Israel, VHIO-Spain and UOC-USA. Patients with available baseline data and known clinical outcomes (response to IO according to Response Evaluation Criteria In Solid Tumors (RECIST) OS) were included in the analysis. All patients were enrolled as part of the I^3^LUNG international, multicenter, retrospective and prospective observational study (NCT05537922). Eligibility criteria included adults aged 18 years or older and a histologically confirmed diagnosis of stage IIIC–IVB NSCLC according to the 8th edition of the TNM staging system. The study was conducted in compliance with the Declaration of Helsinki and Good Clinical Practice guidelines, with ethics approval obtained at each participating site.

### Clinical data

The study population was subdivided into three patient cohorts based on NSCLC disease stage:

- C1, Curative Setting: patients with stage III NSCLC treated with curative-intent chemoradiation therapy (concomitant or sequential) followed by maintenance IO.
- C2, Non-Curative Setting, First Metastatic Line: includes patients with advanced or metastatic NSCLC who received first-line IO, either alone or in combination with CHT or other therapeutic agents.
- C3, Non-Curative Setting, Later Lines: includes patients with advanced or metastatic NSCLC treated with IO in any line beyond first-line, either alone or in combination with CHT or other agents.

For patients who received IO in both curative and non-curative settings, their data were duplicated and included in both C1 and either C2 or C3, depending on their metastatic treatment line. CONSORT flow is shown in Extended Data Fig. 1a.

The data curation process began with involved hypothesis-driven feature selection, from more than 11,000 features from the electronic case report forms within the centralized I^3^LUNG platform. Features were categorized as (1) baseline features: CB and genomics features available before the administration of the first IO cycle and (2) treatment-related features: associated with treatment efficacy and toxicity. To reduce dimensionality, two independent blinded groups of oncologists (L.P. and C. Silvestri versus A.S. and C.G.) performed feature review and selection. A total of 227 features, including age at IO start and patient’s comorbidities (full list provided in Supplementary Information 1, Table 1), were consistently selected by both groups. These baseline IO features were further divided into (1) descriptive features, used for exploratory analysis and submodel development, and (2) predictive features, used to train the models. The final feature selection was performed by M.C.G. and A.P., senior oncologists with extensive experience in lung cancer treatment, resulting in a final set of nine CB features used for model development.

The second step focused on data cleaning and quality control, including standardization of free-text entries and correction of inconsistencies in dates and numerical values. Quality control was performed through three iterative validation rounds with participating centers to resolve missing values, inaccurate entries and dataset discrepancies, thereby improving overall data reliability and consistency.

### New non-IO cohorts

To address the question of the predictive value of our models, we have collected and curated data from four additional cohorts; three of them are not part of the I^3^LUNG study. In detail:

1. C-EGFR-INT: patients with EGFR-mutant NSCLC treated with EGFR-TKIs (from INT)
2. C-EGFR-UOC: patients with EGFR-mutant NSCLC treated with EGFR-TKIs (from UOC)
3. C-StageIII-CHT: patients with stage III NSCLC treated with neoadjuvant CHT (from INT) and followed by surgery
4. C-LineI-CHT: patients from I^3^LUNG C3 who received first-line CHT before second-line IO (baseline CHT features used)

### Clinical endpoints

We used two types of clinical endpoints to evaluate treatment efficacy: response outcomes and survival outcomes. Response outcomes were assessed using RECIST version 1.1, leading to the definition of the key response metric, DCR, where patients were classified as:

- Nonresponders (class 0): progressive disease (PD)
- Responders (class 1): stable disease (SD), partial response (PR) and complete response (CR)

We have used two additional response metrics:

1. ORR:
  - Nonresponders (class 0): PD and SD
  - Responders (class 1): PR and CR
2. CBR:
  - Nonresponders (class 0): PD and SD with PFS < 6 months
  - Responders (class 1): PR, CR and SD with PFS ≥ 6 months

We selected OS as the time from initiation of IO to death or last follow-up to assess treatment durability. Progression-free survival (PFS) was defined as the time from the initiation of IO therapy to disease progression or death.

The primary endpoints of the study included the identification of long responders (OS at 24 months (OS24)), poor responders (OS at 6 months (OS6)) and DCR for classification tasks and OS for survival analysis.

For both OS6 and OS24, we defined binary survival endpoints using a landmark approach:

1. OS6:
  - Class 0: patients who died within 6 months from treatment initiation
  - Class 1: patients who survived ≥6 months
2. OS24:
  - Class 0: patients who died within 24 months
  - Class 1: patients who survived ≥24 months

To avoid misclassification and follow-up bias, patients with follow-up shorter than the respective landmark time who were still alive at last contact were excluded from the corresponding analysis. This ensured that survival status at the landmark timepoint could be determined with certainty for all included patients.

The secondary endpoints of the study included ORR and CBR for classification tasks. Results on these endpoints are shown in Supplementary Information 2 (Figs. 1 and 2) and Supplementary Information 4 (Figs. 1 and 2).

Full statistical analysis of the CB across centers and cohorts is shown in Supplementary Information 1.

### CT scans

CT scans were collected in Digital Imaging and Communications in Medicine (DICOM) format and underwent quality control, harmonization and segmentation. Scans were predominantly acquired at baseline prior to IO initiation. Given the real-world design of the study, when a baseline IO CT was unavailable or when the primary lesion was not present for a previous surgery, scans obtained at diagnosis were included.

Given the multicenter real-world design, CT acquisition was heterogeneous, and CT scans with missing metadata, usage of nonstandard or soft tissue kernels, artifacts, slice thickness out of the range 1–5 mm (for example, 10 mm), clinical exclusion (for example, patients with concurrent malignancies or without pulmonary lesion) and corrupted DICOM were excluded (Extended Data Fig. 1bii). Segmentations were performed in a three-dimensional slicer, using a semiautomated approach with manual refinement by a single trained radiologist. Difficult cases were reviewed by a senior radiologist (B.R.N.), ensuring consistent segmentation across all cases with no interoperator variability. The final segmentation masks were exported as NRRD files by the team at SZMC.

Of 896 CT scans used, 110 patients (12–33% in C2 and 77% in C3) did not have available pre-IO CT scan, and 41 (5–26% in C2 and 15% in C3) received surgery. For 23 patients (3–16% in C2 and 7% in C3), CT scans were collected after IO initiation. The distribution of time intervals between CT acquisition and IO initiation is provided in Supplementary Information 7, Fig. 1a. To assess their impact, we reran the models with and without these CT scans; performance remained stable, and multimodal improvements were maintained.

When available, contrast‑enhanced scans were preferred; of 896 CT scans, 758 (84.6%) were contrast, and 138 (15.4%) were noncontrast. Robustness analyses were performed to assess PYRAD features extracted from contrast versus noncontrast CT scans. Differences were observed, likely due to the limited number of noncontrast images (Supplementary Information 7, Robustness Analysis). However, excluding noncontrast images did not impact performance in either unimodal or selected multimodal models using PYRAD features. Available metadata analysis, with focus on manufacturer, slice thickness and contrast information of the included CT scans, is shown in Supplementary Information 7, Fig. 1bi–iii across TRAIN, TEST and EXVALl and in Fig. 1ci–iiiby center. To assess the robustness and discriminative power of radiomic features, we applied a contour randomization approach to each lesion segmentation delineated by the radiologist team at SZMC. For each lesion, we generated 10 randomized versions of the original mask, resulting in 11 segmentations per lesion (1 original + 10 perturbed). Radiomic features were extracted from each of these segmentations using PYRAD, resulting in 1,400 features per setting. To evaluate feature stability and relevance, we applied a statistical framework based on the inverse coefficient of selection, IS_*f*_. This metric captures both intrasample robustness ${\sigma }_{\mathrm{intra},f}^{2}$ and intersample discriminability ${\sigma }_{\mathrm{inter},f}^{2}$. Let $l\in \{1,\ldots ,L\}$, $p\in \{1,\ldots ,P\}$, $f\in \{1,\ldots ,F\}$ denote lesion, perturbation and feature indices, respectively. Formally, the inverse coefficient of selection, IS_*f*_, is defined as follows:

$${{\rm{IS}}}_{f}=\frac{{\sigma }_{\mathrm{intra},f}^{2}}{{\sigma }_{\mathrm{inter},f}^{2}}$$

where

$${\sigma }_{\mathrm{intra},f}^{2}=\frac{1}{L}\sum _{l=1}^{L}\mathrm{Var}(\{{X}_{l,p,f}{\}}_{p=1}^{P}),{\sigma }_{\mathrm{inter},f}^{2}=\mathrm{Var}(\{{X}_{l,{p}_{0},f}{\}}_{l=1}^{L}),$$

and *X*_*l*,*p*,*f*_ is the value of the feature *f* for lesion *l* and perturbation *p*. *σ*_intra_ variance was computed by assessing the average variability of each feature across all perturbation settings for a given lesion, where lower intravariance indicates higher robustness. *σ*_inter_ variance was computed as the variance of each feature across all patients in the original setting, serving as a measure of discriminative power, where higher indicates better. Finally, IS_*f*_ is defined as the ratio of intravariance and intervariance, where lower values indicate more stable and discriminative features. Features were ranked by IS_*f*_, and we then applied an iterative selection strategy to avoid redundancy: at each step, the next-best-ranked feature was added only if it was not highly correlated with any of the already selected features. This process ensured the selection of a compact, nonredundant set of 128 robust and informative radiomic features^25^. Example of expert CT segmentation with perturbations is shown in Supplementary Information 7, Fig. 1d.

Regarding the FMRAD approach, we used a domain-specific foundation model developed for cancer imaging biomarker discovery^26^ to extract high-level imaging features from CT scans. This model was pretrained using contrastive learning on a large dataset of 11,467 radiographic lesions, enabling it to learn generalized and transferable representations of tumor morphology. For our study, we applied this foundation model to volumetric image patches of size 50 × 50 × 50 mm, extracted around the centroid of the segmented primary lesion. The lesion center was determined from the segmentation mask, and the surrounding cubic volume was resampled and standardized as required by the foundation model input specifications. The resulting embeddings served as compact and expressive imaging descriptors for subsequent multimodal integration and predictive modeling. Future steps within the I^3^LUNG project include the development and evaluation of multi-lesion segmentation approaches to better capture disease heterogeneity and improve predictive performance.

### DP

DP H&E slides were obtained at the time of diagnosis or at IO initiation; when biopsy material at IO start was available, it was preferentially used. The distribution of time intervals between biopsy and DP acquisition is shown in Supplementary Information 7, Fig. 2ai,ii. We compiled a total of 998 histopathological slides for our study, sourced from five institutions: 117 slides from GHD, 364 from INT, 81 from MH, 147 from SZMC and 191 from UOC. Of these, 64 slides were excluded from further analysis due to quality control concerns, such as insufficient tissue, physical damage (that is, scratched slides), poor staining quality and other technical artifacts that could compromise downstream analysis (CONSORT flow in Extended Data Fig. 1biii). Each remaining slide was individually reviewed, with systematic annotation of pen markings, scanner-induced artifacts and out-of-focus regions. For tumor region identification, we trained a segmentation model using The Cancer Genome Atlas (TCGA) dataset. Tile extraction was performed at ×20 magnification, followed by Reinhard normalization, targeting only areas within the segmented tumor regions while avoiding the previously annotated artifacts. We used Prov-GigaPath^27^, a slide-based pretrained foundation model: first, to encode each extracted tile into a vector; then, to aggregate each bag of vectors into a single embedding representative of the whole slide Image. To determine whether our results were driven by our choice in DP feature extractor, we re-extracted 768-dimensional features using TITAN^28^, a multimodal whole-slide foundation model pretrained using self-supervised learning on whole-slide images and vision-language alignment with pathology reports. We compared our results using GigaPath DP features to those extracted by TITAN across all three outcomes and modality combinations. Results were consistent with our primary analysis: substituting TITAN features did not yield significant improvement over the CB-only model in any outcome.

### Genomics data

To include genomics data into our models, we first identified a core set of primary driver genes relevant to NSCLC: *KRAS*, *TP53*, *STK11*, *ALK*, *EGFR*, *RET* and *ROS1* (ref.^29^). Pathogenic alterations were annotated using three widely recognized databases: ClinVar^30^ (via Varsome), Cancer Hotspot^31^ and OncoKB^32^. Given minor inconsistencies among databases, an alteration was classified as pathogenic only if at least two of the three sources agreed. For model construction, we encoded individual binary features for *KRAS*, *TP53* and *STK11*, where 1 indicated a detected mutation, 0 indicated a wild-type allele and NA indicated missing testing information. Additionally, we created a composite ‘driver’ feature to capture actionable alterations relevant at the first line for a target therapy and associated with uncertain or limited evidence of benefit from IO and generally negative outcome (*EGFR*, *ALK*, ROS1 and RET). These features were coded as 1 if any of these were altered upon testing, 0 if at least one was tested and not altered and NA if no testing data were available. This approach helped reduce missingness, given the common practice of testing these genes together in clinical workflows, and aimed to preserve signal strength for modeling of IO response and survival.

### Development of the AI tools

We evaluated two main AI approaches for treatment outcome prediction: MLEF and DLIF. The data preprocessing and analysis pipelines were identical for both methods.

As part of the CB used for model training, we included a curated set of clinical and biological features selected by two expert lung oncologists (M.G. and A.P.) for the main analysis reported in this paper. These included patient characteristics (sex, ECOG PS and smoking status), molecular features (PD-L1 expression), metastatic sites (bone, liver and brain) and laboratory values (NLR and LDH). Details on feature encoding are provided in Supplementary Information 1, Table 2. In addition, we have run the data-driven approach by selecting CB using least absolute shrinkage and selection operator (LASSO) starting from 227 features, because there was no significant difference in results (Supplementary Information 7, Table 1); here we report results obtained by using nine selected features as a starting point. Missing values were handled using multivariate imputation via the IterativeImputer (1.6.1). We used CV with each fold corresponding to a different center (that is, leave-one-center-out (LOCO)), resulting in a total of five CV folds.

We performed the main analysis on the combined C2 and C3 to increase the cohort size. Additionally, we performed several subgroup analyses to evaluate performance across clinically homogeneous subpopulations. These included models trained on C2, IO-only, IO/CHT, PD-L1 ≥ 50%, PD-L1 < 50%, PD-L1 1–49%, PD-L1 < 1%, P53, KRAS, STK11, squamous histology, adenocarcinoma histology and site-specific cohorts; summary of all analyses is in Supplementary Information 2, Table 1. For benchmarking, we compared model performance against individual clinical biomarkers, including PD-L1 expression, NLR, LDH and ECOG PS.

#### ML CB-only and MLEF

Before training the models, we performed feature selection using LASSO regression for FMRAD due to its high embedding dimensionality (more than 4,000 in length). For all other modalities, we started from the initial number of features (as shown in Fig. 1a); we concatenated them across all modalities; and we applied LASSO to select the most relevant predictors.

We implemented and evaluated two ML classifiers: (1) logistic regression and (2) random forest. We selected the optimal hyperparameters by maximizing the AUC during model tuning via Bayesian optimization. The best MLEF classifier was selected using CV AUC. All models were trained exclusively on the training cohort, using LOCO, and then applied directly to TEST and to the EXVAL cohort without threshold adjustment: a fixed decision threshold of 0.5 was used. In addition, to assess potential positive selection bias in the multimodal population, we conducted dedicated sensitivity analyses, which did not demonstrate evidence of systematic selection bias (Supplementary Information 2, Tables 2 and 3).

All performance results include 95% CI, and comparisons across models were conducted using DeLong’s test for AUC differences, with *P* values calculated using a two-sided *z*-test: NS (not significant), *P ≤ 0.05, **P ≤ 0.01, ***P ≤ 0.001, ****P ≤ 0.0001. To evaluate model performance against single biomarkers, we focused on a subset of TEST for which biomarker measurements were available.

For the survival analysis, we implemented a Cox proportional hazards model as baseline, using the ‘lifelines’ package in Python^33^. Additionally, we evaluated two ML-based survival models (random survival forest (RSF) and gradient boosting survival (GBS)) implemented with scikit-survival^34^. Because no differences between models were noted, all the results for survival analysis refer to the Cox model.

All performance results include 95% CI, and C-index differences were tested using bootstrapping (*n* = 1,000) with two-sided *P* values derived from the bootstrap distribution. Given the exploratory, retrospective nature of this study and the large number of subgroup analyses and pairwise comparisons performed, all such analyses are considered hypothesis generating. No adjustment for multiple comparisons was applied; all *P* values are nominal and should be interpreted in the context of multiple testing. Results from the independent TEST and EXVAL cohorts are considered the primary evidence of model generalizability.

Because the MLEF pipeline cannot handle patients with missing modalities, all multimodal models exclude these patients. For transparency, each MLEF multimodal model was reported along with the number of patients included in the analysis as well as the best model for that analysis, and it was compared to the unimodal CB-only matched model using the same patient subset.

#### DL CB-only and DLIF

Our second approach, DLIF, was designed to address the missing modalities across patient cohorts. We implemented a supervised intermediate fusion pipeline for both classification and survival prediction tasks, integrating CB, DP, RAD and genomics using an attention-based multiple instance learning (MIL) framework^35^. In this framework, each patient is represented as a bag of vectors, allowing a variable number of instances per patient. This design is particularly well suited to complex oncological datasets, such as the I^3^LUNG retrospective cohort (Fig. 1b–d), where full multimodal coverage was available only for a subset of patients. Modality-specific encoders projected each modality into a shared embedding space, and a crossmodal reconstruction loss was incorporated during training to promote consistent and interpretable latent representations across modalities. Both loss functions combine a primary task-specific loss with a reconstruction loss component:

$${L}_{\mathrm{total}}={L}_{\mathrm{primary}}+\lambda {L}_{\mathrm{reconstruction}},$$

where *λ* is the reconstruction weight parameter. The reconstruction loss encourages the model to learn meaningful crossmodal representations by requiring it to reconstruct missing modalities from available ones. For a batch with *N* samples and *M* modalities, let ${x}_{m}^{(i)}$ be the input feature for sample *i* and modality *m*, ${\hat{x}}_{m,j}^{(i)}$ be the reconstruction of modality *j* from modality *m* for sample *i* and ${M}^{(i)}\in {\left\{0,1\right\}}^{M}$ be the modality mask indicating available modalities for sample *i*, and the reconstruction loss is computed as:

$${L}_{\mathrm{reconstruction}}=\frac{1}{M}\sum _{j=1}^{M}\frac{{\sum }_{i=1}^{N}{\sum }_{m=1}^{M}{M}_{m}^{(i)} {M}_{\!j}^{(i)} \mathrm{MSE}({\hat{x}}_{m,j}^{(i)},{x}_{\!j}^{(i)})}{{\sum }_{i=1}^{N}{\sum }_{m=1}^{M}{M}_{m}^{(i)} {M}_{\!j}^{(i)}},$$

where the double summation ensures that only valid reconstructions (where both source and target modalities are available) contribute to the loss; the normalization by the count of valid reconstructions prevents bias when modalities are missing; and mean squared error (MSE) is computed as $\mathrm{MSE}(\hat{x},x)=\frac{1}{d}{\sum }_{k=1}^{d}{({\hat{x}}_{k}-{x}_{k})}^{2}$ where *d* is the feature dimension. For multimodal classification tasks, the primary loss is cross-entropy:

$${L}_{\mathrm{classification}}=-\frac{1}{N}\mathop{\sum }\limits_{i=1}^{N}\mathop{\sum }\limits_{c=1}^{C}{y}_{c}^{\left(i\right)}\log \left(\sigma {\left({z}^{\left(i\right)}\right)}_{c}\right),$$

where *C* is the number of classes; ${y}_{c}^{(i)}$ is the one-hot encoded ground truth for sample *i* and class *c*; and $\sigma \left({z}^{\left(i\right)}\right)$ is the softmax of the model’s logits for sample *i*. Optional class weights $w\in {R}^{C}$ can be applied:

$${L}_{\mathrm{classification}}=-\frac{1}{N}\mathop{\sum }\limits_{i=1}^{N}\mathop{\sum }\limits_{c=1}^{C}{w}_{c}{y}_{c}^{\left(i\right)}\log \left(\sigma {\left({z}^{\left(i\right)}\right)}_{c}\right).$$

For survival analysis, the primary loss uses the Cox proportional hazards model:

$${L}_{\mathrm{survival}}=\frac{1}{{\sum }_{i=1}^{N}{\delta }_{i}}\sum _{i=1}^{N}{\delta }_{i}[{h}_{i}-\mathrm{log}(\sum _{j\in {R}_{i}}\exp ({h}_{\!j}))],$$

where *h*_*i*_ is the log-hazard prediction for sample *i*; *δ*_*i*_ is the event indicator (1 if event occurred, 0 if censored); and ${R}_{i}=\left\{j:{T}_{\!j}\ge {T}_{i}\right\}$ is the risk set (samples with survival time ≥*T*_*i*_). Samples are sorted by descending survival time for computational efficiency. To prevent overflow in the exponential operations, the log-sum-exp trick is used:

$$\log \left(\mathop{\sum }\limits_{j\in {R}_{i}}\exp ({h}_{\!j})\right)=\gamma +\log \left(\mathop{\sum }\limits_{j\in {R}_{i}}\exp ({h}_{\!j}-\gamma )\right),$$

where $$\gamma ={\max }_{j\in {R}_{i}}{h}_{\!j}.$$

This design constraint promotes information alignment across modalities and effective fusion through the MIL attention mechanism.

### Fairness evaluation

To assess fairness, we conducted a post hoc auditing focusing on potential biases across subgroups in TEST and EXVAL. We used center and sex as protected attributes for TEST and race and sex for EXVAL.

To quantify fairness, for classification models, we used equalized odds, which requires that both TPR (also known as sensitivity) and FPR are consistent across subgroups. TPR and FPR were calculated for the best-performing CB-only MLEF model.

$$\mathrm{TPR}=\frac{\mathrm{TP}}{\mathrm{TP}+\mathrm{FN}};\mathrm{FPR}=\frac{\mathrm{FP}}{\mathrm{FP}+\mathrm{TN}}.$$

For survival analysis models, we applied parity in C-index to verify whether model discrimination remained consistent across centers.

Fairness was evaluated using statistical tests to compare model performance across subgroups. For the classification tasks, we performed a permutation test with 1,000 iterations; for the survival task, we used bootstrapping with *n* = 1,000. In both cases, *P* values were calculated using a two-sided test for comparison between the two groups.

Per-center calibration analysis was conducted on the ML CB-only (logistic regression C23) model, selected as the case showing the largest statistically significant difference in TPR (*P* < 0.05). For each center, intercept recalibration was performed by fitting a logistic regression on the LOCO held-out set of that center, adjusting only the intercept while keeping all other model parameters fixed; this choice preserves the discriminative structure learned on the full cohort while correcting for center-specific shifts in the predicted probability scale. The recalibrated model was then evaluated on the corresponding center’s test subset. Classification metrics (TPR, FPR, sensitivity and specificity) were computed before and after recalibration for each of the five centers (GHD, INT, MH, SZMC and VHIO), and results are reported in Supplementary 5, Section 1, Table 2.

### Explainability

Explainability analysis was conducted using SHAP^36^. For each clinical endpoint, selecting the best-performing model, we generated summary SHAP plots trained on the TRAIN and applied both on the TRAIN and TEST. In addition, we produced individual waterfall plots for each patient in the TEST. These graphs are included in either the main text (Fig. 4c) or Extended Data Fig. 5, and these types of graphs were also used for the clinical usability study.

In the global SHAP summary plots, each point represents one patient, and the position on the *x* axis reflects the direction and magnitude of that feature’s contribution to the model output (for example, on Fig. 4c(i), positive SHAP values indicate a contribution toward class 1 (OS ≥ 24 months), whereas negative SHAP values indicate a contribution toward class 0 (OS < 24 months)). Features are ordered by mean absolute SHAP value, representing their overall importance across the cohort. In the individual waterfall plots (local explanations; Fig. 4c(iii)–(vi)), SHAP values illustrate how each feature shifts the prediction from the baseline (average model output) to the final predicted probability for a specific patient, thereby highlighting the key drivers of that individual prediction.

To identify the most influential features across outcomes, we created a summary graph (Fig. 4c(ii)) by calculating mean-ranked feature importance based on SHAP global values across all outcomes—the higher the rank, the higher the feature importance across analysis.

### Clinical usability with ML CB-only model

The primary objective of the clinical usability study was to assess the clinical applicability of the I^3^LUNG tool. The clinical usability patient cohort consisted of 100 patients, of whom 35 had CT scans available, and 42 had H&E slides available. Patients were stratified into subgroups randomly.

The study involved 20 medical oncologists, consisting of 10 expert lung oncologists and 10 nonexperts in lung cancer (including resident doctors and general oncologists) from the five centers (INT, VHIO, GHD, MH and UOC) from the consortium. Each patient was evaluated by one lung expert and one nonexpert, with each physician evaluating a total of 10 patients.

The study design consisted of two evaluation phases (Fig. 5). In phase 1, medical oncologists had access to important clinical data (all nine CB features plus other medical relevant characteristics; Supplementary Information 1, Table 1), segmented CT scan slice (when available) and DP slide. In phase 2, they had access to the available data and models output with global and local SHAP explanations. Supporting material provided to medical oncologists who were participating in the study is available in Supplementary Videos 1 and 2, including the tutorials on how to read the model’s explanation (tutorials for global and local explainability with SHAP). Patient evaluations were conducted based on two key outcomes: prediction of treatment response (DCR) and of survival (OS).

For treatment response, physicians categorized each patient as either a responder (CR, PR or SD) or a nonresponder (PD), following DCR RECIST^37^. For survival, physicians assigned each patient to one of five predefined OS time intervals: <6, 6–12, 12–18, 18–24 and ≥24 months, and the tool generated an individual OS probability curve for each patient.

To evaluate the impact of XAI and physician expertise on prediction performance, we performed a series of univariable logistic regression analyses. For DCR, two models were run, and, in both cases, the outcome was binary, indicating prediction success (1: correct prediction; 0: incorrect). In the first analysis, to assess the effect of XAI, we used phase as the covariate (0: no XAI, phase 1; 1: with XAI, phase 2); for the second analysis, to assess the effect of physician expertise, we used expertise level as the covariate (0: non-lung-expert oncologist; 1: lung expert oncologist). For OS prediction, success was defined as whether the predicted OS range overlapped with the true OS ± 25% interval (1: overlap; 0: no overlap). The ±25% margin was selected to apply a stricter and more conservative definition of prediction accuracy, ensuring consistency between model outputs and clinicians’ broad survival categories while avoiding artificial inflation of predictive performance. Similarly, two univariable logistic regression models were applied: first, to test the effect of XAI use, where phase was used as the covariate; second, to assess the role of expertise, the covariate was (0: non-lung-expert oncologist; 1: lung expert oncologist). All models were run for the full physician group and separately for experts and nonexperts. All the results are presented as odds ratio and AUC with their 95% CI and are shown in Supplementary Information 6.

The degree of agreement between the expert and nonexpert physicians’ DCR and OS evaluations was assessed by computing the Cohen’s *κ* and the weighted Cohen’s *κ*, respectively. Based on the observed *κ*, the degree of concordance was considered as follows:

- 0: no agreement

- (0.01–0.20): slight agreement

- (0.21–0.40): fair agreement

- (0.41–0.60): moderate agreement

- (0.61–0.80): substantial agreement

- (0.81–0.99): near-perfect agreement

- 1: perfect agreement

McNemar’s test for paired data was employed to assess any differences between experts and nonexperts in the discordant predictions in phase 1 and phase 2.

Furthermore, sensitivity, specificity, accuracy, precision and F1 were computed for the DCR prediction according to phase and physicians’ expertise. The 95% CIs of these metrics were calculated by using the Clopper–Pearson exact binomial method. McNemar’s test for paired data was employed to assess any statistically significant differences. The F1 score was derived according to phase and physicians’ expertise for the DCR prediction. Its 95% CI was computed by using bootstrap methods with 1,000 replications.

Due to the time-to-event nature of the OS, conventional classification metrics based on true positives and true negatives could not be applied. Therefore, we defined a patient-level correctness score based on the agreement between the true OS and the physician’s predicted OS ranges as follows:

- 0 points were assigned in case of different and nonconsecutive ranges for the true OS and the predicted OS by the physician.

- 1 point was assigned in case of different, but consecutive, ranges for the true OS and the predicted OS by the physician.

- 2 points were assigned in case of identical ranges for the true OS and the predicted OS by the physician.

The Wilcoxon signed-rank test for paired data was employed to assess any statistically significant differences between the assigned scores’ distributions. A total correctness score was computed by summing all scores according to phase and physicians’ expertise.

Finally, the confusion matrix after using XAI^38^ was derived for all physicians, for experts only and for nonexperts only (Fig. 5c and Supplementary Information 6, Tables 38–40). When a medical oncologist decides with the assistance of the XAI tool, four errors can occur: (1) false confirmation error (FCoE), (2) false conflict error (FCE), (3) true conflict error (TCE) and (4) true confirmation error (TCoE). Furthermore, in case of correct prediction, there are four possible scenarios: (1) correct true confirmation case (CTCo), (2) correct true conflict case (CTC), (3) correct false confirmation case (CFCo) and (4) correct false conflict case (CFC). All are summarized and explained in Supplementary Information 6, Table 1.

### Ethics committee details

IO cohorts obtained approval from all of the centers within the I^3^LUNG study protocol:

1. Comitato etico Fondazione IRCSS Istituto Nazionale dei Tumori (INT), ethics committee code 147/22, approved on 25 July 2022
2. Ethik-Kommission Universitäumalt zu Lübeck (GHD), ethics committee code 2022-439, approved on 17 October 2022 (clinical study)
3. Scientific Committee Metropolitan Hospital (MH), ethics committee code 30.08.2022, approved on 30 August 2022
4. Helsinki Committee Share Zedek Medical Center (SZMC), ethics committee code 0240-22-SZMC, approved on 20 September 2022
5. Comité de Ética de Investigación con medicamentos y Comisión de Proyectos de Investigación del Hospital Universitari Vall D’Hebron, approved on 17 January 2023
6. Institutional Review Board University of Chicago (UOC), ethics committee code IRB22-1305, approved on 8 September 2022

### Non-IO cohorts

C-EGFR-INT: These data were collected within the APOLLO 11 study and approved by the ethics committee of INT (reference no. INT 128/22) on 29 June 2022.

C-EGFR-UOC: Institutional Review Board University of Chicago (UOC), ethics committee code IRB24-2188, approved on 23 December 2024

C-StageIII-CHT: For this cohort collected in INT, the data protection impact assessment was developed and is published at the INT website https://dpm.istitutotumori.mi.it/public/documents/ (called Informativa-I^3^LUNG Monocentrico), in compliance with applicable personal data protection laws, including Regulation (EU) 2016/679 (General Data Protection Regulation) and relevant national legislation. A distinct and appropriate legal basis was identified for each category of personal data processed, tailored to the nature and purpose of each specific processing activity. Suitable technical and organizational measures were put in place to ensure an adequate level of data security, in line with the principles of data minimization, purpose limitation and access control, ensuring that only authorized individuals were granted access to personal information. The research was carried out in accordance with applicable ethical standards and under the supervision of the relevant institutional bodies. Throughout the entire research process, the accountability principle was upheld, meaning that the data controller was not only committed to complying with data protection requirements but was also in a position to demonstrate such compliance at any time.

Generative AI tools, specifically GPT-5.3, were used for English language editing to improve the clarity of this paper. The AI-assisted language editing was performed under human oversight, and the final paper was thoroughly reviewed and edited by the authors to ensure accuracy and integrity. All intellectual content, literature analysis and scientific conclusions were conducted by the authors.

### Reporting summary

Further information on research design is available in the Nature Portfolio Reporting Summary linked to this article.

## Online content

Any methods, additional references, Nature Portfolio reporting summaries, source data, extended data, supplementary information, acknowledgements, peer review information; details of author contributions and competing interests; and statements of data and code availability are available at https://doi.org/10.1038/s41591-026-04488-2.

## Supplementary information

Supplementary Information Supplementary Information 1: Tables 1–36. Supplementary Information 2: Figs. 1 and 2, Tables 1–5, and Figs. 3–6. Supplementary Information 4: Figs. 1–24. Supplementary Information 5: Table 1, Fig. 1, Tables 2 and 3, Figs. 2 and 3, Table 4, Fig. 4, Table 5, and Fig. 5. Supplementary Information 6: Tables 1–40. Supplementary Information 7: Figs. 1 and 2, Tables 1 and 2, and Robustness Analysis. Plus the Observational Study Protocol.

Reporting Summary

Peer Review File

Supplementary Table 1 Summary Excel file with results for all MLEF analyses.

Supplementary Video 1 Supporting material for clinical usability study global SHAP tutorial provided to physicians.

Supplementary Video 2 Supporting material for clinical usability local global SHAP tutorial provided to physicians.

## Data Availability

Deidentified data supporting the findings of this study are publicly available on Zenodo (https://zenodo.org/records/17535424) and will remain accessible for 20 years. The shared dataset includes CB variables, genomics data and extracted features derived from medical imaging analyses, including radiomics and foundation-model-based features from CT scans and DP images (GigaPath and TITAN). These are necessary data to replicate the study. During the peer review process, reviewers were provided with access to these data necessary to reproduce the study analyses. The I^3^LUNG project is an ongoing EU-funded initiative, with additional analyses and related papers planned through June 2027. Therefore, the complete raw multimodal dataset, including original imaging files, cannot currently be fully released. In accordance with the consortium’s data-sharing policy and applicable European Commission regulations governing data access, sharing and open science within EU-funded projects, the full raw datasets from all modalities will become available 5 years after the official completion of the project.

## Code availability

Data analyses were performed using a combination of commercial and open-source software tools, including SAS software version 9.4 (SAS Institute), R (version 4.2.1), Python (version 3.11.15 for MLEF and version 3.9.25 for DLIF) and 3D Slicer (version 5.2.2). Custom scripts developed for data preprocessing and model development are publicly available on GitHub (https://github.com/AI-ON-Laboratory/I3LUNG_PDSS).
