# Supplementary material for: Clinical usability of an explainable AI decision support tool and evaluation of multimodal models in NSCLC
Source: Nat Med. 2026 Sep 13;32(9):3235–47. doi: 10.1038/s41591-026-04488-2 (PMC13577906; doi:10.1038/s41591-026-04488-2)
Supplement: Supplementary file 1 [file 41591_2026_4488_MOESM1_ESM.pdf]

nature medicine

Supplementary information https://doi.org/10.1038/s41591-026-04488-2

Clinical usability of an explainable AI decision support tool and evaluation of multimodal models in NSCLC

In the format provided by the authors and unedited

Supplementary Information 1

This supplementary document provides extended clinical details and analyses that complement the main study. It includes a comprehensive list of hypothesis-driven and data-driven features, baseline characteristics of the patient cohorts across multiple centers, alongside statistical analysis comparisons of the Training, Testing, and External Validation sets. Key sections explore feature distributions stratified by specific predictive endpoints, including 6-month Overall Survival (OS6), 24-month Overall Survival (OS24), and Disease Control Rate (DCR). Furthermore, it assesses data robustness by systematically comparing patient populations with complete versus incomplete multimodal records. Finally, it provides quality control and metadata evaluations for CT acquisition parameters and H&E digital slides.

Table 1. List of 227 features used in the data-driven model, and descriptive features given to the clinicians during the clinical usability study. Some of the features have been one-hot encoded, leading to 322 features in total.

Features Name	Given to clinicians during the clinical usability study	Data-driven features
AGE_AT_DIAGNOSIS	YES	YES
SEX	YES	YES
NSCLC_HISTOLOGY_ADENOCARCINOMA	YES	YES
NSCLC_HISTOLOGY_OTHER	YES	YES
NSCLC_HISTOLOGY_SQUAMOUS	YES	YES
STAGE_DIAGNOSIS_T	YES	YES
STAGE_DIAGNOSIS_N	YES	YES
STAGE_DIAGNOSIS_M	YES	YES
DIAG_STAGE	YES	YES
MENOPAUSE	NO	YES
PREGNANCY	NO	YES

CONCOMITANT_DISEASE	NO	YES
CONCOMITANT_DISEASES_NUM	NO	YES
DISEASE_CARDIOVASCULAR_DISORDERS	YES	YES
DISEASE_RENAL_DISORDERS	YES	YES
DISEASE_OTHER_NEOPLASMS	YES	YES
DISEASE_PULMONARY_DISEASE	YES	YES
DISEASE_AUTOIMMUNE_DISORDERS	YES	YES
DISEASE_HEPATIC_DISORDERS	YES	YES
DISEASE_INFECTIONS	YES	YES
DISEASE_GI_DISORDERS	YES	YES
DISEASE_ENDOCRINE_DISORDERS	YES	YES
DISEASE_DIABETES	YES	YES
DISEASE_NEUROLOGICAL_DISORDERS	YES	YES
DISEASE_DISLIPIDEMIA	YES	YES
ALLERGY	NO	YES
OTHER_CANCER_TYPE_BREAST	NO	YES
OTHER_CANCER_TYPE_GASTRO-INTESTINAL	NO	YES
OTHER_CANCER_TYPE_GYNECOLOGICAL	NO	YES

OTHER_CANCER_TYPE_HEAD & NECK	NO	YES
OTHER_CANCER_TYPE_HEMATOLOGICAL	NO	YES
OTHER_CANCER_TYPE_OTHER_NON SPECIFIED	NO	YES
OTHER_CANCER_TYPE_SARCOMA AND SOFT TISSUE	NO	YES
OTHER_CANCER_TYPE_SKIN_MELANOMA	NO	YES
OTHER_CANCER_TYPE_THORACIC NEOPLASMS	NO	YES
OTHER_CANCER_TYPE_UROGENITAL	NO	YES
EVER_SMOKED	NO	YES
SMOKING_CURRENT	YES	YES
SMOKING_FORMER	YES	YES
SMOKING_NEVER	YES	YES
SMOKING_PACKYEAR_CUTOFF_10	NO	YES
SMOKING_PACKSYEAR	YES	YES
E_CIGARETTES	NO	YES
PASSIVE_SMOKING	NO	YES
ASBESTOS	NO	YES
ALCOHOL	NO	YES
DRUG	NO	YES

FAMILY_CANCER	NO	YES
PROFESSIONAL_STATUS	NO	YES
EDUCATION	NO	YES
RESIDENCE_CITY	NO	YES
RESIDENCE_VILLAGE	NO	YES
RACE_AMERICAN INDIAN OR ALASKA NATIVE	YES	YES
RACE_ASIAN	YES	YES
RACE_BLACK OR AFRICAN AMERICAN	YES	YES
RACE_NATIVE HAWAIIAN OR OTHER PACIFIC ISLANDER	YES	YES
RACE_WHITE	YES	YES
ETHNICITY_AFRICAN	YES	YES
ETHNICITY_AMERICAN	YES	YES
ETHNICITY_ASIATIC	YES	YES
ETHNICITY_CAUCASIAN	YES	YES
ALLERGIES_NUM	NO	YES
ALLERGY_ANTIBIOTICS	NO	YES
ALLERGY_CONTRAST_MEDIUM	NO	YES
ALLERGY_ANTICANCER_DRUGS	NO	YES

ALLERGY_ASPIRIN_FANS	NO	YES
ONCOLOGICAL_COMORBIDITY	NO	YES
NEOAD_TR_1	NO	YES
NEO_IMMUNO	NO	YES
NEO_TARGET	NO	YES
NEO_PLATINUM_BASED_CHEMO	NO	YES
SURGERY_1	YES	YES
ADJ_CHEMO	YES	YES
ADJ_TR	YES	YES
ADJ_IMMUNO	YES	YES
ADJ_TARGET	YES	YES
ADJ_PLATINUM_BASED_CHEMO	NO	YES
ADJ_RT	YES	YES
CONCO_SEQ_CONCOMITANT	NO	YES
CONCO_SEQ_SEQUENTIAL	NO	YES
BMI_BASAL	YES	YES
EGFR	YES	YES
KRAS	YES	YES
KRAS_MUT	NO	YES

BRAF	YES	YES
MET_EX14SKIP	YES	YES
MET_AMP	YES	YES
ALK	YES	YES
ROS1	YES	YES
RET	YES	YES
NTRK	YES	YES
NRG1	NO	YES
NRG1_ALT	NO	YES
P53	YES	YES
KI67	NO	YES
BIO_GRADING	NO	YES
TTF1	NO	YES
P40	NO	YES
CK7	NO	YES
CK20	NO	YES
SYNAPTOPHYSIN	NO	YES
CROMOGRANIN_A	NO	YES
NAPSIN	NO	YES

PDL1 CATEGORY	YES	YES
PDL1 CONTINUOUS	NO	YES
HER2 INSERTION	NO	YES
HER2 AMPLIFICATION	NO	YES
HER2 MUTATION	NO	YES
HER2 GENERAL	YES	YES
IHC_STK11	NO	YES
STK11 MUT	NO	YES
STK11 GENERAL	YES	YES
FGFR	NO	YES
DRIVER_MUTATION	NO	YES
RTK_RAS_PATHWAY	NO	YES
DNA_DAMAGE_PATHWAYS	NO	YES
CELL_CYCLE_ALTERATION	NO	YES
HRD_ALTERATION	NO	YES
EPIGENOMIC_REG	NO	YES
WNT_PATH	NO	YES
NF_ALTERATION	NO	YES
PIK3CA PATHWAY	NO	YES

PROG_DATE_IMP_CRIT	NO	YES
AGE_AT_IO_START	NO	YES
AGE_METASTATIC	NO	YES
METS_DIAGNOSIS	NO	YES
CRT	NO	YES
RADICAL_IMMUNO_TREATMENT	NO	YES
RADICAL_TARGET_TREATMENT	NO	YES
RADICAL_CHEMO_TREATMENT	NO	YES
RADICAL_PLATIN	NO	YES
RADICAL_RT_BEFORE_IMMUNO	NO	YES
RADICAL_RT_DOSE	NO	YES
METASTATIC_STAGE	NO	YES
METASTATIC_SITE_ADRENAL	NO	YES
METASTATIC_SITE_BONE	NO	YES
METASTATIC_SITE_BONE MARROW	NO	YES
METASTATIC_SITE_LEPTOMENINGEAL	NO	YES
METASTATIC_SITE_LIVER	NO	YES
METASTATIC_SITE_LUNG	NO	YES

METASTATIC_SITE_LYMPH NODES	NO	YES
METASTATIC_SITE_OTHER	NO	YES
METASTATIC_SITE_PARENCHYMAL BRAIN	NO	YES
METASTATIC_SITE_PERICARDIAL EFFUSION	NO	YES
METASTATIC_SITE_PLEURA	NO	YES
METASTATIC_SITE_PLEURAL EFFUSION	NO	YES
METASTATIC_SITE_SKIN	NO	YES
METASTATIC_SITE_SOFT TISSUE	NO	YES
CHEMO_CARBOBASED	NO	YES
CHEMO_CISBASED	NO	YES
CHEMO_OTHER	NO	YES
T_IO_START	YES	YES
N_IO_START	YES	YES
M_IO_START	YES	YES
STAGE_IO_START	YES	YES
SITE_METS_IO_START_ADRENAL	YES	YES
SITE_METS_IO_START_BONE	YES	YES
SITE_METS_IO_START_BONE MARROW	YES	YES

SITE_METS_IO_START_LEPTOMENINGEAL	YES	YES
SITE_METS_IO_START_LIVER	YES	YES
SITE_METS_IO_START_LUNG	YES	YES
SITE_METS_IO_START_LYMPH NODES	YES	YES
SITE_METS_IO_START_OTHER	YES	YES
SITE_METS_IO_START_PARENCHYMAL BRAIN	YES	YES
SITE_METS_IO_START_PERICARDIAL EFFUSION	YES	YES
SITE_METS_IO_START_PLEURA	YES	YES
SITE_METS_IO_START_PLEURAL EFFUSION	YES	YES
SITE_METS_IO_START_SKIN	YES	YES
SITE_METS_IO_START_SOFT TISSUE	YES	YES
CONCO_TP_1	NO	YES
CONCO_TP_ANTIBIOTICS_ANTIVIRALS_ANTIFUNGALS	YES	YES
CONCO_TP_STEROIDS_IMMUNODEPRESSANT	YES	YES
CONCO_TP_OPPIOD	YES	YES
CONCO_TP_PARACETAMOL	YES	YES
CONCO_TP_ASPIRIN_FANS	YES	YES
CONCO_TP_PPIS	YES	YES

CONCO_TP_CALCIUM_BONE_METABOLISM	YES	YES
CONCO_TP_ANTI_EMETICS	YES	YES
CONCO_TP_ANTIDEPRESSANTS_BZN_OTHER_SNC	YES	YES
CONCO_TP_ANTIHYPERTENSIVE_DIURETICS_IPB	YES	YES
CONCO_TP_STATINS_HYPOLIDEMICS	YES	YES
CONCO_TP_ANTICOAGULANTS	YES	YES
CONCO_TP_INSULIN_METFORMIN_ANTIDIABETICS	YES	YES
CONCO_TP_ANTIHISTAMINES.	YES	YES
CONCO_TP_LAXATIVES	YES	YES
CONCO_TP_ANTIDIARRHEALS	YES	YES
CONCO_TP_ANTITUSSIVE	YES	YES
CONCO_TP_INHALED_X_BPCO_ASTHMA	YES	YES
CONCO_TP_CUTANEOUS	YES	YES
CONCO_TP_OCULAR_AGENTS	YES	YES
CONCO_TP_VITAMINES	YES	YES
CONCO_TP_OTHER_ANTICANCER_DRUGS	YES	YES
CONCO_TP_HORMONAL	YES	YES
CONCO_TP_THYROID	YES	YES

CONCO_TP_G-CSF_EPOS	YES	YES
CONCO_TP_POTASSIUM_MAGNESIUM	YES	YES
CONCO_TP_OTHER	YES	YES
STEROID_1	NO	YES
STEROID_NAME_1	NO	YES
IMMUNOSUP_1	NO	YES
IO_IOCT	YES	YES
IO_ALONE	YES	YES
HEIGHT_BASAL	NO	YES
WEIGHT_BASAL	NO	YES
ECOG	YES	YES
WEIGHT_LOSS	YES	YES
CF08_BASAL_SYMPTOMS_ALOPECIA/ HAIR LOSS	NO	YES
CF08_BASAL_SYMPTOMS_ANOREXIA	NO	YES
CF08_BASAL_SYMPTOMS_BONE PAIN	NO	YES
CF08_BASAL_SYMPTOMS_CHEST PAIN	NO	YES
CF08_BASAL_SYMPTOMS_CONSTIPATION	NO	YES
CF08_BASAL_SYMPTOMS_DIARRHEA	NO	YES

CF08_BASAL_SYMPTOMS_DYSGEUSIA/ TASTE ALTERATION	NO	YES
CF08_BASAL_SYMPTOMS_DYSPHAGIA	NO	YES
CF08_BASAL_SYMPTOMS_DYSPNOEA	NO	YES
CF08_BASAL_SYMPTOMS_FATIGUE	NO	YES
CF08_BASAL_SYMPTOMS_FEVER	NO	YES
CF08_BASAL_SYMPTOMS_HAEMOPTYSIS	NO	YES
CF08_BASAL_SYMPTOMS_INSOMNIA	NO	YES
CF08_BASAL_SYMPTOMS_NAUSEA	NO	YES
CF08_BASAL_SYMPTOMS_OTHER	NO	YES
CF08_BASAL_SYMPTOMS_PERIPHERAL NEUROPATHY	NO	YES
CF08_BASAL_SYMPTOMS_RASH/PRURITUS	NO	YES
CF08_BASAL_SYMPTOMS_SKIN CHANGES/ DRY SKIN/XERODERMIA	NO	YES
CF08_BASAL_SYMPTOMS_UNKNOWN/NOT AVAILABLE	NO	YES
CF08_BASAL_SYMPTOMS_VOMITING	NO	YES
HBV	YES	YES
HCV	NO	YES
HIV	NO	YES

TBC	NO	YES
OTHER_VIRUS	NO	YES
HB	NO	YES
HCT	NO	YES
PLT	NO	YES
LEUKOCYTES_BASAL	YES	YES
NEUTROPHYL_BASAL	YES	YES
NLR	YES	YES
MONOCYTE_BASAL	NO	YES
EOSINOPHIL_BASAL	NO	YES
LYMPHOCYTES_BASAL	YES	YES
BASOPHIL_BASAL	NO	YES
LDH	YES	YES
CREATININ	YES	YES
IRON	NO	YES
TRANFERRIN	NO	YES
FERRITIN	NO	YES
COLESTEROL	NO	YES
HDL	NO	YES

LDL	NO	YES
TRIGLYCERIDES	NO	YES
AST	NO	YES
ALT	YES	YES
GGT	NO	YES
ALP	YES	YES
INR	NO	YES
PROTEIN_TOT	NO	YES
ALBUMIN	YES	YES
GLUCOSE	NO	YES
CRP	NO	YES
NA	NO	YES
K	NO	YES
CA	NO	YES
P	NO	YES
MG	NO	YES
AMYLASE	NO	YES
LIPASE	NO	YES
TSH	YES	YES

FT3	NO	YES
FT4	NO	YES
ACTH	NO	YES
CORTISOL_H8	NO	YES
URINE	NO	YES
CPK	NO	YES
TNT	NO	YES
TNI	NO	YES
BRAIN_LESION_NUMBER	YES	YES
LOCAL_TREATMENTS	NO	YES
RT_SITE_COHORT2_3_ADRENAL	NO	YES
RT_SITE_COHORT2_3_BONE	NO	YES
RT_SITE_COHORT2_3_LEPTOMENINGEAL	NO	YES
RT_SITE_COHORT2_3_LIVER	NO	YES
RT_SITE_COHORT2_3_LUNG	NO	YES
RT_SITE_COHORT2_3_LYMPH NODES	NO	YES
RT_SITE_COHORT2_3_OTHER	NO	YES
RT_SITE_COHORT2_3_PARENCHYMAL BRAIN	YES	YES
RT_SITE_COHORT2_3_PERICA	NO	YES

RDIAL EFFUSION		
RT_SITE_COHORT2_3_PLEURA	NO	YES
RT_SITE_COHORT2_3_SOFT TISSUE	NO	YES
RT_DOSE_COHORT2_3	YES	YES
RT_FRACTION_COHORT2_3	NO	YES
RT_BESTRESP_COHORT2_3	NO	YES
LINE_IO_COHORT3	NO	YES
FIRST_LINE	NO	YES
PREVIOUS_CHEMO_TP	NO	YES
PREVIOUS_IMMUNO_TP	NO	YES
PREVIOUS_PLATINUM_BASED_CHEMO	NO	YES
PREVIOUS_TARGET_TP	NO	YES
PREVIOUS_TAXANE_BASED_CHEMO	NO	YES
SITE_METS_IO_START_UNKNOWN/NOT AVAILABLE	NO	YES
BASAL_SYMPTOMS_ANOREXIA	NO	YES
BASAL_SYMPTOMS_BONE PAIN	NO	YES
BASAL_SYMPTOMS_CHEST PAIN	NO	YES
BASAL_SYMPTOMS_CONSTIPATION	NO	YES

BASAL_SYMPTOMS_DIARRHEA	NO	YES
BASAL_SYMPTOMS_DYSGEUSIA/ TASTE ALTERATION	NO	YES
BASAL_SYMPTOMS_DYSPHAGIA	NO	YES
BASAL_SYMPTOMS_DYSPNOEA	NO	YES
BASAL_SYMPTOMS_FATIGUE	NO	YES
BASAL_SYMPTOMS_FEVER	NO	YES
BASAL_SYMPTOMS_HAEMOPTYSIS	NO	YES
BASAL_SYMPTOMS_INSOMNIA	NO	YES
BASAL_SYMPTOMS_NAUSEA	NO	YES
BASAL_SYMPTOMS_OTHER	NO	YES
BASAL_SYMPTOMS_PERIPHERAL NEUROPATHY	NO	YES
BASAL_SYMPTOMS_RASH/PRURITUS	NO	YES
BASAL_SYMPTOMS_UNKNOWN/NOT AVAILABLE	NO	YES
BASAL_SYMPTOMS_VOMITING	NO	YES
RT_SITE_COHORT2_3_PLEURAL EFFUSION	NO	YES
RT_SITE_COHORT2_3_SKIN	NO	YES
DRIVER	NO	YES
LIPI	YES	YES

EPSILON	YES	YES
IMMUNOTHERAPY_AGENT	YES	NO
CHEMOTHERAPY_AGENT	YES	NO
DIAGNOSIS_YEAR	YES	NO
METASTATIC_DIAGNOSIS_YEAR	YES	NO
SURGERY_DATE	YES	NO
ADJUVANT_TREATMENT_START	YES	NO
ADJUVANT_TREATMENT_END	YES	NO
DURVALUMAB_IN_MAINTENANCE	YES	NO
CHEMORADIOTHERAPY	YES	NO
CHEMOTHERAPY_AGENT_IN_CTRT	YES	NO
BRAIN_RADIOTHERAPY_TYPE	YES	NO

Table 2 List of hypothesis-driven features

Name	Description	Encoding
SEX	Sex of the patient	0: female<br>1: male
ECOG	Ecog performance status	An integer between 0 and 5<br>0: best<br>5: worst
LDH	Lactate dehydrogenase	Numeric (U/L)
NLR	Neutrophils to lymphocytes ratio	Numeric
SMOKING_CURRENT	Current smoker	0: no<br>1: yes
SMOKING_FORMER	Former smoker	0: no<br>1: yes

SMOKING_NEVER	Never smoked	0: no<br>1: yes
PDL1	Category of PDL1	0: <1%<br>1: ≥1%, <50%<br>2: ≥50%
SITE_METS_IO_START_PARENCHYMAL BRAIN	Parenchymal brain metastases	0: no<br>1: yes
SITE_METS_IO_START_LIVER	Liver metastases	0: no<br>1: yes
SITE_METS_IO_START_BONE	Bone metastases	0: no<br>1: yes
RACE*	The self-reported race of the patient	White, Asian, Native Hawaiian or other pacific islander, Black or African American, American Indian or Alaska Native

Table 3. Patients characteristics across the centers (INT, GHD, MH, SZMC, UOC, VHIO) and overall, including p-value for comparison across centers. *: p-value of Kruskal-Wallis' Test for Medians **: p-value of Chi-Square Test ***: p-value of Fisher's Exact Test ****: p-value of Fisher's Exact Test based on 10000 Monte Carlo simulations. In bold are statistically significant p values.

Variable	INT (n=684)	GHD (n=430)	MH (n=231)	SZMC (n=273)	UOC (n=251)	VHIO (n=206)	p-value	Overall (n=2075)
Sex- n(%)							<0.0001**	
Female	436 (63.7)	262 (60.9)	176 (76.2)	176 (64.5)	148 (59.0)	138 (67.0)		1291 (62.2)
Male	248 (36.3)	168 (39.1)	55 (23.8)	97 (35.5)	103 (41.0)	68 (33.0)		784 (37.8)
Menopausal - n (%)							<0.0001****	
No	17 (6.9)	32 (19.3)	6 (11.1)	13 (18.1)	6 (5.4)	16 (23.5)		90 (12.5)
Yes	231 (93.1)	134 (80.7)	48 (88.9)	59 (81.9)	105 (94.6)	52 (76.5)		629 (87.5)
Missing	0	2	1	25	37	0		65
Previous Pregnancy - n (%)							0.0224****	
No	1 (6.3)	1 (12.5)	14 (41.2)	1 (20.0)	5 (17.9)	0 (0.0)		22 (21.8)
Yes	15 (93.8)	7 (87.5)	20 (58.8)	4 (80.0)	23 (82.1)	10 (100.0)		79 (78.2)
Missing	232	160	21	92	120	58		683
Age at Diagnosis (Years)							0.0021*	
Mean (SD)	66.3 (10.0)	65.9 (9.5)	66.4 (9.4)	66.9 (9.3)	67.0 (9.8)	63.2 (11.5)		66.1 (9.9)

Median (Q1 - Q3)	67.4 (60.2-73.4)	66.1 (59.1-73.5)	67.8 (60.9-73.2)	67.8 (60.5-72.8)	67.3 (60.0-73.9)	62.9 (55.3-71.9)		66.9 (59.6-73.3)
Min - Max	25.7 - 92.7	33.5 - 92.5	35.4 - 87.4	34.3 - 93.4	34.8 - 91.7	34.4 - 89.9		25.7 - 93.4
Missing	0	0	1	7	1	0		9
BMI at Baseline							0.0009*	
Mean (SD)	24.2 (4.1)	24.7 (4.7)	25.7 (4.7)	24.5 (5.0)	24.8 (5.4)	25.5 (4.6)		24.7 (4.7)
Median (Q1 - Q3)	23.9 (21.4-26.8)	24.4 (21.6-27.2)	25.1 (22.9-28.1)	23.9 (21.3-27.0)	24.7 (20.5-28.7)	25.5 (21.8-28.8)		24.3 (21.5-27.5)
Min - Max	13.5 - 46.2	14.4 - 45.8	17.5 - 59.0	3.7 - 37.5	12.6 - 42.2	14.7 - 38.8		3.7 - 59.0
Missing	75	30	2	35	0	104		246
Professional Status - n (%)							<0.0001**	
No	3 (6.4)	10 (21.3)	93 (78.8)	4 (8.5)	69 (60.5)	1 (6.7)		180 (46.4)
Yes	44 (93.6)	37 (78.7)	25 (21.2)	43 (91.5)	45 (39.5)	14 (93.3)		208 (53.6)
Missing	637	383	113	226	137	191		1687
Level of Education - n (%)							0.0254****	
Degree	6 (85.7)	3 (100.0)	21 (52.5)	16 (94.1)	28 (66.7)	0 (0.0)		74 (67.9)
Primary school	0 (0.0)	0 (0.0)	1 (2.5)	0 (0.0)	0 (0.0)	0 (0.0)		1 (0.9)
Secondary school	1 (14.3)	0 (0.0)	18 (45.0)	1 (5.9)	14 (33.3)	0 (0.0)		34 (31.2)
Missing	677	427	191	256	209	206		1966
Residence - n (%)							<0.0001****	
City	339 (99.7)	0 (0.0)	177 (86.3)	55 (98.2)	78 (32.5)	11 (91.7)		660 (77.4)
Village	1 (0.3)	0 (0.0)	28 (13.7)	1 (1.8)	162 (67.5)	1 (8.3)		193 (22.6)
Missing	344	430	26	217	11	194		1222
Smoking Status - n (%)							<0.0001**	
Never	50 (7.8)	19 (5.6)	14 (6.3)	30 (11.8)	23 (9.2)	23 (11.2)		159 (8.3)
Former	407 (63.8)	144 (42.4)	95 (42.8)	114 (44.7)	189 (75.3)	127 (61.7)		1076 (56.3)
Current	181 (28.4)	177 (52.1)	113 (50.9)	111 (43.5)	39 (15.5)	56 (27.2)		677 (35.4)
Missing	46	90	9	18	2	0		163
Smoking Packs per Year (Among Current Smokers)							<0.0001*	

Mean (SD)	45.8 (61.5)	46.2 (20.0)	67.4 (34.0)	68.7 (36.2)	74.1 (101.9)	44.1 (20.4)		55.1 (42.0)
Median (Q1 - Q3)	37.3 (18.0-50.0)	40.0 (35.0-59.0)	60.0 (40.0-100.0)	60.0 (40.0-100.0)	40.0 (30.0-60.0)	40.0 (30.0-50.0)		50.0 (35.0-65.0)
Min - Max	0.9 - 547.5	2.0 - 120.0	1.0 - 175.0	20.0 - 250.0	7.0 - 365.0	2.0 - 100.0		0.9 - 547.5
Missing	87	7	0	21	19	1		135
E-Cigarettes Use - n (%)							0.1631***	
No	344 (98.3)	14 (93.3)	136 (97.1)	52 (98.1)	210 (99.5)	1 (100.0)		757 (98.3)
Yes	6 (1.7)	1 (6.7)	4 (2.9)	1 (1.9)	1 (0.5)	0 (0.0)		13 (1.7)
Missing	334	415	91	220	40	205		1305
Exposure to Passive Smoking - n (%)							0.0025***	
No	318 (98.1)	1 (100.0)	131 (100.0)	23 (88.5)	161 (100.0)	0 (0.0)		634 (98.6)
Yes	6 (1.9)	0 (0.0)	0 (0.0)	3 (11.5)	0 (0.0)	0 (0.0)		9 (1.4)
Missing	360	429	100	247	90	206		1432
Level of Alcohol Consumption							<0.0001**	
Abuse	6 (17.1)	35 (53.8)	27 (12.6)	0 (0.0)	36 (15.2)	7 (70.0)		111 (18.5)
No	20 (57.1)	10 (15.4)	146 (68.2)	39 (97.5)	128 (54.0)	1 (10.0)		344 (57.2)
Social	9 (25.7)	20 (30.8)	41 (19.2)	1 (2.5)	73 (30.8)	2 (20.0)		146 (24.3)
Missing	649	365	17	233	14	196		1474
Drugs Consumption							<0.0001****	
No	13 (81.3)	16 (80.0)	136 (99.3)	33 (89.2)	200 (85.1)	0 (0.0)		398 (89.4)
Yes	3 (18.8)	4 (20.0)	1 (0.7)	4 (10.8)	35 (14.9)	0 (0.0)		47 (10.6)
Missing	668	410	94	236	16	206		1630
Allergies - n (%)							<0.0001**	
No	421 (71.5)	35 (66.0)	217 (93.9)	143 (65.0)	119 (48.4)	4 (40.0)		939 (69.6)
Yes	168 (28.5)	18 (34.0)	14 (6.1)	77 (35.0)	127 (51.6)	6 (60.0)		410 (30.4)
Missing	95	377	0	53	5	196		726
Number of Allergies (Among those with allergies)							0.0001*	
Mean (SD)	1.5 (1.0)	1.7 (0.9)	1.7 (1.4)	1.3 (0.9)	1.9 (1.4)	1.8 (1.3)		1.6 (1.2)

Median (Q1 - Q3)	1.0 (1.0-2.0)	1.5 (1.0-2.0)	1.0 (1.0-2.0)	1.0 (1.0-1.0)	1.0 (1.0-2.0)	1.0 (1.0-3.0)		1.0 (1.0-2.0)
Min - Max	0.0 - 7.0	1.0 - 4.0	1.0 - 6.0	0.0 - 6.0	1.0 - 7.0	1.0 - 4.0		0.0 - 7.0
Oncological Comorbidities - n (%)							<0.0001**	
No	435 (75.4)	53 (35.3)	198 (86.8)	181 (85.0)	202 (84.5)	5 (35.7)		1074 (75.6)
Yes	142 (24.6)	97 (64.7)	30 (13.2)	32 (15.0)	37 (15.5)	9 (64.3)		347 (24.4)
Missing	107	280	3	60	12	192		654
ECOG Performance Status at Baseline - n (%)							<0.0001**	
0-1	567 (83.8)	298 (79.9)	216 (94.7)	145 (90.6)	203 (81.9)	174 (87.4)		1603 (85.0)
2-3-4	110 (16.2)	75 (20.1)	12 (5.3)	15 (9.4)	45 (18.1)	25 (12.6)		282 (15.0)
Missing	7	57	3	113	3	7		190
Concomitant Disease - n (%)							<0.0001**	
No	76 (11.2)	38 (9.8)	58 (25.1)	23 (8.5)	32 (13.9)	49 (23.9)		276 (13.8)
Yes	604 (88.8)	351 (90.2)	173 (74.9)	247 (91.5)	199 (86.1)	156 (76.1)		1730 (86.2)
Missing	4	41	0	3	20	1		69
Cardiovascular Disorders - n (%)							<0.0001**	
No	164 (27.2)	117 (33.4)	38 (22.0)	104 (42.3)	59 (29.6)	56 (36.8)		538 (31.2)
Yes	439 (72.8)	233 (66.6)	135 (78.0)	142 (57.7)	140 (70.4)	96 (63.2)		1185 (68.8)
Missing	1	1	0	1	0	4		7
Renal Disorders - n (%)							<0.0001**	
No	546 (90.5)	343 (98.0)	168 (97.1)	221 (89.8)	176 (88.4)	130 (85.5)		1584 (91.9)
Yes	57 (9.5)	7 (2.0)	5 (2.9)	25 (10.2)	23 (11.6)	22 (14.5)		139 (8.1)
Missing	1	1	0	1	0	4		7
Other Neoplasms - n (%)							<0.0001**	
No	530 (87.9)	290 (82.9)	165 (95.4)	226 (91.9)	185 (93.0)	140 (92.1)		1536 (89.1)
Yes	73 (12.1)	60 (17.1)	8 (4.6)	20 (8.1)	14 (7.0)	12 (7.9)		187 (10.9)
Missing	1	1	0	1	0	4		7

Endocrine Disorders - n (%)							<0.0001**	
No	462 (76.6)	341 (97.4)	155 (89.6)	222 (90.2)	149 (74.9)	144 (94.7)		1473 (85.5)
Yes	141 (23.4)	9 (2.6)	18 (10.4)	24 (9.8)	50 (25.1)	8 (5.3)		250 (14.5)
Missing	1	1	0	1	0	4		7
Pulmonary Disease - n (%)							<0.0001**	
No	467 (77.4)	202 (57.7)	130 (75.1)	131 (53.3)	141 (70.9)	99 (65.1)		1170 (67.9)
Yes	136 (22.6)	148 (42.3)	43 (24.9)	115 (46.7)	58 (29.1)	53 (34.9)		553 (32.1)
Missing	1	1	0	1	0	4		7
Autoimmune Disorders - n (%)							<0.0001**	
No	537 (89.1)	335 (95.7)	169 (97.7)	213 (86.6)	158 (79.4)	146 (96.1)		1558 (90.4)
Yes	66 (10.9)	15 (4.3)	4 (2.3)	33 (13.4)	41 (20.6)	6 (3.9)		165 (9.6)
Missing	1	1	0	1	0	4		7
Hepatic Disorders - n (%)							<0.0001**	
No	554 (91.9)	342 (97.7)	172 (99.4)	228 (92.7)	180 (90.5)	145 (95.4)		1621 (94.1)
Yes	49 (8.1)	8 (2.3)	1 (0.6)	18 (7.3)	19 (9.5)	7 (4.6)		102 (5.9)
Missing	1	1	0	1	0	4		7
Infections - n (%)							<0.0001**	
No	490 (81.3)	341 (97.4)	170 (98.3)	200 (81.3)	137 (68.8)	145 (95.4)		1483 (86.1)
Yes	113 (18.7)	9 (2.6)	3 (1.7)	46 (18.7)	62 (31.2)	7 (4.6)		240 (13.9)
Missing	1	1	0	1	0	4		7
Gastrointestinal Disorders - n (%)							<0.0001**	
No	513 (85.1)	342 (97.7)	167 (96.5)	218 (88.6)	168 (84.4)	129 (84.9)		1537 (89.2)
Yes	90 (14.9)	8 (2.3)	6 (3.5)	28 (11.4)	31 (15.6)	23 (15.1)		186 (10.8)
Missing	1	1	0	1	0	4		7
Diabetes - n (%)							<0.0001**	
No	527 (87.4)	287 (82.0)	127 (73.4)	182 (74.0)	156 (78.4)	119 (78.3)		1398 (81.1)
Yes	76 (12.6)	63 (18.0)	46 (26.6)	64 (26.0)	43 (21.6)	33 (21.7)		325 (18.9)
Missing	1	1	0	1	0	4		7

Neurological Disorders - n (%)							<0.0001**	
No	548 (90.9)	343 (98.0)	168 (97.1)	228 (92.7)	179 (89.9)	148 (97.4)		1614 (93.7)
Yes	55 (9.1)	7 (2.0)	5 (2.9)	18 (7.3)	20 (10.1)	4 (2.6)		109 (6.3)
Missing	1	1	0	1	0	4		7
Dislipidemia - n (%)							<0.0001**	
No	497 (82.4)	341 (97.4)	108 (62.4)	201 (81.7)	124 (62.3)	84 (55.3)		1355 (78.6)
Yes	106 (17.6)	9 (2.6)	65 (37.6)	45 (18.3)	75 (37.7)	68 (44.7)		368 (21.4)
Missing	1	1	0	1	0	4		7

Legend**:** N: number of subjects. SD: Standard Deviation. Q1 - Q3: First - Third Quartile. Min - Max: Minimum - Maximum

Table 4. Treatment and tumor characteristics across the centers (INT, GHD, MH, SZMC, UOC, VHIO) and overall, including p-value for comparison across centers. *: p-value of Kruskal-Wallis' Test for Medians **: p-value of Chi-Square Test ***: p-value of Fisher's Exact Test ****: p-value of Fisher's Exact Test based on 10000 Monte Carlo simulations.

Variable	INT (n=684)	GHD (n=430)	MH (n=231)	SZMC (n=273)	UOC (n=251)	VHIO (n=206)	p-value	Overall (n=2075)
Treatment Arm (Immuno Alone vs Immuno + Chemo vs Immuno + Other) - n (%)							<0.0001****	
Immuno Alone	250 (55.9)	126 (46.3)	33 (17.0)	40 (17.4)	72 (34.1)	52 (61.9)		573 (39.8)
Immuno + Chemo	197 (44.1)	140 (51.5)	160 (82.5)	189 (82.2)	139 (65.9)	32 (38.1)		857 (59.6)
Immuno + Other	0 (0.0)	6 (2.2)	1 (0.5)	1 (0.4)	0 (0.0)	0 (0.0)		8 (0.6)
Missing	237	158	37	43	40	122		637
Treatment Arm (Immuno Alone/Immuno + Other vs Immuno + Chemo) - n (%)							<0.0001**	
Immuno Alone/Immuno + Other	250 (55.9)	132 (48.5)	34 (17.5)	41 (17.8)	72 (34.1)	52 (61.9)		581 (40.4)
Immuno + Chemo	197 (44.1)	140 (51.5)	160 (82.5)	189 (82.2)	139 (65.9)	32 (38.1)		857 (59.6)
Missing	237	158	37	43	40	122		637
Age at Treatment Start (Years)							0.0025*	
Mean (SD)	67.4 (9.9)	66.7 (9.6)	67.5 (9.4)	67.2 (9.1)	68.4 (10.0)	64.4 (11.3)		67.0 (9.9)
Median (Q1 - Q3)	68.6 (61.2-74.3)	67.1 (60.1-74.3)	68.8 (61.4-73.9)	68.1 (61.9-73.2)	69.3 (61.8-75.6)	64.7 (57.2-72.6)		67.9 (60.5-74.1)
Min - Max	27.5 - 92.8	34.1 - 92.6	35.6 - 87.0	34.4 - 93.5	35.9 - 91.7	34.6 - 90.0		27.5 - 93.5

Missing	4	13	22	63	125	0		227
Biopsy Type - n (%)							<0.0001**	
Histological	641 (94.8)	140 (42.0)	185 (80.1)	207 (95.4)	43 (17.2)	10 (100.0)		1226 (71.4)
Citological	22 (3.3)	103 (30.9)	14 (6.1)	5 (2.3)	166 (66.4)	0 (0.0)		310 (18.1)
Surgery Specimen	13 (1.9)	90 (27.0)	32 (13.9)	5 (2.3)	41 (16.4)	0 (0.0)		181 (10.5)
Missing	8	97	0	56	1	196		358
Biopsy Site (Lung vs Other) - n (%)							<0.0001**	
Lung	420 (63.2)	275 (84.6)	186 (80.5)	194 (86.6)	135 (54.0)	5 (50.0)		1215 (71.3)
Other	245 (36.8)	50 (15.4)	45 (19.5)	30 (13.4)	115 (46.0)	5 (50.0)		490 (28.7)
Missing	19	105	0	49	1	196		370
PDL1 Percentage - n (%)							<0.0001**	
< 1%	198 (33.3)	69 (24.0)	90 (42.5)	83 (36.6)	101 (45.9)	63 (48.8)		604 (36.2)
1-49%	219 (36.9)	101 (35.1)	73 (34.4)	41 (18.1)	50 (22.7)	23 (17.8)		507 (30.4)
≥ 50%	177 (29.8)	118 (41.0)	49 (23.1)	103 (45.4)	69 (31.4)	43 (33.3)		559 (33.5)
Missing	90	142	19	46	31	77		405
Number of Metastases (1 vs > 1) - n (%)							0.0009**	
1	315 (49.8)	155 (50.5)	132 (67.0)	130 (56.0)	105 (49.5)	107 (52.5)		944 (52.9)
> 1	317 (50.2)	152 (49.5)	65 (33.0)	102 (44.0)	107 (50.5)	97 (47.5)		840 (47.1)
Missing	52	123	34	41	39	2		291
Number of Metastases							<0.0001*	
Mean (SD)	1.8 (1.1)	1.8 (1.1)	1.4 (0.7)	1.7 (1.0)	1.8 (1.0)	1.9 (1.3)		1.8 (1.1)
Median (Q1 - Q3)	2.0 (1.0-2.0)	1.0 (1.0-2.0)	1.0 (1.0-2.0)	1.0 (1.0-2.0)	2.0 (1.0-2.0)	1.0 (1.0-2.0)		1.0 (1.0-2.0)
Min - Max	1.0 - 7.0	1.0 - 7.0	1.0 - 4.0	1.0 - 7.0	1.0 - 6.0	1.0 - 7.0		1.0 - 7.0
Missing	52	123	34	41	39	2		291
Diagnosis Stage (All stages) - n (%)							<0.0001****	
I	36 (5.3)	2 (0.5)	12 (5.2)	2 (0.7)	11 (4.4)	11 (5.4)		74 (3.6)
II	35 (5.2)	3 (0.7)	7 (3.1)	8 (3.0)	16 (6.4)	5 (2.5)		74 (3.6)
III	127 (18.9)	128 (29.9)	54 (23.6)	23 (8.6)	62 (24.7)	41 (20.3)		435 (21.2)
IV	475 (70.6)	295 (68.9)	156 (68.1)	235 (87.7)	162 (64.5)	145 (71.8)		1468 (71.6)
Missing	11	2	2	5	0	4		24
T Stage - n (%)							<0.0001**	

T0+T1+T2	241 (35.9)	119 (27.7)	73 (31.9)	162 (61.6)	213 (85.2)	70 (45.5)		878 (44.0)
T3+T4	430 (64.1)	311 (72.3)	156 (68.1)	101 (38.4)	37 (14.8)	84 (54.5)		1119 (56.0)
Missing	13	0	2	10	1	52		78
N Stage - n (%)							0.0006**	
N0+N1	176 (26.2)	113 (26.3)	75 (32.8)	101 (38.4)	89 (35.6)	53 (34.2)		607 (30.4)
N2+N3	495 (73.8)	317 (73.7)	154 (67.2)	162 (61.6)	161 (64.4)	102 (65.8)		1391 (69.6)
Missing	13	0	2	10	1	51		77
M Stage - n (%)							<0.0001**	
M0	199 (29.6)	131 (30.8)	73 (31.9)	30 (11.5)	90 (36.1)	60 (29.3)		583 (28.6)
M1	474 (70.4)	294 (69.2)	156 (68.1)	230 (88.5)	159 (63.9)	145 (70.7)		1458 (71.4)
Missing	11	5	2	13	2	1		34
Surgery - n (%)							0.7903**	
No	553 (80.8)	345 (80.2)	186 (81.2)	216 (82.4)	205 (81.7)	159 (77.2)		1664 (80.7)
Yes	131 (19.2)	85 (19.8)	43 (18.8)	46 (17.6)	46 (18.3)	47 (22.8)		398 (19.3)
Missing	0	0	2	11	0	0		13
Neoadjuvant Treatment - n (%)							<0.0001**	
No	648 (94.9)	44 (68.8)	213 (93.0)	214 (97.3)	6 (2.7)	3 (21.4)		1128 (78.8)
Yes	35 (5.1)	20 (31.3)	16 (7.0)	6 (2.7)	216 (97.3)	11 (78.6)		304 (21.2)
Missing	1	366	2	53	29	192		643
Immunotherapy - n (%)							<0.0001****	
No	34 (97.1)	19 (95.0)	16 (100.0)	6 (100.0)	4 (1.9)	11 (100.0)		90 (29.6)
Yes	1 (2.9)	1 (5.0)	0 (0.0)	0 (0.0)	212 (98.1)	0 (0.0)		214 (70.4)
Platinum Based Chemotherapy - n (%)							0.0057****	
No	2 (5.7)	1 (5.0)	1 (6.3)	2 (33.3)	56 (25.9)	1 (9.1)		63 (20.7)
Yes	33 (94.3)	19 (95.0)	15 (93.8)	4 (66.7)	160 (74.1)	10 (90.9)		241 (79.3)
Target Therapy - n (%)							0.1982****	
No	35 (100.0)	20 (100.0)	15 (93.8)	5 (83.3)	211 (97.7)	11 (100.0)		297 (97.7)
Yes	0 (0.0)	0 (0.0)	1 (6.3)	1 (16.7)	5 (2.3)	0 (0.0)		7 (2.3)
Adjuvant Therapy - n (%)							0.0002**	
No	642 (93.9)	395 (91.9)	207 (89.6)	249 (94.7)	214 (85.3)	181 (88.3)		1888 (91.5)
Yes	42 (6.1)	35 (8.1)	24 (10.4)	14 (5.3)	37 (14.7)	24 (11.7)		176 (8.5)

Missing	0	0	0	10	0	1		11
Immunotherapy - n (%)							<0.0001****	
No	41 (97.6)	33 (94.3)	22 (91.7)	8 (57.1)	15 (40.5)	24 (100.0)		143 (81.3)
Yes	1 (2.4)	2 (5.7)	2 (8.3)	6 (42.9)	22 (59.5)	0 (0.0)		33 (18.8)
Platinum Based Chemotherapy - n (%)							<0.0001****	
No	1 (2.4)	7 (20.0)	1 (4.2)	1 (7.1)	14 (37.8)	13 (54.2)		37 (21.0)
Yes	41 (97.6)	28 (80.0)	23 (95.8)	13 (92.9)	23 (62.2)	11 (45.8)		139 (79.0)
Target Therapy - n (%)							0.0606****	
No	42 (100.0)	35 (100.0)	24 (100.0)	14 (100.0)	34 (91.9)	24 (100.0)		173 (98.3)
Yes	0 (0.0)	0 (0.0)	0 (0.0)	0 (0.0)	3 (8.1)	0 (0.0)		3 (1.7)
Radiotherapy - n (%)							<0.0001****	
No	28 (66.7)	2 (8.7)	16 (66.7)	6 (66.7)	23 (63.9)	2 (16.7)		77 (52.7)
Yes	14 (33.3)	21 (91.3)	8 (33.3)	3 (33.3)	13 (36.1)	10 (83.3)		69 (47.3)
Missing	0	12	0	5	1	12		30

Legend**:** N: number of subjects. SD: Standard Deviation. Q1 - Q3: First - Third Quartile. Min - Max: Minimum - Maximum.

Table 5. Clinical characteristics of patients in Train, Test and Ext. Val. sets, including p-values for comparison across groups (Train vs ExVal vs Test; Train vs ExVal; Test vs ExVal). * p-value of Kruskal-Wallis Test for Medians; ** p-value of Chi-Square Test; *** p-value of Fisher's Exact Test; **** p-value of Mann-Whitney U test.

Variable	Train (n=1550)	Test (n=274)	ExVal (UOC) (n=251)	p-value Train vs ExVal vs Test	p-value Train vs ExVal	p-value Test vs ExVal
Sex				<0.001**	<0.001**	<0.001**
Female	537 (34.6%)	99 (36.1%)	148 (59.0%)			
Male	1013 (65.4%)	175 (63.9%)	103 (41.0%)			
Missing	0 (0.0%)	0 (0.0%)	0 (0.0%)			
Smoking – Current				<0.001**	<0.001**	<0.001**
0 (No)	871 (61.9%)	152 (60.1%)	212 (84.5%)			
1 (Yes)	537 (38.1%)	101 (39.9%)	39 (15.5%)			
Missing	142 (9.2%)	21 (7.7%)	0 (0.0%)			
Smoking – Former				<0.001**	<0.001**	<0.001**
0 (No)	654 (46.4%)	120 (47.4%)	62 (24.7%)			
1 (Yes)	754 (53.6%)	133 (52.6%)	189 (75.3%)			
Missing	142 (9.2%)	21 (7.7%)	0 (0.0%)			
Smoking – Never				0.798**	0.745**	0.610**
0 (No)	1291 (91.7%)	234 (92.5%)	228 (90.8%)			
1 (Yes)	117 (8.3%)	19 (7.5%)	23 (9.2%)			

Missing	142 (9.2%)	21 (7.7%)	0 (0.0%)			
Brain metastases				0.568**	0.335**	0.620**
0 (No)	1174 (79.8%)	210 (78.9%)	164 (76.6%)			
1 (Yes)	298 (20.2%)	56 (21.1%)	50 (23.4%)			
Missing	78 (5.0%)	8 (2.9%)	37 (14.7%)			
Liver metastases				0.954**	1.000**	0.967**
0 (No)	1234 (83.8%)	221 (83.1%)	179 (83.6%)			
1 (Yes)	238 (16.2%)	45 (16.9%)	35 (16.4%)			
Missing	78 (5.0%)	8 (2.9%)	37 (14.7%)			
Bone metastases				0.945**	1.000**	0.904**
0 (No)	951 (64.6%)	169 (63.5%)	138 (64.5%)			
1 (Yes)	521 (35.4%)	97 (36.5%)	76 (35.5%)			
Missing	78 (5.0%)	8 (2.9%)	37 (14.7%)			
LDH (U/L)	272.0 [199.8–376.0]	260.0 [202.0–369.0]	238.0 [182.5–307.5]	0.118*	0.040****	0.052****
Missing	498 (32.1%)	91 (33.2%)	216 (86.1%)			
NLR	4.6 [3.0–7.8]	4.4 [2.9–7.0]	4.5 [2.6–7.9]	0.325*	0.365****	0.793****
Missing	182 (11.7%)	32 (11.7%)	0 (0.0%)			
ECOG PS				<0.001***	<0.001***	<0.001**
0	470 (33.8%)	90 (36.3%)	6 (2.4%)			
1	725 (52.2%)	115 (46.4%)	197 (79.4%)			
2	184 (13.2%)	37 (14.9%)	41 (16.5%)			
3	8 (0.6%)	6 (2.4%)	4 (1.6%)			
4	2 (0.1%)	0 (0.0%)	0 (0.0%)			
Missing	161 (10.4%)	26 (9.5%)	3 (1.2%)			
PD-L1 category				0.010**	0.004**	0.014**
<1%	428 (34.7%)	75 (34.9%)	101 (45.9%)			
1-49%	383 (31.0%)	74 (34.4%)	50 (22.7%)			
≥ 50%	424 (34.3%)	66 (30.7%)	69 (31.4%)			
Missing	315 (20.3%)	59 (21.5%)	31 (12.4%)			
Histology squamous				<0.001**	<0.001**	<0.001**
No (0)	1167 (76.1%)	201 (74.2%)	220 (89.1%)			
Yes (1)	367 (23.9%)	70 (25.8%)	27 (10.9%)			
Missing	16 (1.0%)	3 (1.1%)	4 (1.6%)			
IO + ChT				<0.001**	<0.001**	<0.001**
No (0)	904 (58.3%)	161 (58.8%)	96 (38.2%)			
Yes (1)	646 (41.7%)	113 (41.2%)	155 (61.8%)			
Missing	0 (0.0%)	0 (0.0%)	0 (0.0%)			
Therapy line				<0.001**	<0.001**	<0.001**
1 (C2)	1046 (67.5%)	181 (66.1%)	210 (83.7%)			
2 (C3)	344 (22.2%)	66 (24.1%)	36 (14.3%)			
≥ 3 (C3)	160 (10.3%)	27 (9.9%)	5 (2.0%)			
Missing	0 (0.0%)	0 (0.0%)	0 (0.0%)			
Age at IO start	67.9 [60.3 - 73.8]	67.4 [61.1 - 74.7]	69.3 [61.9 - 75.6]	0.19*	0.074****	0.24****

Table 6. Clinical characteristics of patients in Train, Test and ExVal sets, including p-values for comparison across groups (Train vs ExVal vs Test; Train vs ExVal; Test vs ExVal) for OS24 outcome. * p-value of Kruskal-Wallis Test for Medians; ** p-value of Chi-Square Test; *** p-value of Fisher's Exact Test.

Variable	Train (n= 1283)	Test (n=226)	ExVal (n=190)	p-value Train vs ExVal vs Test
Sex				<0.001**
Female	450 (35.1%)	85 (37.6%)	119 (62.6%)	
Male	833 (64.9%)	141 (62.4%)	71 (37.4%)	
Missing	0 (0.0%)	0 (0.0%)	0 (0.0%)	
Smoking – Current				<0.001**
0 (No)	728 (63.0%)	127 (61.7%)	164 (86.3%)	
1 (Yes)	427 (37.0%)	79 (38.3%)	26 (13.7%)	
Missing	128 (10.0%)	20 (8.8%)	0 (0.0%)	
Smoking – Former				<0.001**
0 (No)	517 (44.8%)	94 (45.6%)	47 (24.7%)	
1 (Yes)	638 (55.2%)	112 (54.4%)	143 (75.3%)	
Missing	128 (10.0%)	20 (8.8%)	0 (0.0%)	
Smoking – Never				0.279**
0 (No)	1065 (92.2%)	191 (92.7%)	169 (88.9%)	
1 (Yes)	90 (7.8%)	15 (7.3%)	21 (11.1%)	
Missing	128 (10.0%)	20 (8.8%)	0 (0.0%)	
Brain metastases				0.393**
0 (No)	981 (80.1%)	173 (78.6%)	124 (75.6%)	
1 (Yes)	244 (19.9%)	47 (21.4%)	40 (24.4%)	
Missing	58 (4.5%)	6 (2.7%)	26 (13.7%)	
Liver metastases				0.492**
0 (No)	1010 (82.4%)	181 (82.3%)	129 (78.7%)	
1 (Yes)	215 (17.6%)	39 (17.7%)	35 (21.3%)	
Missing	58 (4.5%)	6 (2.7%)	26 (13.7%)	
Bone metastases				0.812**
0 (No)	780 (63.7%)	138 (62.7%)	108 (65.9%)	
1 (Yes)	445 (36.3%)	82 (37.3%)	56 (34.1%)	
Missing	58 (4.5%)	6 (2.7%)	26 (13.7%)	
LDH (U/L)	290.5 [204.0–392.0]	285.5 [208.5–386.0]	231.0 [182.5–307.5]	0.027*
Missing	395 (30.8%)	72 (31.9%)	159 (83.7%)	
NLR	4.8 [3.2–8.0]	4.6 [3.2–7.2]	4.8 [2.7–8.4]	0.698*
Missing	134 (10.4%)	26 (11.5%)	0 (0.0%)	
ECOG PS				<0.001***
0	347 (30.1%)	70 (34.0%)	5 (2.7%)	
1	629 (54.6%)	97 (47.1%)	144 (77.0%)	
2	167 (14.5%)	33 (16.0%)	34 (18.2%)	
3	8 (0.7%)	6 (2.9%)	4 (2.1%)	
4	2 (0.2%)	0 (0.0%)	0 (0.0%)	
Missing	130 (10.1%)	20 (8.8%)	3 (1.6%)	
PD-L1 category				0.013**
<1%	342 (34.7%)	57 (33.3%)	74 (45.1%)	

1-49%	317 (32.2%)	65 (38.0%)	36 (22.0%)	
≥ 50%	327 (33.2%)	49 (28.7%)	54 (32.9%)	
Missing	297 (23.1%)	55 (24.3%)	26 (13.7%)	

Table 7. Clinical characteristics of patients in Train, Test and ExVal sets, including p-values for comparison across groups (Train vs ExVal vs Test; Train vs ExVal; Test vs ExVal) for OS6 outcome. * p-value of Kruskal-Wallis Test for Medians; ** p-value of Chi-Square Test; *** p-value of Fisher's Exact Test.

Variable	Train (n=1473)	Test (n=259)	ExVal (n=240)	p-value Train vs ExVal vs Test
Sex				<0.001**
Female	518 (35.2%)	93 (35.9%)	144 (60.0%)	
Male	955 (64.8%)	166 (64.1%)	96 (40.0%)	
Missing	0 (0.0%)	0 (0.0%)	0 (0.0%)	
Smoking – Current				<0.001**
0 (No)	825 (61.8%)	142 (59.7%)	204 (85.0%)	
1 (Yes)	510 (38.2%)	96 (40.3%)	36 (15.0%)	
Missing	138 (9.4%)	21 (8.1%)	0 (0.0%)	
Smoking – Former				<0.001**
0 (No)	618 (46.3%)	114 (47.9%)	59 (24.6%)	
1 (Yes)	717 (53.7%)	124 (52.1%)	181 (75.4%)	
Missing	138 (9.4%)	21 (8.1%)	0 (0.0%)	
Smoking – Never				0.685**
0 (No)	1227 (91.9%)	220 (92.4%)	217 (90.4%)	
1 (Yes)	108 (8.1%)	18 (7.6%)	23 (9.6%)	
Missing	138 (9.4%)	21 (8.1%)	0 (0.0%)	
Brain metastases				0.692**
0 (No)	1113 (79.6%)	199 (79.0%)	157 (77.0%)	
1 (Yes)	286 (20.4%)	53 (21.0%)	47 (23.0%)	
Missing	74 (5.0%)	7 (2.7%)	36 (15.0%)	
Liver metastases				0.692**
0 (No)	1168 (83.5%)	210 (83.3%)	169 (82.8%)	
1 (Yes)	231 (16.5%)	42 (16.7%)	35 (17.2%)	
Missing	74 (5.0%)	7 (2.7%)	36 (15.0%)	
Bone metastases				0.831**
0 (No)	906 (64.8%)	159 (63.1%)	134 (65.7%)	
1 (Yes)	493 (35.2%)	93 (36.9%)	70 (34.3%)	
Missing	74 (5.0%)	7 (2.7%)	36 (15.0%)	
LDH (U/L)	274.5 [200.0–376.2]	270.0 [203.0–379.0]	238.5 [188.8–308.2]	0.151*
Missing	465 (31.6%)	86 (33.2%)	206 (85.8%)	
NLR	4.6 [3.0–7.8]	4.4 [2.9–7.1]	4.5 [2.6–7.8]	0.278*
Missing	163 (11.1%)	30 (11.6%)	0 (0.0%)	
ECOG PS				<0.001***
0	437 (33.1%)	84 (35.7%)	6 (2.5%)	
1	697 (52.8%)	109 (46.4%)	188 (79.3%)	
2	176 (13.3%)	36 (15.3%)	39 (16.5%)	
3	8 (0.6%)	6 (2.6%)	4 (1.7%)	
4	2 (0.2%)	0 (0.0%)	0 (0.0%)	
Missing	153 (10.4%)	24 (9.3%)	3 (1.2%)	
PD-L1 category				0.004**
<1%	399 (34.2%)	65 (32.3%)	98 (46.7%)	
1-49%	365 (31.3%)	72 (35.8%)	48 (22.9%)	
≥ 50%	401 (34.4%)	64 (31.8%)	64 (30.5%)	

Missing	308 (20.9%)	58 (22.4%)	30 (12.5%)	

Table 8. Clinical characteristics of patients in Train, Test and ExVal sets, including p-values for comparison across groups (Train vs ExVal vs Test; Train vs ExVal; Test vs ExVal) for DCR outcome. * p-value of Kruskal-Wallis Test for Medians; ** p-value of Chi-Square Test; *** p-value of Fisher's Exact Test.

Variable	Train (n=1544)	Test (n=273)	ExVal (n=251)	p-value Train vs ExVal vs Test
Sex				<0.001**
Female	536 (34.7%)	98 (35.9%)	148 (59.0%)	
Male	1008 (65.3%)	175 (64.1%)	103 (41.0%)	
Missing	0 (0.0%)	0 (0.0%)	0 (0.0%)	
Smoking – Current				<0.001**
0 (No)	868 (61.9%)	152 (60.3%)	212 (84.5%)	
1 (Yes)	535 (38.1%)	100 (39.7%)	39 (15.5%)	
Missing	141 (9.1%)	21 (7.7%)	0 (0.0%)	
Smoking – Former				<0.001**
0 (No)	651 (46.4%)	119 (47.2%)	62 (24.7%)	
1 (Yes)	752 (53.6%)	133 (52.8%)	189 (75.3%)	
Missing	141 (9.1%)	21 (7.7%)	0 (0.0%)	
Smoking – Never				0.803**
0 (No)	1287 (91.7%)	233 (92.5%)	228 (90.8%)	
1 (Yes)	116 (8.3%)	19 (7.5%)	23 (9.2%)	
Missing	141 (9.1%)	21 (7.7%)	0 (0.0%)	
Brain metastases				0.568**
0 (No)	1174 (79.8%)	210 (78.9%)	164 (76.6%)	
1 (Yes)	298 (20.2%)	56 (21.1%)	50 (23.4%)	
Missing	72 (4.7%)	7 (2.6%)	37 (14.7%)	
Liver metastases				0.954**
0 (No)	1234 (83.8%)	221 (83.1%)	179 (83.6%)	
1 (Yes)	238 (16.2%)	45 (16.9%)	35 (16.4%)	
Missing	72 (4.7%)	7 (2.6%)	37 (14.7%)	
Bone metastases				0.945**
0 (No)	951 (64.6%)	169 (63.5%)	138 (64.5%)	
1 (Yes)	521 (35.4%)	97 (36.5%)	76 (35.5%)	
Missing	72 (4.7%)	7 (2.6%)	37 (14.7%)	
LDH (U/L)	272.0 [199.5–376.0]	260.0 [202.0–369.0]	238.0 [182.5–307.5]	0.119*
Missing	493 (31.9%)	90 (33.0%)	216 (86.1%)	
NLR	4.6 [3.0–7.8]	4.4 [2.9–7.0]	4.5 [2.6–7.9]	0.318*
Missing	177 (11.5%)	31 (11.4%)	0 (0.0%)	
ECOG PS				<0.001***
0	469 (33.8%)	90 (36.3%)	6 (2.4%)	
1	725 (52.2%)	115 (46.4%)	197 (79.4%)	
2	184 (13.3%)	37 (14.9%)	41 (16.5%)	
3	8 (0.6%)	6 (2.4%)	4 (1.6%)	
4	2 (0.1%)	0 (0.0%)	0 (0.0%)	
Missing	156 (10.1%)	25 (9.2%)	3 (1.2%)	
PD-L1 category				0.010**
<1%	428 (34.8%)	75 (35.0%)	101 (45.9%)	
1-49%	379 (30.8%)	74 (34.6%)	50 (22.7%)	
≥ 50%	422 (34.3%)	65 (30.4%)	69 (31.4%)	
Missing	315 (20.4%)	59 (21.6%)	31 (12.4%)	

Table 9. Clinical characteristics of patients in Train set for OS24 across centers (GHD, INT, MH, SZMC and VHIO), including p-value for comparison across centers. * p-value of Kruskal-Wallis Test for Medians; ** p-value of Chi-Square Test; *** p-value of Fisher's Exact Test.

Variable	GHD (n=362)	INT (n=510)	MH (n=108)	SZMC (n=139)	VHIO (n=164)	p-value
Sex						0.189**
Female	140 (38.7%)	178 (34.9%)	28 (25.9%)	47 (33.8%)	57 (34.8%)	
Male	222 (61.3%)	332 (65.1%)	80 (74.1%)	92 (66.2%)	107 (65.2%)	
Missing	0 (0.0%)	0 (0.0%)	0 (0.0%)	0 (0.0%)	0 (0.0%)	
Smoking – Current						<0.001**
0 (No)	136 (47.7%)	348 (73.4%)	52 (49.1%)	73 (57.9%)	119 (72.6%)	
1 (Yes)	149 (52.3%)	126 (26.6%)	54 (50.9%)	53 (42.1%)	45 (27.4%)	
Missing	77 (21.3%)	36 (7.1%)	2 (1.9%)	13 (9.4%)	0 (0.0%)	
Smoking – Former						<0.001**
0 (No)	164 (57.5%)	158 (33.3%)	59 (55.7%)	73 (57.9%)	63 (38.4%)	
1 (Yes)	121 (42.5%)	316 (66.7%)	47 (44.3%)	53 (42.1%)	101 (61.6%)	
Missing	77 (21.3%)	36 (7.1%)	2 (1.9%)	13 (9.4%)	0 (0.0%)	
Smoking – Never						0.001**
0 (No)	270 (94.7%)	442 (93.2%)	101 (95.3%)	106 (84.1%)	146 (89.0%)	
1 (Yes)	15 (5.3%)	32 (6.8%)	5 (4.7%)	20 (15.9%)	18 (11.0%)	
Missing	77 (21.3%)	36 (7.1%)	2 (1.9%)	13 (9.4%)	0 (0.0%)	
Brain metastases						0.437**
0 (No)	273 (82.0%)	394 (78.3%)	92 (85.2%)	95 (79.8%)	127 (78.4%)	
1 (Yes)	60 (18.0%)	109 (21.7%)	16 (14.8%)	24 (20.2%)	35 (21.6%)	
Missing	29 (8.0%)	7 (1.4%)	0 (0.0%)	20 (14.4%)	2 (1.2%)	
Liver metastases						0.722**
0 (No)	276 (82.9%)	412 (81.9%)	89 (82.4%)	103 (86.6%)	130 (80.2%)	
1 (Yes)	57 (17.1%)	91 (18.1%)	19 (17.6%)	16 (13.4%)	32 (19.8%)	
Missing	29 (8.0%)	7 (1.4%)	0 (0.0%)	20 (14.4%)	2 (1.2%)	
Bone metastases						<0.001**
0 (No)	241 (72.4%)	292 (58.1%)	67 (62.0%)	73 (61.3%)	107 (66.0%)	
1 (Yes)	92 (27.6%)	211 (41.9%)	41 (38.0%)	46 (38.7%)	55 (34.0%)	
Missing	29 (8.0%)	7 (1.4%)	0 (0.0%)	20 (14.4%)	2 (1.2%)	
LDH (U/L)	217.5 [182.0–288.0]	324.0 [258.5–419.0]	210.5 [174.2–332.5]	251.0 [194.0–393.0]	402.5 [326.0–549.5]	<0.001*
Missing	110 (30.4%)	87 (17.1%)	68 (63.0%)	18 (12.9%)	112 (68.3%)	
NLR	5.9 [3.9–9.1]	4.6 [3.1–7.5]	3.6 [2.8–5.6]	3.9 [2.7–6.9]	4.5 [3.2–7.6]	<0.001*
Missing	33 (9.1%)	36 (7.1%)	14 (13.0%)	9 (6.5%)	42 (25.6%)	
ECOG PS						<0.001***
0	74 (23.5%)	137 (27.1%)	66 (61.1%)	34 (50.7%)	36 (22.9%)	
1	177 (56.2%)	289 (57.1%)	37 (34.3%)	26 (38.8%)	100 (63.7%)	
2	57 (18.1%)	78 (15.4%)	5 (4.6%)	6 (9.0%)	21 (13.4%)	
3	6 (1.9%)	2 (0.4%)	0 (0.0%)	0 (0.0%)	0 (0.0%)	
4	1 (0.3%)	0 (0.0%)	0 (0.0%)	1 (1.5%)	0 (0.0%)	
Missing	47 (13.0%)	4 (0.8%)	0 (0.0%)	72 (51.8%)	7 (4.3%)	
PD-L1 category						<0.001**

<1%	60 (24.9%)	149 (33.7%)	40 (40.8%)	43 (39.4%)	50 (52.1%)	
1-49%	80 (33.2%)	166 (37.6%)	35 (35.7%)	18 (16.5%)	18 (18.8%)	
≥ 50%	101 (41.9%)	127 (28.7%)	23 (23.5%)	48 (44.0%)	28 (29.2%)	
Missing	121 (33.4%)	68 (13.3%)	10 (9.3%)	30 (21.6%)	68 (41.5%)	

Table 10. Clinical characteristics of patients in Test set for OS24 across centers (GHD, INT, MH, SZMC and VHIO), including p-value for comparison across centers. * p-value of Kruskal-Wallis Test for Medians; ** p-value of Chi-Square Test; *** p-value of Fisher's Exact Test.

Variable	GHD (n=63)	INT (n=97)	MH (n=17)	SZMC (n=20)	VHIO (n=29)	p-value
Sex						0.225**
Female	27 (42.9%)	41 (42.3%)	4 (23.5%)	5 (25.0%)	8 (27.6%)	
Male	36 (57.1%)	56 (57.7%)	13 (76.5%)	15 (75.0%)	21 (72.4%)	
Missing	0 (0.0%)	0 (0.0%)	0 (0.0%)	0 (0.0%)	0 (0.0%)	
Smoking – Current						0.017**
0 (No)	25 (49.0%)	66 (73.3%)	8 (50.0%)	9 (45.0%)	19 (65.5%)	
1 (Yes)	26 (51.0%)	24 (26.7%)	8 (50.0%)	11 (55.0%)	10 (34.5%)	
Missing	12 (19.0%)	7 (7.2%)	1 (5.9%)	0 (0.0%)	0 (0.0%)	
Smoking – Former						0.022**
0 (No)	30 (58.8%)	30 (33.3%)	9 (56.2%)	12 (60.0%)	13 (44.8%)	
1 (Yes)	21 (41.2%)	60 (66.7%)	7 (43.8%)	8 (40.0%)	16 (55.2%)	
Missing	12 (19.0%)	7 (7.2%)	1 (5.9%)	0 (0.0%)	0 (0.0%)	
Smoking – Never						<0.001***
0 (No)	47 (92.2%)	84 (93.3%)	15 (93.8%)	19 (95.0%)	26 (89.7%)	
1 (Yes)	4 (7.8%)	6 (6.7%)	1 (6.2%)	1 (5.0%)	3 (10.3%)	
Missing	12 (19.0%)	7 (7.2%)	1 (5.9%)	0 (0.0%)	0 (0.0%)	
Brain metastases						0.484**
0 (No)	52 (85.2%)	70 (73.7%)	12 (75.0%)	16 (84.2%)	23 (79.3%)	
1 (Yes)	9 (14.8%)	25 (26.3%)	4 (25.0%)	3 (15.8%)	6 (20.7%)	
Missing	2 (3.2%)	2 (2.1%)	1 (5.9%)	1 (5.0%)	0 (0.0%)	
Liver metastases						<0.001***
0 (No)	49 (80.3%)	76 (80.0%)	12 (75.0%)	18 (94.7%)	26 (89.7%)	
1 (Yes)	12 (19.7%)	19 (20.0%)	4 (25.0%)	1 (5.3%)	3 (10.3%)	
Missing	2 (3.2%)	2 (2.1%)	1 (5.9%)	1 (5.0%)	0 (0.0%)	
Bone metastases						0.077**
0 (No)	43 (70.5%)	51 (53.7%)	13 (81.2%)	14 (73.7%)	17 (58.6%)	
1 (Yes)	18 (29.5%)	44 (46.3%)	3 (18.8%)	5 (26.3%)	12 (41.4%)	
Missing	2 (3.2%)	2 (2.1%)	1 (5.9%)	1 (5.0%)	0 (0.0%)	
LDH (U/L)	221.5 [197.2–321.0]	342.0 [267.0–423.0]	233.5 [185.8–254.8]	225.0 [205.0–252.2]	441.0 [309.0–461.5]	<0.001*
Missing	19 (30.2%)	20 (20.6%)	9 (52.9%)	2 (10.0%)	22 (75.9%)	
NLR	5.1 [3.5–8.1]	5.0 [3.5–7.1]	3.5 [2.3–4.6]	3.8 [3.1–5.7]	4.5 [3.3–6.7]	0.237*
Missing	5 (7.9%)	9 (9.3%)	3 (17.6%)	1 (5.0%)	8 (27.6%)	
ECOG PS						0.004***
0	19 (35.2%)	25 (26.3%)	11 (68.8%)	9 (75.0%)	6 (20.7%)	
1	24 (44.4%)	47 (49.5%)	5 (31.2%)	2 (16.7%)	19 (65.5%)	
2	7 (13.0%)	21 (22.1%)	0 (0.0%)	1 (8.3%)	4 (13.8%)	
3	4 (7.4%)	2 (2.1%)	0 (0.0%)	0 (0.0%)	0 (0.0%)	

4	0 (0.0%)	0 (0.0%)	0 (0.0%)	0 (0.0%)	0 (0.0%)	
Missing	9 (14.3%)	2 (2.1%)	1 (5.9%)	8 (40.0%)	0 (0.0%)	
PD-L1 category						<0.001***
<1%	9 (21.4%)	24 (30.8%)	8 (53.3%)	7 (43.8%)	9 (45.0%)	
1-49%	20 (47.6%)	33 (42.3%)	5 (33.3%)	4 (25.0%)	3 (15.0%)	
≥ 50%	13 (31.0%)	21 (26.9%)	2 (13.3%)	5 (31.2%)	8 (40.0%)	
Missing	21 (33.3%)	19 (19.6%)	2 (11.8%)	4 (20.0%)	9 (31.0%)	

Table 11. Clinical characteristics of patients in Train set for OS6 across centers (GHD, INT, MH, SZMC and VHIO), including p-value for comparison across centers. * p-value of Kruskal-Wallis Test for Medians; ** p-value of Chi-Square Test; *** p-value of Fisher's Exact Test.

Variable	GHD (n=365)	INT (n=567)	MH (n=165)	SZMC (n=202)	VHIO (n=174)	p-value
Sex						0.022**
Female	141 (38.6%)	201 (35.4%)	40 (24.2%)	77 (38.1%)	59 (33.9%)	
Male	224 (61.4%)	366 (64.6%)	125 (75.8%)	125 (61.9%)	115 (66.1%)	
Missing	0 (0.0%)	0 (0.0%)	0 (0.0%)	0 (0.0%)	0 (0.0%)	
Smoking – Current						<0.001**
0 (No)	138 (48.1%)	380 (72.0%)	72 (45.3%)	107 (57.2%)	128 (73.6%)	
1 (Yes)	149 (51.9%)	148 (28.0%)	87 (54.7%)	80 (42.8%)	46 (26.4%)	
Missing	78 (21.4%)	39 (6.9%)	6 (3.6%)	15 (7.4%)	0 (0.0%)	
Smoking – Former						<0.001**
0 (No)	164 (57.1%)	190 (36.0%)	94 (59.1%)	105 (56.1%)	65 (37.4%)	
1 (Yes)	123 (42.9%)	338 (64.0%)	65 (40.9%)	82 (43.9%)	109 (62.6%)	
Missing	78 (21.4%)	39 (6.9%)	6 (3.6%)	15 (7.4%)	0 (0.0%)	
Smoking – Never						0.005**
0 (No)	272 (94.8%)	486 (92.0%)	152 (95.6%)	162 (86.6%)	155 (89.1%)	
1 (Yes)	15 (5.2%)	42 (8.0%)	7 (4.4%)	25 (13.4%)	19 (10.9%)	
Missing	78 (21.4%)	39 (6.9%)	6 (3.6%)	15 (7.4%)	0 (0.0%)	
Brain metastases						0.697**
0 (No)	276 (82.1%)	442 (79.1%)	130 (79.8%)	132 (78.1%)	133 (77.3%)	
1 (Yes)	60 (17.9%)	117 (20.9%)	33 (20.2%)	37 (21.9%)	39 (22.7%)	
Missing	29 (7.9%)	8 (1.4%)	2 (1.2%)	33 (16.3%)	2 (1.1%)	
Liver metastases						0.069**
0 (No)	279 (83.0%)	455 (81.4%)	143 (87.7%)	151 (89.3%)	140 (81.4%)	
1 (Yes)	57 (17.0%)	104 (18.6%)	20 (12.3%)	18 (10.7%)	32 (18.6%)	
Missing	29 (7.9%)	8 (1.4%)	2 (1.2%)	33 (16.3%)	2 (1.1%)	
Bone metastases						<0.001**
0 (No)	243 (72.3%)	325 (58.1%)	109 (66.9%)	115 (68.0%)	114 (66.3%)	
1 (Yes)	93 (27.7%)	234 (41.9%)	54 (33.1%)	54 (32.0%)	58 (33.7%)	
Missing	29 (7.9%)	8 (1.4%)	2 (1.2%)	33 (16.3%)	2 (1.1%)	
LDH (U/L)	217.5 [182.0–287.0]	314.0 [233.0–406.0]	217.0 [181.0–333.0]	238.0 [193.0–315.0]	390.0 [306.0–531.0]	<0.001*
Missing	111 (30.4%)	102 (18.0%)	106 (64.2%)	29 (14.4%)	117 (67.2%)	
NLR	5.9 [3.9–9.1]	4.6 [3.1–7.5]	3.3 [2.4–5.4]	3.9 [2.7–6.8]	4.6 [3.3–7.6]	<0.001*
Missing	34 (9.3%)	45 (7.9%)	24 (14.5%)	17 (8.4%)	43 (24.7%)	

ECOG PS						<0.001***
0	75 (23.7%)	162 (28.8%)	107 (65.6%)	57 (51.4%)	36 (21.6%)	
1	178 (56.2%)	319 (56.8%)	48 (29.4%)	42 (37.8%)	110 (65.9%)	
2	57 (18.0%)	79 (14.1%)	8 (4.9%)	11 (9.9%)	21 (12.6%)	
3	6 (1.9%)	2 (0.4%)	0 (0.0%)	0 (0.0%)	0 (0.0%)	
4	1 (0.3%)	0 (0.0%)	0 (0.0%)	1 (0.9%)	0 (0.0%)	
Missing	48 (13.2%)	5 (0.9%)	2 (1.2%)	91 (45.0%)	7 (4.0%)	
PD-L1 category						<0.001**
<1%	60 (24.6%)	166 (33.5%)	59 (39.3%)	60 (35.5%)	54 (50.9%)	
1-49%	80 (32.8%)	181 (36.5%)	54 (36.0%)	31 (18.3%)	19 (17.9%)	
≥ 50%	104 (42.6%)	149 (30.0%)	37 (24.7%)	78 (46.2%)	33 (31.1%)	
Missing	121 (33.2%)	71 (12.5%)	15 (9.1%)	33 (16.3%)	68 (39.1%)	

Table 12. Clinical characteristics of patients in Test set for OS6 across centers (GHD, INT, MH, SZMC and VHIO), including p-value for comparison across centers. * p-value of Kruskal-Wallis Test for Medians; ** p-value of Chi-Square Test; *** p-value of Fisher's Exact Test.

Variable	GHD (n=65)	INT (n=101)	MH (n=26)	SZMC (n=36)	VHIO (n=31)	p-value
Sex						0.236**
Female	27 (41.5%)	41 (40.6%)	6 (23.1%)	11 (30.6%)	8 (25.8%)	
Male	38 (58.5%)	60 (59.4%)	20 (76.9%)	25 (69.4%)	23 (74.2%)	
Missing	0 (0.0%)	0 (0.0%)	0 (0.0%)	0 (0.0%)	0 (0.0%)	
Smoking – Current						0.011**
0 (No)	25 (47.2%)	67 (71.3%)	13 (52.0%)	16 (45.7%)	21 (67.7%)	
1 (Yes)	28 (52.8%)	27 (28.7%)	12 (48.0%)	19 (54.3%)	10 (32.3%)	
Missing	12 (18.5%)	7 (6.9%)	1 (3.8%)	1 (2.8%)	0 (0.0%)	
Smoking – Former						0.006**
0 (No)	32 (60.4%)	33 (35.1%)	13 (52.0%)	23 (65.7%)	13 (41.9%)	
1 (Yes)	21 (39.6%)	61 (64.9%)	12 (48.0%)	12 (34.3%)	18 (58.1%)	
Missing	12 (18.5%)	7 (6.9%)	1 (3.8%)	1 (2.8%)	0 (0.0%)	
Smoking – Never						<0.001***
0 (No)	49 (92.5%)	88 (93.6%)	24 (96.0%)	31 (88.6%)	28 (90.3%)	
1 (Yes)	4 (7.5%)	6 (6.4%)	1 (4.0%)	4 (11.4%)	3 (9.7%)	
Missing	12 (18.5%)	7 (6.9%)	1 (3.8%)	1 (2.8%)	0 (0.0%)	
Brain metastases						0.559**
0 (No)	54 (85.7%)	73 (74.5%)	20 (80.0%)	28 (80.0%)	24 (77.4%)	
1 (Yes)	9 (14.3%)	25 (25.5%)	5 (20.0%)	7 (20.0%)	7 (22.6%)	
Missing	2 (3.1%)	3 (3.0%)	1 (3.8%)	1 (2.8%)	0 (0.0%)	
Liver metastases						<0.001***
0 (No)	50 (79.4%)	79 (80.6%)	21 (84.0%)	32 (91.4%)	28 (90.3%)	
1 (Yes)	13 (20.6%)	19 (19.4%)	4 (16.0%)	3 (8.6%)	3 (9.7%)	
Missing	2 (3.1%)	3 (3.0%)	1 (3.8%)	1 (2.8%)	0 (0.0%)	
Bone metastases						0.075**
0 (No)	44 (69.8%)	53 (54.1%)	20 (80.0%)	24 (68.6%)	18 (58.1%)	
1 (Yes)	19 (30.2%)	45 (45.9%)	5 (20.0%)	11 (31.4%)	13 (41.9%)	

Missing	2 (3.1%)	3 (3.0%)	1 (3.8%)	1 (2.8%)	0 (0.0%)	
LDH (U/L)	219.0 [183.8–316.8]	342.0 [261.8–419.8]	219.0 [193.0–249.0]	222.0 [202.5–257.5]	387.0 [264.8–451.8]	<0.001*
Missing	19 (29.2%)	23 (22.8%)	17 (65.4%)	4 (11.1%)	23 (74.2%)	
NLR	5.1 [3.4–8.1]	4.8 [3.4–7.0]	3.1 [2.1–4.4]	3.7 [2.9–5.6]	4.5 [3.0–7.5]	0.034*
Missing	5 (7.7%)	9 (8.9%)	6 (23.1%)	2 (5.6%)	8 (25.8%)	
ECOG PS						<0.001***
0	20 (35.7%)	25 (25.3%)	19 (76.0%)	13 (54.2%)	7 (22.6%)	
1	25 (44.6%)	49 (49.5%)	5 (20.0%)	10 (41.7%)	20 (64.5%)	
2	7 (12.5%)	23 (23.2%)	1 (4.0%)	1 (4.2%)	4 (12.9%)	
3	4 (7.1%)	2 (2.0%)	0 (0.0%)	0 (0.0%)	0 (0.0%)	
4	0 (0.0%)	0 (0.0%)	0 (0.0%)	0 (0.0%)	0 (0.0%)	
Missing	9 (13.8%)	2 (2.0%)	1 (3.8%)	12 (33.3%)	0 (0.0%)	
PD-L1 category						0.061**
<1%	9 (20.5%)	25 (30.5%)	11 (45.8%)	11 (37.9%)	9 (40.9%)	
1-49%	21 (47.7%)	34 (41.5%)	8 (33.3%)	6 (20.7%)	3 (13.6%)	
≥ 50%	14 (31.8%)	23 (28.0%)	5 (20.8%)	12 (41.4%)	10 (45.5%)	
Missing	21 (32.3%)	19 (18.8%)	2 (7.7%)	7 (19.4%)	9 (29.0%)	

Table 13. Clinical characteristics of patients in Train set for DCR across centers (GHD, INT, MH, SZMC and VHIO), including p-value for comparison across centers. * p-value of Kruskal-Wallis Test for Medians; ** p-value of Chi-Square Test; *** p-value of Fisher's Exact Test.

Variable	GHD (n=365)	INT (n=580)	MH (n=194)	SZMC (n=230)	VHIO (n=175)	p-value
Sex						0.010**
Female	141 (38.6%)	206 (35.5%)	46 (23.7%)	83 (36.1%)	60 (34.3%)	
Male	224 (61.4%)	374 (64.5%)	148 (76.3%)	147 (63.9%)	115 (65.7%)	
Missing	0 (0.0%)	0 (0.0%)	0 (0.0%)	0 (0.0%)	0 (0.0%)	
Smoking – Current						<0.001**
0 (No)	138 (48.1%)	388 (71.6%)	89 (47.8%)	124 (58.2%)	129 (73.7%)	
1 (Yes)	149 (51.9%)	154 (28.4%)	97 (52.2%)	89 (41.8%)	46 (26.3%)	
Missing	78 (21.4%)	38 (6.6%)	8 (4.1%)	17 (7.4%)	0 (0.0%)	
Smoking – Former						<0.001**
0 (No)	164 (57.1%)	197 (36.3%)	110 (59.1%)	114 (53.5%)	66 (37.7%)	
1 (Yes)	123 (42.9%)	345 (63.7%)	76 (40.9%)	99 (46.5%)	109 (62.3%)	
Missing	78 (21.4%)	38 (6.6%)	8 (4.1%)	17 (7.4%)	0 (0.0%)	
Smoking – Never						0.046**
0 (No)	272 (94.8%)	499 (92.1%)	173 (93.0%)	188 (88.3%)	155 (88.6%)	
1 (Yes)	15 (5.2%)	43 (7.9%)	13 (7.0%)	25 (11.7%)	20 (11.4%)	
Missing	78 (21.4%)	38 (6.6%)	8 (4.1%)	17 (7.4%)	0 (0.0%)	
Brain metastases						0.639**
0 (No)	276 (82.1%)	452 (78.7%)	158 (81.4%)	154 (79.0%)	134 (77.5%)	
1 (Yes)	60 (17.9%)	122 (21.3%)	36 (18.6%)	41 (21.0%)	39 (22.5%)	
Missing	29 (7.9%)	6 (1.0%)	0 (0.0%)	35 (15.2%)	2 (1.1%)	
Liver metastases						0.017**
0 (No)	279 (83.0%)	467 (81.4%)	171 (88.1%)	176 (90.3%)	141 (81.5%)	
1 (Yes)	57 (17.0%)	107 (18.6%)	23 (11.9%)	19 (9.7%)	32 (18.5%)	

Missing	29 (7.9%)	6 (1.0%)	0 (0.0%)	35 (15.2%)	2 (1.1%)	
Bone metastases						<0.001**
0 (No)	243 (72.3%)	330 (57.5%)	131 (67.5%)	133 (68.2%)	114 (65.9%)	
1 (Yes)	93 (27.7%)	244 (42.5%)	63 (32.5%)	62 (31.8%)	59 (34.1%)	
Missing	29 (7.9%)	6 (1.0%)	0 (0.0%)	35 (15.2%)	2 (1.1%)	
LDH (U/L)	217.5 [182.0–287.0]	313.0 [229.0–406.0]	214.0 [176.0–296.0]	238.5 [194.0–322.2]	390.0 [306.0–531.0]	<0.001*
Missing	111 (30.4%)	109 (18.8%)	115 (59.3%)	40 (17.4%)	118 (67.4%)	
NLR	5.9 [3.9–9.1]	4.6 [3.1–7.5]	3.2 [2.4–5.4]	4.0 [2.8–7.0]	4.5 [3.2–7.6]	<0.001*
Missing	34 (9.3%)	46 (7.9%)	30 (15.5%)	24 (10.4%)	43 (24.6%)	
ECOG PS						<0.001***
0	75 (23.7%)	167 (28.9%)	123 (63.4%)	67 (50.8%)	37 (22.0%)	
1	178 (56.2%)	325 (56.3%)	60 (30.9%)	52 (39.4%)	110 (65.5%)	
2	57 (18.0%)	83 (14.4%)	11 (5.7%)	12 (9.1%)	21 (12.5%)	
3	6 (1.9%)	2 (0.3%)	0 (0.0%)	0 (0.0%)	0 (0.0%)	
4	1 (0.3%)	0 (0.0%)	0 (0.0%)	1 (0.8%)	0 (0.0%)	
Missing	48 (13.2%)	3 (0.5%)	0 (0.0%)	98 (42.6%)	7 (4.0%)	
PD-L1 category						<0.001**
<1%	60 (24.6%)	172 (33.8%)	73 (41.0%)	69 (36.1%)	54 (50.5%)	
1-49%	80 (32.8%)	184 (36.1%)	61 (34.3%)	34 (17.8%)	20 (18.7%)	
≥ 50%	104 (42.6%)	153 (30.1%)	44 (24.7%)	88 (46.1%)	33 (30.8%)	
Missing	121 (33.2%)	71 (12.2%)	16 (8.2%)	39 (17.0%)	68 (38.9%)	

Table 14. Clinical characteristics of patients in Test set for DCR across centers (GHD, INT, MH, SZMC and VHIO), including p-value for comparison across centers. * p-value of Kruskal-Wallis Test for Medians; ** p-value of Chi-Square Test; *** p-value of Fisher's Exact Test.

Variable	GHD (n=65)	INT (n=101)	MH (n=35)	SZMC (n=41)	VHIO (n=31)	p-value
Sex						0.274**
Female	27 (41.5%)	41 (40.6%)	9 (25.7%)	13 (31.7%)	8 (25.8%)	
Male	38 (58.5%)	60 (59.4%)	26 (74.3%)	28 (68.3%)	23 (74.2%)	
Missing	0 (0.0%)	0 (0.0%)	0 (0.0%)	0 (0.0%)	0 (0.0%)	
Smoking – Current						0.010**
0 (No)	25 (47.2%)	68 (72.3%)	19 (55.9%)	19 (47.5%)	21 (67.7%)	
1 (Yes)	28 (52.8%)	26 (27.7%)	15 (44.1%)	21 (52.5%)	10 (32.3%)	
Missing	12 (18.5%)	7 (6.9%)	1 (2.9%)	1 (2.4%)	0 (0.0%)	
Smoking – Former						0.004**
0 (No)	32 (60.4%)	32 (34.0%)	16 (47.1%)	26 (65.0%)	13 (41.9%)	
1 (Yes)	21 (39.6%)	62 (66.0%)	18 (52.9%)	14 (35.0%)	18 (58.1%)	
Missing	12 (18.5%)	7 (6.9%)	1 (2.9%)	1 (2.4%)	0 (0.0%)	
Smoking – Never						<0.001***
0 (No)	49 (92.5%)	88 (93.6%)	33 (97.1%)	35 (87.5%)	28 (90.3%)	
1 (Yes)	4 (7.5%)	6 (6.4%)	1 (2.9%)	5 (12.5%)	3 (9.7%)	
Missing	12 (18.5%)	7 (6.9%)	1 (2.9%)	1 (2.4%)	0 (0.0%)	
Brain metastases						0.528**
0 (No)	54 (85.7%)	74 (74.7%)	26 (76.5%)	32 (82.1%)	24 (77.4%)	

1 (Yes)	9 (14.3%)	25 (25.3%)	8 (23.5%)	7 (17.9%)	7 (22.6%)	
Missing	2 (3.1%)	2 (2.0%)	1 (2.9%)	2 (4.9%)	0 (0.0%)	
Liver metastases						0.297**
0 (No)	50 (79.4%)	79 (79.8%)	28 (82.4%)	36 (92.3%)	28 (90.3%)	
1 (Yes)	13 (20.6%)	20 (20.2%)	6 (17.6%)	3 (7.7%)	3 (9.7%)	
Missing	2 (3.1%)	2 (2.0%)	1 (2.9%)	2 (4.9%)	0 (0.0%)	
Bone metastases						0.053**
0 (No)	44 (69.8%)	53 (53.5%)	26 (76.5%)	28 (71.8%)	18 (58.1%)	
1 (Yes)	19 (30.2%)	46 (46.5%)	8 (23.5%)	11 (28.2%)	13 (41.9%)	
Missing	2 (3.1%)	2 (2.0%)	1 (2.9%)	2 (4.9%)	0 (0.0%)	
LDH (U/L)	219.0 [183.8–316.8]	342.0 [263.5–416.5]	207.0 [174.0–254.0]	219.0 [193.5–251.5]	387.0 [264.8–451.8]	<0.001*
Missing	19 (29.2%)	22 (21.8%)	20 (57.1%)	6 (14.6%)	23 (74.2%)	
NLR	5.1 [3.4–8.1]	4.8 [3.5–7.0]	3.1 [2.1–4.5]	3.7 [2.6–5.7]	4.5 [3.0–7.5]	0.012*
Missing	5 (7.7%)	8 (7.9%)	8 (22.9%)	2 (4.9%)	8 (25.8%)	
ECOG PS						<0.001***
0	20 (35.7%)	25 (25.0%)	24 (70.6%)	14 (51.9%)	7 (22.6%)	
1	25 (44.6%)	50 (50.0%)	9 (26.5%)	11 (40.7%)	20 (64.5%)	
2	7 (12.5%)	23 (23.0%)	1 (2.9%)	2 (7.4%)	4 (12.9%)	
3	4 (7.1%)	2 (2.0%)	0 (0.0%)	0 (0.0%)	0 (0.0%)	
4	0 (0.0%)	0 (0.0%)	0 (0.0%)	0 (0.0%)	0 (0.0%)	
Missing	9 (13.8%)	1 (1.0%)	1 (2.9%)	14 (34.1%)	0 (0.0%)	
PD-L1 category						0.007**
<1%	9 (20.5%)	26 (31.7%)	17 (53.1%)	14 (41.2%)	9 (40.9%)	
1-49%	21 (47.7%)	34 (41.5%)	10 (31.2%)	6 (17.6%)	3 (13.6%)	
≥ 50%	14 (31.8%)	22 (26.8%)	5 (15.6%)	14 (41.2%)	10 (45.5%)	
Missing	21 (32.3%)	19 (18.8%)	3 (8.6%)	7 (17.1%)	9 (29.0%)	

Table 15. Values of genomic data in C23, including percentages of patients with no driver mutation reported, with driver alterations and with missing information for genes EGFR, ALK, ROS, RET

Gene	No driver mutation reported	Driver alteration percentage	Missing information percentage
EGFR	77.4%	1.7%	20.9%
ALK	77.2%	0.3%	22.5%
ROS	69.1%	0.1%	30.8%
RET	54.4%	0.5%	45.1%

Table 16. Statistical comparison of feature distributions between patients with complete multimodal data vs incomplete data, for CB RAD in the Train set. * p-value of Mann-Whitney U Test; ** p-value of Chi-Square Test; *** p-value of Fisher's Exact Test.

Variable	Complete (n=642)	Incomplete (n=908)	p-value
Sex			0.081**
Female	239 (37.2%)	298 (32.8%)	
Male	403 (62.8%)	610 (67.2%)	

Missing	0 (0.0%)	0 (0.0%)	
Smoking – Current			0.753**
0 (No)	353 (61.3%)	518 (62.3%)	
1 (Yes)	223 (38.7%)	314 (37.7%)	
Missing	66 (10.3%)	76 (8.4%)	
Smoking – Former			0.832**
0 (No)	270 (46.9%)	384 (46.2%)	
1 (Yes)	306 (53.1%)	448 (53.8%)	
Missing	66 (10.3%)	76 (8.4%)	
Smoking – Never			0.943**
0 (No)	529 (91.8%)	762 (91.6%)	
1 (Yes)	47 (8.2%)	70 (8.4%)	
Missing	66 (10.3%)	76 (8.4%)	
Brain metastases			0.233**
0 (No)	469 (78.2%)	705 (80.8%)	
1 (Yes)	131 (21.8%)	167 (19.2%)	
Missing	42 (6.5%)	36 (4.0%)	
Liver metastases			0.518**
0 (No)	498 (83.0%)	736 (84.4%)	
1 (Yes)	102 (17.0%)	136 (15.6%)	
Missing	42 (6.5%)	36 (4.0%)	
Bone metastases			0.446**
0 (No)	395 (65.8%)	556 (63.8%)	
1 (Yes)	205 (34.2%)	316 (36.2%)	
Missing	42 (6.5%)	36 (4.0%)	
LDH (U/L)	283.0 [203.0–363.0]	264.0 [196.0–383.0]	0.333*
Missing	159 (24.8%)	339 (37.3%)	
NLR	4.6 [3.2–7.8]	4.6 [2.9–7.7]	0.390*
Missing	40 (6.2%)	142 (15.6%)	
ECOG PS			<0.001***
0	167 (29.3%)	303 (37.0%)	
1	326 (57.3%)	399 (48.7%)	
2	70 (12.3%)	114 (13.9%)	
3	6 (1.1%)	2 (0.2%)	
4	0 (0.0%)	2 (0.2%)	
Missing	73 (11.4%)	88 (9.7%)	
PD-L1 category			0.739**
0	174 (36.0%)	254 (33.8%)	
1	148 (30.6%)	235 (31.3%)	
2	162 (33.5%)	262 (34.9%)	
Missing	158 (24.6%)	157 (17.3%)	

Table 17. Statistical comparison of feature distributions between patients with complete multimodal data vs incomplete data, for CB RAD in the Test set. * p-value of Mann-Whitney U Test; ** p-value of Chi-Square Test; *** p-value of Fisher's Exact Test.

Variable	Complete (n=116)	Incomplete (n=158)	p-value
Sex			0.361**
Female	46 (39.7%)	53 (33.5%)	
Male	70 (60.3%)	105 (66.5%)	
Missing	0 (0.0%)	0 (0.0%)	
Smoking – Current			0.791**
(No)	64 (61.5%)	88 (59.1%)	
(Yes)	40 (38.5%)	61 (40.9%)	
Missing	12 (10.3%)	9 (5.7%)	
Smoking – Former			0.965**
0 (No)	50 (48.1%)	70 (47.0%)	
1 (Yes)	54 (51.9%)	79 (53.0%)	
Missing	12 (10.3%)	9 (5.7%)	
Smoking – Never			0.413**
0 (No)	94 (90.4%)	140 (94.0%)	
1 (Yes)	10 (9.6%)	9 (6.0%)	
Missing	12 (10.3%)	9 (5.7%)	
Brain metastases			0.695**
0 (No)	91 (80.5%)	119 (77.8%)	
1 (Yes)	22 (19.5%)	34 (22.2%)	
Missing	3 (2.6%)	5 (3.2%)	
Liver metastases			1.000**
0 (No)	94 (83.2%)	127 (83.0%)	
1 (Yes)	19 (16.8%)	26 (17.0%)	
Missing	3 (2.6%)	5 (3.2%)	
Bone metastases			0.485**
0 (No)	75 (66.4%)	94 (61.4%)	
1 (Yes)	38 (33.6%)	59 (38.6%)	
Missing	3 (2.6%)	5 (3.2%)	
LDH (U/L)	312.0 [219.0–409.0]	240.0 [186.0–333.0]	<0.001*
Missing	34 (29.3%)	57 (36.1%)	
NLR	4.7 [3.2–7.7]	4.3 [2.6–6.3]	0.070*
Missing	8 (6.9%)	24 (15.2%)	
ECOG PS			<0.001***
0	30 (28.6%)	60 (42.0%)	
1	55 (52.4%)	60 (42.0%)	
2	17 (16.2%)	20 (14.0%)	
3	3 (2.9%)	3 (2.1%)	
4	0 (0.0%)	0 (0.0%)	
Missing	11 (9.5%)	15 (9.5%)	
PD-L1 category			0.595**
0	28 (32.2%)	47 (36.7%)	
1	29 (33.3%)	45 (35.2%)	
2	30 (34.5%)	36 (28.1%)	
Missing	29 (25.0%)	30 (19.0%)	

Table 18. Statistical comparison of feature distributions between patients with complete multimodal data vs incomplete data, for CB RAD in ExVal. * p-value of Mann-Whitney U Test; ** p-value of Chi-Square Test; *** p-value of Fisher's Exact Test.

Variable	Complete (n=138)	Incomplete (n=113)	p-value
Sex			0.583**
Female	84 (60.9%)	64 (56.6%)	
Male	54 (39.1%)	49 (43.4%)	
Missing	0 (0.0%)	0 (0.0%)	

Smoking – Current			0.155**
0 (No)	112 (81.2%)	100 (88.5%)	
1 (Yes)	26 (18.8%)	13 (11.5%)	
Missing	0 (0.0%)	0 (0.0%)	
Smoking – Former			0.478**
0 (No)	37 (26.8%)	25 (22.1%)	
1 (Yes)	101 (73.2%)	88 (77.9%)	
Missing	0 (0.0%)	0 (0.0%)	
Smoking – Never			0.614**
0 (No)	127 (92.0%)	101 (89.4%)	
1 (Yes)	11 (8.0%)	12 (10.6%)	
Missing	0 (0.0%)	0 (0.0%)	
Brain metastases			0.770**
0 (No)	88 (77.9%)	76 (75.2%)	
1 (Yes)	25 (22.1%)	25 (24.8%)	
Missing	25 (18.1%)	12 (10.6%)	
Liver metastases			1.000**
0 (No)	95 (84.1%)	84 (83.2%)	
1 (Yes)	18 (15.9%)	17 (16.8%)	
Missing	25 (18.1%)	12 (10.6%)	
Bone metastases			0.452**
0 (No)	76 (67.3%)	62 (61.4%)	
1 (Yes)	37 (32.7%)	39 (38.6%)	
Missing	25 (18.1%)	12 (10.6%)	
LDH (U/L)	294.0 [199.0–341.0]	210.0 [172.8–251.8]	0.035*
Missing	121 (87.7%)	95 (84.1%)	
NLR	4.2 [2.6–7.2]	4.8 [2.6–9.4]	0.391*
Missing	0 (0.0%)	0 (0.0%)	
ECOG PS			<0.001***
0	2 (1.5%)	4 (3.6%)	
1	109 (79.6%)	88 (79.3%)	
2	24 (17.5%)	17 (15.3%)	
3	2 (1.5%)	2 (1.8%)	
4	0 (0.0%)	0 (0.0%)	
Missing	1 (0.7%)	2 (1.8%)	
PD-L1 category			0.151**
0	59 (49.2%)	42 (42.0%)	
1	30 (25.0%)	20 (20.0%)	
2	31 (25.8%)	38 (38.0%)	
Missing	18 (13.0%)	13 (11.5%)	

Table 19. Statistical comparison of feature distributions between patients with complete multimodal data vs incomplete data, for CB DP in Train set. * p-value of Mann-Whitney U Test; ** p-value of Chi-Square Test; *** p-value of Fisher's Exact Test.

Variable	Complete (n=605)	Incomplete (n=945)	p-value
Sex			0.577**
Female	204 (33.7%)	333 (35.2%)	
Male	401 (66.3%)	612 (64.8%)	
Missing	0 (0.0%)	0 (0.0%)	
Smoking – Current			0.334**
0 (No)	363 (63.5%)	508 (60.8%)	

1 (Yes)	209 (36.5%)	328 (39.2%)	
Missing	33 (5.5%)	109 (11.5%)	
Smoking – Former			0.729**
0 (No)	262 (45.8%)	392 (46.9%)	
1 (Yes)	310 (54.2%)	444 (53.1%)	
Missing	33 (5.5%)	109 (11.5%)	
Smoking – Never			0.329**
0 (No)	519 (90.7%)	772 (92.3%)	
1 (Yes)	53 (9.3%)	64 (7.7%)	
Missing	33 (5.5%)	109 (11.5%)	
Brain metastases			0.450**
0 (No)	454 (78.7%)	720 (80.4%)	
1 (Yes)	123 (21.3%)	175 (19.6%)	
Missing	28 (4.6%)	50 (5.3%)	
Liver metastases			0.077**
0 (No)	471 (81.6%)	763 (85.3%)	
1 (Yes)	106 (18.4%)	132 (14.7%)	
Missing	28 (4.6%)	50 (5.3%)	
Bone metastases			0.018**
0 (No)	351 (60.8%)	600 (67.0%)	
1 (Yes)	226 (39.2%)	295 (33.0%)	
Missing	28 (4.6%)	50 (5.3%)	
LDH (U/L)	272.0 [194.0–375.0]	271.0 [202.0–377.0]	0.649*
Missing	174 (28.8%)	324 (34.3%)	
NLR	4.6 [3.0–7.9]	4.6 [3.0–7.6]	0.595*
Missing	46 (7.6%)	136 (14.4%)	
ECOG PS			<0.001***
0	152 (28.0%)	318 (37.6%)	
1	315 (58.0%)	410 (48.5%)	
2	75 (13.8%)	109 (12.9%)	
3	0 (0.0%)	8 (0.9%)	
4	1 (0.2%)	1 (0.1%)	
Missing	62 (10.2%)	99 (10.5%)	
PD-L1 category			0.020**
0	172 (32.6%)	256 (36.2%)	
1	151 (28.7%)	232 (32.8%)	
2	204 (38.7%)	220 (31.1%)	
Missing	78 (12.9%)	237 (25.1%)	

Table 20. Statistical comparison of feature distributions between patients with complete multimodal data vs incomplete data, for CB DP in Test set. * p-value of Mann-Whitney U Test; ** p-value of Chi-Square Test; *** p-value of Fisher's Exact Test.

Variable	Complete (n=98)	Incomplete (n=176)	p-value
Sex			0.617**
Female	33 (33.7%)	66 (37.5%)	
Male	65 (66.3%)	110 (62.5%)	
Missing	0 (0.0%)	0 (0.0%)	
Smoking – Current			0.837**
0 (No)	54 (58.7%)	98 (60.9%)	
1 (Yes)	38 (41.3%)	63 (39.1%)	
Missing	6 (6.1%)	15 (8.5%)	
Smoking – Former			0.821**
0 (No)	45 (48.9%)	75 (46.6%)	

1 (Yes)	47 (51.1%)	86 (53.4%)	
Missing	6 (6.1%)	15 (8.5%)	
Smoking – Never			1.000**
0 (No)	85 (92.4%)	149 (92.5%)	
1 (Yes)	7 (7.6%)	12 (7.5%)	
Missing	6 (6.1%)	15 (8.5%)	
Brain metastases			0.471**
0 (No)	77 (81.9%)	133 (77.3%)	
1 (Yes)	17 (18.1%)	39 (22.7%)	
Missing	4 (4.1%)	4 (2.3%)	
Liver metastases			0.838**
0 (No)	77 (81.9%)	144 (83.7%)	
1 (Yes)	17 (18.1%)	28 (16.3%)	
Missing	4 (4.1%)	4 (2.3%)	
Bone metastases			0.097**
0 (No)	53 (56.4%)	116 (67.4%)	
1 (Yes)	41 (43.6%)	56 (32.6%)	
Missing	4 (4.1%)	4 (2.3%)	
LDH (U/L)	263.5 [201.8–385.2]	260.0 [202.0–364.0]	0.822*
Missing	32 (32.7%)	59 (33.5%)	
NLR	4.4 [3.3–6.8]	4.4 [2.6–7.1]	0.341*
Missing	8 (8.2%)	24 (13.6%)	
ECOG PS			<0.001***
0	25 (28.7%)	65 (40.4%)	
1	44 (50.6%)	71 (44.1%)	
2	18 (20.7%)	19 (11.8%)	
3	0 (0.0%)	6 (3.7%)	
4	0 (0.0%)	0 (0.0%)	
Missing	11 (11.2%)	15 (8.5%)	
PD-L1 category			0.074**
0	22 (25.9%)	53 (40.8%)	
1	32 (37.6%)	42 (32.3%)	
2	31 (36.5%)	35 (26.9%)	
Missing	13 (13.3%)	46 (26.1%)	

Table 21. Statistical comparison of feature distributions between patients with complete multimodal data vs incomplete data, for CB DP in ExVal. * p-value of Mann-Whitney U Test; ** p-value of Chi-Square Test; *** p-value of Fisher's Exact Test.

Variable	Complete (n=143)	Incomplete (n=108)	p-value
Sex			0.410**
Female	88 (61.5%)	60 (55.6%)	
Male	55 (38.5%)	48 (44.4%)	
Missing	0 (0.0%)	0 (0.0%)	
Smoking – Current			0.921**
0 (No)	120 (83.9%)	92 (85.2%)	
1 (Yes)	23 (16.1%)	16 (14.8%)	
Missing	0 (0.0%)	0 (0.0%)	
Smoking – Former			0.348**
0 (No)	39 (27.3%)	23 (21.3%)	
1 (Yes)	104 (72.7%)	85 (78.7%)	
Missing	0 (0.0%)	0 (0.0%)	
Smoking – Never			0.290**
0 (No)	127 (88.8%)	101 (93.5%)	
1 (Yes)	16 (11.2%)	7 (6.5%)	
Missing	0 (0.0%)	0 (0.0%)	
Brain metastases			0.381**
0 (No)	88 (73.9%)	76 (80.0%)	

1 (Yes)	31 (26.1%)	19 (20.0%)	
Missing	24 (16.8%)	13 (12.0%)	
Liver metastases			1.000**
0 (No)	100 (84.0%)	79 (83.2%)	
1 (Yes)	19 (16.0%)	16 (16.8%)	
Missing	24 (16.8%)	13 (12.0%)	
Bone metastases			0.722**
0 (No)	75 (63.0%)	63 (66.3%)	
1 (Yes)	44 (37.0%)	32 (33.7%)	
Missing	24 (16.8%)	13 (12.0%)	
LDH (U/L)	238.0 [150.0–309.0]	242.0 [188.8–281.5]	0.920*
Missing	122 (85.3%)	94 (87.0%)	
NLR	4.7 [2.5–8.4]	4.4 [2.7–7.4]	0.741*
Missing	0 (0.0%)	0 (0.0%)	
ECOG PS			<0.001***
0	5 (3.5%)	1 (0.9%)	
1	113 (80.1%)	84 (78.5%)	
2	21 (14.9%)	20 (18.7%)	
3	2 (1.4%)	2 (1.9%)	
4	0 (0.0%)	0 (0.0%)	
Missing	2 (1.4%)	1 (0.9%)	
PD-L1 category			0.272**
0	54 (41.5%)	47 (52.2%)	
1	33 (25.4%)	17 (18.9%)	
2	43 (33.1%)	26 (28.9%)	
Missing	13 (9.1%)	18 (16.7%)	

Table 22. Statistical comparison of feature distributions between patients with complete multimodal data vs incomplete data, for CB DP RAD in the Train set. * p-value of Mann-Whitney U Test; ** p-value of Chi-Square Test; *** p-value of Fisher's Exact Test.

Variable	Complete (n=269)	Incomplete (n=1281)	p-value
Sex			0.455**
Female	99 (36.8%)	438 (34.2%)	
Male	170 (63.2%)	843 (65.8%)	
Missing	0 (0.0%)	0 (0.0%)	
Smoking – Current			0.016**
0 (No)	179 (68.6%)	692 (60.3%)	
1 (Yes)	82 (31.4%)	455 (39.7%)	
Missing	8 (3.0%)	134 (10.5%)	
Smoking – Former			0.080**
0 (No)	108 (41.4%)	546 (47.6%)	
1 (Yes)	153 (58.6%)	601 (52.4%)	
Missing	8 (3.0%)	134 (10.5%)	
Smoking – Never			0.344**
0 (No)	235 (90.0%)	1056 (92.1%)	
1 (Yes)	26 (10.0%)	91 (7.9%)	
Missing	8 (3.0%)	134 (10.5%)	
Brain metastases			0.128**
0 (No)	194 (76.1%)	980 (80.5%)	
1 (Yes)	61 (23.9%)	237 (19.5%)	
Missing	14 (5.2%)	64 (5.0%)	
Liver metastases			0.541**
0 (No)	210 (82.4%)	1024 (84.1%)	
1 (Yes)	45 (17.6%)	193 (15.9%)	

Missing	14 (5.2%)	64 (5.0%)	
Bone metastases			0.297**
0 (No)	157 (61.6%)	794 (65.2%)	
1 (Yes)	98 (38.4%)	423 (34.8%)	
Missing	14 (5.2%)	64 (5.0%)	
LDH (U/L)	279.0 [199.5–355.5]	271.0 [200.0–378.0]	0.924*
Missing	66 (24.5%)	432 (33.7%)	
NLR	4.5 [3.1–7.7]	4.6 [3.0–7.8]	0.938*
Missing	15 (5.6%)	167 (13.0%)	
ECOG PS			<0.001***
0	60 (25.0%)	410 (35.7%)	
1	152 (63.3%)	573 (49.9%)	
2	28 (11.7%)	156 (13.6%)	
3	0 (0.0%)	8 (0.7%)	
4	0 (0.0%)	2 (0.2%)	
Missing	29 (10.8%)	132 (10.3%)	
PD-L1 category			0.353**
0	79 (32.5%)	349 (35.2%)	
1	71 (29.2%)	312 (31.5%)	
2	93 (38.3%)	331 (33.4%)	
Missing	26 (9.7%)	289 (22.6%)	

Table 23. Statistical comparison of feature distributions between patients with complete multimodal data vs incomplete data, for CB DP RAD in the Test set. * p-value of Mann-Whitney U Test; ** p-value of Chi-Square Test; *** p-value of Fisher's Exact Test.

Variable	Complete (n=47)	Incomplete (n=227)	p-value
Sex			0.613**
Female	19 (40.4%)	80 (35.2%)	
Male	28 (59.6%)	147 (64.8%)	
Missing	0 (0.0%)	0 (0.0%)	
Smoking – Current			1.000**
0 (No)	27 (60.0%)	125 (60.1%)	
1 (Yes)	18 (40.0%)	83 (39.9%)	
Missing	2 (4.3%)	19 (8.4%)	
Smoking – Former			0.959**
0 (No)	22 (48.9%)	98 (47.1%)	
1 (Yes)	23 (51.1%)	110 (52.9%)	
Missing	2 (4.3%)	19 (8.4%)	
Smoking – Never			0.754***
0 (No)	41 (91.1%)	193 (92.8%)	
1 (Yes)	4 (8.9%)	15 (7.2%)	
Missing	2 (4.3%)	19 (8.4%)	
Brain metastases			0.638**
0 (No)	38 (82.6%)	172 (78.2%)	
1 (Yes)	8 (17.4%)	48 (21.8%)	
Missing	1 (2.1%)	7 (3.1%)	
Liver metastases			0.579**
0 (No)	40 (87.0%)	181 (82.3%)	
1 (Yes)	6 (13.0%)	39 (17.7%)	
Missing	1 (2.1%)	7 (3.1%)	
Bone metastases			1.000**
0 (No)	29 (63.0%)	140 (63.6%)	
1 (Yes)	17 (37.0%)	80 (36.4%)	

Missing	1 (2.1%)	7 (3.1%)	
LDH (U/L)	285.0 [204.0–388.5]	259.5 [202.0–364.2]	0.973*
Missing	16 (34.0%)	75 (33.0%)	
NLR	4.4 [3.3–7.6]	4.4 [2.7–6.8]	0.295*
Missing	4 (8.5%)	28 (12.3%)	
ECOG PS			<0.001***
0	11 (26.2%)	79 (38.3%)	
1	24 (57.1%)	91 (44.2%)	
2	7 (16.7%)	30 (14.6%)	
3	0 (0.0%)	6 (2.9%)	
4	0 (0.0%)	0 (0.0%)	
Missing	5 (10.6%)	21 (9.3%)	
PD-L1 category			0.251**
0	12 (29.3%)	63 (36.2%)	
1	12 (29.3%)	62 (35.6%)	
2	17 (41.5%)	49 (28.2%)	
Missing	6 (12.8%)	53 (23.3%)	

Table 24. Statistical comparison of feature distributions between patients with complete multimodal data vs incomplete data, for CB DP RAD in ExVal. * p-value of Mann-Whitney U Test; ** p-value of Chi-Square Test; *** p-value of Fisher's Exact Test.

Variable	Complete (n=84)	Incomplete (n=167)	p-value
Sex			1.000**
Female	50 (59.5%)	98 (58.7%)	
Male	34 (40.5%)	69 (41.3%)	
Missing	0 (0.0%)	0 (0.0%)	
Smoking – Current			0.100**
0 (No)	66 (78.6%)	146 (87.4%)	
1 (Yes)	18 (21.4%)	21 (12.6%)	
Missing	0 (0.0%)	0 (0.0%)	
Smoking – Former			0.141**
0 (No)	26 (31.0%)	36 (21.6%)	
1 (Yes)	58 (69.0%)	131 (78.4%)	
Missing	0 (0.0%)	0 (0.0%)	
Smoking – Never			1.000**
0 (No)	76 (90.5%)	152 (91.0%)	
1 (Yes)	8 (9.5%)	15 (9.0%)	
Missing	0 (0.0%)	0 (0.0%)	
Brain metastases			0.832**
0 (No)	51 (75.0%)	113 (77.4%)	
1 (Yes)	17 (25.0%)	33 (22.6%)	
Missing	16 (19.0%)	21 (12.6%)	
Liver metastases			1.000**
0 (No)	57 (83.8%)	122 (83.6%)	
1 (Yes)	11 (16.2%)	24 (16.4%)	
Missing	16 (19.0%)	21 (12.6%)	
Bone metastases			0.842**
0 (No)	45 (66.2%)	93 (63.7%)	
1 (Yes)	23 (33.8%)	53 (36.3%)	
Missing	16 (19.0%)	21 (12.6%)	
LDH (U/L)	294.0 [217.5–334.5]	224.5 [176.2–278.5]	0.183*
Missing	73 (86.9%)	143 (85.6%)	
NLR	4.3 [2.5–7.4]	4.6 [2.7–8.3]	0.702*

Missing	0 (0.0%)	0 (0.0%)	
ECOG PS			<0.001***
0	2 (2.4%)	4 (2.4%)	
1	67 (80.7%)	130 (78.8%)	
2	13 (15.7%)	28 (17.0%)	
3	1 (1.2%)	3 (1.8%)	
4	0 (0.0%)	0 (0.0%)	
Missing	1 (1.2%)	2 (1.2%)	
PD-L1 category			0.414**
0	37 (48.1%)	64 (44.8%)	
1	20 (26.0%)	30 (21.0%)	
2	20 (26.0%)	49 (34.3%)	
Missing	7 (8.3%)	24 (14.4%)	

Table 25. Statistical comparison of feature distributions between patients with complete multimodal data vs incomplete data, for CB DP RAD G in the Train set. * p-value of Mann-Whitney U Test; ** p-value of Chi-Square Test; *** p-value of Fisher's Exact Test.

Variable	Complete (n=216)	Incomplete (n=1334)	p-value
Sex			0.918**
Female	76 (35.2%)	461 (34.6%)	
Male	140 (64.8%)	873 (65.4%)	
Missing	0 (0.0%)	0 (0.0%)	
Smoking – Current			0.007**
0 (No)	150 (70.4%)	721 (60.3%)	
1 (Yes)	63 (29.6%)	474 (39.7%)	
Missing	3 (1.4%)	139 (10.4%)	
Smoking – Former			0.031**
0 (No)	84 (39.4%)	570 (47.7%)	
1 (Yes)	129 (60.6%)	625 (52.3%)	
Missing	3 (1.4%)	139 (10.4%)	
Smoking – Never			0.451**
0 (No)	192 (90.1%)	1099 (92.0%)	
1 (Yes)	21 (9.9%)	96 (8.0%)	
Missing	3 (1.4%)	139 (10.4%)	
Brain metastases			0.623**
0 (No)	158 (78.2%)	1016 (80.0%)	
1 (Yes)	44 (21.8%)	254 (20.0%)	
Missing	14 (6.5%)	64 (4.8%)	
Liver metastases			0.974**
0 (No)	170 (84.2%)	1064 (83.8%)	
1 (Yes)	32 (15.8%)	206 (16.2%)	
Missing	14 (6.5%)	64 (4.8%)	
Bone metastases			0.526**
0 (No)	126 (62.4%)	825 (65.0%)	
1 (Yes)	76 (37.6%)	445 (35.0%)	
Missing	14 (6.5%)	64 (4.8%)	
LDH (U/L)	281.0 [200.5–354.2]	269.5 [198.5–377.8]	0.898*
Missing	42 (19.4%)	456 (34.2%)	
NLR	4.3 [2.9–7.5]	4.6 [3.0–7.9]	0.389*
Missing	10 (4.6%)	172 (12.9%)	
ECOG PS			<0.001***
0	52 (27.1%)	418 (34.9%)	
1	119 (62.0%)	606 (50.6%)	

2	21 (10.9%)	163 (13.6%)	
3	0 (0.0%)	8 (0.7%)	
4	0 (0.0%)	2 (0.2%)	
Missing	24 (11.1%)	137 (10.3%)	
PD-L1 category			0.518**
0	65 (32.8%)	363 (35.0%)	
1	58 (29.3%)	325 (31.3%)	
2	75 (37.9%)	349 (33.7%)	
Missing	18 (8.3%)	297 (22.3%)	

Table 26. Statistical comparison of feature distributions between patients with complete multimodal data vs incomplete data, for CB DP RAD G in the Test set. * p-value of Mann-Whitney U Test; ** p-value of Chi-Square Test; *** p-value of Fisher's Exact Test.

Variable	Complete (n=40)	Incomplete (n=234)	p-value
Sex			0.709**
Female	16 (40.0%)	83 (35.5%)	
Male	24 (60.0%)	151 (64.5%)	
Missing	0 (0.0%)	0 (0.0%)	
Smoking – Current			0.810**
0 (No)	24 (63.2%)	128 (59.5%)	
1 (Yes)	14 (36.8%)	87 (40.5%)	
Missing	2 (5.0%)	19 (8.1%)	
Smoking – Former			0.854**
0 (No)	17 (44.7%)	103 (47.9%)	
1 (Yes)	21 (55.3%)	112 (52.1%)	
Missing	2 (5.0%)	19 (8.1%)	
Smoking – Never			1.000***
0 (No)	35 (92.1%)	199 (92.6%)	
1 (Yes)	3 (7.9%)	16 (7.4%)	
Missing	2 (5.0%)	19 (8.1%)	
Brain metastases			0.763**
0 (No)	32 (82.1%)	178 (78.4%)	
1 (Yes)	7 (17.9%)	49 (21.6%)	
Missing	1 (2.5%)	7 (3.0%)	
Liver metastases			0.354***
0 (No)	35 (89.7%)	186 (81.9%)	
1 (Yes)	4 (10.3%)	41 (18.1%)	
Missing	1 (2.5%)	7 (3.0%)	
Bone metastases			0.920**
0 (No)	24 (61.5%)	145 (63.9%)	
1 (Yes)	15 (38.5%)	82 (36.1%)	
Missing	1 (2.5%)	7 (3.0%)	
LDH (U/L)	286.0 [214.0–391.0]	257.0 [202.0–363.5]	0.528*
Missing	11 (27.5%)	80 (34.2%)	
NLR	4.6 [3.2–7.5]	4.4 [2.8–6.8]	0.453*
Missing	2 (5.0%)	30 (12.8%)	
ECOG PS			<0.001***
0	11 (31.4%)	79 (37.1%)	
1	18 (51.4%)	97 (45.5%)	
2	6 (17.1%)	31 (14.6%)	
3	0 (0.0%)	6 (2.8%)	
4	0 (0.0%)	0 (0.0%)	
Missing	5 (12.5%)	21 (9.0%)	
PD-L1 category			0.505**

0	11 (30.6%)	64 (35.8%)	
1	11 (30.6%)	63 (35.2%)	
2	14 (38.9%)	52 (29.1%)	
Missing	4 (10.0%)	55 (23.5%)	

Table 27. Statistical comparison of feature distributions between patients with complete multimodal data vs incomplete data, for CB DP RAD G in ExVal. * p-value of Mann-Whitney U Test; ** p-value of Chi-Square Test; *** p-value of Fisher's Exact Test.

Variable	Complete (n=83)	Incomplete (n=168)	p-value
Sex			1.000**
Female	49 (59.0%)	99 (58.9%)	
Male	34 (41.0%)	69 (41.1%)	
Missing	0 (0.0%)	0 (0.0%)	
Smoking – Current			0.088**
0 (No)	65 (78.3%)	147 (87.5%)	
1 (Yes)	18 (21.7%)	21 (12.5%)	
Missing	0 (0.0%)	0 (0.0%)	
Smoking – Former			0.120**
0 (No)	26 (31.3%)	36 (21.4%)	
1 (Yes)	57 (68.7%)	132 (78.6%)	
Missing	0 (0.0%)	0 (0.0%)	
Smoking – Never			1.000**
0 (No)	75 (90.4%)	153 (91.1%)	
1 (Yes)	8 (9.6%)	15 (8.9%)	
Missing	0 (0.0%)	0 (0.0%)	
Brain metastases			0.768**
0 (No)	50 (74.6%)	114 (77.6%)	
1 (Yes)	17 (25.4%)	33 (22.4%)	
Missing	16 (19.3%)	21 (12.5%)	
Liver metastases			1.000**
0 (No)	56 (83.6%)	123 (83.7%)	
1 (Yes)	11 (16.4%)	24 (16.3%)	
Missing	16 (19.3%)	21 (12.5%)	
Bone metastases			0.690**
0 (No)	45 (67.2%)	93 (63.3%)	
1 (Yes)	22 (32.8%)	54 (36.7%)	
Missing	16 (19.3%)	21 (12.5%)	
LDH (U/L)	294.0 [217.5–334.5]	224.5 [176.2–278.5]	0.183*
Missing	72 (86.7%)	144 (85.7%)	
NLR	4.5 [2.5–7.6]	4.6 [2.7–8.2]	0.779*
Missing	0 (0.0%)	0 (0.0%)	
ECOG PS			<0.001***
0	2 (2.4%)	4 (2.4%)	
1	66 (80.5%)	131 (78.9%)	
2	13 (15.9%)	28 (16.9%)	
3	1 (1.2%)	3 (1.8%)	
4	0 (0.0%)	0 (0.0%)	
Missing	1 (1.2%)	2 (1.2%)	
PD-L1 category			0.437**
0	36 (47.4%)	65 (45.1%)	
1	20 (26.3%)	30 (20.8%)	
2	20 (26.3%)	49 (34.0%)	
Missing	7 (8.4%)	24 (14.3%)	

Table 28. Statistical comparison of feature distributions between patients with multimodal data, CB DP RAD G, in Train vs Test vs ExVal. *p-value of Kruskal-Wallis Test for Medians; ** p-value of Chi-Square Test; *** p-value of Fisher's Exact Test.

Variable	Train (n=216)	Test (n=40)	Ext. Validation (n=83)	p-value
Sex				<0.001**
Female	76 (35.2%)	16 (40.0%)	49 (59.0%)	
Male	140 (64.8%)	24 (60.0%)	34 (41.0%)	
Missing	0 (0.0%)	0 (0.0%)	0 (0.0%)	
Smoking – Current				0.191**
0 (No)	150 (70.4%)	24 (63.2%)	65 (78.3%)	
1 (Yes)	63 (29.6%)	14 (36.8%)	18 (21.7%)	
Missing	3 (1.4%)	2 (5.0%)	0 (0.0%)	
Smoking – Former				0.288**
0 (No)	84 (39.4%)	17 (44.7%)	26 (31.3%)	
1 (Yes)	129 (60.6%)	21 (55.3%)	57 (68.7%)	
Missing	3 (1.4%)	2 (5.0%)	0 (0.0%)	
Smoking – Never				0.930**
0 (No)	192 (90.1%)	35 (92.1%)	75 (90.4%)	
1 (Yes)	21 (9.9%)	3 (7.9%)	8 (9.6%)	
Missing	3 (1.4%)	2 (5.0%)	0 (0.0%)	
Brain metastases				0.664**
0 (No)	158 (78.2%)	32 (82.1%)	50 (74.6%)	
1 (Yes)	44 (21.8%)	7 (17.9%)	17 (25.4%)	
Missing	14 (6.5%)	1 (2.5%)	16 (19.3%)	
Liver metastases				0.645**
0 (No)	170 (84.2%)	35 (89.7%)	56 (83.6%)	
1 (Yes)	32 (15.8%)	4 (10.3%)	11 (16.4%)	
Missing	14 (6.5%)	1 (2.5%)	16 (19.3%)	
Bone metastases				0.757**
0 (No)	126 (62.4%)	24 (61.5%)	45 (67.2%)	
1 (Yes)	76 (37.6%)	15 (38.5%)	22 (32.8%)	
Missing	14 (6.5%)	1 (2.5%)	16 (19.3%)	
LDH (U/L)	281.0 [200.5–354.2]	286.0 [214.0–391.0]	294.0 [217.5–334.5]	0.842*
Missing	42 (19.4%)	11 (27.5%)	72 (86.7%)	
NLR	4.3 [2.9–7.5]	4.6 [3.2–7.5]	4.5 [2.5–7.6]	0.775*
Missing	10 (4.6%)	2 (5.0%)	0 (0.0%)	
ECOG PS				<0.001***
0	52 (27.1%)	11 (31.4%)	2 (2.4%)	
1	119 (62.0%)	18 (51.4%)	66 (80.5%)	
2	21 (10.9%)	6 (17.1%)	13 (15.9%)	
3	0 (0.0%)	0 (0.0%)	1 (1.2%)	
4	0 (0.0%)	0 (0.0%)	0 (0.0%)	
Missing	24 (11.1%)	5 (12.5%)	1 (1.2%)	
PD-L1 category				0.197**
0	65 (32.8%)	11 (30.6%)	36 (47.4%)	
1	58 (29.3%)	11 (30.6%)	20 (26.3%)	

2	75 (37.9%)	14 (38.9%)	20 (26.3%)	
Missing	18 (8.3%)	4 (10.0%)	7 (8.4%)	

Table 29. Comparison of C-LineI-CHT cohort with cohort 2: Features comparison. Continuous variables were tested by means of the Wilcoxon's Test. Categorical variables were tested by means of the Chi-Square or Fisher Test, as appropriate.

	Cohort 2 Set (n=1437)	C-LineI-CHT (n=210)	Overall (n=1647)
Sex - n (%)			
Female	542 (37.7)	81 (38.6)	623 (37.8)
Male	895 (62.3)	129 (61.4)	1024 (62.2)
P-value		0.8116	
Baseline ECOG Performance Status - n (%)			
0	409 (32.0)	86 (51.5)	495 (34.2)
1	697 (54.5)	76 (45.5)	773 (53.4)
2	164 (12.8)	5 (3.0)	169 (11.7)
3	9 (0.7)	0 (0.0)	9 (0.6)
4	1 (0.1)	0 (0.0)	1 (0.1)
Missing	157	43	200
p-value		<.0001	
Baseline LDH			
Mean (SD)	304.2 (260.1)	383.5 (184.9)	314.6 (252.9)
Median (Q1 - Q3)	243.0 (188.0-338.0)	333.0 (280.5-411.5)	259.0 (196.0-358.0)
Min - Max	0.0 - 5063.0	145.0 - 1284.0	0.0 - 5063.0
Missing	582	82	664
P-value		<.0001	
NLR			
Mean (SD)	7.2 (12.7)	5.4 (4.6)	7.0 (12.1)
Median (Q1 - Q3)	4.6 (2.9-7.9)	4.3 (2.9-5.7)	4.6 (2.9-7.7)
Min - Max	0.0 - 245.6	0.6 - 36.7	0.0 - 245.6
Missing	133	71	204
P-value		0.0507	
Smoking Status - n (%)			
Never	105 (7.8)	9 (4.5)	114 (7.4)
Former	748 (55.5)	131 (66.2)	879 (56.9)
Current	495 (36.7)	58 (29.3)	553 (35.8)
Missing	89	12	101
P-value		0.0137	
PD-L1 - n (%)			
< 1%	420 (32.7)	68 (45.0)	488 (34.0)
1-49%	369 (28.7)	66 (43.7)	435 (30.3)
≥ 50%	496 (38.6)	17 (11.3)	513 (35.7)
Missing	152	59	211
P-value		<.0001	
Parenchymal Brain as Metastatic Site - n (%)			
No	1057 (79.1)	180 (87.0)	1237 (80.1)
Yes	280 (20.9)	27 (13.0)	307 (19.9)
Missing	100	3	103
P-value		0.0081	
Liver as Metastatic Site - n (%)			
No	1133 (84.7)	186 (89.9)	1319 (85.4)

Yes	204 (15.3)	21 (10.1)	225 (14.6)
Missing	100	3	103
P-value		0.0524	
Bones as Metastatic Site - n (%)			
No	876 (65.5)	144 (69.6)	1020 (66.1)
Yes	461 (34.5)	63 (30.4)	524 (33.9)
Missing	100	3	103
P-value		0.2527	

Legend: N: number of patients. SD: Standard deviation. Q1: First quartile. Q3: Third quartile. Min-Max: Minimum-Maximum.

Table 30. Comparison of C-LineI-CHT cohort with cohort 2: Overall Survival Comparison. The log-rank test was employed to assess any differences in terms of Overall Survival.

	Cohort 2 Set (n=1437)	C-LineI-CHT (n=210)	Overall (n=1647)
**Censored - n (%)**	533 (37.1)	14 (6.7)	547 (33.2)
**Deaths - n (%)**	904 (62.9)	196 (93.3)	1100 (66.8)
**Kaplan-Meier estimate of OS (months) - n (%)**			
First quartile	5.6	11.3	6.1
Median [95% CI]	15.6 [14.0-17.0]	19.2 [16.9-23.0]	16.3 [15.1-17.3]
Third quartile	38.0	44.2	38.5
P-value			0.0548

Legend: N: Number of subjects. SD: standard deviation. Q1 - Q3: first – third quartile. Min - Max: minimum – maximum values. CI: confidence interval.

Table 31. Comparison of C-StageIII-CHT cohort with cohort 2 and 3: Features comparison. Continuous variables were tested by means of the Wilcoxon's Test. Categorical variables were tested by means of the Chi-Square or Fisher Test, as appropriate.

	Cohort 2-3 Set (n=2075)	C-StageIII-CHT (n=97)	p-value	Overall (n=2172)
Sex - n (%)			0.5873	
Female	784 (37.8)	34 (35.1)		818 (37.7)
Male	1291 (62.2)	63 (64.9)		1354 (62.3)
Baseline ECOG Performance Status - n (%)			0.0188	
0	566 (30.0)	38 (40.0)		604 (30.5)
1	1037 (55.0)	54 (56.8)		1091 (55.1)
2	262 (13.9)	3 (3.2)		265 (13.4)
3	18 (1.0)	0 (0.0)		18 (0.9)
4	2 (0.1)	0 (0.0)		2 (0.1)
Missing	190	2		192
Baseline LDH			0.6114	
Mean (SD)	325.2 (247.6)	282.3 (105.4)		322.4 (241.3)
Median (Q1 - Q3)	268.0 (199.0-372.0)	296.5 (194.0-339.0)		271.0 (199.0-368.5)
Min - Max	0.0 - 5063.0	91.0 - 656.0		0.0 - 5063.0
Missing	805	11		816
NLR			<.0001	
Mean (SD)	7.0 (11.3)	3.9 (2.3)		6.8 (11.1)
Median (Q1 - Q3)	4.5 (2.9-7.7)	3.2 (2.2-4.9)		4.5 (2.9-7.6)
Min - Max	0.0 - 245.6	1.1 - 12.4		0.0 - 245.6
Missing	214	7		221
Smoking Status - n (%)			<.0001	
Never	159 (8.3)	18 (21.4)		177 (8.9)

Former	1076 (56.3)	66 (78.6)		1142 (57.2)
Current	677 (35.4)	0 (0.0)		677 (33.9)
Missing	163	13		176
PD-L1 - n (%)			0.5876	
< 1%	604 (36.2)	17 (29.8)		621 (36.0)
1-49%	507 (30.4)	20 (35.1)		527 (30.5)
≥ 50%	559 (33.5)	20 (35.1)		579 (33.5)
Missing	405	40		445
Parenchymal Brain as Metastatic Site - n (%)			<.0001	
No	1548 (79.3)	97 (100.0)		1645 (80.3)
Yes	404 (20.7)	0 (0.0)		404 (19.7)
Missing	123	0		123
Liver as Metastatic Site - n (%)			<.0001	
No	1634 (83.7)	97 (100.0)		1731 (84.5)
Yes	318 (16.3)	0 (0.0)		318 (15.5)
Missing	123	0		123
Bones as Metastatic Site - n (%)			<.0001	
No	1258 (64.4)	97 (100.0)		1355 (66.1)
Yes	694 (35.6)	0 (0.0)		694 (33.9)
Missing	123	0		123

Legend: N: number of patients. SD: Standard deviation. Q1: First quartile. Q3: Third quartile. Min-Max: Minimum-Maximum.

Table 32. Comparison of C-StageIII-CHT cohort with cohort 2 and 3: Overall Survival Comparison. The log-rank test was employed to assess any differences in terms of Overall Survival.

	Cohort 2-3 Set (n=2075)	C-StageIII-CHT (n=97)	Overall (n=2172)
Censored - n (%)	620 (29.9)	52 (53.6)	672 (30.9)
Deaths - n (%)	1455 (70.1)	45 (46.4)	1500 (69.1)
Kaplan-Meier estimate of OS (months) - n (%)			
First quartile	4.7	7.9	4.9
Median [95% CI]	13.0 [12.0-14.0]	32.8 [18.8-87.6]	13.4 [12.4-14.6]
Third quartile	33.2	87.6	34.5
p-value			<.0001

Legend: N: Number of subjects. SD: standard deviation. Q1 - Q3: first – third quartile. Min - Max: minimum – maximum values. CI: confidence interval.

Table 33. Comparison of C-EGFR-INT cohort with cohort 2 and 3: Features comparison. Continuous variables were tested by means of the Wilcoxon's Test. Categorical variables were tested by means of the Chi-Square or Fisher Test, as appropriate.

	Cohort 2-3 Set (n=2075)	C-EGFR-INT (n=132)	p-value	Overall (n=2207)
Sex - n (%)			<.0001	
Female	784 (37.8)	89 (67.4)		873 (39.6)
Male	1291 (62.2)	43 (32.6)		1334 (60.4)
Baseline ECOG Performance Status - n (%)			0.6802	

0	566 (30.0)	44 (33.6)		610 (30.3)
1	1037 (55.0)	64 (48.9)		1101 (54.6)
2	262 (13.9)	21 (16.0)		283 (14.0)
3	18 (1.0)	2 (1.5)		20 (1.0)
4	2 (0.1)	0 (0.0)		2 (0.1)
Missing	190	1		191
Baseline LDH			0.0719	
Mean (SD)	325.2 (247.6)	343.7 (457.4)		326.6 (269.0)
Median (Q1 - Q3)	268.0 (199.0-372.0)	244.5 (184.0-356.0)		266.0 (197.0-372.0)
Min - Max	0.0 - 5063.0	86.0 - 4495.0		0.0 - 5063.0
Missing	805	28		833
NLR			0.5883	
Mean (SD)	7.0 (11.3)	7.7 (8.5)		7.0 (11.2)
Median (Q1 - Q3)	4.5 (2.9-7.7)	4.8 (2.8-8.5)		4.6 (2.9-7.7)
Min - Max	0.0 - 245.6	0.3 - 41.8		0.0 - 245.6
Missing	214	25		239
Smoking Status - n (%)			<.0001	
Never	159 (8.3)	25 (19.7)		184 (9.0)
Former	1076 (56.3)	64 (50.4)		1140 (55.9)
Current	677 (35.4)	38 (29.9)		715 (35.1)
Missing	163	5		168
PD-L1 - n (%)			0.0005	
< 1%	604 (36.2)	51 (44.7)		655 (36.7)
1-49%	507 (30.4)	15 (13.2)		522 (29.3)
≥ 50%	559 (33.5)	48 (42.1)		607 (34.0)
Missing	405	18		423
Parenchymal Brain as Metastatic Site - n (%)			0.0001	
No	1548 (79.3)	86 (65.2)		1634 (78.4)
Yes	404 (20.7)	46 (34.8)		450 (21.6)
Missing	123	0		123
Liver as Metastatic Site - n (%)			0.9100	
No	1634 (83.7)	110 (83.3)		1744 (83.7)
Yes	318 (16.3)	22 (16.7)		340 (16.3)
Missing	123	0		123
Bones as Metastatic Site - n (%)			<.0001	
No	1258 (64.4)	57 (43.2)		1315 (63.1)
Yes	694 (35.6)	75 (56.8)		769 (36.9)
Missing	123	0		123

Legend**:** N: number of patients. SD: Standard deviation. Q1: First quartile. Q3: Third quartile. Min-Max: Minimum-Maximum.

Table 34. Comparison of C-EGFR-INT cohort with cohort 2 and 3: Overall Survival Comparison. The log-rank test was employed to assess any differences in terms of Overall Survival.

	Cohort 2-3 Set (n=2075)	C-EGFR-INT (n=132)	Overall (n=2207)
Censored - n (%)	620 (29.9)	59 (44.7)	679 (30.8)
Deaths - n (%)	1455 (70.1)	73 (55.3)	1528 (69.2)
Kaplan-Meier estimate of OS (months) - n (%)			
First quartile	4.7	14.5	5.0
Median [95% CI]	13.0 [12.0-14.0]	25.3 [20.1-34.2]	13.8 [12.7-15.2]
Third quartile	33.2	57.5	34.5
Log-rank test, p-value			<.0001

Legend: N: Number of subjects. SD: standard deviation. Q1 - Q3: first – third quartile. Min - Max: minimum – maximum values. CI: confidence interval.

Table 35. Comparison of EGFR-UOC cohort with cohort 2 and 3: Continuous variables were tested by means of the Wilcoxon's Test. Categorical variables were tested by means of the Chi-Square or Fisher Test, as appropriate.

	Cohort 2-3 Set (n=2075)	C-EGFR-UOC (n=139)	Overall (n=2214)
Sex - n (%)			
Female	784 (37.8)	96 (69.1)	880 (39.7)
Male	1291 (62.2)	43 (30.9)	1334 (60.3)
P-value		<0.0001	
Baseline ECOG Performance Status - n (%)			
0	566 (30.0)	4 (3.2)	570 (28.4)
1	1037 (55.0)	62 (49.6)	1099 (54.7)
2	262 (13.9)	51 (40.8)	313 (15.6)
**3**	18 (1.0)	7 (5.6)	25 (1.2)
4	2 (0.1)	1 (0.8)	3 (0.1)
Missing	190	14	204
P-value		0.0001	
Baseline LDH			
Mean (SD)	325.16 (247.61)	250.18 (105.54)	323.54 (245.65)
Median (Q1 - Q3)	268.0 (199.25-372.0)	226.0 (188.5-271.25)	267 (199-370)
Min - Max	0.0 - 5063.0	136.0 - 682.0	0.0-5063
Missing	805	111	916
P-value		0.0400	
NLR			
Mean (SD)	6.99 (11.32)	4.07 (4.40)	6.83 (11.08)
Median (Q1 - Q3)	4.55 (2.95-7.69)	3.23 (1.88-4.60)	4.45 (2.88-7.55)
Min - Max	0.0 - 245.56	0.2 - 34.68	0.0 - 245.56
Missing	214	34	248
P-value		<0.0001	
Smoking Status - n (%)			
Never	159 (8.3)	73 (52.5)	232 (11.3)
Former	1076 (56.3)	62 (44.6)	1138 (55.5)
Current	677 (35.4)	4 (2.9)	681 (33.2)
Missing	163	0	163
P-value		<0.0001	
PD-L1 - n (%)			
< 1%	604 (36.2)	34 (40.5)	638 (36.4)
1-49%	507 (30.4)	31 (36.9)	538 (30.7)
≥ 50%	559 (33.5)	19 (22.6)	578 (33)
Missing	405	55	460
P-value		0.1117	
Parenchymal Brain as Metastatic Site - n (%)			
No	1548 (79.3)	101 (72.7)	1649 (78.9)
Yes	404 (20.7)	38 (27.3)	442 (21.1)
Missing	123	0	123
P-value		0.0809	
Liver as Metastatic Site - n (%)			
No	1634 (83.7)	117 (84.2)	1751 (83.7)
Yes	318 (16.3)	22 (15.8)	340 (16.3)
Missing	123	0	123

P-value		0.9807	
Bones as Metastatic Site - n (%)			
No	1258 (64.4)	74 (53.2)	1332 (63.7)
Yes	694 (35.6)	65 (46.8)	759 (36.3)
Missing	123	0	123
P-value		0.0103	

Legend**:** N: number of patients. SD: Standard deviation. Q1: First quartile. Q3: Third quartile. Min-Max: Minimum-Maximum.

Table 36. Comparison of EGFR-UOC cohort with cohort 2 and 3: Overall Survival Comparison. The log-rank test was employed to assess any differences in terms of Overall Survival.

	Cohort 2-3 Set (n=2075)	C-EGFR-UOC (n=139)	Overall (n=2214)
Censored - n (%)	620	43	663
Deaths - n (%)	1454	96	1559
Kaplan-Meier estimate of OS (months) - n (%)			
First quartile	4.66	30.58	4.99
Median [95% CI]	12.98 [11.99–13.99]	46.02 [40.87–57.56]	14.62 [13.4-15.77]
Third quartile	33.25	79.63	38.5
Log-rank test, p-value		<0.0001	

Legend**:** N: Number of subjects. SD: standard deviation. Q1 - Q3: first – third quartile. Min - Max: minimum – maximum values. CI: confidence interval.

Supplementary Information 2

This supplementary document provides additional MLEF analyses and details that complement the main manuscript. It includes a comprehensive list of subanalyses, and further exploration of model robustness across multimodal-complete and modality-incomplete populations. Key analyses also include ablation analysis on the multimodal-complete subset, subgroup analysis based on PDL1 expression and genomics, and sensitivity analysis excluding EGFR as a driver. Additionally, this section provides more details for prognostic and predictive analysis

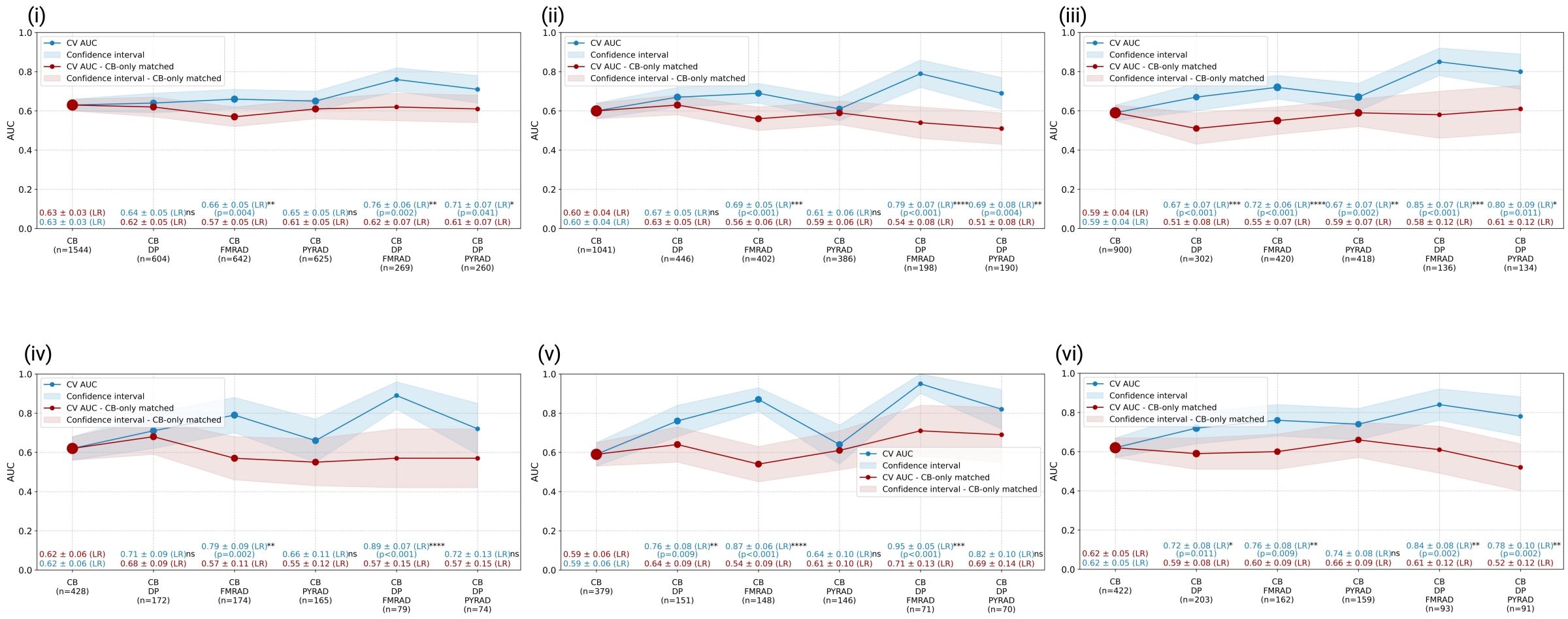

Figure 1. Classification performance of MLEF unimodal, bimodal, and multimodal models for ORR endpoint on different subanalysis: (i) C23, (ii) C2, (iii) IO-only, (iv) PDL1 <1%, (v) PDL1 1-49%, (vi) PDL1 ≥ 50%.

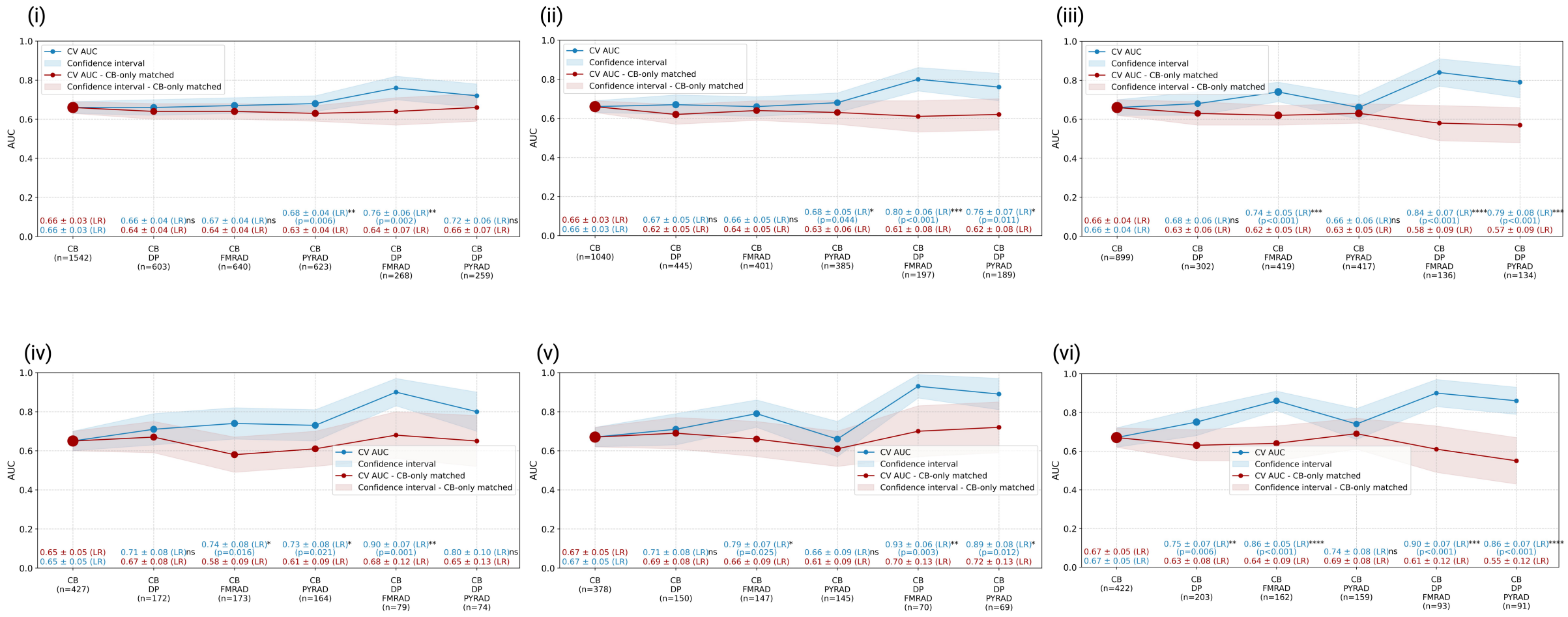

Figure 2. Classification performance of MLEF unimodal, bimodal, and multimodal models for CBR endpoint on different subanalysis: (i) C23, (ii) C2, (iii) IO-only, (iv) PDL1 <1%, (v) PDL1 1-49%, (vi) PDL1 ≥ 50%.

Name	Description
C23 main analysis	NSCLC pts treated with IO or IO/CHT at I or further metastatic lines (Cohort 2 and 3)
C2 first line	NSCLC pts treated with IO or IO/CHT at I metastatic lines (Cohort 2)
C23 PDL1 < 1%	NSCLC pts treated with IO or IO/CHT at I or further metastatic lines (Cohort 2 and 3) with PDL1 < 1%
C23 PDL1 1-49%	NSCLC pts treated with IO or IO/CHT at I or further metastatic lines (Cohort 2 and 3) with 1% ≤ PDL1 < 50%
C23 PDL1 ≥ 50%	NSCLC pts treated with IO or IO/CHT at I or further metastatic lines (Cohort 2 and 3) with PDL1 ≥50%
C23 PDL1<50%	NSCLC pts treated with IO or IO/CHT at I or further metastatic lines (Cohort 2 and 3) with PDL1 < 50%
C23 IO/CHT	NSCLC pts treated with IO/CHT at I or further metastatic lines (Cohort 2 and 3)
C23 IO-only	NSCLC pts treated with IO-only at I or further metastatic lines (Cohort 2 and 3)
C23 adeno	Adenocarcinoma NSCLC pts treated with IO or IO/CHT at I or further metastatic lines (Cohort 2 and 3)
C23 squamous	Squamous NSCLC pts treated with IO or IO/CHT at I or further metastatic lines (Cohort 2 and 3)
C23 INT	NSCLC pts treated with IO or IO/CHT at I or further metastatic lines (Cohort 2 and 3) from INT, Italy
C23 KRAS	NSCLC pts treated with IO or IO/CHT at I or further metastatic lines (Cohort 2 and 3) with KRAS mutation
C23 P53	NSCLC pts treated with IO or IO/CHT at I or further metastatic lines (Cohort 2 and 3) with P53 mutation

Table 1. List of performed subanalyses

Table 2. Sensitivity analysis: Results of the sensitivity analysis on the multimodal-complete subset. P-value computed using DeLong test. a) CV results of MLEF classification cohort C23; b) MLEF classification cohort C2, CV results.

a)

Outcome	Modality 1	Modality 2	Result Change	p-value
DCR	CB	CB DP	0.59 → 0.70	0.01

(n=269)	CB	CB FMRAD	0.59 → 0.75	0.0003
	CB DP	CB DP FMRAD	0.70 → 0.77	0.02
	CB FM RAD	CB DP FMRAD	0.75 → 0.77	0.42
OS6 (n=260)	CB	CB DP	0.69 → 0.69	0.89
	CB	CB FMRAD	0.69 → 0.77	0.06
	CB DP	CB DP FMRAD	0.69 → 0.77	0.02
	CB FMRAD	CB DP FMRAD	0.77 → 0.77	0.76
OS24 (n=238)	CB	CB DP	0.65 → 0.72	0.07
	CB	CB FMRAD	0.65 → 0.78	0.02
	CB DP	CB DP FMRAD	0.72 → 0.77	0.30
	CB FMRAD	CB DP FMRAD	0.78 → 0.77	0.49

b)

Outcome	Modality 1	Modality 2	Result Change	p-value
DCR (n=198)	CB	CB DP	0.63 → 0.73	0.04
	CB	CB FMRAD	0.63 → 0.79	0.0009
	CB DP	CB DP FMRAD	0.73 → 0.81	0.01
	CB FMRAD	CB DP FMRAD	0.79 → 0.81	0.42
OS6 (n=190)	CB	CB DP	0.69 → 0.74	0.27
	CB	CB FMRAD	0.69 → 0.84	0.002
	CB DP	CB DP FMRAD	0.74 → 0.85	0.01
	CB FMRAD	CB DP FMRAD	0.84 → 0.85	0.57
OS24 (n=168)	CB	CB DP	0.68 → 0.79	0.03
	CB	CB FMRAD	0.68 → 0.83	0.01
	CB DP	CB DP FMRAD	0.79 → 0.88	0.01
	CB FMRAD	CB DP FMRAD	0.83 → 0.88	0.09

Comment: Progressive addition of modalities beyond CB yielded consistent, statistically significant AUC improvements. For example, in C2 OS6, AUC increased from 0.689 (CB) to 0.843 (CB+FMRAD, $p < 0.01$), confirming that performance gains reflect true data integration rather than population selection effects.

Table 3. Model robustness across populations: Results of the performance of the CB-only MLEF model on both the multimodal-complete and the modality-incomplete test set populations. P-value computed using permutation test. a) C23; b) C2.

a)

Outcome	Modality	AUC multimodal-complete	n	AUC modality-incomplete	n	ΔAUC	p-value
OS6	CB RAD	0.71 [0.61-0.80]	114	0.68 [0.58-0.78]	145	0.03	0.71
	CB DP	0.69 [0.58-0.80]	96	0.69 [0.61-0.78]	163	-0.01	0.94
	CB DP RAD	0.66 [0.48-0.83]	47	0.70 [0.63-0.78]	212	-0.05	0.60
OS24	CB RAD	0.75 [0.65-0.86]	106	0.72 [0.61-0.82]	120	0.04	0.60
	CB DP	0.78 [0.67-0.90]	84	0.71 [0.62-0.81]	142	0.07	0.40
	CB DP RAD	0.64 [0.44-0.85]	42	0.75 [0.67-0.83]	184	-0.11	0.29
DCR	CB RAD	0.72 [0.63-0.81]	116	0.70 [0.61-0.78]	157	0.03	0.68
	CB DP	0.72 [0.61-0.82]	97	0.73 [0.65-0.80]	176	-0.01	0.86
	CB DP RAD	0.68 [0.52-0.85]	47	0.72 [0.66-0.79]	226	-0.04	0.66

b)

Outcome	Modality	AUC multimodal-complete	n	AUC modality-incomplete	n	ΔAUC	p-value
OS6	CB RAD	0.66 [0.48-0.84]	63	0.70 [0.58-0.82]	103	-0.04	0.71
	CB DP	0.65 [0.49-0.81]	68	0.70 [0.57-0.84]	98	-0.05	0.61
	CB DP RAD	0.54 [0.22-0.86]	29	0.71 [0.61-0.82]	137	-0.17	0.25
OS24	CB RAD	0.72 [0.58-0.86]	55	0.79 [0.69-0.90]	83	-0.07	0.43
	CB DP	0.67 [0.49-0.86]	57	0.81 [0.71-0.90]	81	-0.13	0.13
	CB DP RAD	0.59 [0.29-0.90]	24	0.79 [0.71-0.88]	114	-0.20	0.09
DCR	CB RAD	0.60 [0.45-0.75]	65	0.70 [0.59-0.81]	115	-0.09	0.31
	CB DP	0.63 [0.47-0.80]	69	0.70 [0.60-0.80]	111	-0.07	0.45
	CB DP RAD	0.47 [0.13-0.81]	29	0.70 [0.61-0.79]	151	-0.23	0.06

Comment: No statistically significant AUC differences were found between the two populations across cohorts and outcomes (OS6, OS24, DCR; $p > 0.05$), indicating that the multimodal-complete subset does not represent a positively biased, easier-to-predict population.

Table 4. ML CB only models performance across mutation subgroups. a) test results for C23 KRAS-mutated patients; b) test results for C23 P53-mutated patients; c) test results for C23 STK11-mutated patients; d) test results for C23 KRAS and P53-mutated patients

a)

Outcome	n. test	test AUC/c-index
DCR	52	0.63 ± 0.2
OS6	47	0.72 ± 0.19
OS24	34	0.71 ± 0.18
OS	53	0.65 ± 0.14

b)

Outcome	n. test	test AUC/c-index
DCR	46	0.72 ± 0.17
OS6	43	0.77 ± 0.22
OS24	33	0.69 ± 0.18
OS	47	0.60 ± 0.14

c)

Outcome	n. test	test AUC/c-index
DCR	7	/
OS6	8	/
OS24	8	0.87 ± 0.21
OS	8	0.38 ± 0.36

d)

Outcome	n. test	test AUC/c-index
DCR	15	0.62 ± 0.5
OS6	15	0.54 ± 0.61
OS24	9	0.70 ± 0.35
OS	/	/

Table 5. Prognostic vs predictive. a) Performance of classification MLEF CB models on I3LUNG test set and new non-IO cohorts; b) Performance of survival MLEF CB models on I3LUNG test set and new non-IO cohorts.

a)

Cohort	Model	Endpoint	n	Class distribution	AUC	F1	Sensitivity	Specificity
C23 TEST (I3LUNG)	MLEF CB C23	OS6	259	OS < 6m: 77<br>OS ≥ 6m: 182	0.69 ± 0.07	0.62	0.65	0.64
		OS24	226	OS < 24m: 166<br>OS ≥ 24m: 60	0.74 ± 0.07	0.58	0.85	0.50
		ORR	273	NR: 178<br>R: 95	0.66 ± 0.06	0.58	0.88	0.32
		DCR	273	NR: 104<br>R: 169	0.71 ± 0.06	0.64	0.72	0.56
C-EGFR-INT	MLEF CB C23	OS6	129	OS < 6m: 8<br>OS ≥ 6m: 121	0.61 ± 0.221	0.38	0.47	0.62
		OS24	105	OS < 24m: 54<br>OS ≥ 24m: 51	0.53 ± 0.112	0.48	0.59	0.39
		ORR	132	NR: 50<br>R: 82	0.57 ± 0.103	0.50	0.39	0.68
		DCR	132	NR: 3<br>R: 129	0.68 ± 0.114	0.43	0.69	0.33
C-EGFR-UOC	MLEF CB C23	OS6	138	OS < 6m: 5<br>OS ≥ 6m: 133	0.74 ± 0.296	0.29	0.33	0.80
		OS24	129	OS < 24m: 25<br>OS ≥ 24m: 104	0.53 ± 0.125	0.50	0.70	0.32
		ORR	139	NR: 119<br>R: 20	0.50 ± 0.132	0.50	0.10	0.91
		DCR	139	NR: 12<br>R: 127	0.62 ± 0.213	0.26	0.23	0.75
C-StageIII-CHT	MLEF CB C23	OS6	88	OS < 6m: 15<br>OS ≥ 6m: 73	0.68 ± 0.123	0.57	0.99	0.13
		OS24	68	OS < 24m: 37<br>OS ≥ 24m: 31	0.70 ± 0.133	0.34	1.00	0.03
		ORR	91	NR: 46<br>R: 45	0.58 ± 0.118	0.59	0.64	0.54
		DCR	91	NR: 15<br>R: 76	0.65 ± 0.146	0.51	0.97	0.07
C-LineI-CHT		OS6	208	OS < 6m: 18<br>OS ≥ 6m: 190	0.72 ± 0.087	0.51	0.71	0.50

	MLEF CB C2	OS24	205	OS < 24m: 122<br>OS ≥ 24m: 83	0.64 ± 0.078	0.58	0.35	0.82
		ORR	204	NR: 141<br>R: 63	0.58 ± 0.085	0.51	0.62	0.48
		DCR	204	NR: 79<br>R: 125	0.58 ± 0.081	0.54	0.68	0.41

b)

Cohort	Model	Endpoint	n	C-index
C23 TEST (I3LUNG)	MLEF CB C23	OS	273	0.66 ± 0.04
C-EGFR-INT		OS	132	0.58 ± 0.072
C-EGFR-UOC		OS	139	0.61 ± 0.069
C-StageIII-CHT		OS	97	0.63 ± 0.097
C-LineI-CHT	MLEF CB C2	OS	208	0.62 ± 0.039

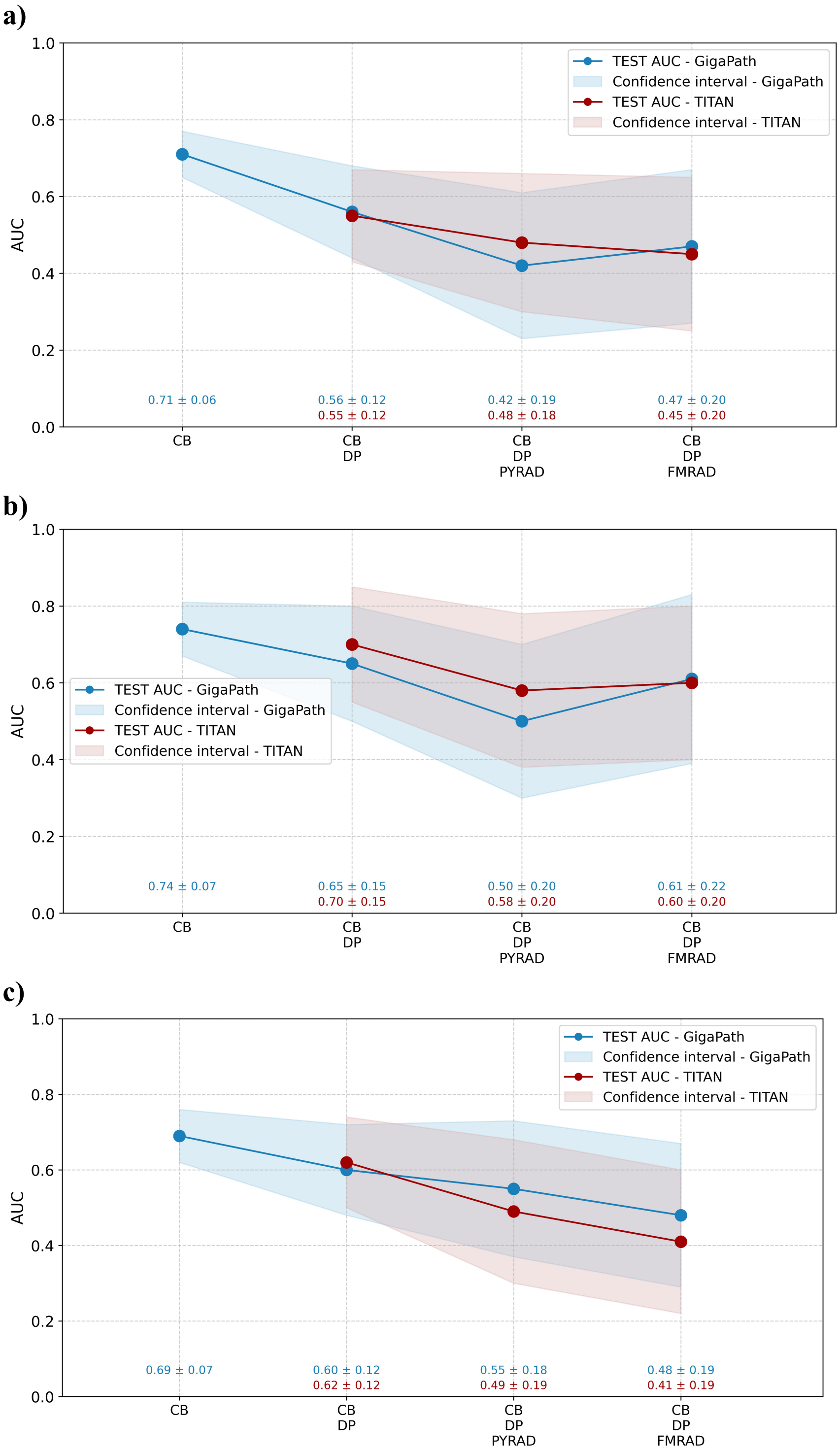

Figure 3. Comparison of MLEF performance in TEST using Prov-GigaPath and TITAN digital pathology features across response outcomes and modality combinations.

To assess whether the results were driven by the choice of digital pathology feature extractor, 768-dimensional slide-level embedding features were re-extracted using TITAN, a multimodal whole-slide foundation model pretrained with self-supervised learning on pathology whole-slide images and vision-language alignment with pathology reports (Ding et al., *Nature Medicine*, 2025). Results obtained using Prov-GigaPath digital pathology features were compared with those obtained using TITAN across all three response outcomes and modality combinations. **a)** DCR, **b)** OS24, **c)** OS6 outcome.

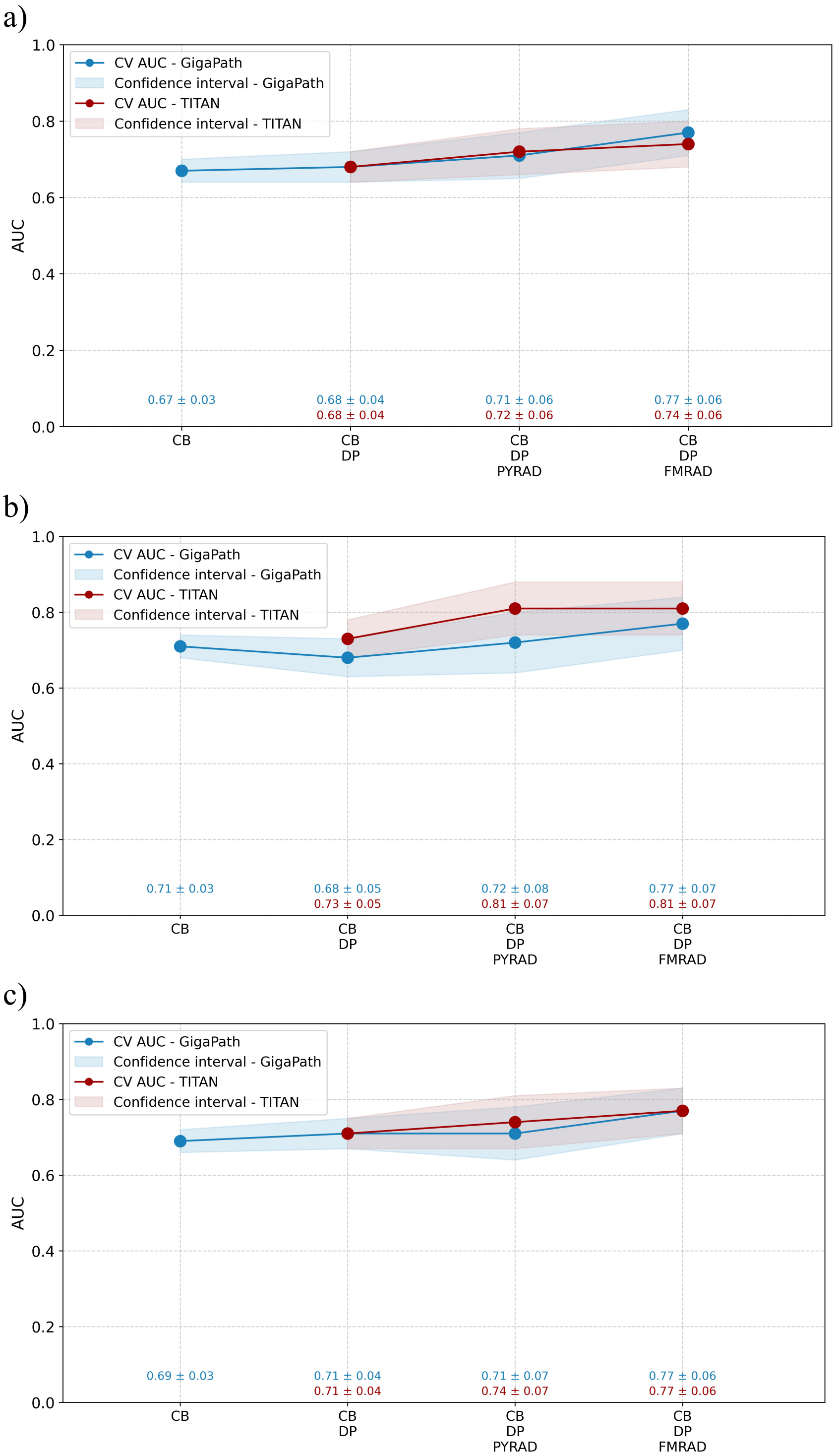

Figure 4. Comparison of MLEF performance in CV using Prov-GigaPath and TITAN digital pathology features across response outcomes and modality combinations.

To assess whether the results were driven by the choice of digital pathology feature extractor, 768-dimensional slide-level embedding features were re-extracted using TITAN, a multimodal whole-slide foundation model pretrained with self-supervised learning on pathology whole-slide images and vision-language alignment with pathology reports (Ding et al., *Nature Medicine*, 2025). Results obtained using Prov-GigaPath digital pathology features were compared with those obtained using TITAN across all three response outcomes and modality combinations. **a)** DCR, **b)** OS24, **c)** OS6 outcome.

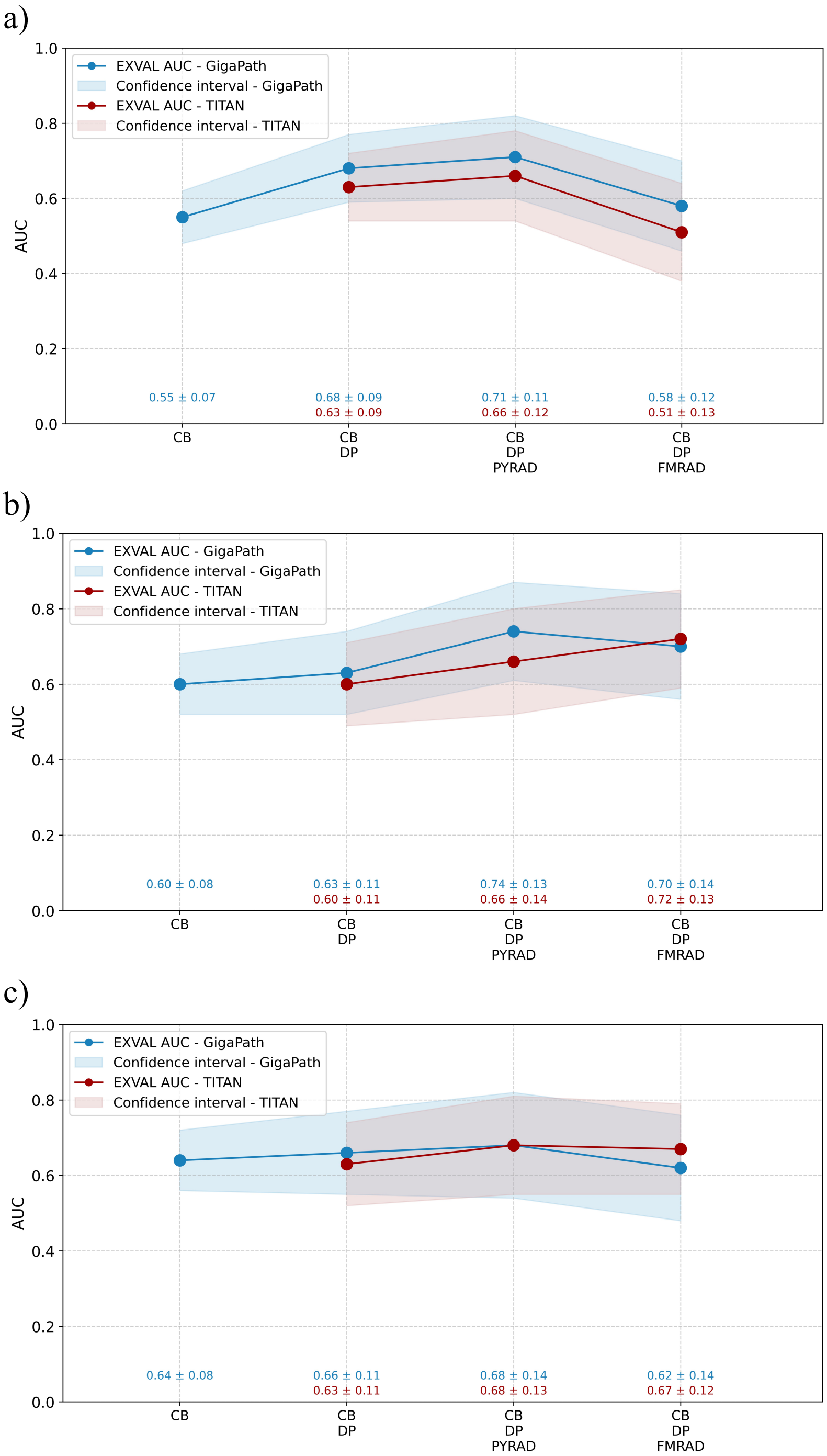

Figure 5. Comparison of MLEF performance in EXVAL using Prov-GigaPath and TITAN digital pathology features across response outcomes and modality combinations.

To assess whether the results were driven by the choice of digital pathology feature extractor, 768-dimensional slide-level embedding features were re-extracted using TITAN, a multimodal whole-slide foundation model pretrained with self-supervised learning on pathology whole-slide images and vision-language alignment with pathology reports (Ding et al., *Nature Medicine*, 2025). Results obtained using Prov-GigaPath digital pathology features were compared with those obtained using TITAN across all three response outcomes and modality combinations. **a)** DCR, **b)** OS24, **c)** OS6 outcome.

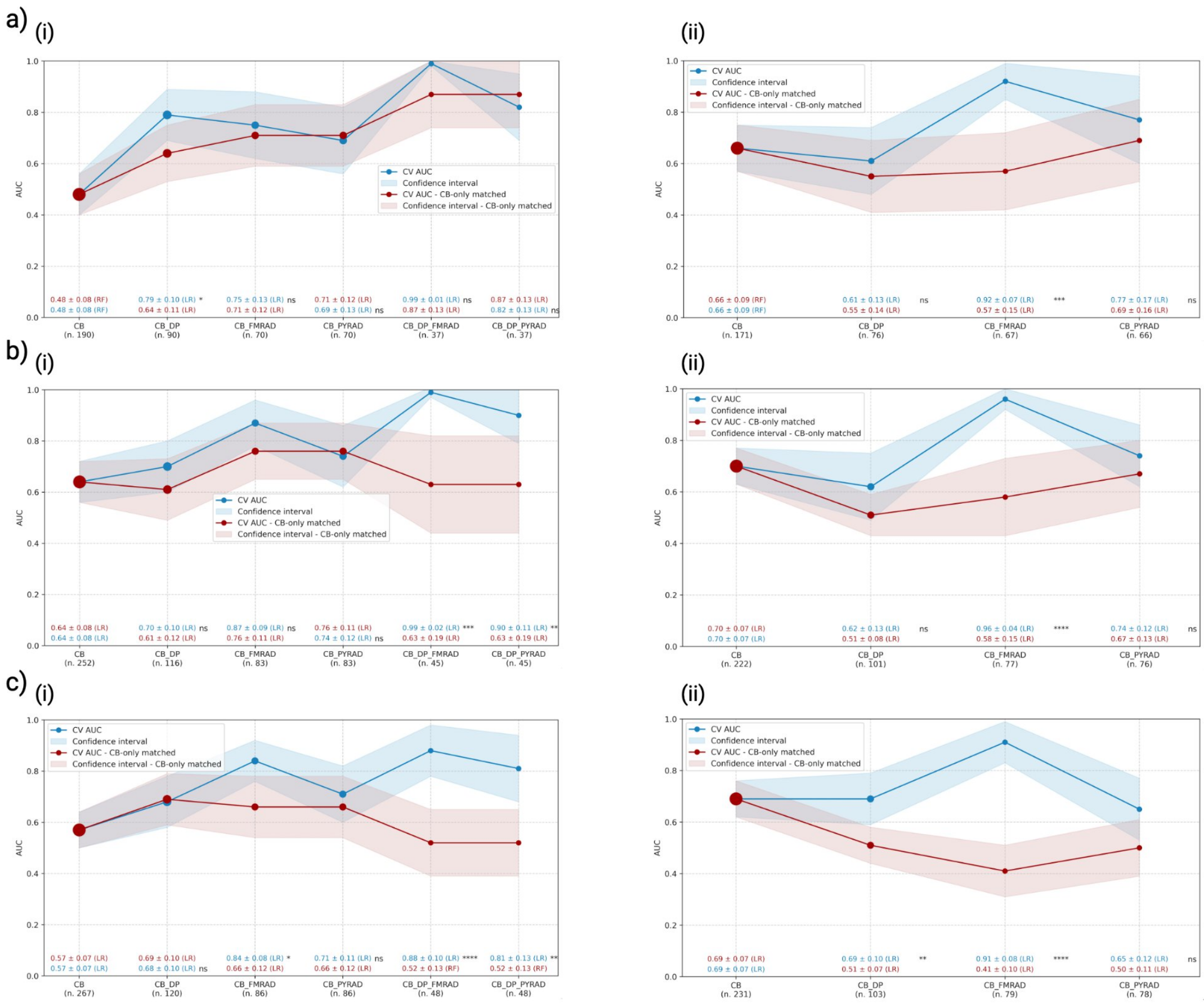

Figure 6. Classification performance of MLEF unimodal, bimodal, and multimodal models for KRAS mutation and P53 mutation patient population. a) OS24 (i) KRAS mutated, (ii) P53 mutated; b) OS6 (i) KRAS mutated, (ii) P53 mutated; DCR (i) KRAS mutated, (ii) P53 mutated.

Supplementary References

1. Xu, H., Usuyama, N., Bagga, J. et al. A whole-slide foundation model for digital pathology from real-world data. Nature 630, 181–188 (2024). https://doi.org/10.1038/s41586-024-07441-w

2. Ding, T., Wagner, S.J., Song, A.H. et al. A multimodal whole-slide foundation model for pathology. Nat Med 31, 3749–3761 (2025). https://doi.org/10.1038/s41591-025-03982-3

Supplementary Information 4

This supplementary document provides additional analyses of the deep learning models evaluated in this work. It is organized into three sections. Section S1 includes a systematic comparison of our DLIF framework against two state-of-the-art baselines specifically designed to handle missing modalities: DyAM (Vanguri et al., Nature Cancer 2022), an attention-based late-fusion architecture, and MCVAE (Zanitti et al., IEEE ICBD 2025), a contrastive variational autoencoder with availability-aware gating. The comparison covers all five endpoints (ORR, DCR, CBR, OS_6, OS_24), both cohorts (C23, C2), and three evaluation sets (LOCO cross-validation, test, external validation), with a full per-modality breakdown of the results. Section S2 presents model robustness analyses across clinically relevant subpopulations. Section S3 compares results between the digital pathology feature extractor used in the original models, Prov-GigaPath (Xu et al., Nature 2024), and a newer model, TITAN (Ding et al., Nature Medicine 2025).

Section S1.

Comparison DLIF, DyAm and MCVAE

Cohort and Endpoints. DyAM (Vanguri et al., 2022) and MCVAE (Zanitti et al., 2025) were applied to our NSCLC cohort of 2.075 patients across two treatment groups (IO+chemo and IO alone), evaluating five binary endpoints: OS6, OS24, CBR, DCR, and ORR.

Additional Details on DyAM

Modalities and Model Configurations. We evaluated unimodal (clinical), bimodal, trimodal, and multimodal configurations following the DyAM design (Vanguri et al., 2022). The attention gate was disabled for unimodal models, as in the original paper. Modalities included 11 clinical variables, 128 PyRadiomics features (PYRAD), 4,096 deep radiomics features (FMRAD), 768 digital pathology features (DP), and a 4-gene genomic panel (G).

Clinical, DP, and G preprocessing was retained from our existing validated pipeline (scaling and imputation). For radiomics, preprocessing was reproduced from Vanguri et al.: outlier- and robustness-based feature selection, scaling, and LASSO dimensionality reduction. Inter-lesion variance terms were omitted as only one lesion per patient was available.

Training and Cross-Validation. The original DyAM 5-fold cross-validation was replaced with leave-one-center-out (LOCO). AUCs were computed using DeLong's method on pooled held-out center predictions. A standard train/test/external-validation split was also implemented.

Hyperparameter tuning used the IO+CHT cohort, OS6 endpoint, and the full multimodal configuration. Training used 90% of the training data; 10% was held out as internal validation. Tuned parameters: number of epochs, learning rate, L2 regularization (α), AR2 loss weight (β).

Additional Details on MCVAE

MCVAE (Zanitti et al., 2025) is designed to handle arbitrary patterns of missing modalities without reconstruction. It was originally validated on TCGA-LUAD and TCGA-LUSC for survival prediction. Because of the focus on survival tasks and the lack of external validation in the original paper, we adapted MCVAE's architecture. To support classification tasks, we replaced the Cox partial likelihood loss with standard cross-entropy. We also modified the fusion mechanism and the reconstruction

objective to improve generalization to external validation data and achieve robust results across clinical centres. We detail each modification and its motivation below.

Encoders. We replaced the modality encoders with simpler ones that match the depth used in DLIF.

Fusion Module. The original fusion module originally computed a weighted sum of modality embeddings using learned gates, followed by a post-fusion projection network. While this worked well for TCGA data, we found that the post-fusion network generalized poorly on the external validation set. We replaced this with a distribution-space fusion that approximates per-modality posteriors as a single Gaussian by matching the first two moments of the weighted mixture: the fused mean is a weighted average of the per-modality means, and the fused variance accounts for both individual variances and the spread of means. The post-fusion projection was removed. This change resulted in more stable performance across tasks and cohorts with fewer learnable parameters. In particular, for each available modality k, the variational encoder produces a posterior $q_{\phi_k}\left(z_k \mid x_k\right) = N\left(\mu_k, \sigma_k^2\right)$. The mixture weights α_k are computed via softmax over learned gating parameters γ_k, masked to available modalities:

$$\alpha_k = \frac{exp\left(\gamma_k\right)}{\sum_{j \in A_i} exp\left(\gamma_j\right)}, \quad k \in A_i,$$

where $\mathcal{A}_i$ denotes the set of available modalities for the patient i. The fused posterior is then approximated as $\left(\mu_{fused}, \sigma_{fused}^2\right)$ with:

$$\mu_{fused} = \sum_{k \in A_i} \alpha_k \, \mu_k,$$

$$\sigma_{fused}^2 = \sum_{k \in A_i} \alpha_k \left(\sigma_k^2 + \mu_k^2\right) - \mu_{fused}^2.$$

Finally, a fused representation z_{fused} is sampled from this distribution via:

$$z_{fused} = \mu_{fused} + \sigma_{fused} \odot \epsilon, \quad \epsilon \sim N\left(0, I\right).$$

Reconstruction Objective. In the original model, modality decoders were reconstructed from the fused representation z_{fused}, serving as a regularizer for the fusion module. This requires the fused representation to simultaneously retain sufficient modality-specific detail for reconstruction (a generative objective) while discarding modality-specific noise for classification/survival (a discriminative objective). However, during external validation, this approach showed limited generalizability, and we adopted modality-specific decoding, which proved more robust across datasets. This objective was therefore rewritten as:

$$L_{recon} = \sum_{k \in A_i} \| x_k - g_{\psi_k}\left(z_k\right) \|_2^2,$$

Where g_{ψ_k} is the decoder for modality k.

Results

At CV level, all three models are indistinguishable across all endpoints and cohorts, confirming equivalent training capacity. Differences emerge at external validation, thus, in the generalization capabilities. DyAM CIs (±0.03–0.05 at EXVAL) are consistently narrower than DLIF (±0.06–0.09) and comparable to MCVAE (±0.03–0.08). Adding modalities beyond clinical data does not improve EXVAL AUC for any model or endpoint.

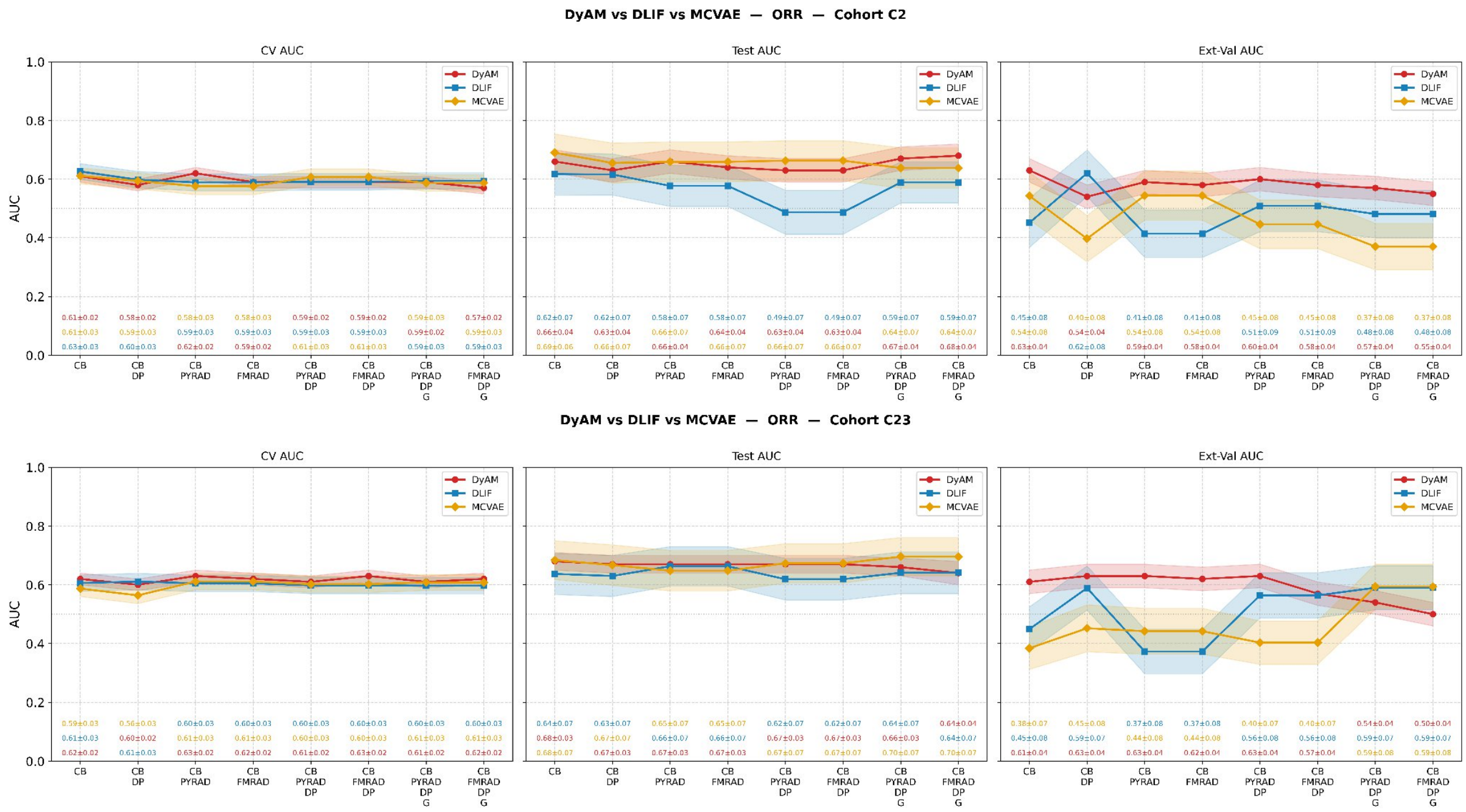

Figure 1. Dyam vs Dlif vs MCVAE, ORR, cohort 2 and cohort 23

DyAM vs DLIF. At CV, all three models CB-only converge at AUC levels between 0.59–0.63. At EXVAL, DyAM CB-only outperforms DLIF CB-only in both cohorts with non-overlapping CIs: in C23, DyAM 0.61±0.04 [0.57–0.65] vs DLIF 0.45±0.08 [0.37–0.53]; in C2, DyAM 0.63±0.04 [0.59–0.67] vs DLIF 0.45±0.08. Confounded radiomics configurations negatively affect DLIF's performance (0.37–0.41) compared to DyAM (0.62–0.63). CB_DP partially recovers DLIF performance (~0.59–0.62).

MCVAE vs DLIF. MCVAE is visibly underperforming on ORR at EXVAL. In C23, MCVAE CB-only configuration to 0.38±0.07 against DLIF's 0.45±0.08, and degrades further with imaging modalities, reaching 0.30–0.37 in confounded configurations. In C2, MCVAE EXVAL ranges between 0.54±0.08 (CB-only) and 0.37±0.08 (all modalities), while DLIF is consistently better. DLIF is the more robust of the two for this endpoint.

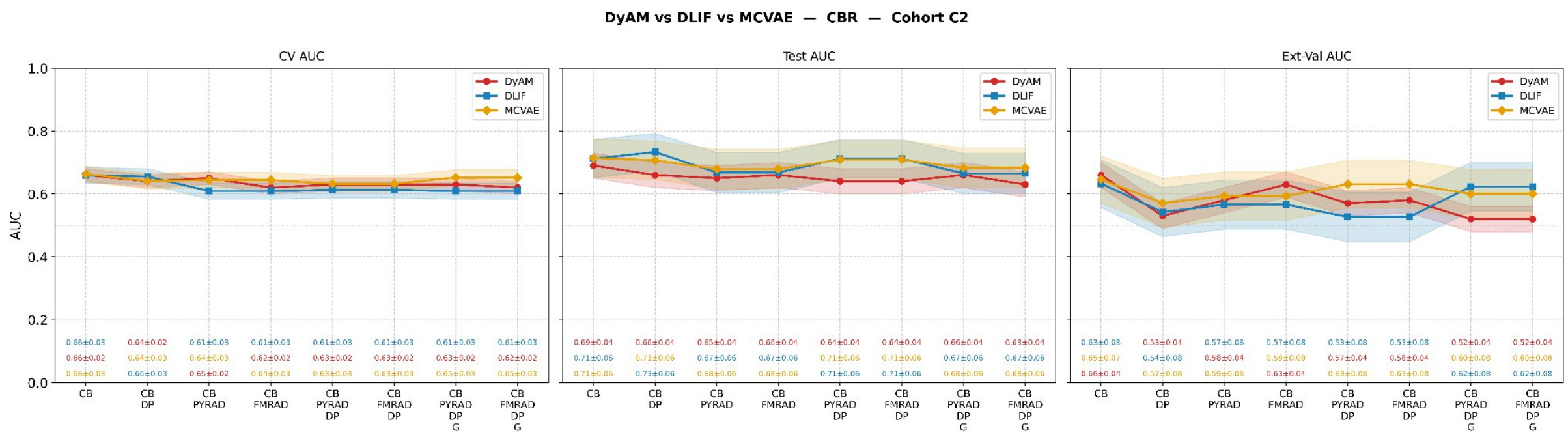

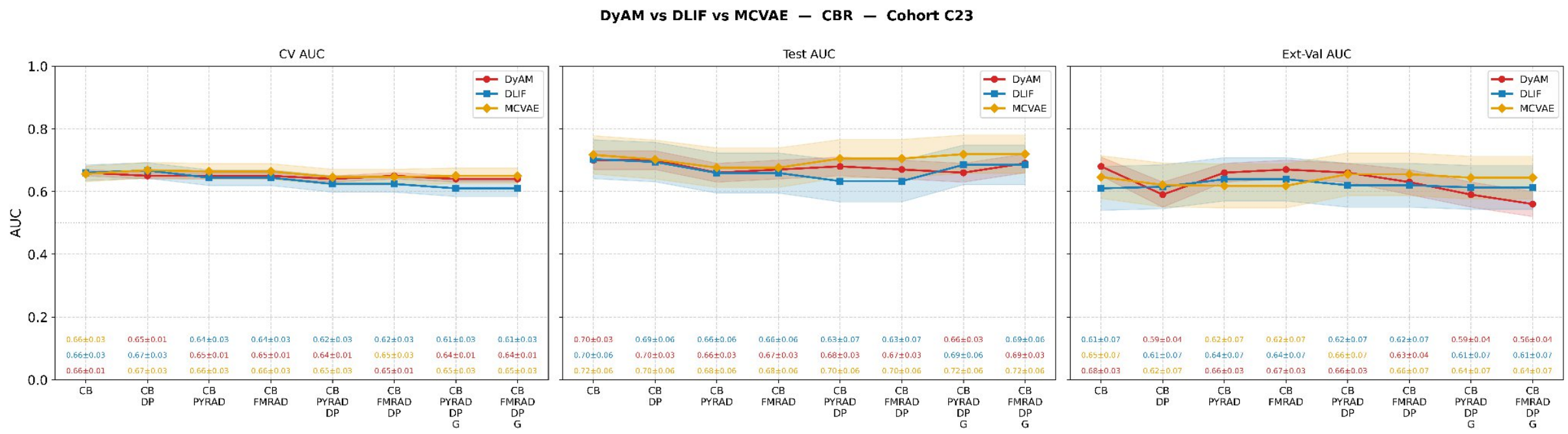

Figure 2. Dyam vs Dlif vs MCVAE, CBR cohort 2 and cohort 23

DyAM vs DLIF. CV (CB-only) is perfectly matched (0.66 in all configurations). DyAM consistently achieves higher EXVAL than DLIF, with non-overlapping CIs in C23 CB-only: DyAM 0.68±0.03 [0.65–0.71] vs DLIF 0.61±0.07 [0.54–0.68]. In C2 the gap persists (DyAM 0.66±0.04 vs DLIF 0.54±0.08) with overlapping CIs.

MCVAE vs DLIF. MCVAE matches or exceeds DLIF on CBR at EXVAL. In C23, MCVAE CB-only reaches 0.68±0.03, below DyAM and outperforming DLIF (0.61±0.07); the gap is borderline non-overlapping. In C2, MCVAE CB-only is 0.65±0.04 vs DLIF 0.54±0.08. MCVAE CIs are notably narrower than DLIF's (±0.03–0.04 vs ±0.07–0.08), suggesting more stable generalization for this endpoint.

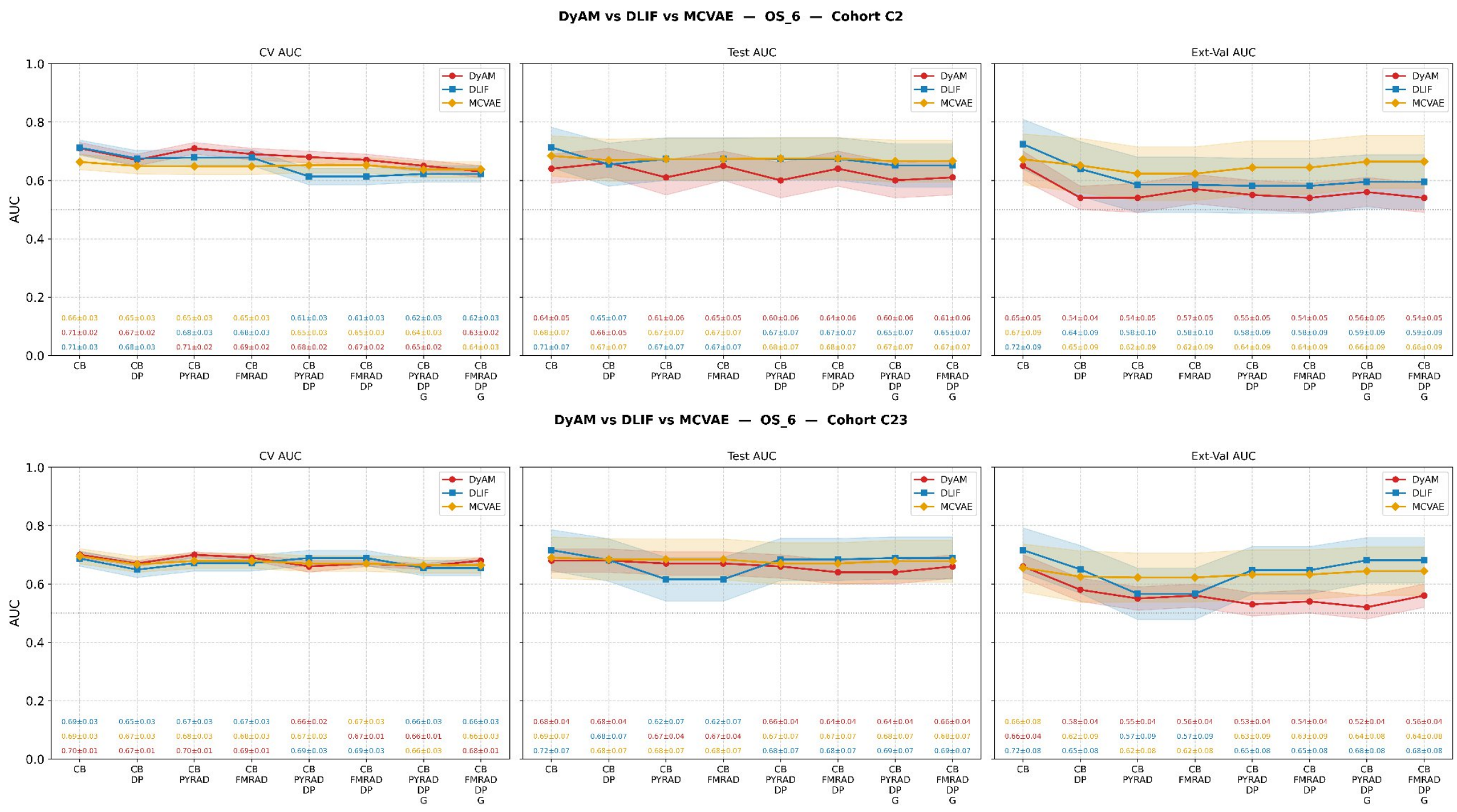

Figure 3. Dyam vs Dlif vs MCVAE, OS6, cohort 2 and cohort 23

DyAM vs DLIF. CV CB-only performance is identical across models (~0.69–0.71). At EXVAL, DLIF CB-only consistently outperforms DyAM CB-only by ~0.06–0.07 across both cohorts: C23 DLIF 0.72±0.08 vs DyAM 0.66±0.04; C2 DLIF 0.72±0.09 vs DyAM 0.65±0.05. DyAM CB-only test AUC also drops below CV in both cohorts (C2: CV 0.71 → test 0.64, C23: CV 0.70 → test 0.68), a pattern absent in DLIF.

MCVAE vs DLIF. For OS6, MCVAE and DLIF are broadly equivalent at EXVAL on clean configurations. In C23, MCVAE (CB-only) reaches 0.72±0.08, matching DLIF and outperforming DyAM. In C2, MCVAE (CB-only) is 0.65±0.09 vs DLIF 0.67±0.09, fully overlapping. Adding imaging modalities degrades EXVAL in MCVAE as well, though less severely than for ORR.

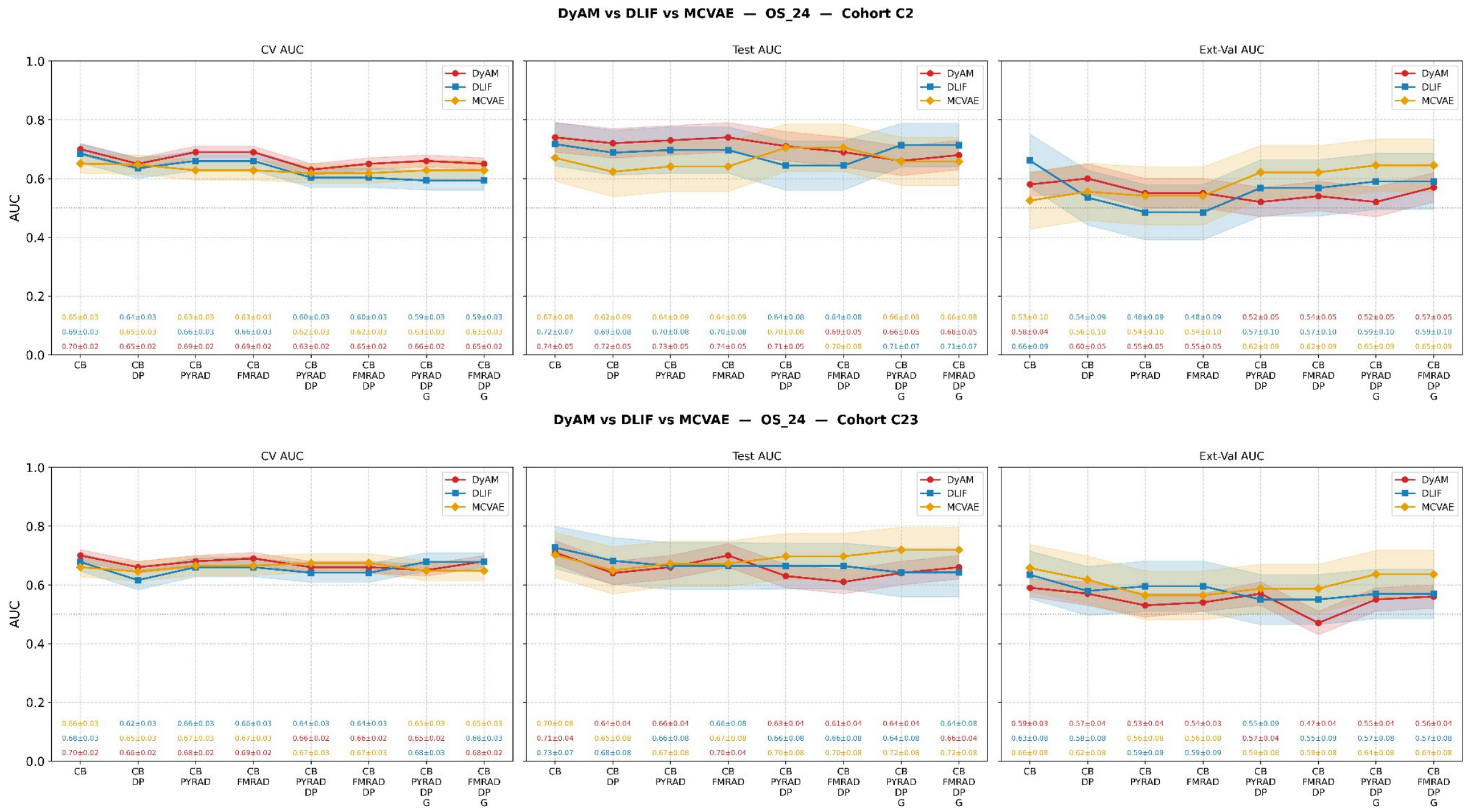

Figure 4. Dyam vs Dlif vs MCVAE, OS24, cohort 2 and cohort 23

DyAM vs DLIF. All models match at CV CB-only with AUC ranging between 0.65–0.70, but suffer large EXVAL drops. DyAM CB-only generalizes poorly compared to DLIF CB-only: C23 0.59±0.03 [0.56–0.62] vs DLIF 0.63±0.08; C2 0.58±0.04 [0.54–0.62] vs DLIF 0.66±0.09 [0.57–0.75]. The C2 gap is suggestive of a real DLIF advantage. Modality additions are uniformly harmful for both models.

MCVAE vs DLIF. In C23, MCVAE CB-only achieves 0.66±0.08 at EXVAL, similarly to DLIF (0.63±0.08). In C2, MCVAE (CB-only) is 0.53±0.10, below DLIF's 0.66±0.09 with overlapping but consistent CIs. MCVAE shows greater instability across modality configurations at EXVAL in C2 (0.48–0.55 for radiomics configurations). Across both cohorts, DLIF holds a modest advantage on OS24 over MCVAE.

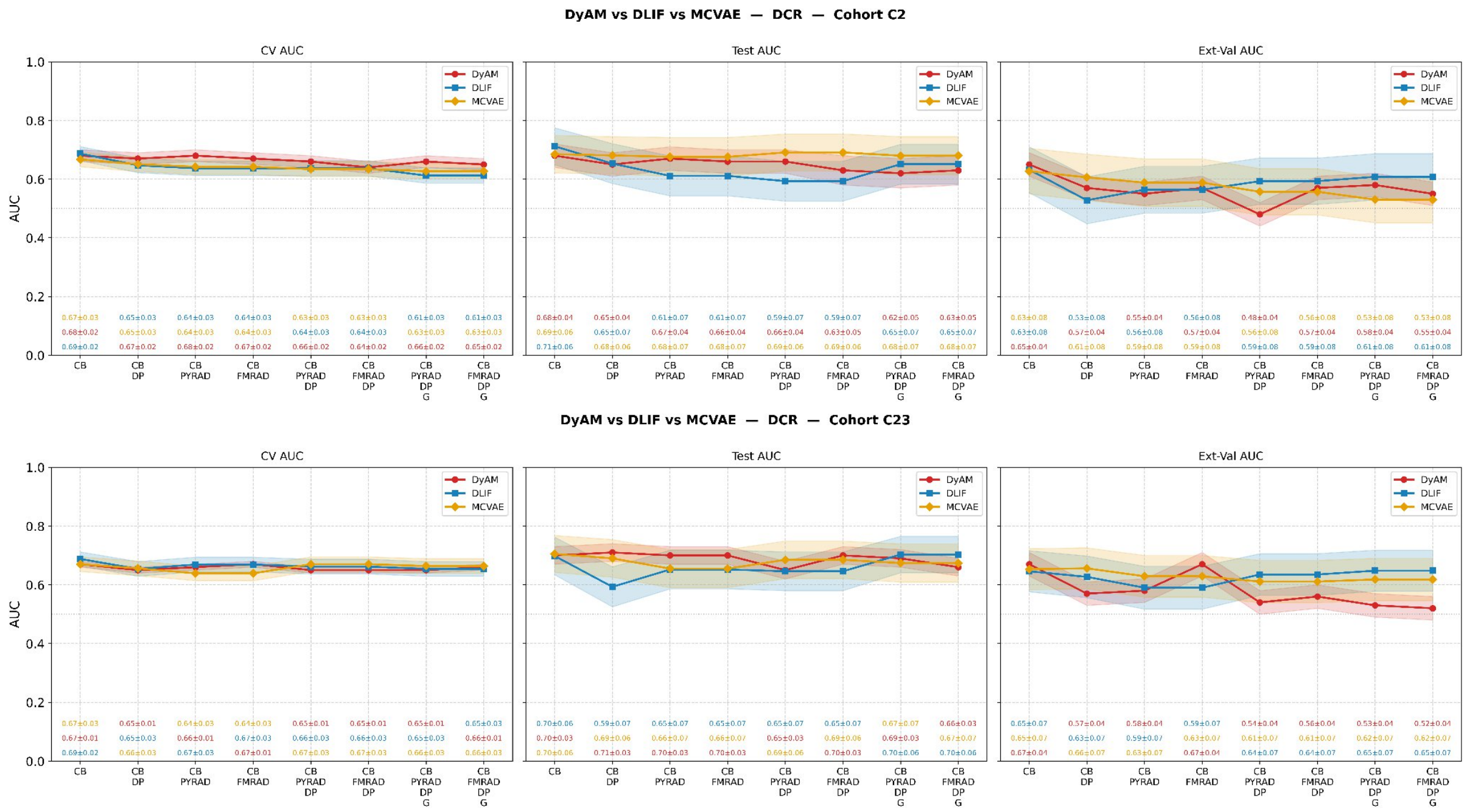

Figure 5. Dyam vs Dlif vs MCVAE, DCR, cohort 2 and cohort 23

DyAM vs DLIF. For CB-only, CV, test, and EXVAL are within CI for both models and cohorts: C23 DyAM 0.67±0.04 [0.63–0.71] vs DLIF 0.65±0.07 [0.58–0.72]; C2 DyAM 0.65±0.04 vs DLIF 0.63±0.08. Neither architecture shows a meaningful advantage. One divergence in CB_FMRAD_DP C23 (DyAM 0.56 vs DLIF 0.64) is confounded by radiomics preprocessing.

MCVAE vs DLIF. MCVAE CB-only EXVAL is 0.67±0.04 in C23 and 0.65±0.04 in C2, overlapping with both DLIF and DyAM. MCVAE CIs remain narrower than DLIF's (±0.04–0.07 vs ±0.07–0.08). The three architectures are functionally equivalent for DCR.

Global Summary. At CV level, DyAM, MCVAE, and DLIF are indistinguishable across all endpoints and cohorts. Performance differences emerge exclusively from generalization to held-out centres. DyAM shows superior EXVAL generalization on response and benefit endpoints (ORR, CBR), where it achieves the highest AUCs with narrower CIs. On survival endpoints (OS6, OS24), DLIF consistently outperforms DyAM at EXVAL by ~0.06–0.07, a direction reproducible across cohorts. For DCR, neither architecture holds a consistent advantage.
MCVAE presents a more differentiated profile. On ORR, MCVAE generalizes poorly at EXVAL, reaching the lowest AUCs observed across the entire comparison. On OS6 it matches DLIF and outperforms DyAM. On OS24 it is intermediate between DyAM and DLIF. On CBR and DCR it tracks DyAM closely and outperforms DLIF, with consistently narrower CIs suggesting more stable variance in generalization for these endpoints.
Adding modalities beyond clinical data does not improve EXVAL AUC in any endpoint for any model. CB-only remains the most robust configuration across all outcomes, models, and cohorts.

Supplementary References

1. Vanguri, Rami S., et al. "Multimodal integration of radiology, pathology and genomics for prediction of response to PD-(L) 1 blockade in patients with non-small cell lung cancer." *Nature cancer* 3.10 (2022): 1151-1164.
2. Zanitti, Michele, et al. "A Contrastive Variational AutoEncoder for NSCLC Survival Prediction with Missing Modalities." *arXiv preprint arXiv:2602.17402* (2026).

Section S2.

Model Robustness across Populations

To examine model robustness across populations, we conducted three experiments on various patient subsets. First, to determine whether the availability of data from different modalities enriched the CB data, we tested the CB-only model on various patient subsets in the presence or absence of the availability of given modalities. Next, we examine the performance of all models on the subset of patients with complete modality availability to determine whether this subset of patients is enriched. Finally, we conduct a sensitivity analysis by determining how the all-modality models perform on the modality-complete patient subset when certain modalities are masked.

CB-only Model Performance on Patient Modality Subsets

We tested the performance of the CB-only model on subsets of patients based on their data availability by modality. For each outcome, the dashed gray line indicates the "baseline" model performance on all patients. Different combinations of modality availability are then tested. For each modality combination, the blue dot represents the performance of the model on the subset of patients who have all the listed modalities available, while the red dot represents the performance of the model on patients who do not have any of the listed modalities.

While the confidence intervals mostly overlap, in the vast majority of the tests, the subset of patients with the specified modalities outperforms the one without. However, it is not evident that the model performs better on patients with all modalities available (as can be seen by the blue dots being below the baseline performance on the model across cohorts and outcomes for this subset). It is also important to note that in the test set analyzed here, there are only 35 to 40 patients with all modalities, depending on the outcome. It appears that the subset of patients that do not have any modality available beyond CB (red dot in the bottom two lines) represents a subpopulation on which the model struggles to predict reliably. Furthermore, we can see from line 4 (Genomics) that the model underperforms for patients without available genomics data.

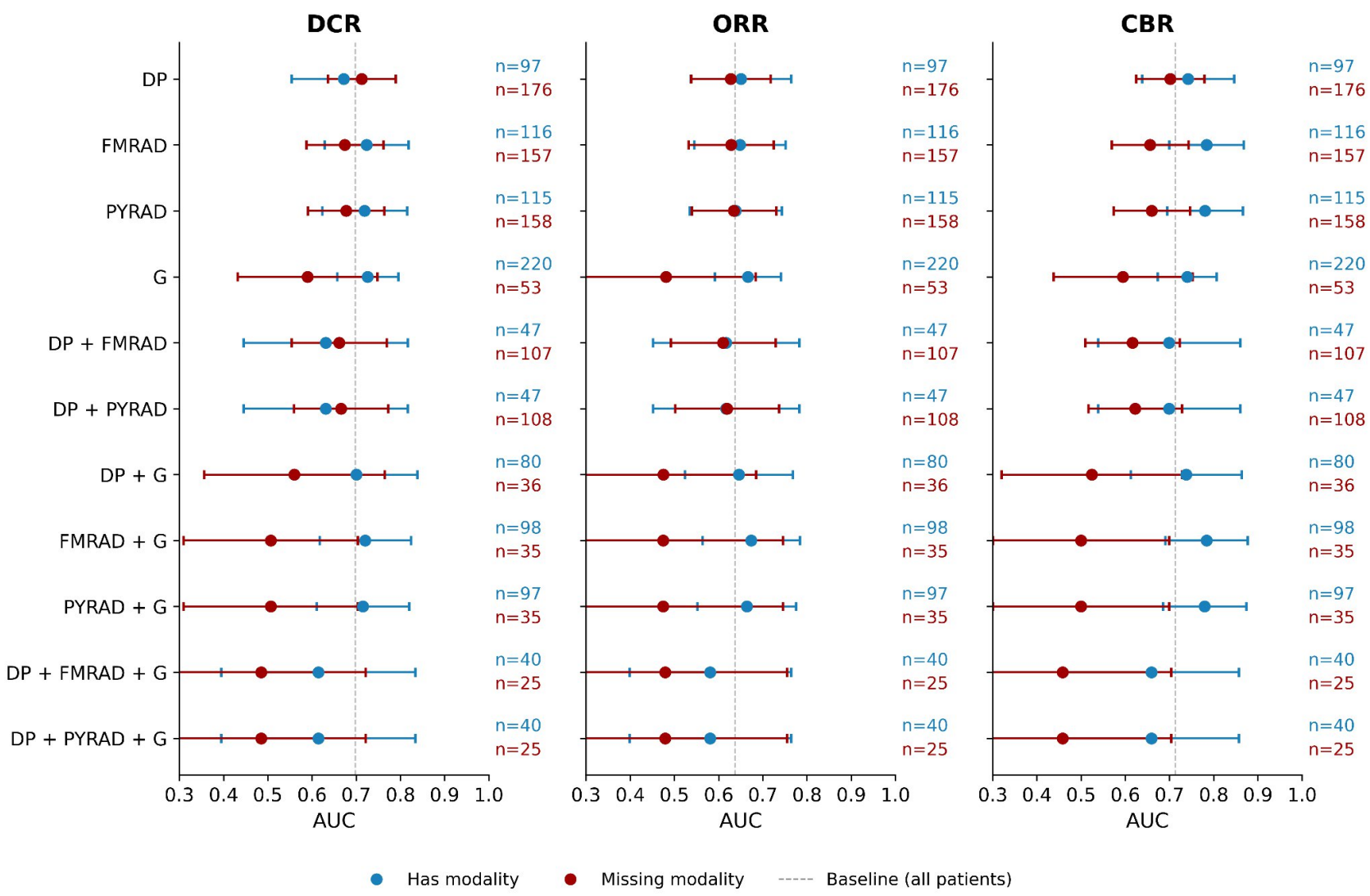

Figure 6. CB Model Performance on Response Outcomes by Data Availability, cohort 23

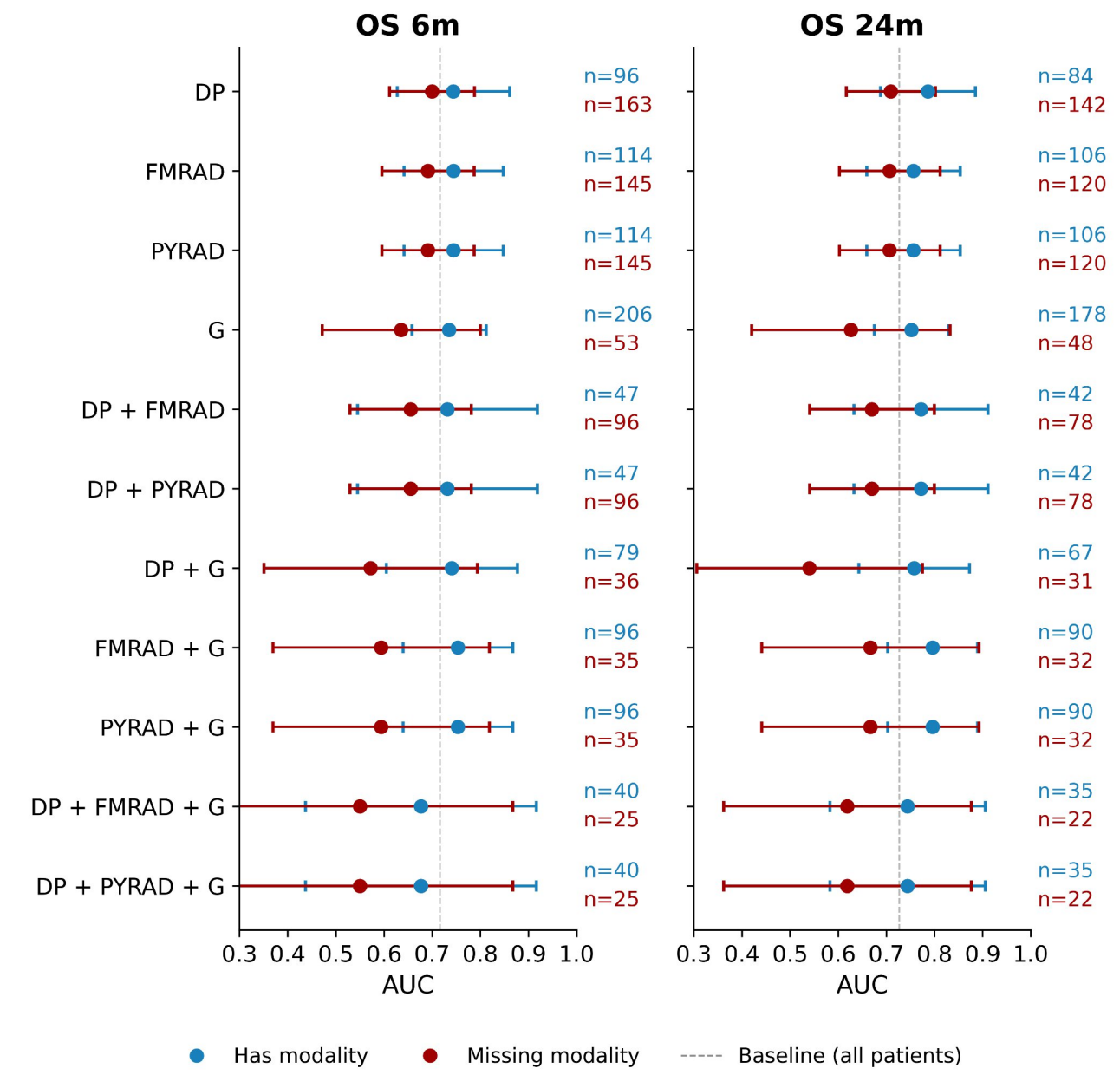

Figure 7. CB Model Performance on Survival Outcomes by Data Availability, cohort 23

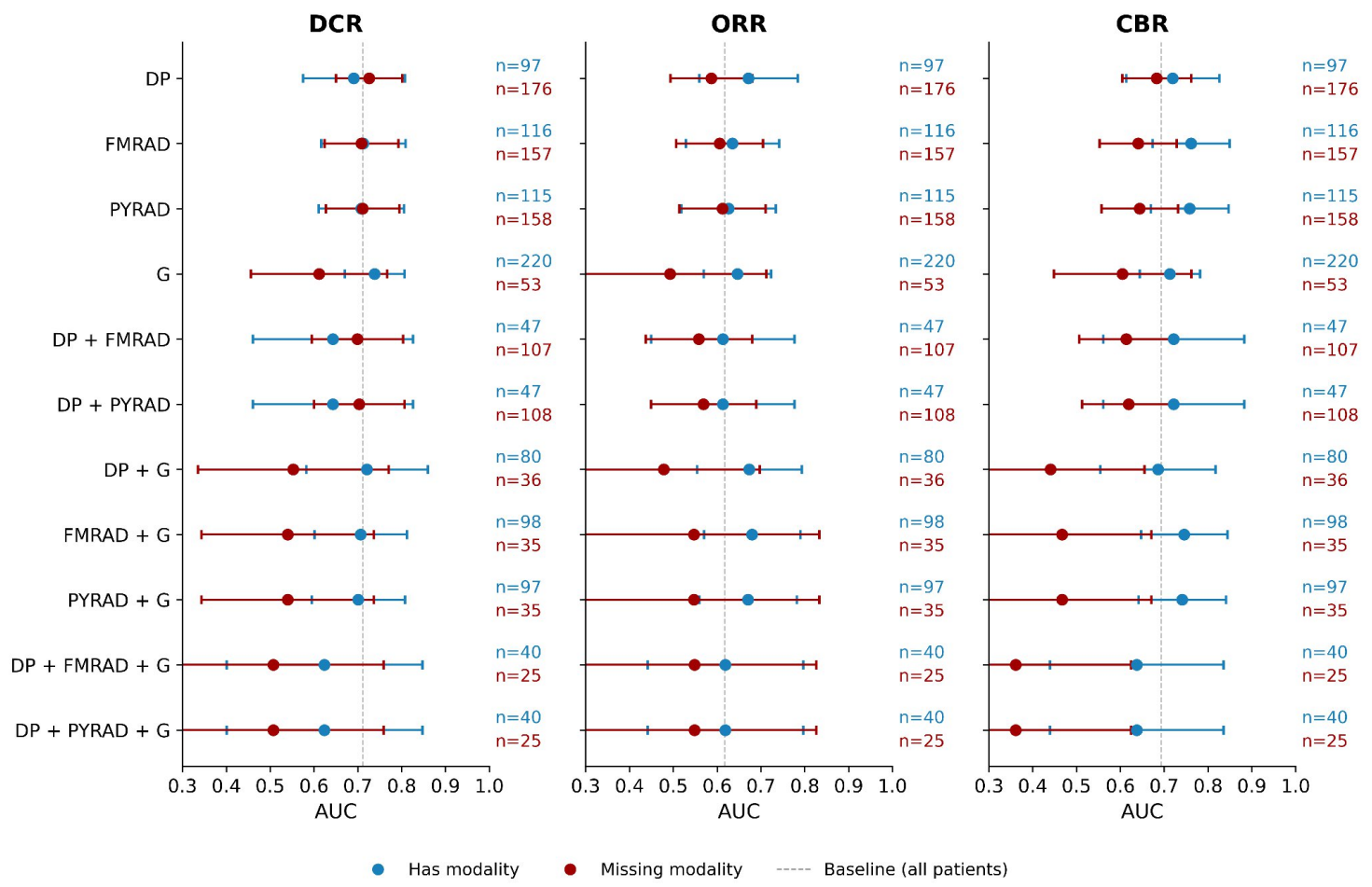

Figure 8. CB Model Performance on Response Outcomes by Data Availability, cohort 2

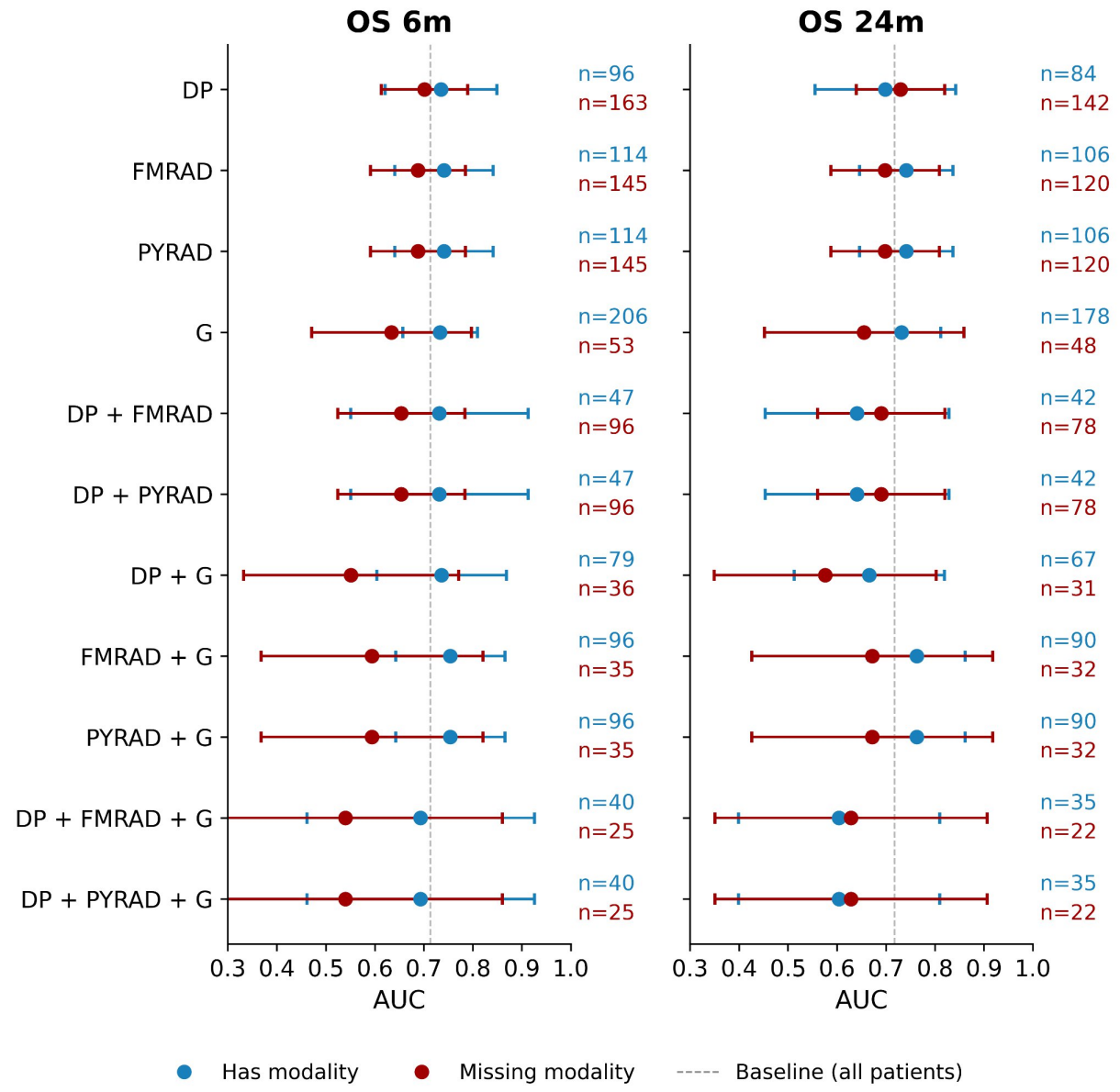

Figure 9. CB Model Performance on Survival Outcomes by Data Availability, cohort 2

Model Performance on Modality-Complete Cohort

To further determine whether the subset of modality-complete patients was particularly enriched, we tested the performance across all models trained on different data modality subsets on the subset of patients that had all data modalities available.

It is important to note that the subset of patients that have complete data is small (n=40), so it is difficult to come to confident conclusions regarding how well models use particular modalities. However, from this experiment, it is evident that the population of patients with complete data does not perform significantly better than the baseline across all models. In fact, this population tends to be slightly (but not significantly) worse than the baseline. These results confirm the findings of point one, that the population that has all modalities is not one in which the model performs disproportionally well.

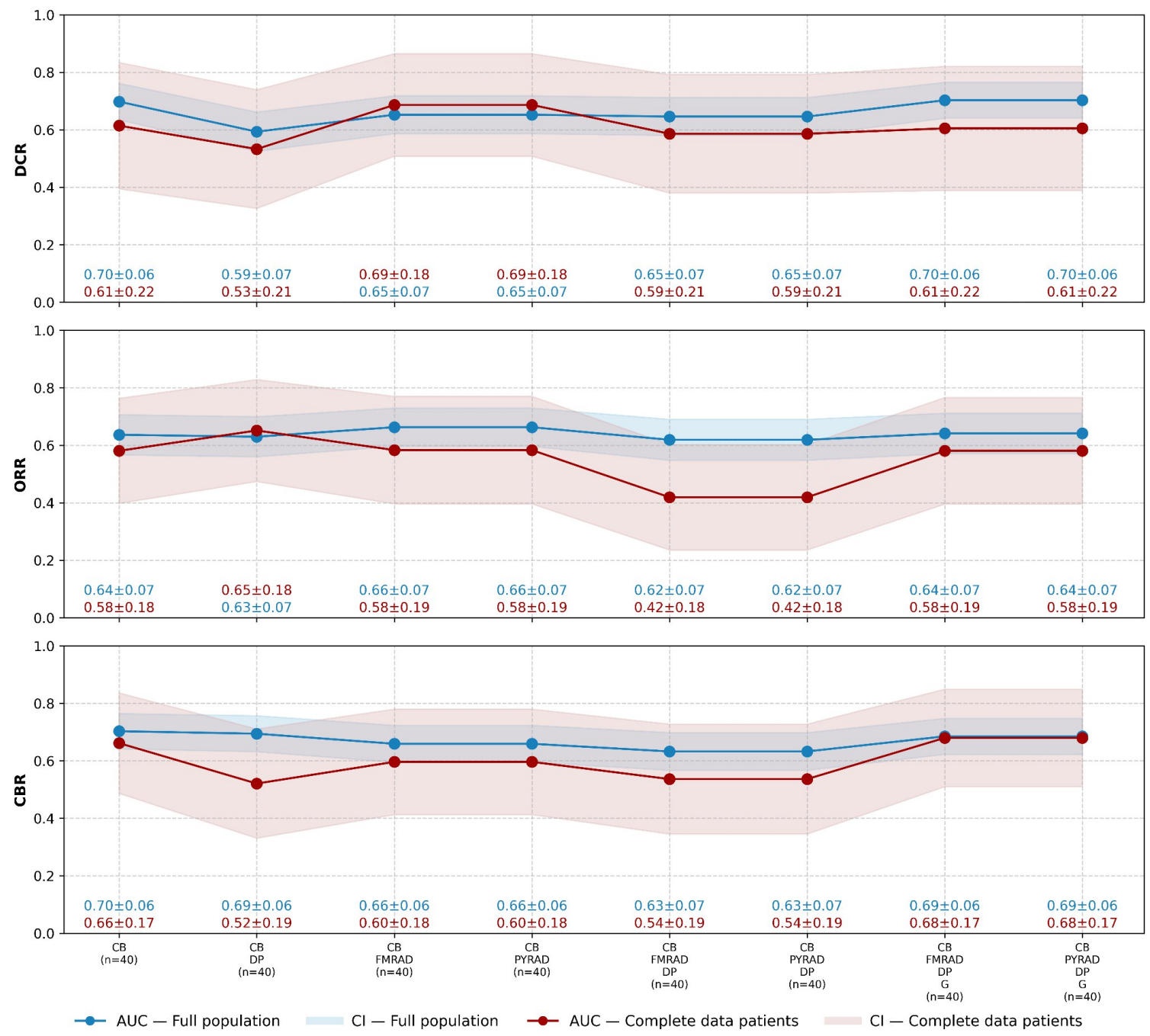

Figure 10. Model Performance on response outcomes in the complete-modality patient subset. Cohort 23

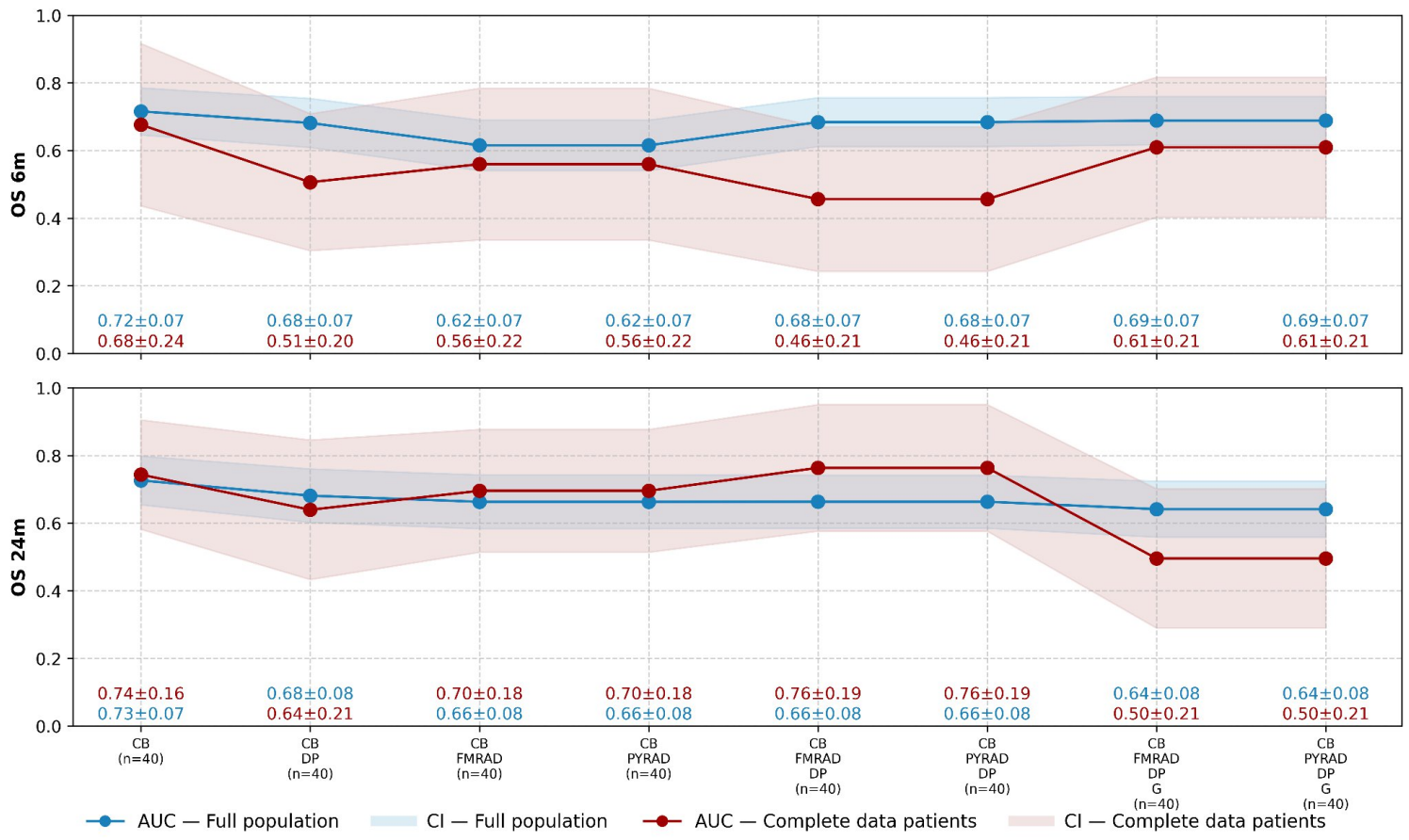

Figure 11. Model Performance on survival outcomes in the complete-modality patient subset. Cohort 23

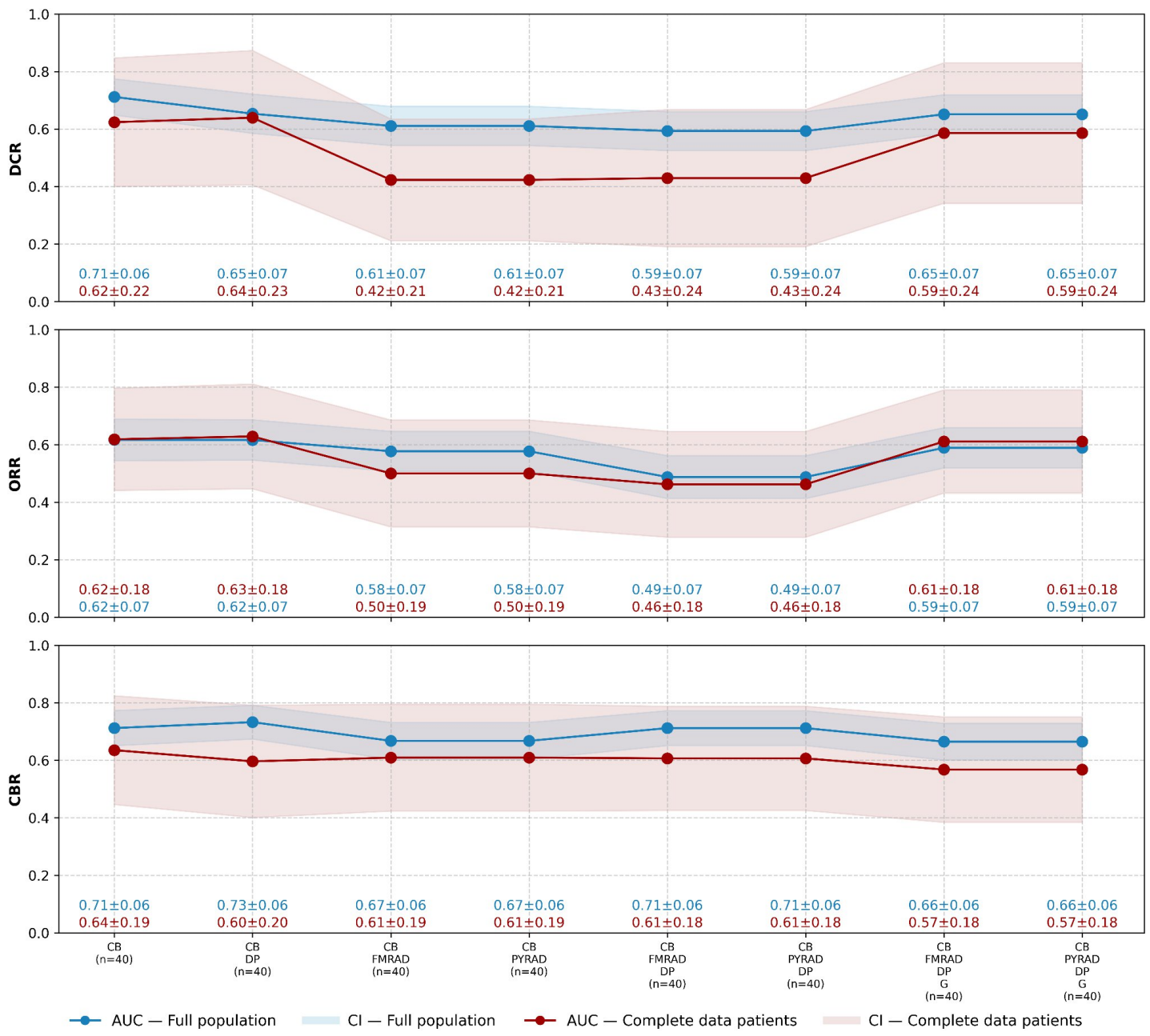

Figure 12. Model Performance on response outcomes in the complete-modality patient subset. Cohort 2

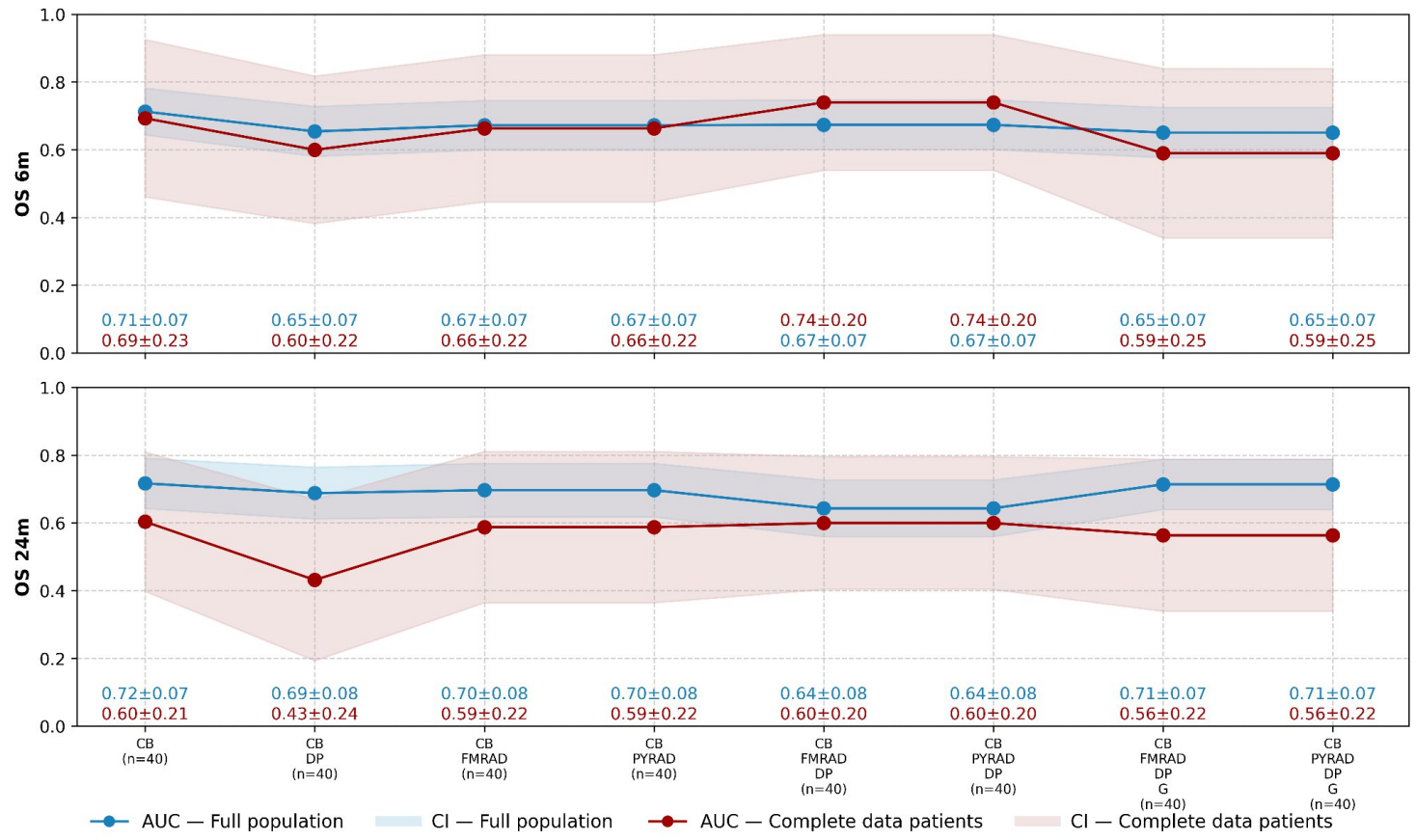

Figure 13. Model Performance on survival outcomes in the complete-modality patient subset. Cohort 2

Sensitivity Analysis with Modality Masking on Modality-Complete Patient Subset

To further examine whether the models trained on all data modalities were using a particular modality to improve predictions, we examined the performance of these models (using modalities CB + PYRAD/FMRAD + DP + GENOMICS) on the subset of patients that had complete data across all modalities by completely masking different combinations of modalities. In these plots, the dashed gray line represents the baseline model performance on all patients. The dashed blue line is the performance of the model on the subset of patients with complete data.

It is important to note that the subset of patients that have complete data is small (n=40), so it is difficult to come to confident conclusions regarding how well the model is utilizing particular modalities. It is challenging to interpret these data, which have been run on such a small set of patients, as they appear to be noisy. However, for most outcomes, the model's performance does not significantly change depending on which modalities are available (non-masked), suggesting that the model is not making full use of the modalities that are not CB on these 40 patients.

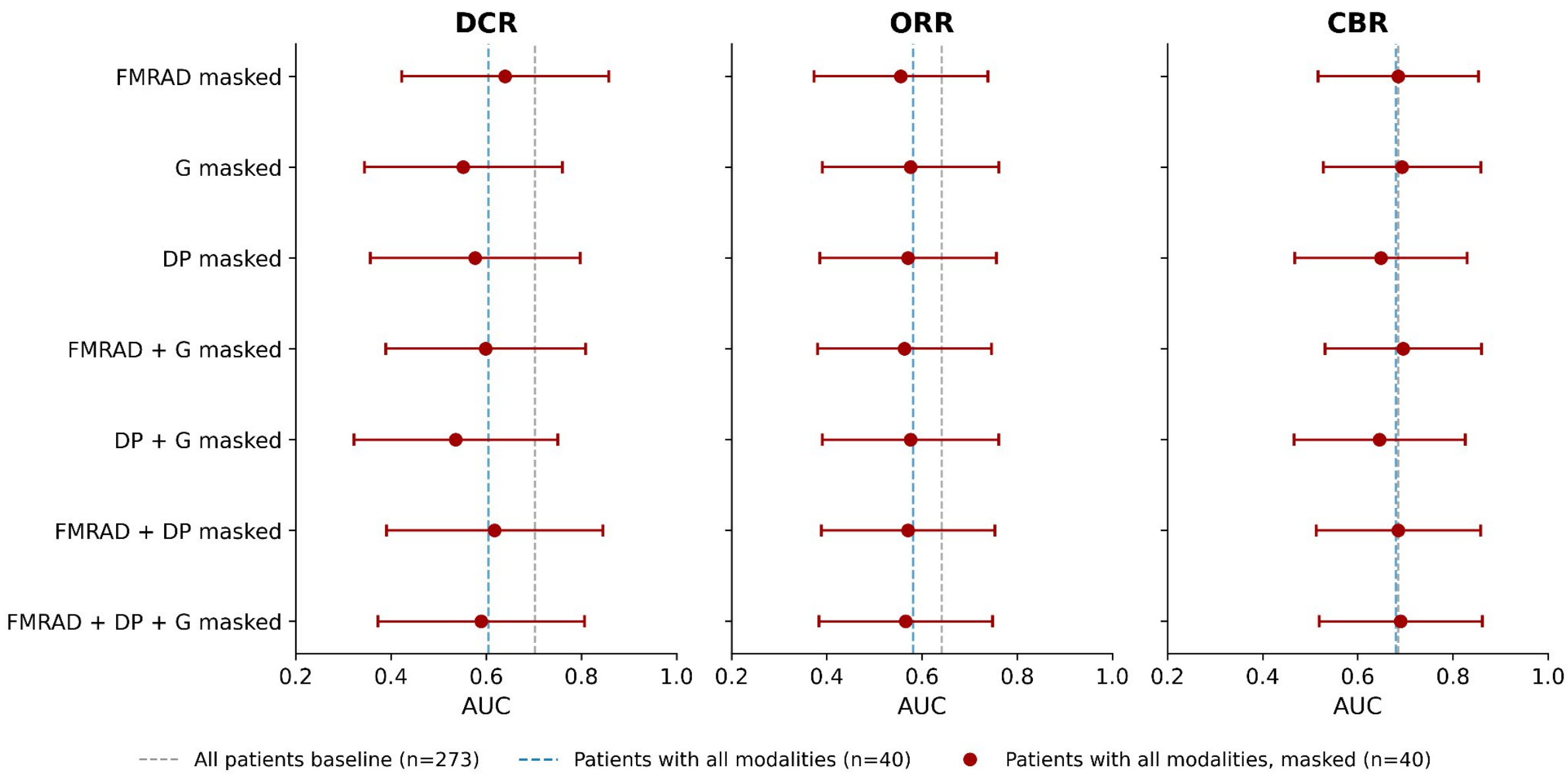

Figure 14. All-modality model performance on response outcomes (FMRAD variant) on the complete-modality patient subset with masked modalities. Cohort 23.

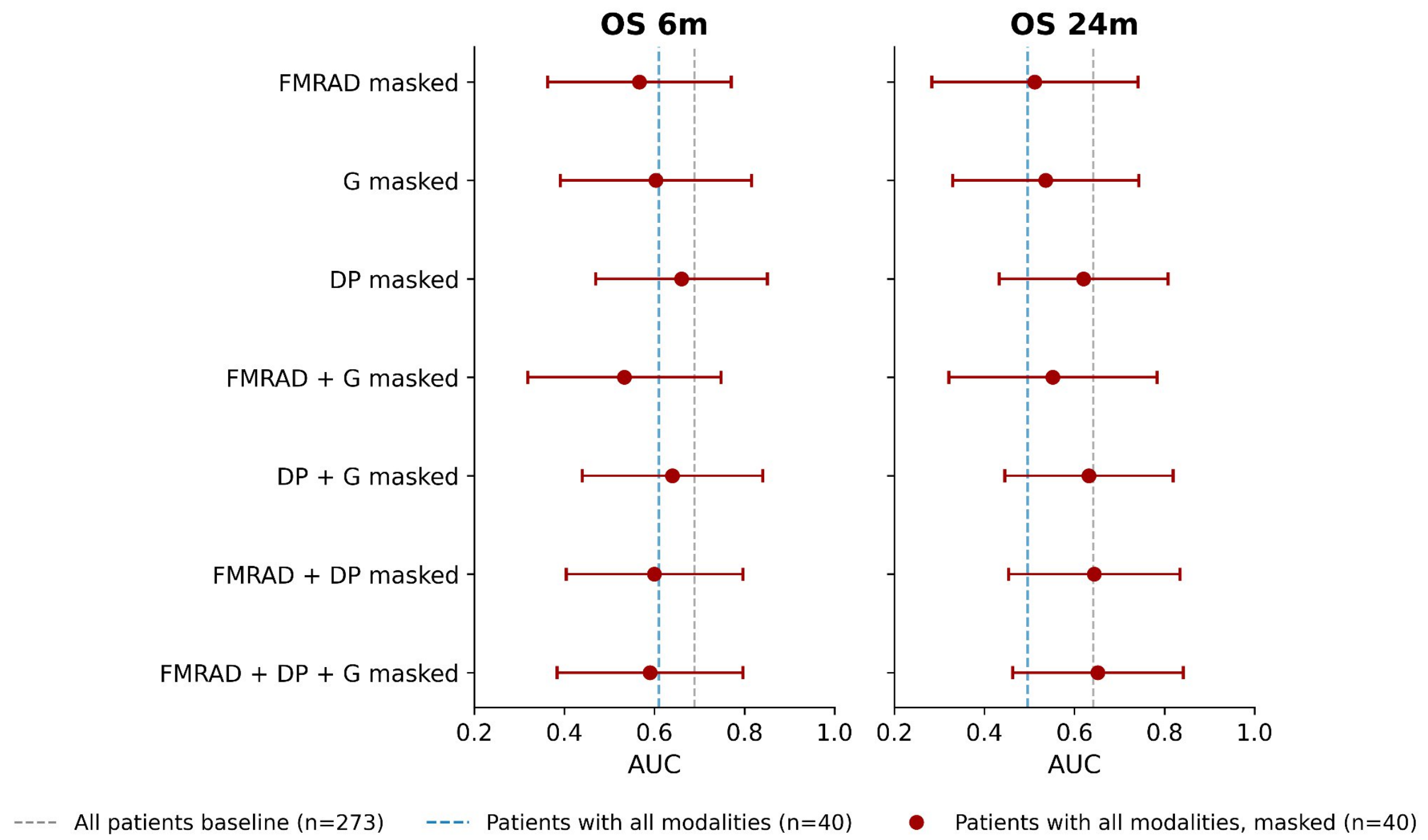

Figure 15. All-modality model performance on survival outcomes (FMRAD variant) on the complete-modality patient subset with masked modalities. Cohort 23.

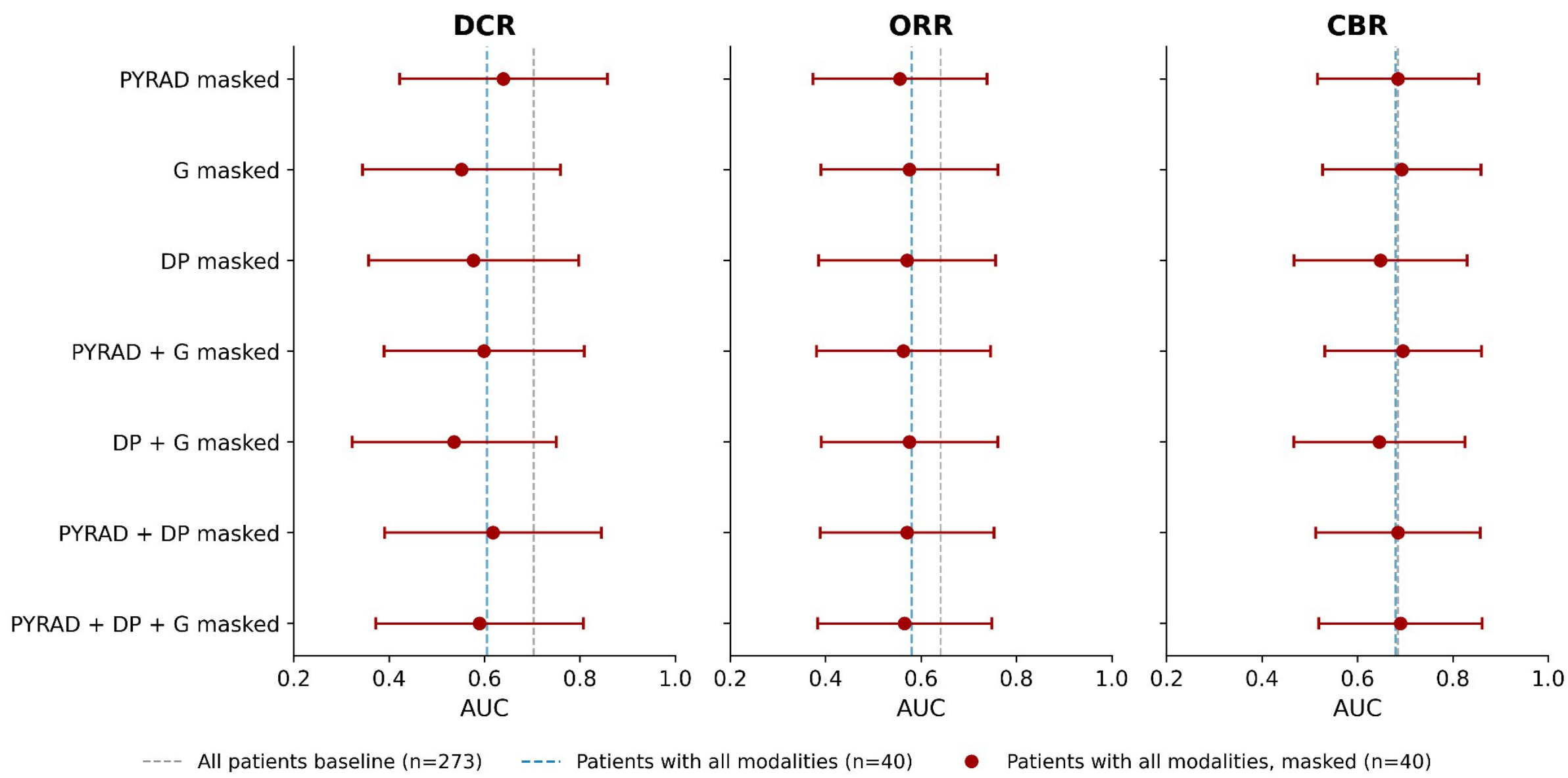

Figure 16. All-modality model performance on response outcomes (PYRAD variant) on the complete-modality patient subset with masked modalities. Cohort 23.

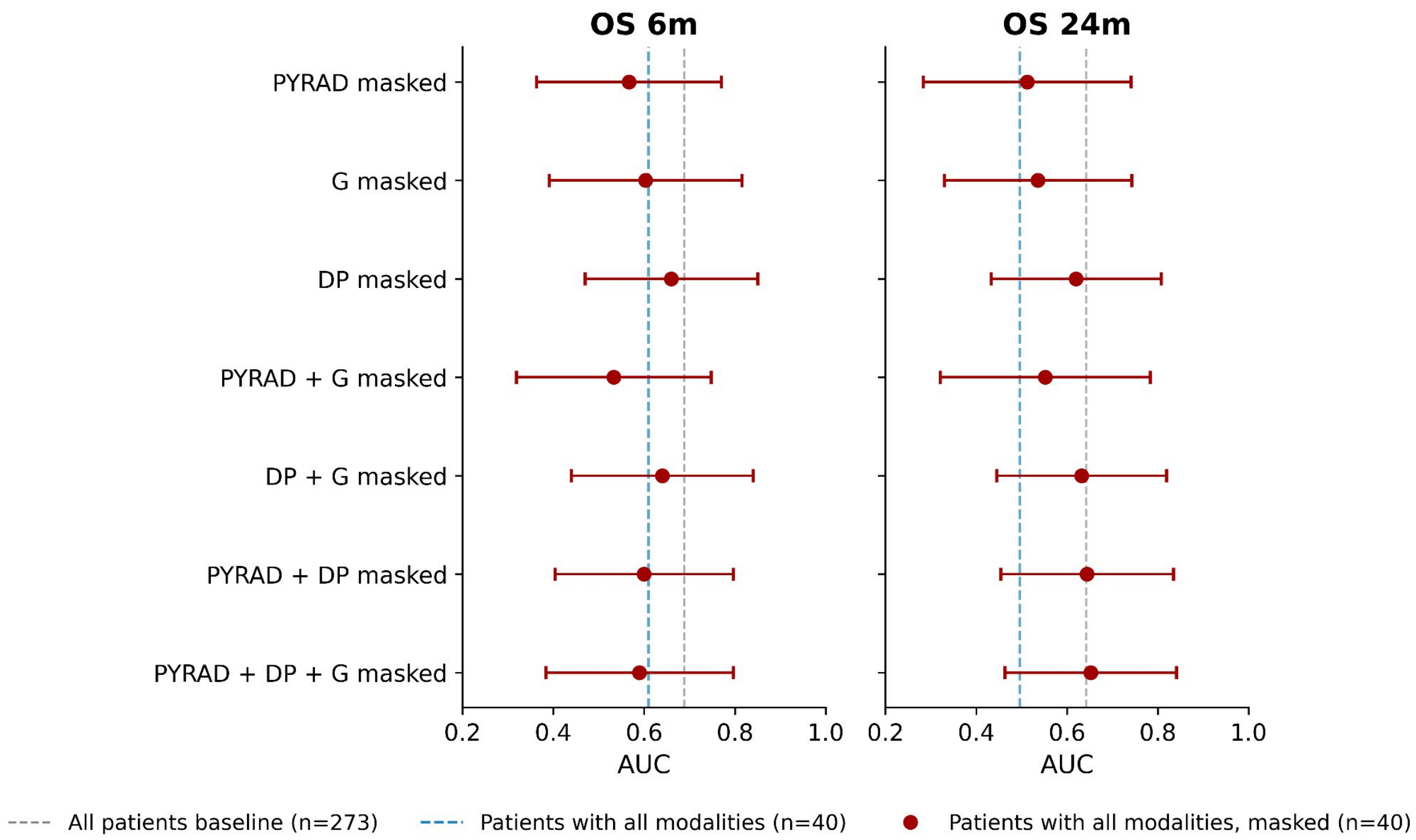

Figure 17. All-modality model performance on survival outcomes (PYRAD variant) on the complete-modality patient subset with masked modalities. Cohort 23.

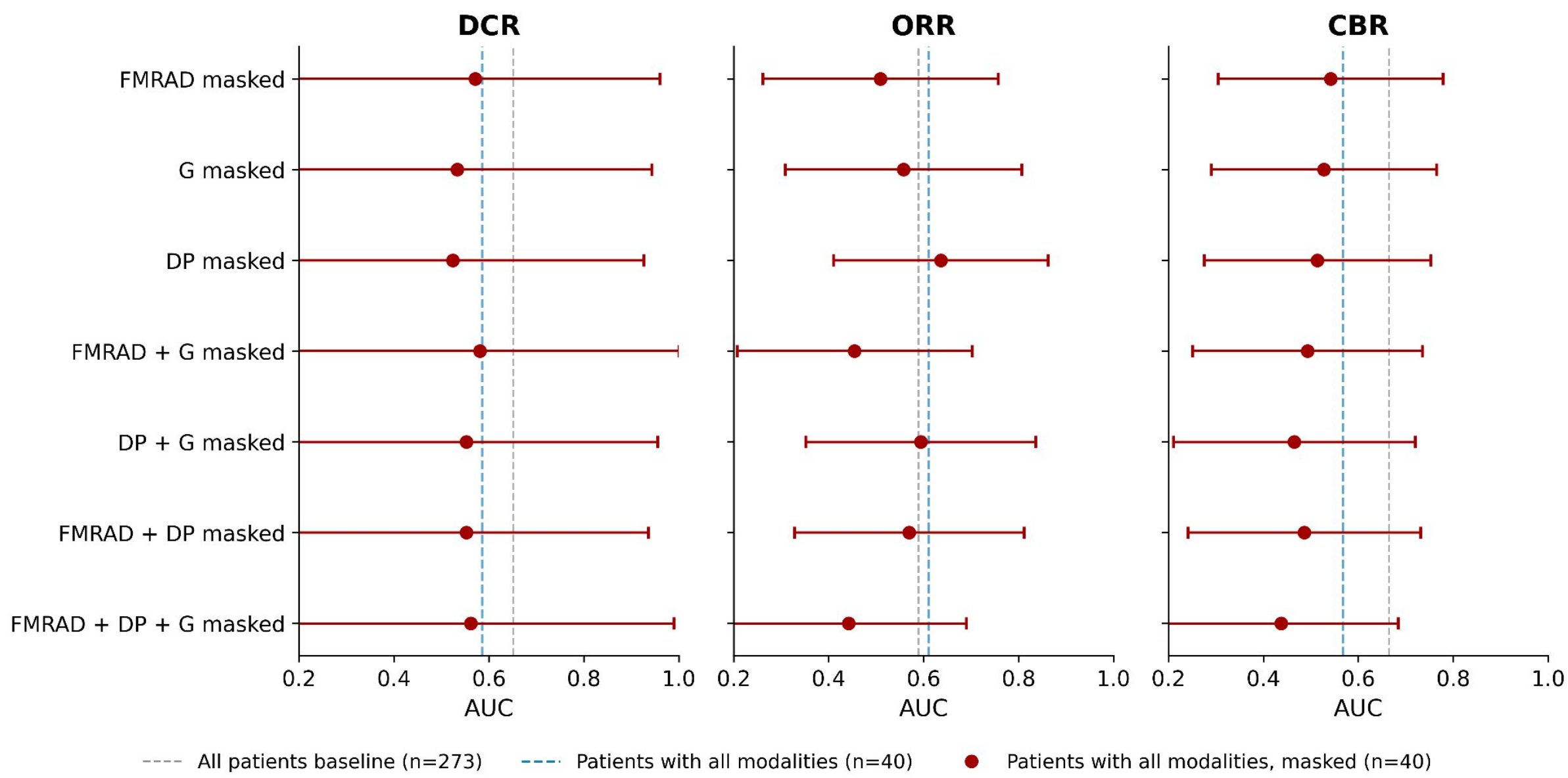

Figure 18. All-modality model performance on response outcomes (FMRAD variant) on the complete-modality patient subset with masked modalities. Cohort 2.

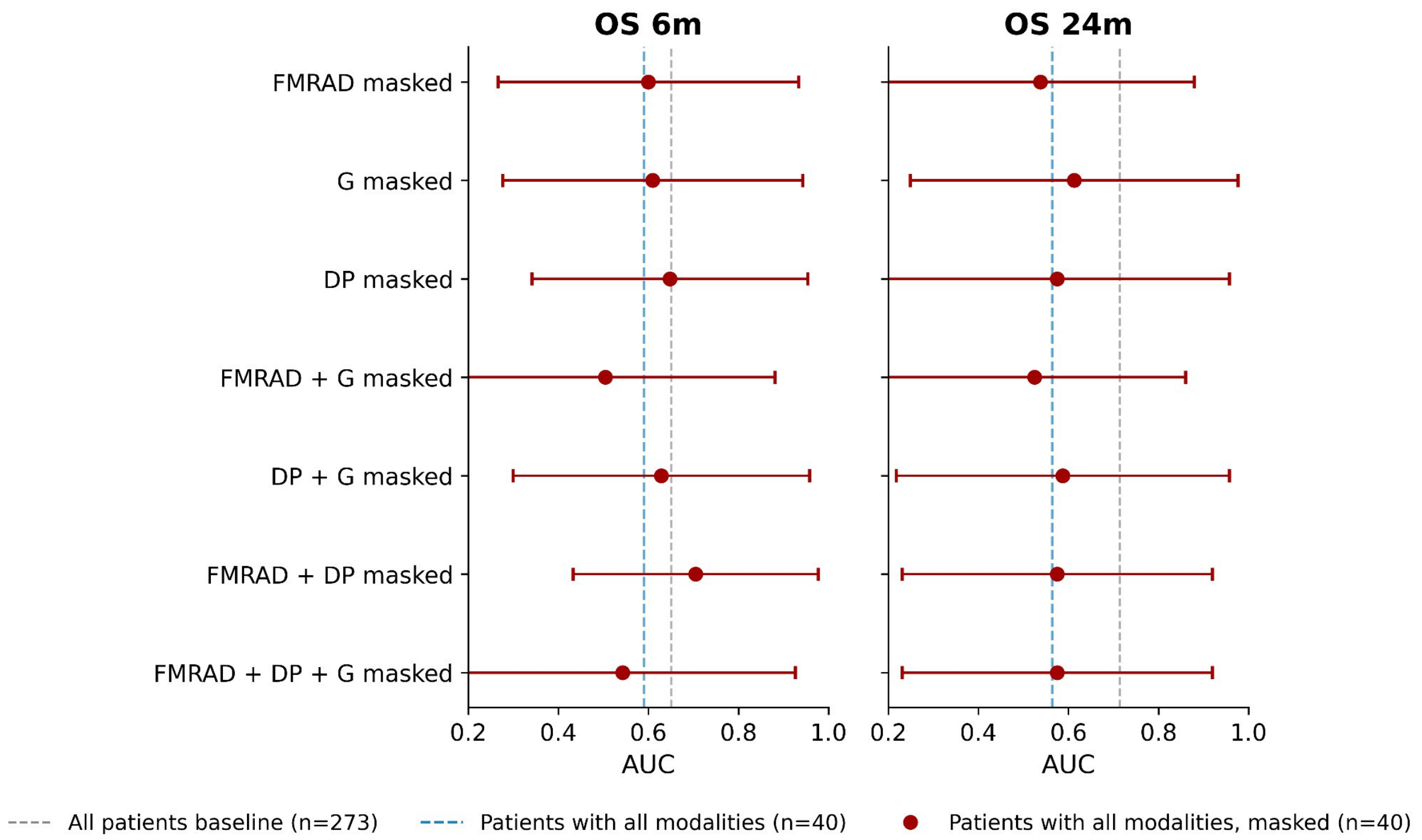

Figure 19. All-modality model performance on survival outcomes (FMRAD variant) on the complete-modality patient subset with masked modalities. Cohort 2.

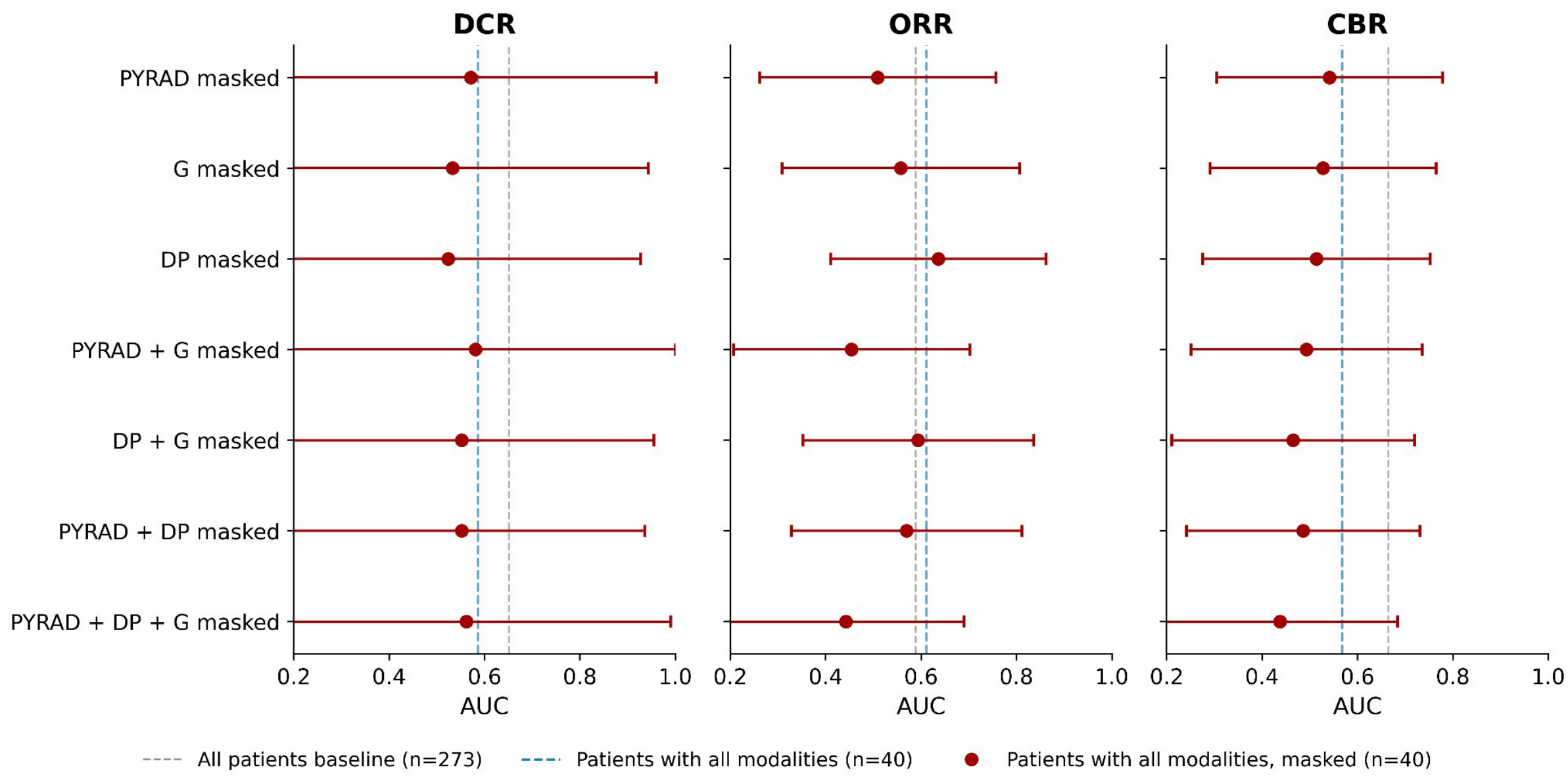

Figure 20. All-modality model performance on response outcomes (PYRAD variant) on the complete-modality patient subset with masked modalities. Cohort 2.

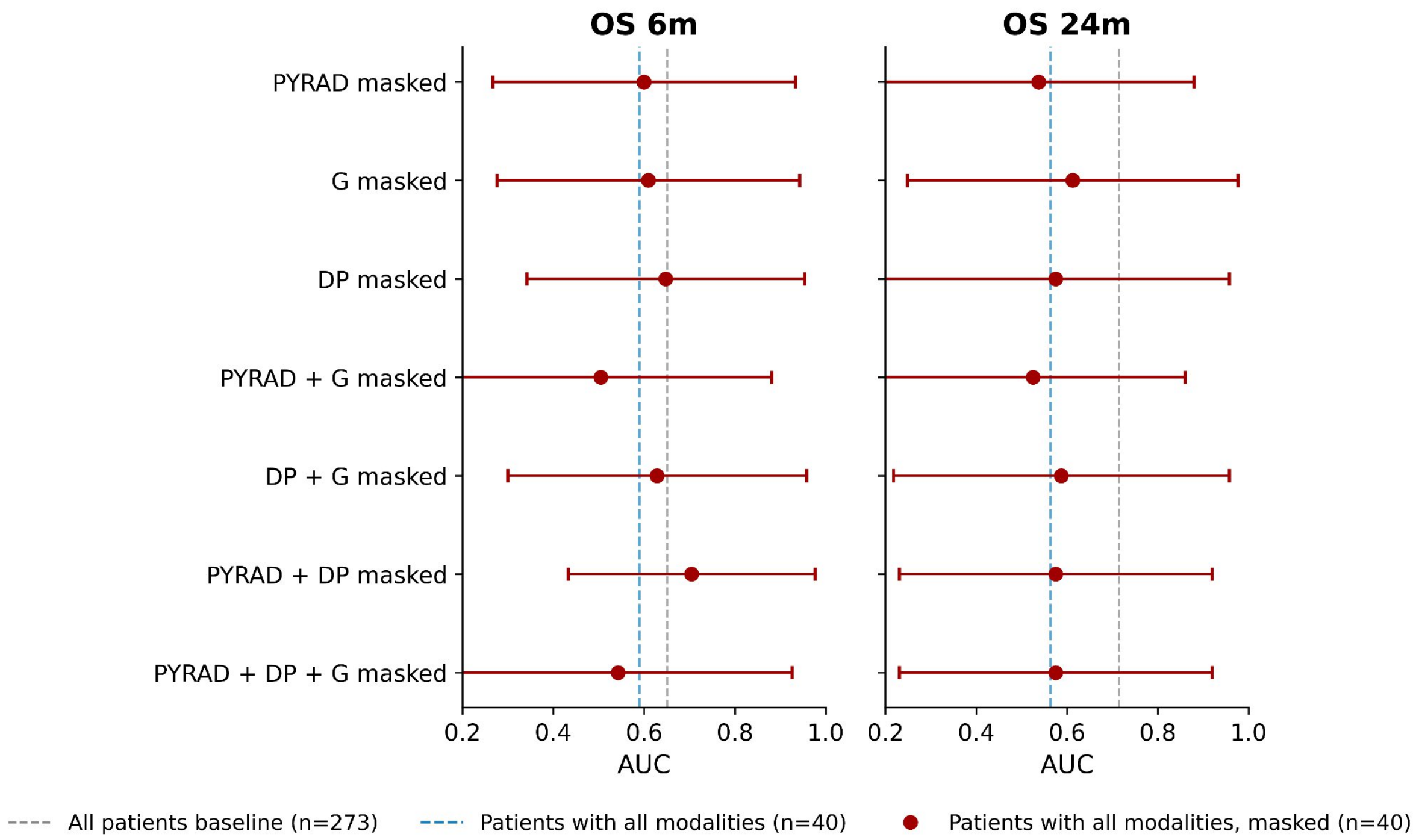

Figure 21. All-modality model performance on survival outcomes (PYRAD variant) on the complete-modality patient subset with masked modalities. Cohort 2.

Conclusions

Across experiments, the modality-complete patient subset does not demonstrate significant enrichment compared to the entire cohort. The CB-only model generally performs better on patients with additional modalities available, though confidence intervals largely overlap. Notably, patients lacking genomics data and those with no modalities beyond CB represent subpopulations where model performance is weakest. Sensitivity analysis further suggests that the all-modality models do not fully leverage non-CB modalities in the modality-complete subset. Together, these findings indicate that while additional modalities show directional benefit, their incremental contribution remains inconclusive at current cohort sizes.

Section S3.

Model Comparison with TITAN Digital Pathology Features

To test the sensitivity of our results to the digital pathology feature extractor used, we re-extracted 768-dimensional slide embedding features using TITAN (Ding et al., Nature Medicine 2025), a multimodal whole-slide foundation model pretrained using self-supervised learning on digital pathology whole slide images and vision-language alignment with pathology reports. We compared our results using Prov-GigaPath digital pathology features to those extracted by TITAN across all three response outcomes and modality combinations.

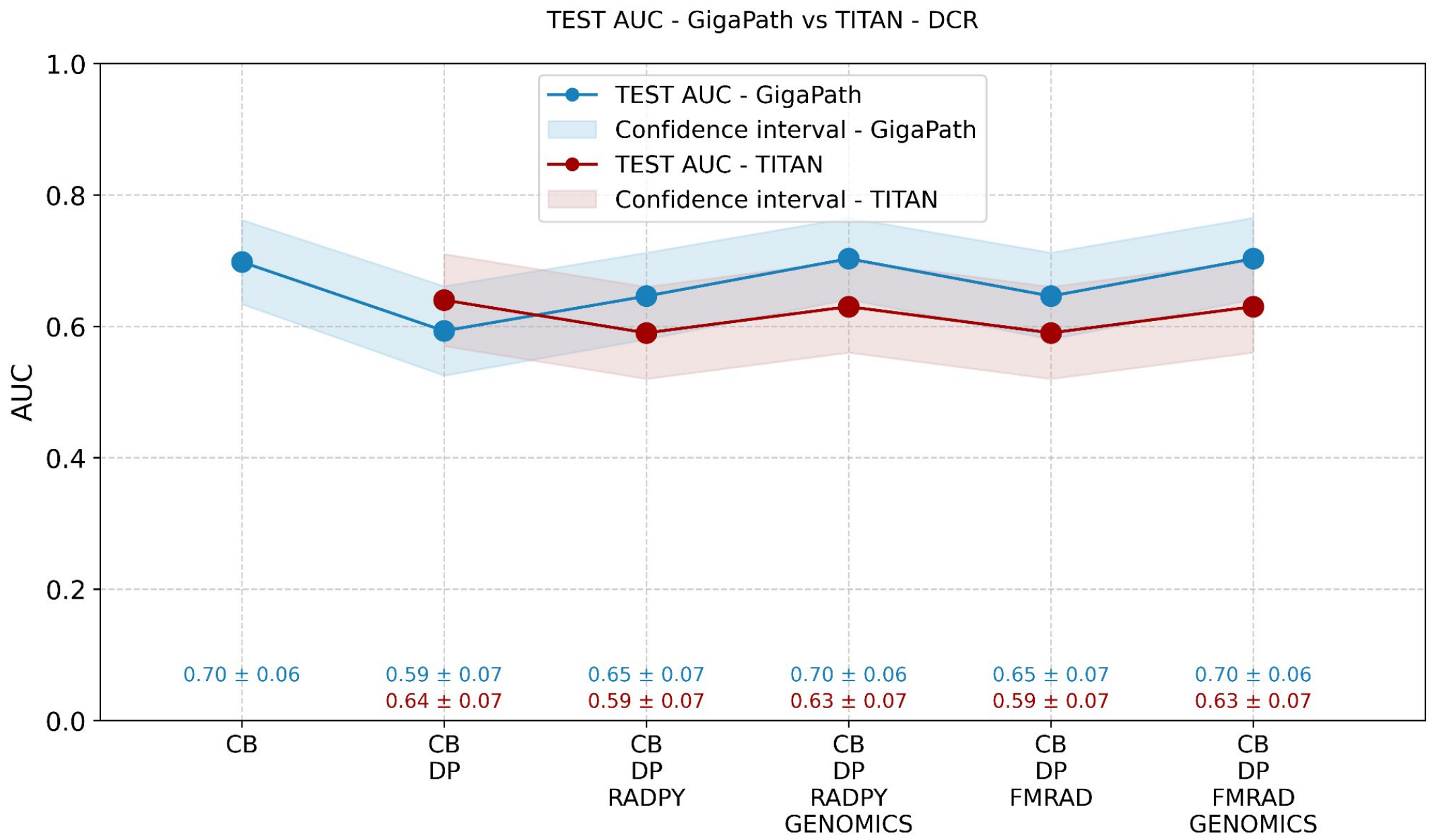

Figure 22. DLIF results in our original Prov-GigaPath-based model vs. TITAN-based model in Test for outcome DCR.

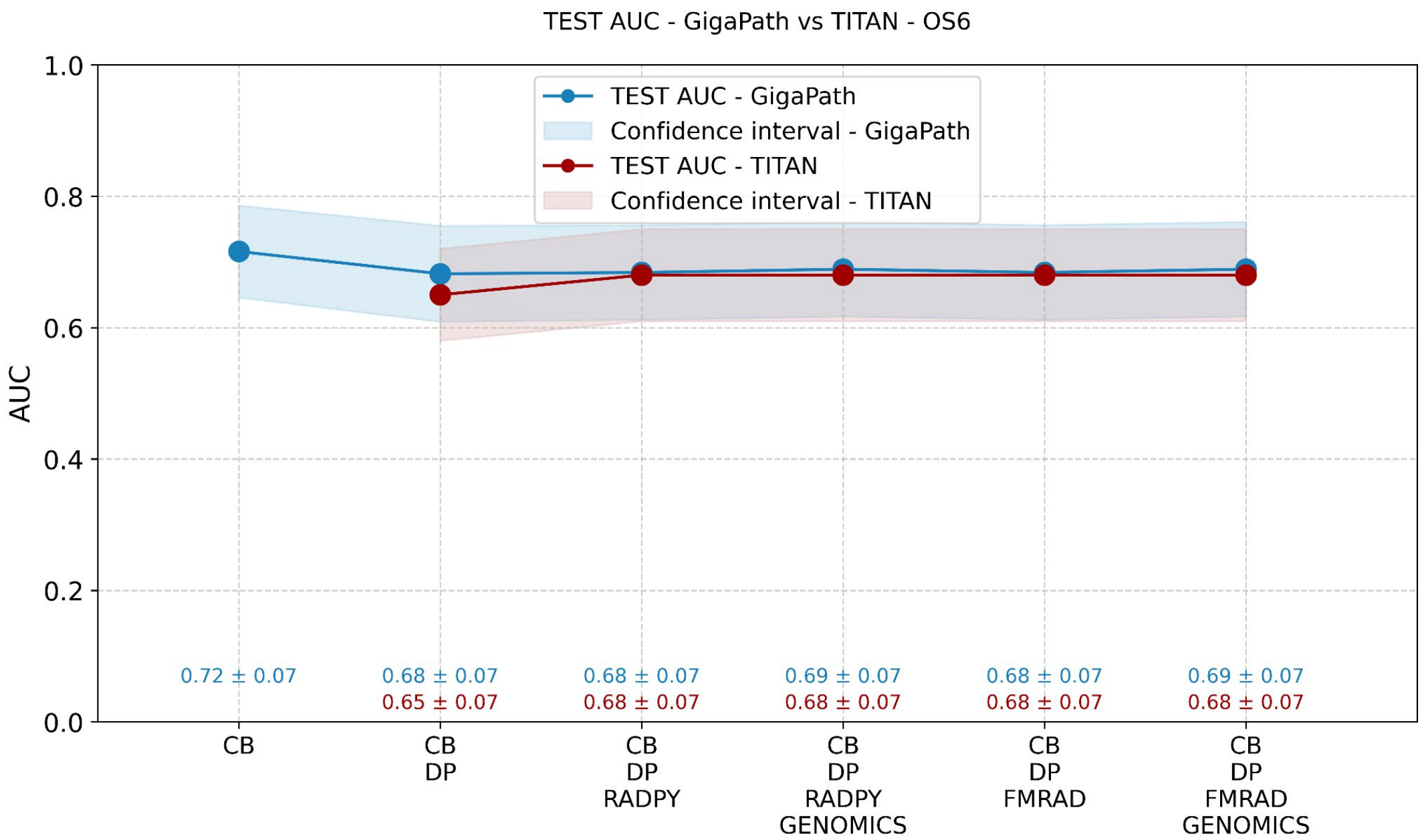

Figure 23. DLIF results in our original Prov-GigaPath-based model vs. TITAN-based model in Test for outcome OS6.

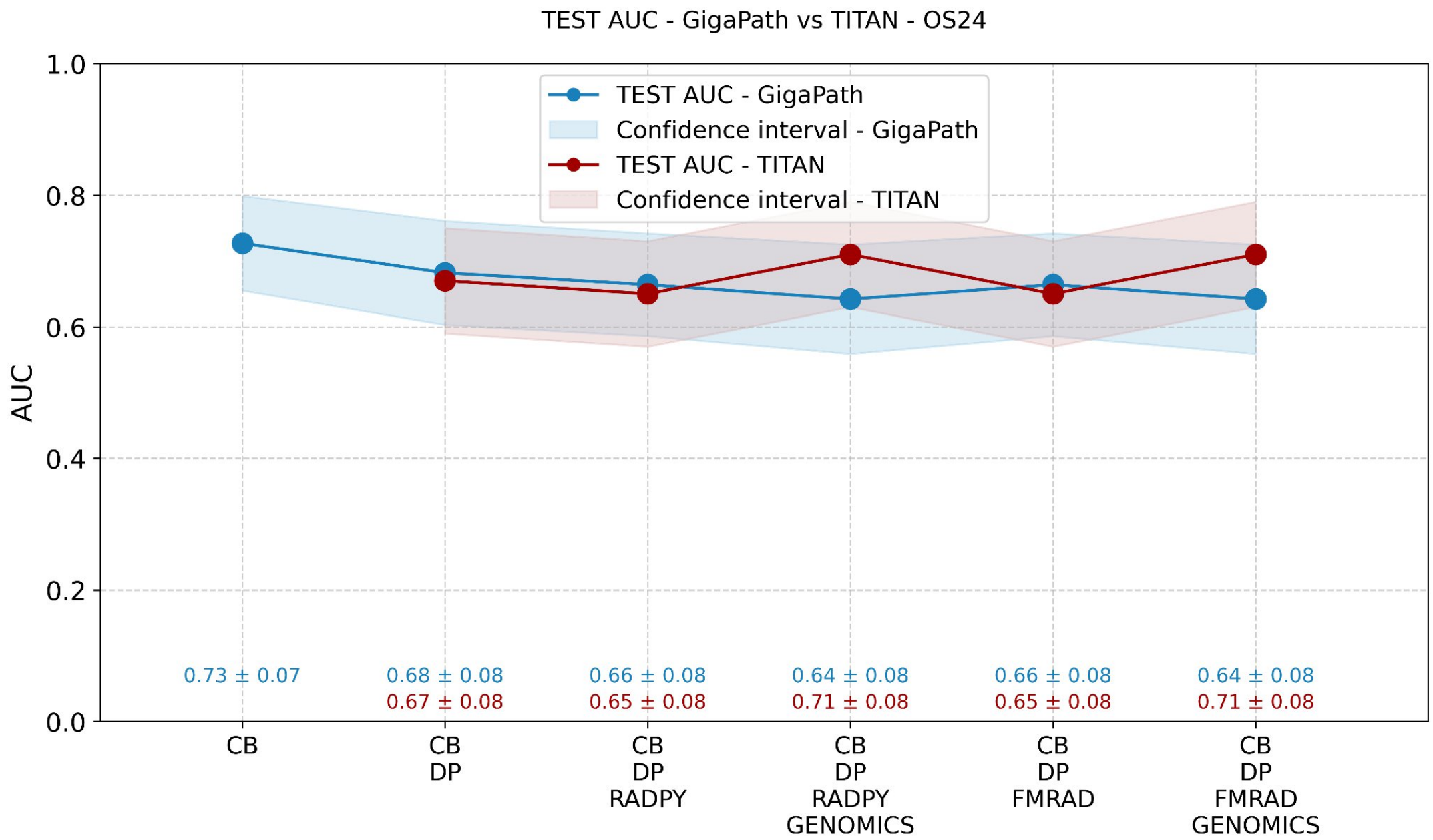

Figure 24. DLIF results in our original Prov-GigaPath-based model vs. TITAN-based model in Test for outcome OS24.

Conclusions

Two observations emerge from this comparison. First, TITAN and Prov-GigaPath features yield equivalent performance across all DP-containing configurations and response outcomes, with fully overlapping confidence intervals. Second, consistent with our primary analysis, no DP-containing configuration, regardless of the feature extractor used, improves over the CB-only baseline.

Supplementary References

1. Xu, H., Usuyama, N., Bagga, J. et al. A whole-slide foundation model for digital pathology from real-world data. Nature 630, 181–188 (2024). https://doi.org/10.1038/s41586-024-07441-w

2. Ding, T., Wagner, S.J., Song, A.H. et al. A multimodal whole-slide foundation model for pathology. Nat Med 31, 3749–3761 (2025). https://doi.org/10.1038/s41591-025-03982-3

Supplementary Information 5

This supplementary document presents two sections.. The first section (**S1**) provides additional investigations on the MLEF model, including confusion matrices, center prediction analysis, calibration analysis per center and Shapley (SHAP) value decomposition stratified by center and sex, motivated by the statistically significant performance differences across groups identified in the fairness analysis. The second section (**S2**) reports a fairness analysis of the DLIF model, evaluating True Positive Rate (TPR) and False Positive Rate (FPR) stratified by sex, center, and race

Section 1

Table 1. Confusion matrix of CB-only MLEF model OS6 test set, split by center.

Dataset	TN	FP	FN	TP
Overall test (n(0)=77, n(1)=182)	49 (18.9%)	28 (10.8%)	64 (24.7%)	118 (45.6%)
GHD (n(0)=23, n(1)=42)	10 (15.4%)	13 (20%)	16 (24.6%)	26 (40%)
INT (n(0)=37, n(1)=64)	27 (26.7%)	10 (9.9%)	27 (26.7%)	37 (36.6%)
MH (n(0)=2, n(1)=24)	1 (3.8%)	1 (3.8%)	1 (3.8%)	23 (88.5%)
SZMC (n(0)=5, n(1)=31)	2 (5.6%)	3 (8.3%)	12 (33.3%)	19 (52.8%)
VHIO (n(0)=10, n(1)=21)	9 (29%)	1 (3.2%)	8 (25.8%)	13 (41.9%)

TN - True Negatives, FP - False Positives, FN - False Negatives, TP - True Positives

Comment: Although MH center's performance is significantly better than the other centers, it suffers from a low number of patients with OS < 6 (class 0, only 2 patients). This can affect the model stability and highlights the importance of having sufficient data from all classes to ensure reliable evaluation.

a)

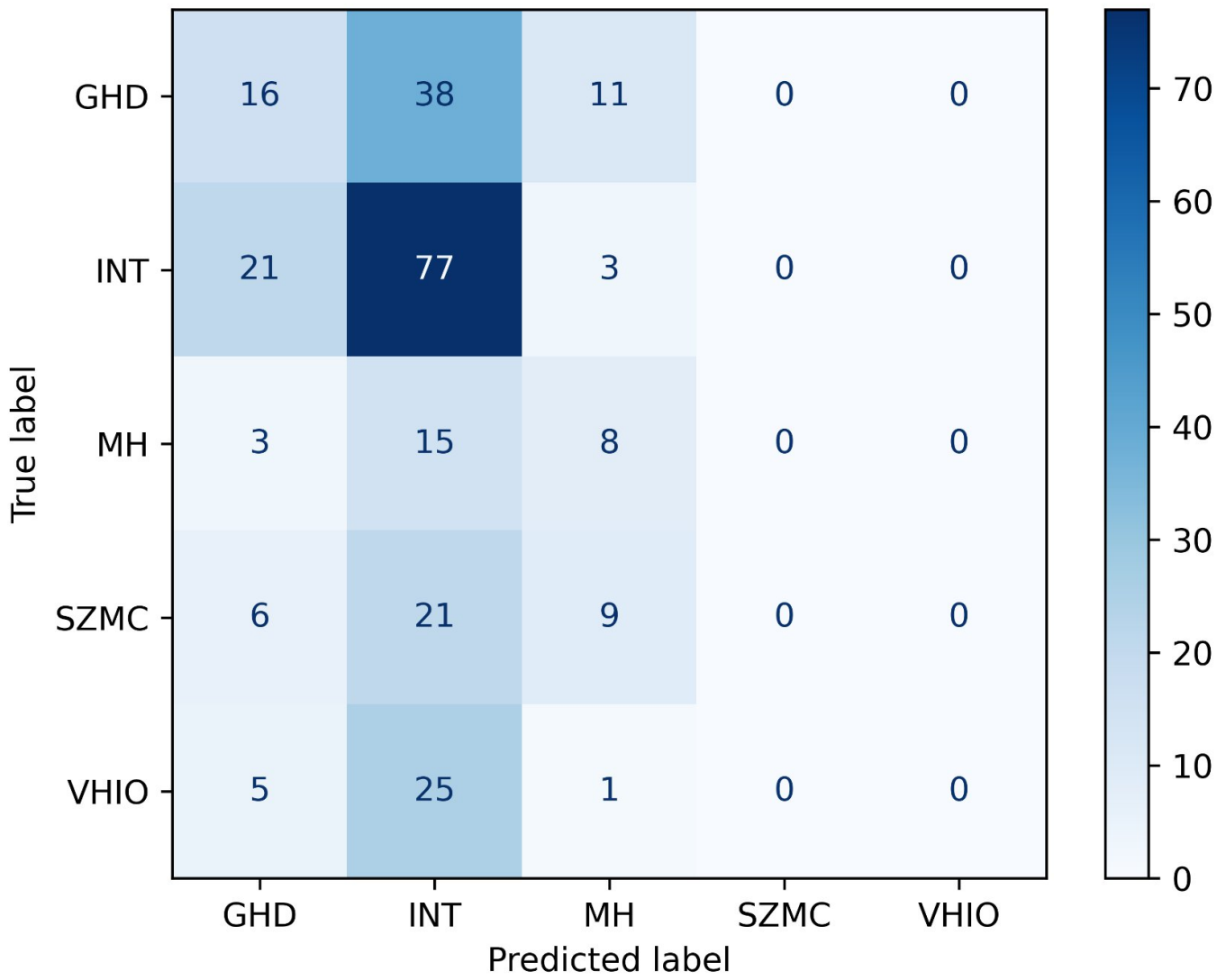

b)

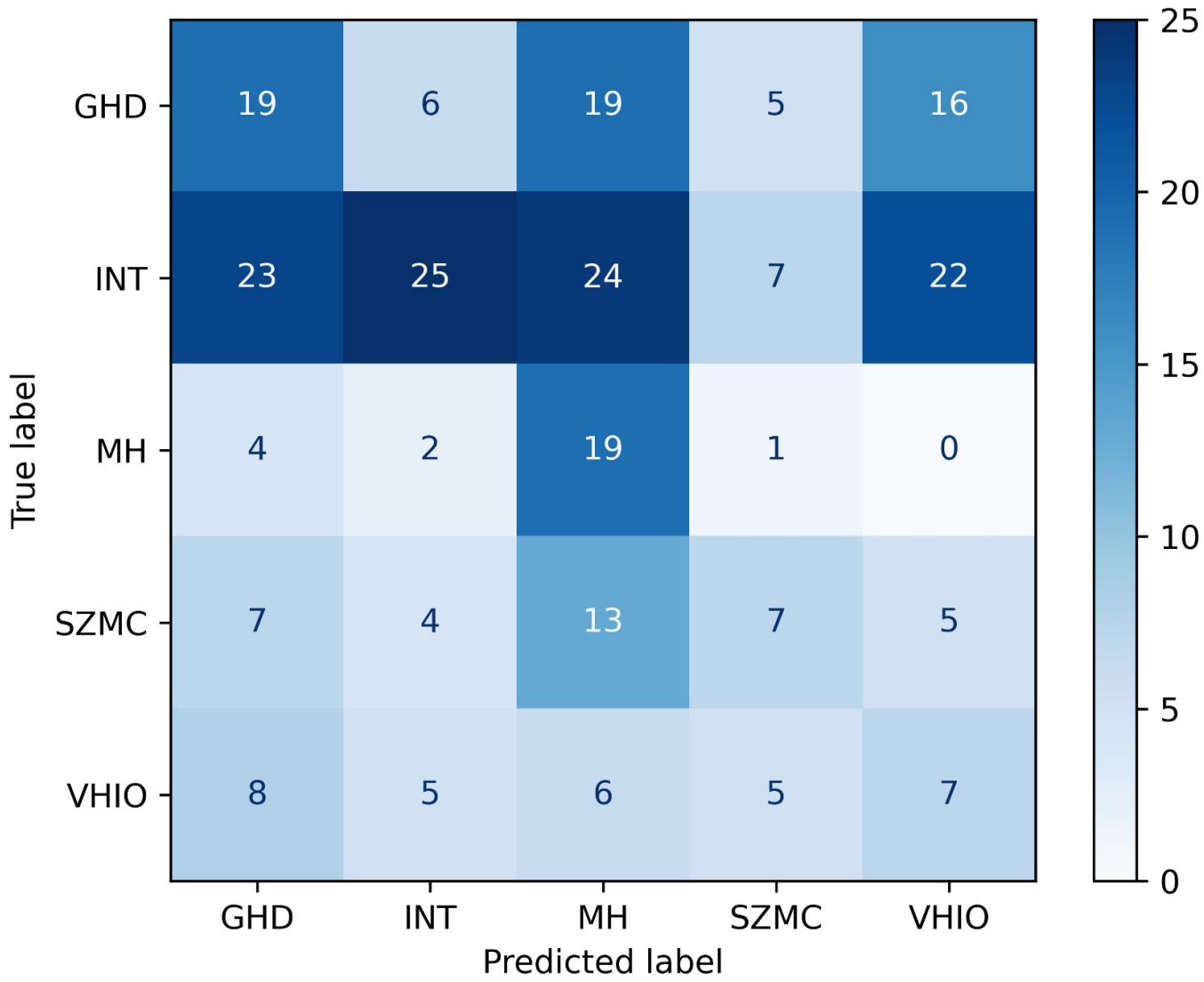

Figure 1. Center Prediction model for MLEF. a) Confusion matrix of the multiclass Logistic Regression model on the OS6 CB-only test set; b) Confusion matrix of the balanced multiclass Logistic Regression model on the OS6 CB-only test set.

The model cannot distinguish between centers, indicating that the clinical features may not have sufficient discriminative power to reliably predict the center. This further suggests that the observed differences in TPR are not driven by the model’s ability to distinguish between centers, but rather by differences in data distribution.

Table 2. OS6 MLEF calibration analysis per center.. This table reports per-center calibration results for a MLEF CB-only (LR C23) model, where the intercept was recalibrated using the LOCO held-out set of each center and evaluated on that center's test subset.

center	intercept	TPR before	TPR after	FPR before	FPR after	spec before	spec after
GHD	0.272	0.62	0.74	0.565	0.70	0.435	0.30
INT	0.602	0.58	0.89	0.270	0.68	0.730	0.32
MH	0.987	0.96	1	0.500	1	0.500	0
SZMC	1.597	0.61	1	0.600	1	0.400	0
VHIO	0.856	0.62	1	0.100	1	0.900	0

Comment: the OS6 MLEF center-specific calibration analysis showed substantial variability across centers. Recalibrated intercepts ranged from 0.272 (GHD) to 1.597 (SZMC). For MH, SZMC, and VHIO, post-calibration performance collapsed (TPR=1, FPR=1, specificity=0), which is likely an artifact of the extremely small number of negative samples (OS <6 months) in the LOCO test sets; under these conditions, recalibration led the model to predict all samples as positive. In contrast, for GHD and INT, where class imbalance was less extreme, recalibration did not improve performance but rather shifted the decision boundary, resulting in a substantial increase in FPR (0.565→0.70 and 0.270→0.68) and a corresponding drop in specificity (0.435→0.30 and 0.730→0.32), despite an increase in TPR. Overall, these findings suggest that center-level recalibration in small and imbalanced cohorts can be unstable and may degrade performance, highlighting the need for larger, more balanced datasets and predefined calibration strategies for reliable site-specific implementation.

Table 3. SHAP analysis across the centres for MLEF. Mean SHAP feature values per center across the OS 6 test set patients.There is a significant difference in the SHAP values of ECOG PS between MH and the other centers. This means that, for MH, the model relies heavily on this variable for prediction.

Feature/Center	GHD	INT	MH	SZMC	VHIO
Bone Mets at IO start	0.02	-0.02	0.04	0.02	-0.01
ECOG PS	-0.05	-0.09	0.32	0.10	-0.03
Liver Mets at IO start	-0.01	-0.001	-0.002	0.02	0.01
Parenchymal Brain Mets at IO start	0.002	-0.01	-0.003	-0.004	-0.008
Smoking current	-0.008	0.004	-0.008	-0.01	0.001
Smoking former	0.001	-0.001	0.001	0.003	-0.0003
Smoking never	0.0002	-0.002	-0.01	0.002	0.001

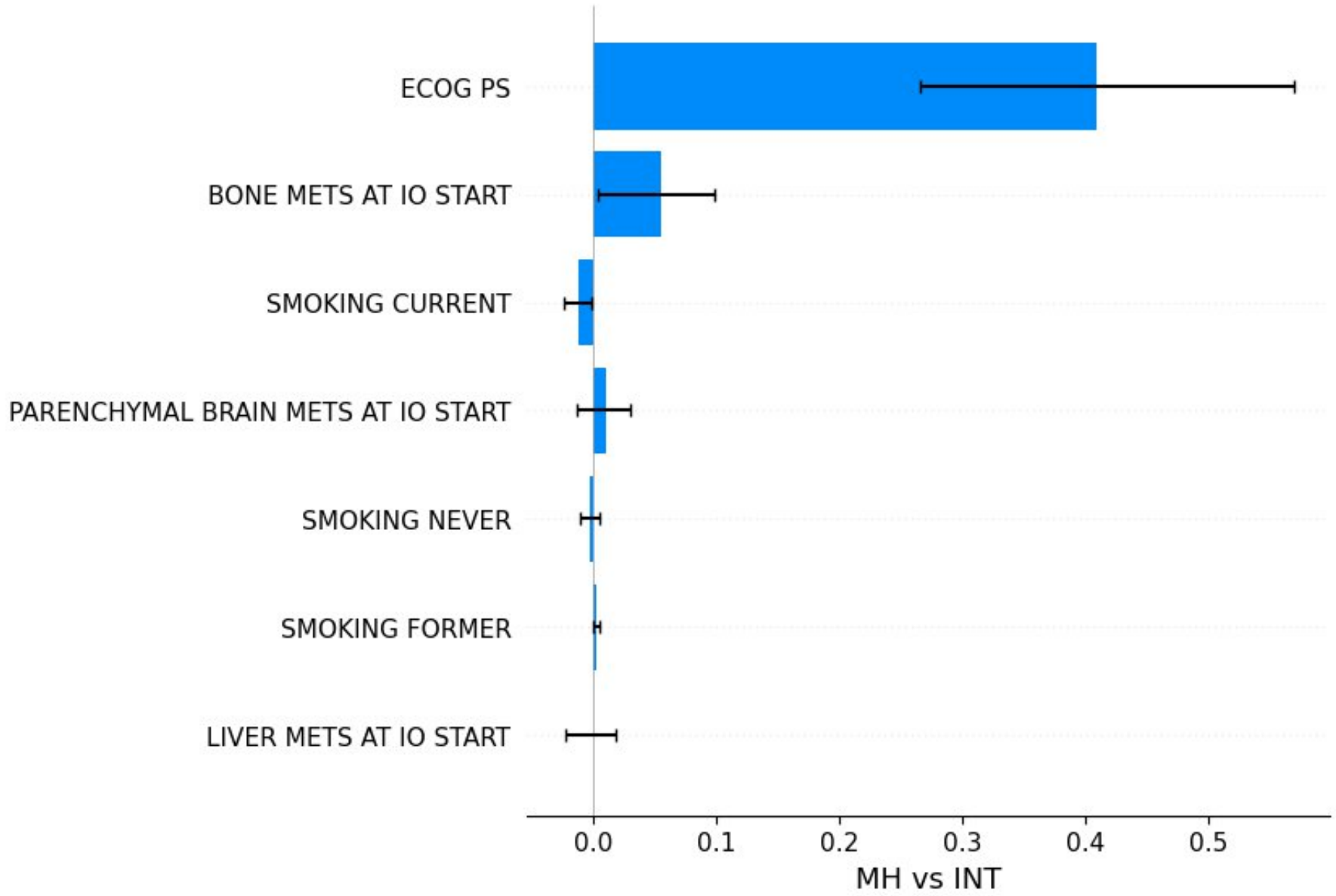

Figure 2. Mean SHAP features differences between MH and INT centers in the OS6 test set patients.

a)

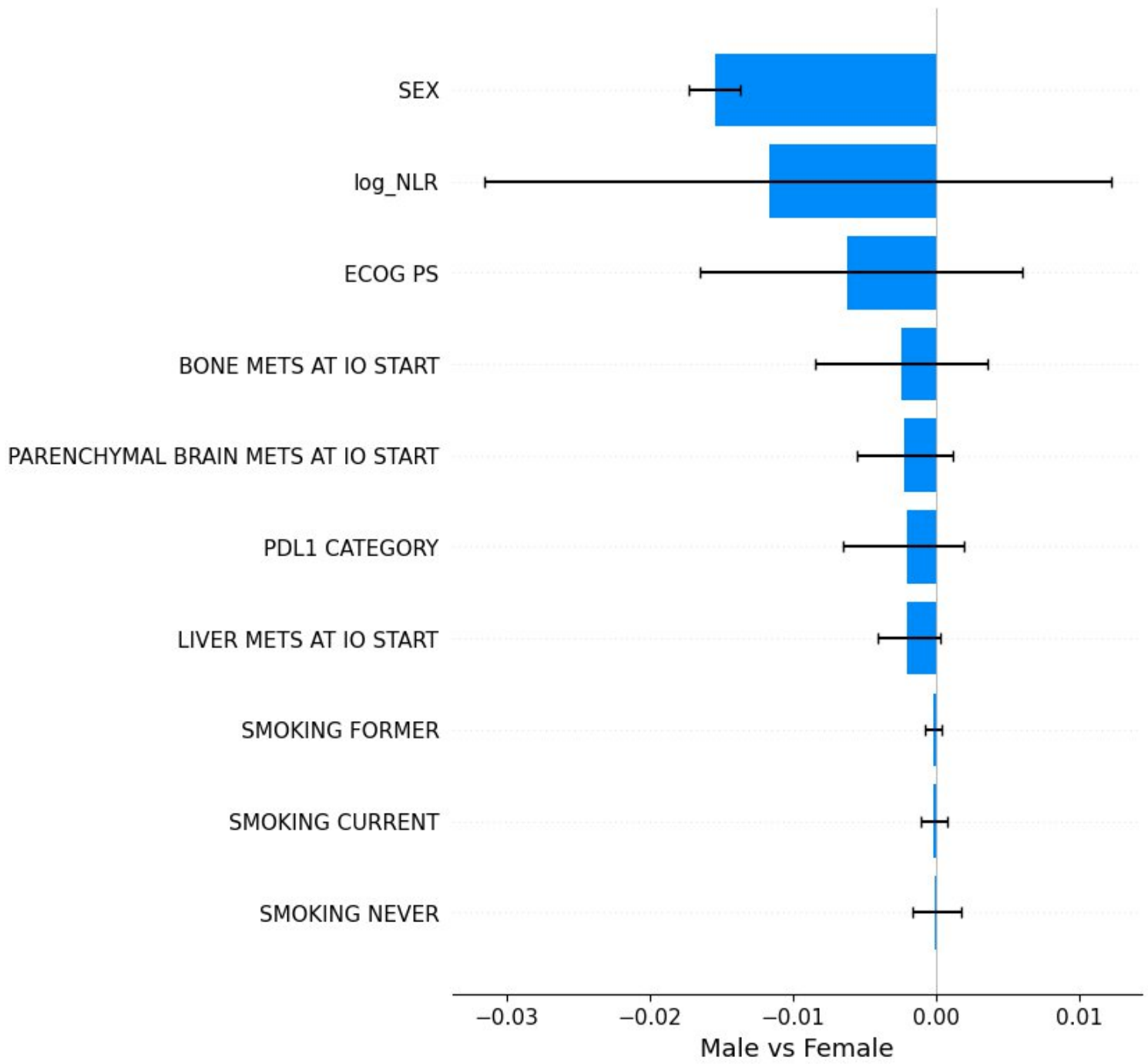

b)

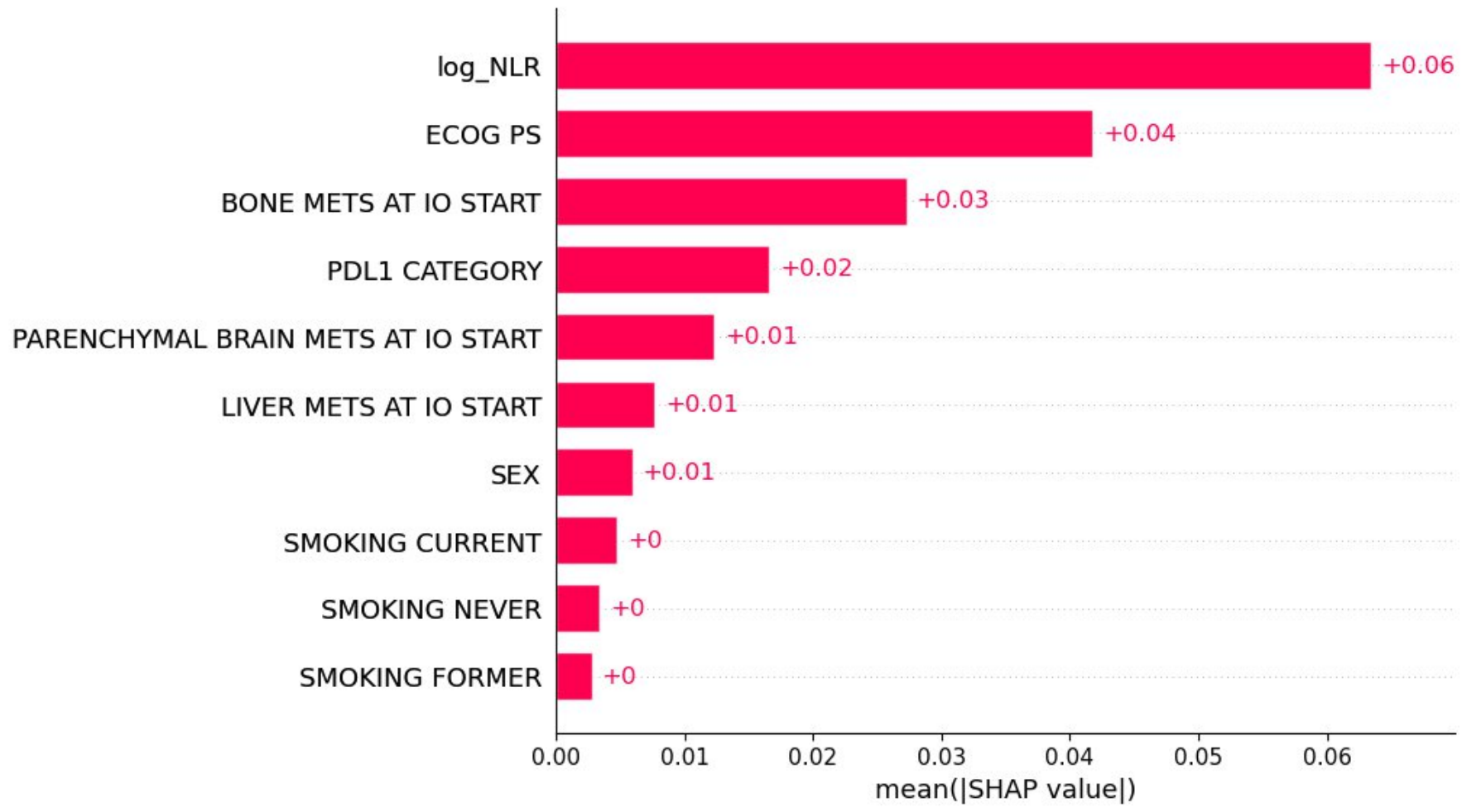

c)

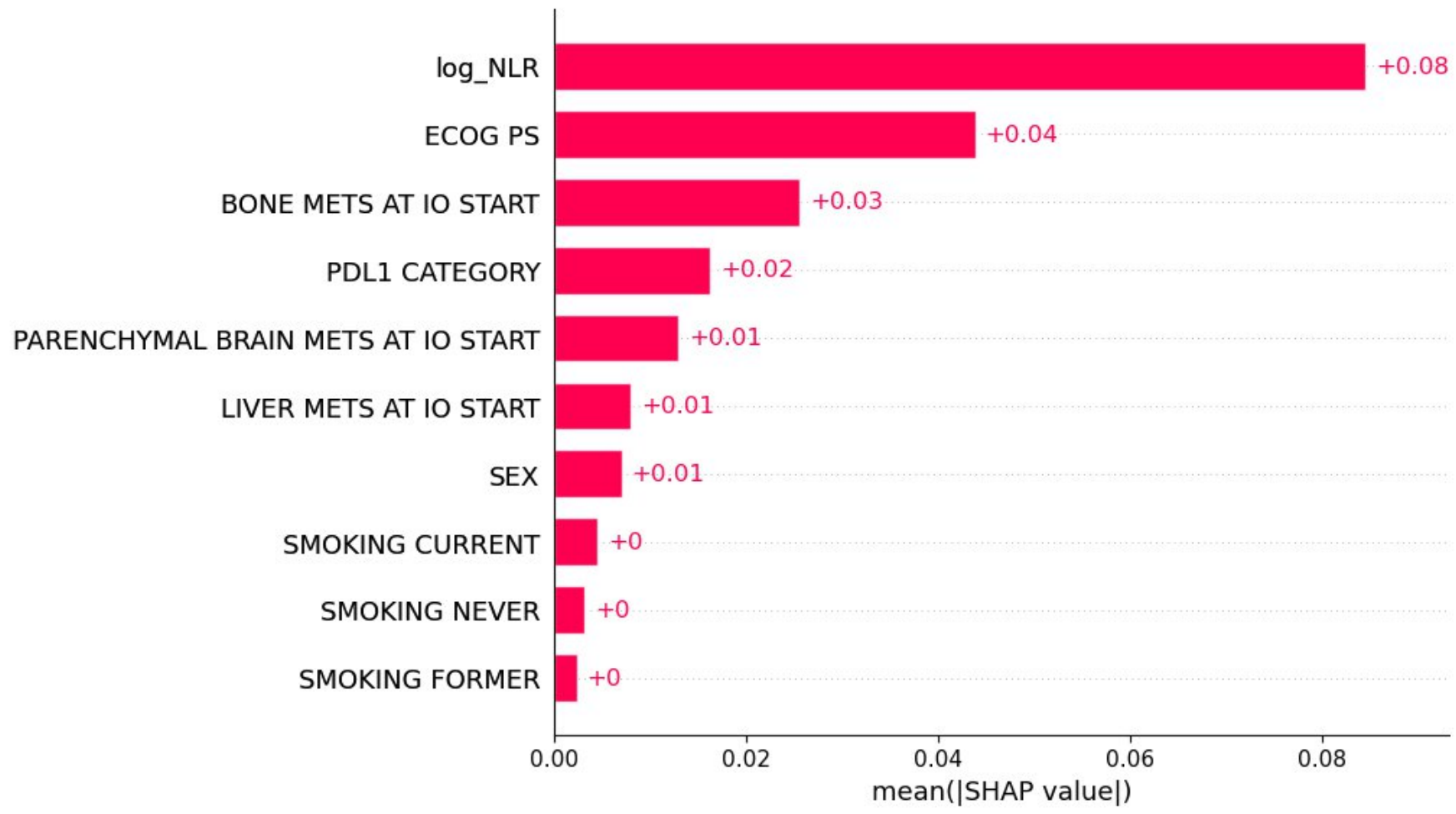

d)

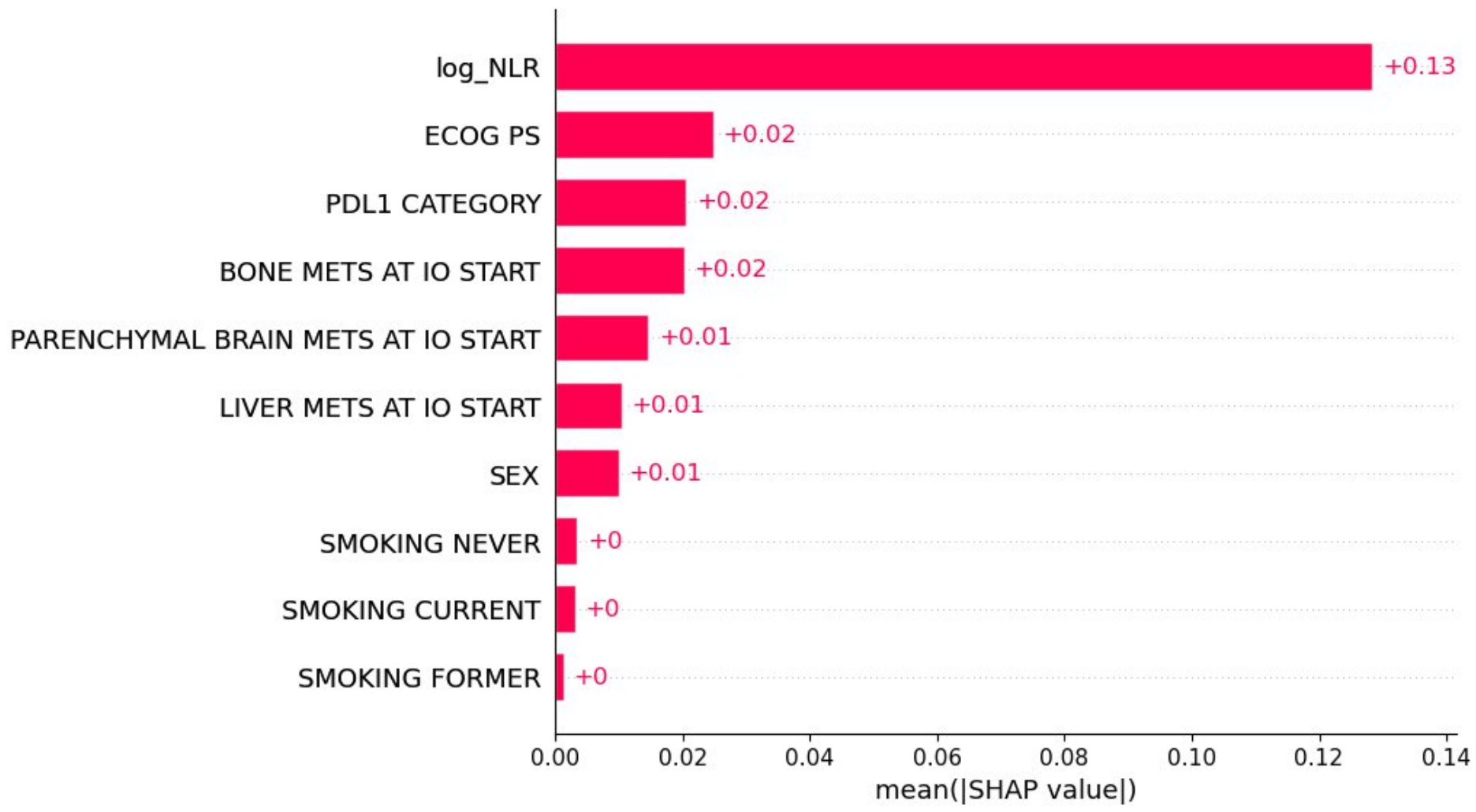

Figure 3. SHAP analysis across sex for MLEF. a) Mean SHAP features differences across sex in OS24 test set patients; b) SHAP bar plots in OS24 train set patients; c) SHAP bar plots in OS24 test set patients; d) SHAP bar plots in OS 24 ExVal set patients.

Table 4. Confusion matrix of CB-only MLEF model OS24 test set, split by sex.

	**TN**	**FP**	**FN**	**TP**
Overall test (n(0)=166,n(1)=60)	83 (36.7%)	83 (36.7%)	9 (4%)	51 (22.6%)
Female (n(0)=60,n(1)=25)	27 (31.8%)	33 (38.8%)	1 (1.2%)	24 (28.8%)
Male (n(0)=106,n(1)=35)	56 (39.7%)	50 (35.5%)	8 (5.7%)	27 (19.1%)

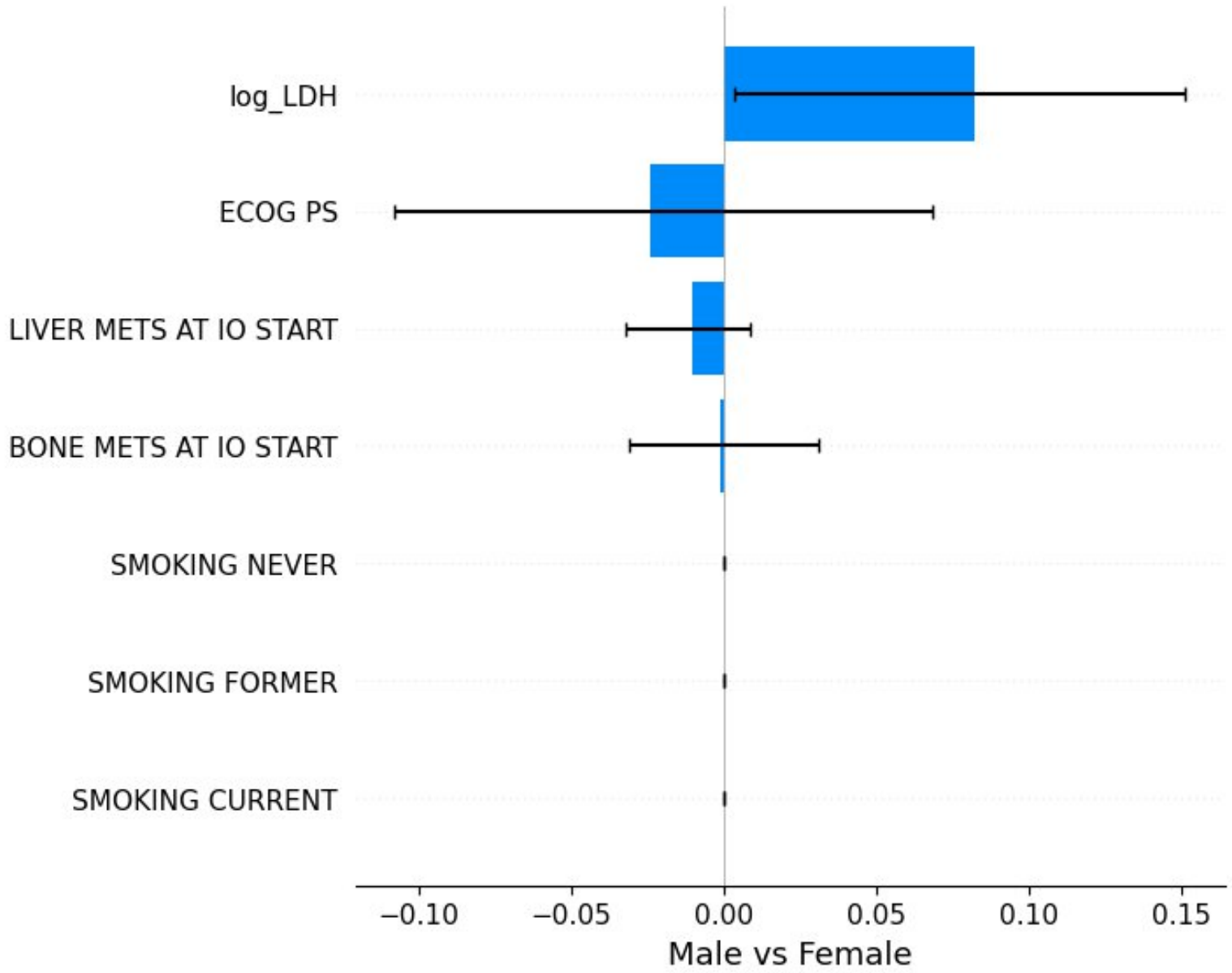

Figure 4. Mean SHAP features differences across sex in DCR test set patients. Regarding the DCR model, the sex feature was not selected by LASSO, and SHAP analysis showed no concerning differences in other features between females and males.

Table 5. Confusion matrix of CB-only MLEF model DCR test set, split by sex.

	TN	FP	FN	TP
Overall test (n(0)=104, n(1)=169)	58 (21.2%)	46 (16.8%)	47 (17.2%)	122 (44.7%)
Female (n(0)=39, n(1)=59)	21 (21.4%)	18 (18.4%)	12 (12.2%)	47 (48.0%)
Male (n(0)=65, n(1)=110)	37 (21.1%)	28 (16.0%	35 (20.0%)	75 (42.9%)

Section 2

a)

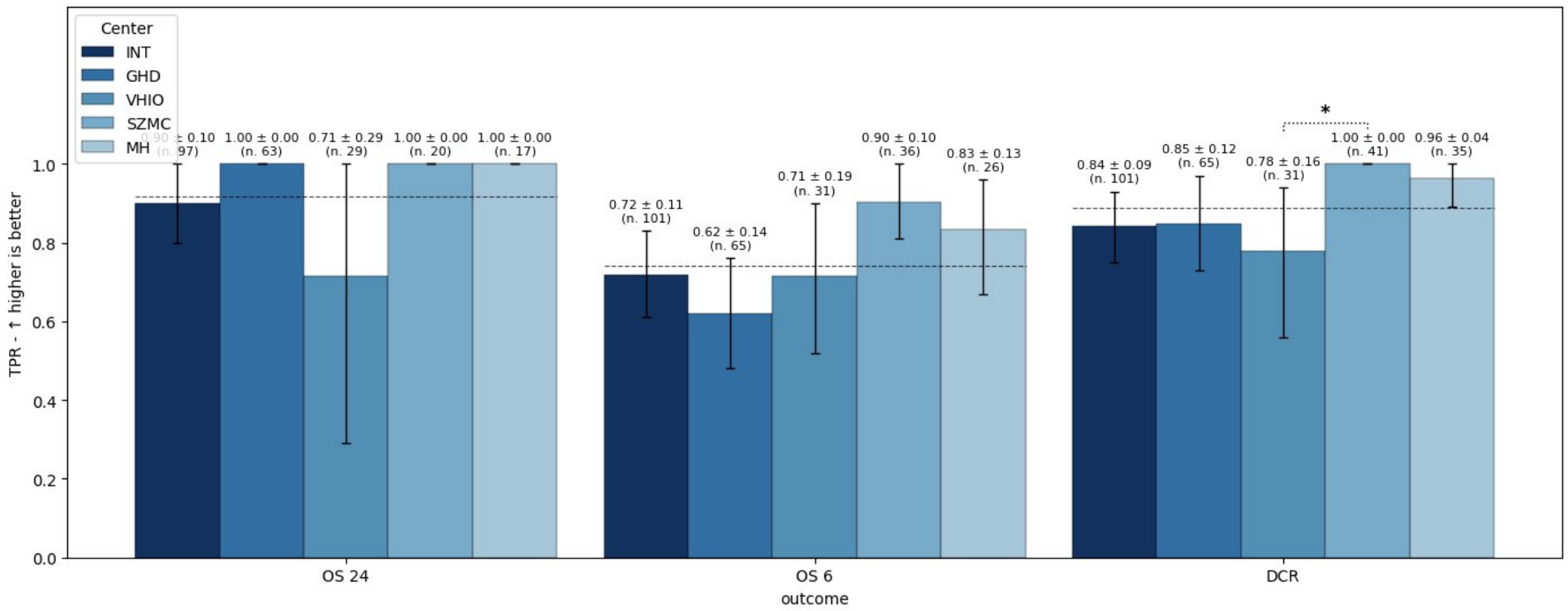

b)

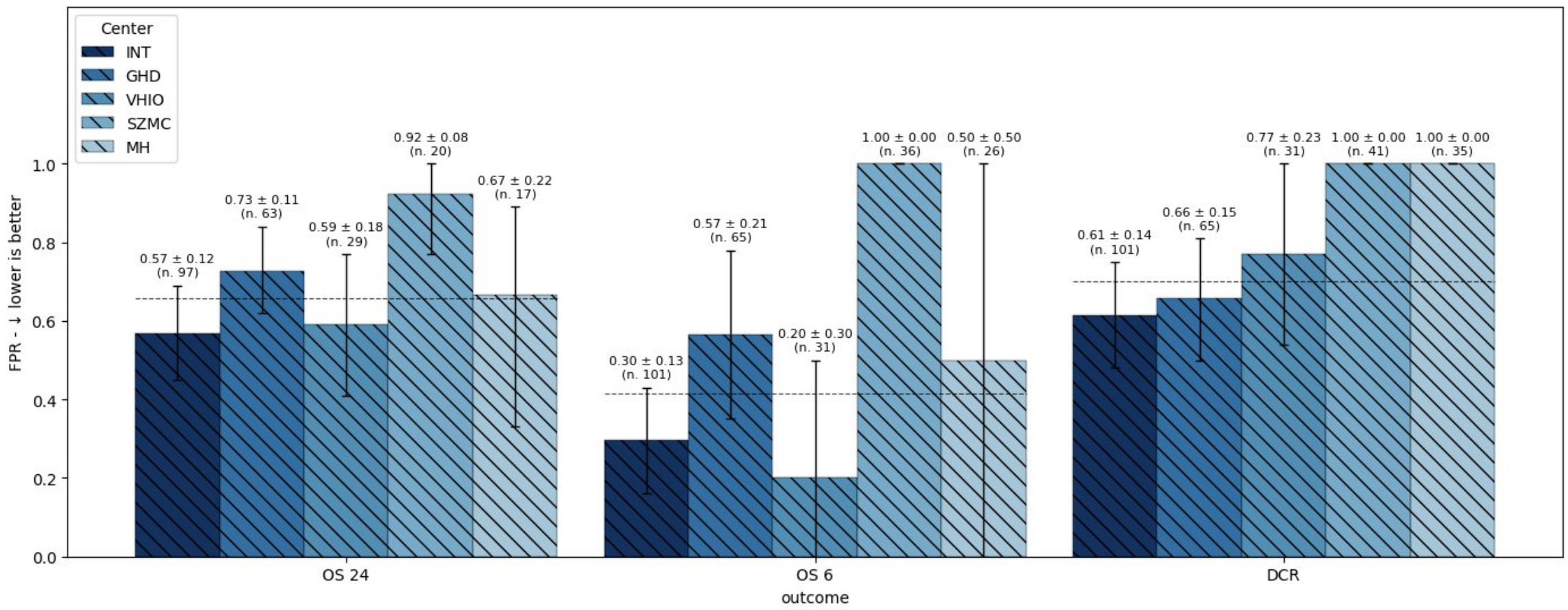

c)

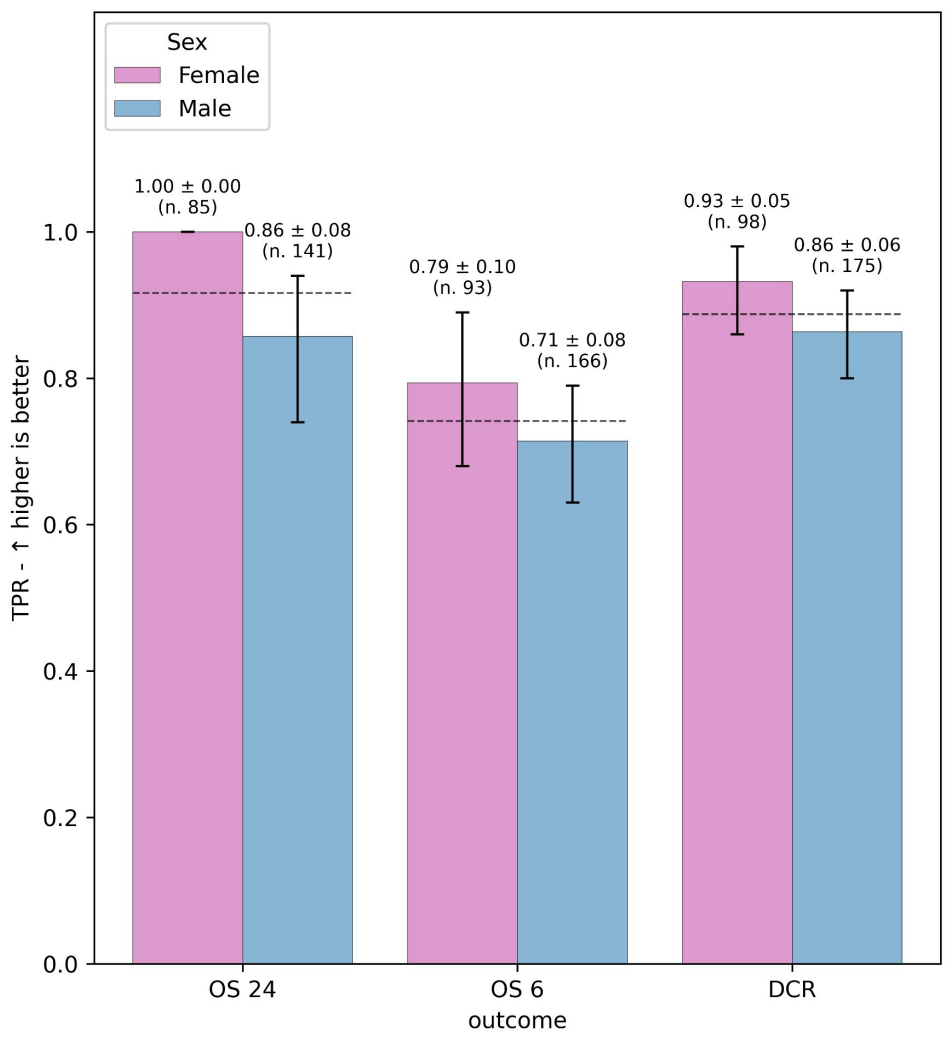

d)

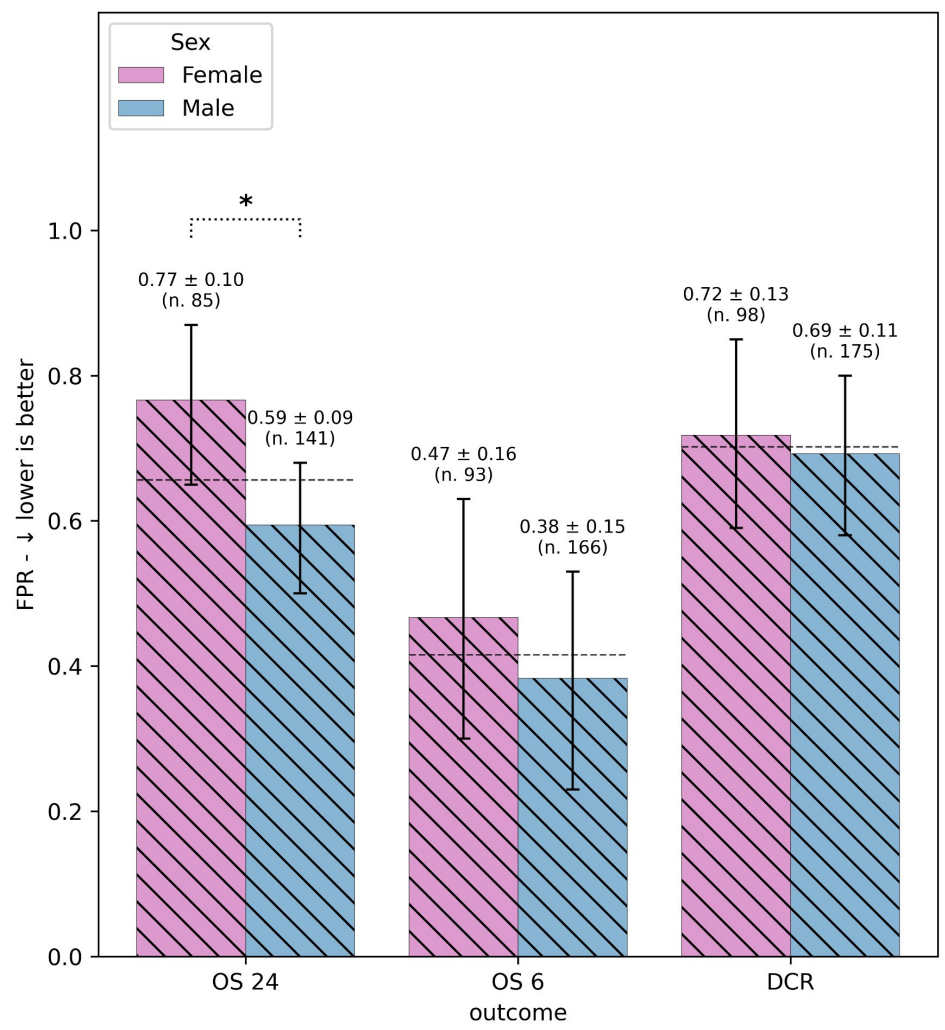

e)

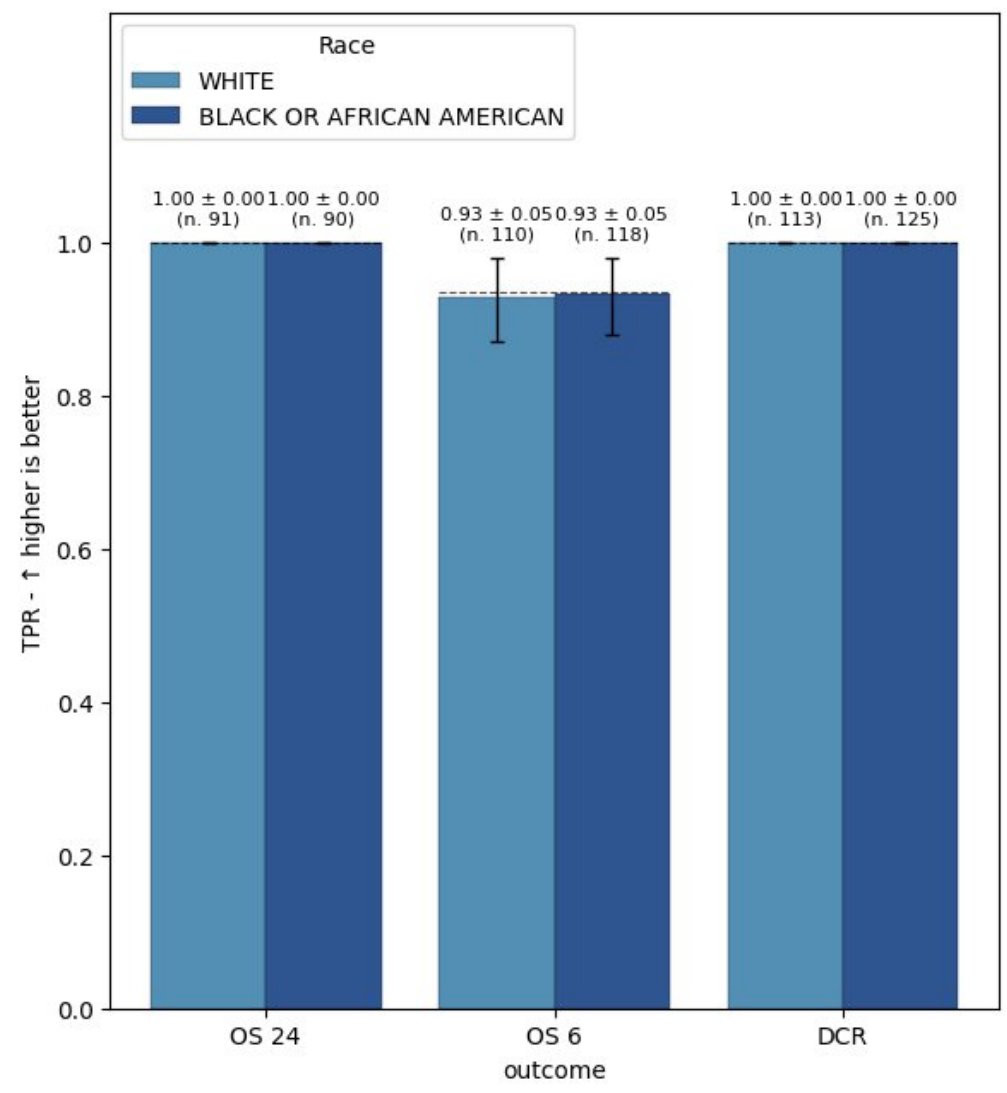

f)

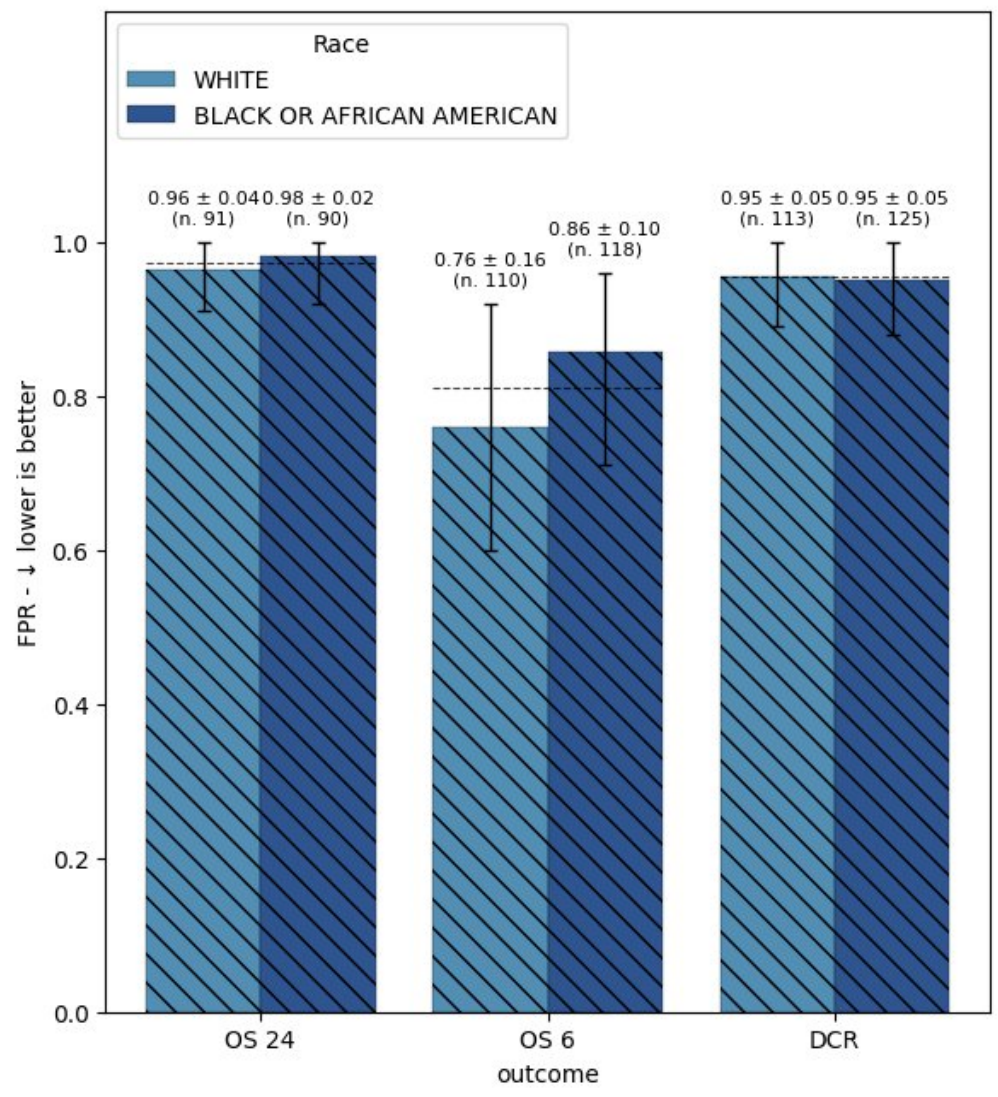

g)

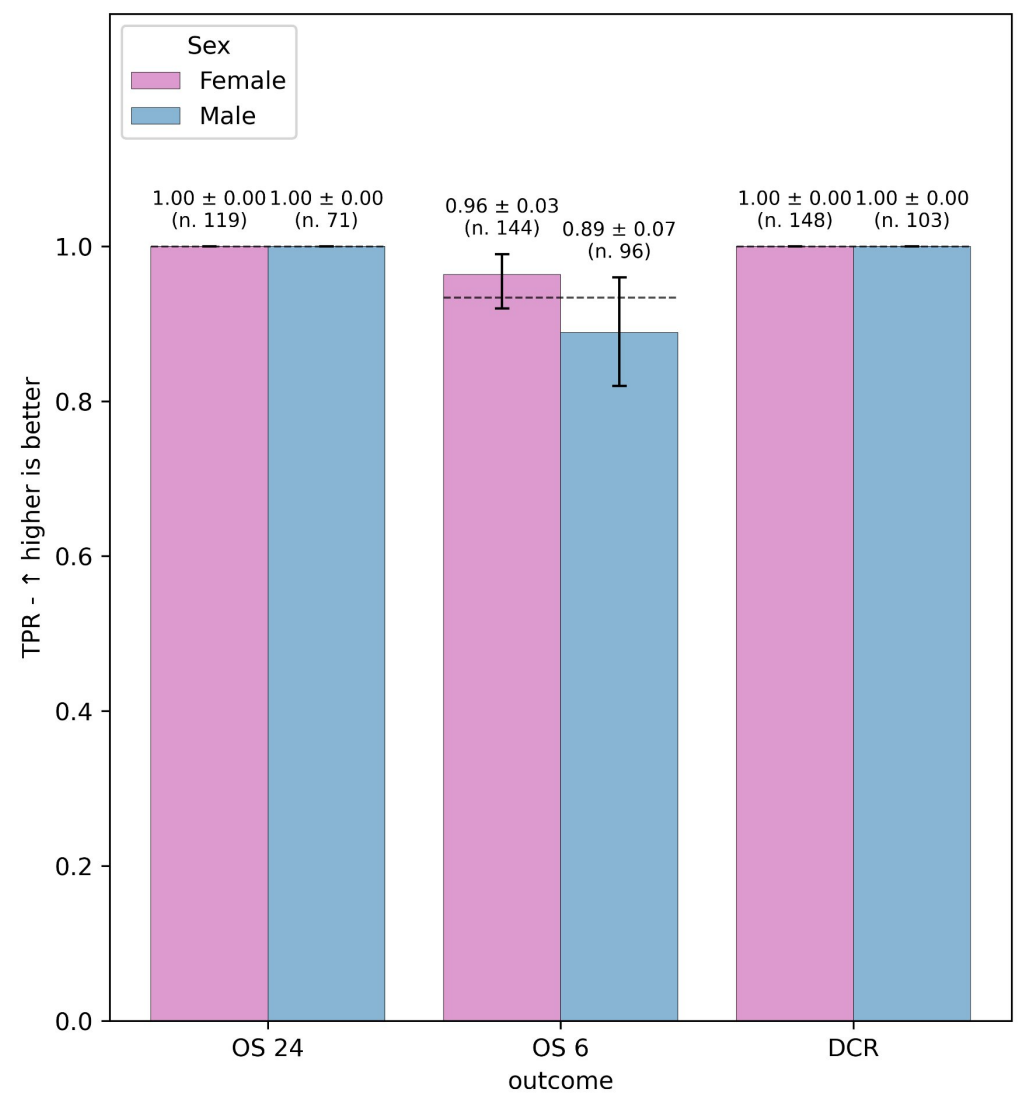

h)

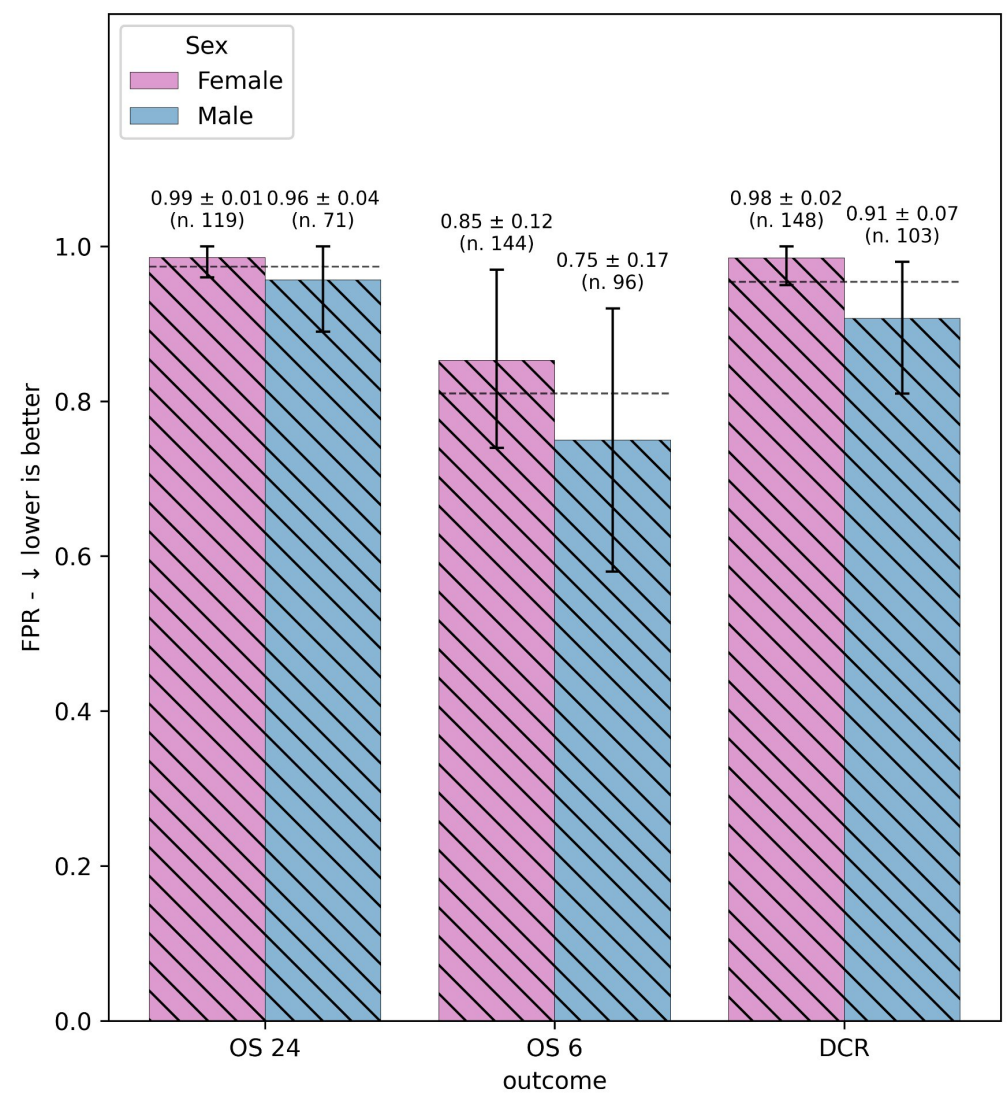

Figure 5. Fairness results of DLIF: a) TPR by center and outcome on test data for DLIF results; b) FPR by center and outcome on test data for DLIF results; c) TPR by sex and outcome on test data for DLIF results; d) FPR by sex and outcome on test data for DLIF results; e) TPR by race and outcome on external validation data for DLIF results; f) FPR by race and outcome on external validation data for DLIF results; g) TPR by sex and outcome on external validation data for DLIF results; h) TPR by sex and outcome on external validation data for DLIF results

There is marginal evidence of significant difference in TPR and FPR across subgroups: we find significant differences between VHIO and SZMC in DCR TPR (p-value ≤ 0.05) and between females and males in OS24 FPR (p-value ≤ 0.05).

Supplementary Information 6

TABLE OF CONTENTS

1. STATISTICAL METHODS 3

Table 1. Confusion matrix template. 4

2. ANALYSIS 1: PHASE 1 vs PHASE 2 5

2.1 DCR 5

Table 2. Contingency table for DCR predictions in phase 1 for all physicians. 5

Table 3. Contingency table for DCR predictions in phase 2 for all physicians. 5

Table 4. Association between DCR prediction success and XAI use for all physicians. Univariable logistic regression model. 5

Table 5. Metrics summary for DCR predictions according to phase for all physicians. 6

Table 6. Contingency table for DCR predictions in phase 1 for expert physicians. 7

Table 7. Contingency table for DCR predictions in phase 2 for expert physicians. 7

Table 8. Association between DCR prediction success and XAI use for expert physicians. Univariable logistic regression model. 7

Table 9. Metrics summary for DCR predictions according to phase for expert physicians. 8

Table 10. Contingency table for DCR predictions in phase 1 for non-expert physicians. 9

Table 11. Contingency table for DCR predictions in phase 2 for non-expert physicians. 9

Table 12. Association between DCR prediction success and XAI use for non-expert physicians. Univariable logistic regression model. 9

Table 13. Metrics summary for DCR predictions according to phase for non-expert physicians. 10

2.2 OS 11

Table 14. Contingency table for OS predictions in phase 1 for all physicians. 11

Table 15. Contingency table for OS predictions in phase 2 for all physicians. 11

Table 16. Association between OS prediction success and XAI use for all physicians. Univariable logistic regression model. 11

Table 17. Total score for OS predictions correctness for all physicians. 11

Table 18. Contingency table for OS predictions in phase 1 for expert physicians. 12

Table 19. Contingency table for OS predictions in phase 2 for expert physicians. 12

Table 20. Association between OS prediction success and XAI use for expert physicians. Univariable logistic regression model. 12

Table 21. Total score for OS predictions correctness for expert physicians. 12

Table 22. Contingency table for OS predictions in phase 1 for non-expert physicians. 13

Table 23. Contingency table for OS predictions in phase 2 for non-expert physicians. 13

Table 24. Association between OS prediction success and XAI use for non-expert physicians. Univariable logistic regression model. 13

Table 25. Total score for OS predictions correctness for non-expert physicians. 13

3. ANALYSIS 2: EXPERTS vs NON-EXPERTS (PHASE 1) 14

3.1 DCR 14
Table 26. Contingency table for DCR predictions by experts and non-experts in phase 1. 14
Table 27. Association between DCR prediction success and physicians'expertise in phase 1. Univariable logistic regression model. 14
Table 28. Metrics summary for DCR predictions according to physicians'expertise in phase 1. 14
3.2 OS 15
Table 29. Contingency table for OS predictions by experts and non-experts in phase 1. 15
Table 30. Association between OS prediction success and physicians'expertise in phase 1. Univariable logistic regression model. 15
Table 31. Total score for OS predictions according to physicians'expertise in phase 1. 15
4. ANALYSIS 3: EXPERTS vs NON-EXPERTS (PHASE 2) 16
4.1 DCR 16
Table 32. Contingency table for DCR predictions by experts and non-experts in phase 2. 16
Table 33. Association between DCR prediction success and physicians'expertise in phase 2. Univariable logistic regression model. 16
Table 34. Metrics summary for DCR predictions according to physicians'expertise in phase 2. 16
4.2 OS 17
Table 35. Contingency table for OS predictions by experts and non-experts in phase 2. 17
Table 36. Association between OS prediction success and physicians'expertise in phase 2. Univariable logistic regression model. 17
Table 37. Total score for OS predictions according to physicians'expertise in phase 2. 17
5. ANALYSIS 4: CONFUSION MATRIX 18
Table 38. Confusion matrix for DCR predictions after using the XAI for all physicians. 18
Table 39. Confusion matrix for DCR predictions after using the XAI for expert physicians. 18
Table 40. Confusion matrix for DCR predictions after using the XAI for non-expert physicians. 19

1. STATISTICAL METHODS

100 patients with a 1 expert to 1 non-lung expert correspondence were included in the analyses.

The association between the DCR prediction success and XAI use for all physicians, for experts only and non-lung experts only was assessed by using a univariable logistic regression model. A binary variable indicating the success of the DCR prediction (1 if the predicted DCR was equal to the true DCR, 0 otherwise) was considered as the response variable; a binary variable corresponding to the phase (1 if phase 1/no XAI use, 2 if phase 2/XAI use) was treated as the covariate. The results were presented as Odds Ratio (OR) and Area Under the Curve (AUC) with their 95% Confidence Interval (95% CI).

The associations between the DCR prediction success and physicians'expertise in phase 1 and phase 2 were assessed by using a univariable logistic regression model. A binary variable indicating the success of the DCR prediction (1 if the predicted DCR was equal to the true DCR, 0 otherwise) was considered as the response variable; a binary variable corresponding to the expertise level (0 if non-expert, 1 if expert) was treated as the covariate. The results were presented as Odds Ratio (OR) and Area Under the Curve (AUC) with their 95% Confidence Interval (95% CI).

The association between the OS prediction success and XAI use for all physicians, for experts only and non-experts only was assessed by using a univariable logistic regression model. A binary variable indicating the success of the OS prediction (1 if the [true OS - 25%; true OS + 25%] interval overlapped with the predicted OS range, 0 otherwise) was considered as the response variable; a binary variable corresponding to the phase (1 if phase 1/no XAI use, 2 if phase 2/XAI use) was treated as the covariate. The results were presented as Odds Ratio (OR) and Area Under the Curve (AUC) with their 95% Confidence Interval (95% CI).

The associations between the OS prediction success and physicians'expertise in phase 1 and phase 2 were assessed by using a univariable logistic regression model. A binary variable indicating the success of the OS prediction (1 if the [true OS - 25%; true OS + 25%] interval overlapped with the predicted OS range, 0 otherwise) was considered as the response variable; a binary variable corresponding to the expertise level (0 if non-expert, 1 if expert) was treated as the covariate. The results were presented as Odds Ratio (OR) and Area Under the Curve (AUC) with their 95% Confidence Interval (95% CI).

The degree of agreement between the expert and non-expert physicians' DCR and OS evaluations were assessed by computing the Cohen's Kappa and the weighted Cohen's Kappa, respectively. Based on the observed Kappa, the degree of concordance were considered as follows:

- 0: no agreement
- [0.01 - 0.20]: slight agreement,
- [0.21 - 0.40]: fair agreement,
- [0.41 - 0.60]: moderate agreement,
- [0.61 - 0.80]: substantial agreement,
- [0.81 - 0.99]: near perfect agreement,
- 1: perfect agreement.

The McNemar's test for paired data was employed to assess any differences between the experts and the non-experts in the discordant predictions in phase 1 and phase 2.

Furthermore, metrics such as the sensitivity, specificity, accuracy and precision were computed for the DCR prediction according to phase and physicians'expertise. The 95% CIs of these metrics were calculated by using the Clopper-Pearson's exact binomial method. The McNemar's test for paired data was employed to assess any statistically significant differences.

The F1-score was derived according to phase and physicians'expertise for the DCR prediction. Its 95% CI was computed by using bootstrap methods with 1000 replications.

Due to the time-to-event nature of the OS endpoint (i.e., it is not possible to define "true positives" and "true negatives" when speaking of survival) it is not possible to compute any of the aforementioned metrics for the OS prediction.

A correctness score for the OS prediction was defined for each patient based on the true OS and the physician's predicted OS ranges as follows:

- A score of 0 points was assigned in case of different and non-consecutive ranges for the true OS and the predicted OS by the physician;

- A score of 1 point was assigned in case of different, but consecutive, ranges for the true OS and the predicted OS by the physician;

- A score of 2 points was assigned in case of identical ranges for the true OS and the predicted OS by the physician.

The Wilcoxon signed-rank test for paired data was employed to assess any statistically significant differences between the assigned scores'distributions. A total correctness score was computed by summing all scores according to the phase and physicians'expertise.

Finally, the confusion matrix after using the XAI was derived according to Rosenbacke et al. for all physicians, for experts only and non-experts only.

The following table shows the confusion matrix template:

Table 1. Confusion matrix template.

	Physician correct		Physician wrong	
XAI correct	True confirmation error	True confirmation (correct)	True conflict (correct)	True conflict error
XAI wrong	False conflict error	False conflict (correct)	False confirmation (correct)	False confirmation error
	Physician changes after XAI advice	Physician stays after XAI advice	Physician changes after XAI advice	Physician stays after XAI advice

2. ANALYSIS 1: PHASE 1 vs PHASE 2

2.1 DCR

Table 2. Contingency table for DCR predictions in phase 1 for all physicians.

Predicted DCR	DCR (Ground truth)	
	PD	DCR
PD	27 (13.5%)	33 (16.5%)
DCR	53 (26.5%)	87 (43.5%)

In the 57% of cases the predicted DCR coincides with the ground truth (13.5%+43.5%) before using the XAI (all physicians).

Table 3. Contingency table for DCR predictions in phase 2 for all physicians.

Predicted DCR	DCR (Ground truth)	
	PD	DCR
PD	25 (12.5%)	16 (8.0%)
DCR	55 (27.5%)	104 (52.0%)

In the 62.5% of cases the predicted DCR coincides with the ground truth (12.5%+52.0%) after using the XAI (all physicians).

Table 4. Association between DCR prediction success and XAI use for all physicians. Univariable logistic regression model.

N = 400		
Phase		p = 0.1250
1	reference	
2	OR (95% CI) = 1.37 (0.92-2.05)	p = 0.1250
AUC (95% CI)	0.539 (0.489-0.590)	

Legend: N: number of predictions. OR: Odds Ratio. AUC: Area Under the Curve. CI: Confidence Interval.

NOTE: ORR = 1.37 means that, overall, using the XAI increases the probability of success (i.e., predicting the true DCR) by 37%. However, this association is not statistically significant.

Table 5. Metrics summary for DCR predictions according to phase for all physicians.

	Phase 1	Phase 2	p-value
Sensitivity (95% CI)	0.725 (0.636-0.803)	0.867 (0.793-0.922)	0.0011
Specificity (95% CI)	0.338 (0.236-0.452)	0.313 (0.214-0.426)	0.7055
Accuracy (95% CI)	0.570 (0.498-0.640)	0.645 (0.574-0.711)	0.0431
Precision (95% CI)	0.621 (0.536-0.702)	0.654 (0.575-0.728)	0.2498
F1-Score (95% CI)	0.669 (0.598-0.733)	0.746 (0.687-0.803)	-

We can reasonably infer that the sensitivity improved after the XAI use based on the non-inclusion in each other's 95% CI. This means that using the XAI changes the physician's opinion on false negatives and it increases the number of DCR patients to be treated. Based on the same interpretation, using the XAI also improves the accuracy (the overall percentage of correct evaluations) and the F1-score. The McNemar's test confirms the conclusions made on the sensitivity and accuracy, since the differences are statistically significant at the 5% nominal level.

Table 6. Contingency table for DCR predictions in phase 1 for expert physicians.

Predicted DCR	DCR (Ground truth)	
	PD	DCR
PD	17 (17.0%)	19 (19.0%)
DCR	23 (23.0%)	41 (41.0%)

In the 58% of cases the predicted DCR coincides with the ground truth (17.0%+41.0%) before using XAI (experts only).

Table 7. Contingency table for DCR predictions in phase 2 for expert physicians.

Predicted DCR	DCR (Ground truth)	
	PD	DCR
PD	13 (13.0%)	10 (10.0%)
DCR	27 (27.0%)	50 (50.0%)

In the 63% of cases the predicted DCR coincides with the ground truth (13.0%+50.0%) after using XAI (experts only).

Table 8. Association between DCR prediction success and XAI use for expert physicians. Univariable logistic regression model.

N = 200		
Phase		p = 0.4698
1	reference	
2	OR (95% CI) = 1.23 (0.70-2.18)	p = 0.4698
AUC (95% CI)	0.526 (0.455-0.597)	

Legend: N: number of predictions. OR: Odds Ratio. AUC: Area Under the Curve. CI: Confidence Interval.

NOTE: ORR = 1.23 means that, for expert physicians, using the XAI increases the probability of success (i.e., predicting the true DCR) by 23%. However, this association is not statistically significant.

Table 9. Metrics summary for DCR predictions according to phase for expert physicians.

	Phase 1	Phase 2	p-value
Sensitivity (95% CI)	0.683 (0.550-0.797)	0.833 (0.715-0.917)	0.0201
Specificity (95% CI)	0.425 (0.270-0.591)	0.325 (0.186-0.491)	0.3173
Accuracy (95% CI)	0.580 (0.477-0.678)	0.630 (0.528-0.724)	0.3692
Precision (95% CI)	0.641 (0.511-0.757)	0.649 (0.532-0.755)	0.6698
F1-Score (95% CI)	0.661 (0.560-0.752)	0.730 (0.641-0.808)	-

We can reasonably infer that the sensitivity improved after the XAI use based on the non-inclusion in each other's 95% CI. This means that using the XAI changes the expert's opinion on false negatives and it increases the number of DCR patients to be treated. The McNemar's test confirms the conclusions made on the sensitivity, since the difference is statistically significant at the 5% nominal level.

Table 10. Contingency table for DCR predictions in phase 1 for non-expert physicians.

Predicted DCR	DCR (Ground truth)	
	PD	DCR
PD	10 (10.0%)	14 (14.0%)
DCR	30 (30.0%)	46 (46.0%)

In the 56% of cases the predicted DCR coincides with the ground truth before using the XAI (non-experts only).

Table 11. Contingency table for DCR predictions in phase 2 for non-expert physicians.

Predicted DCR	DCR (Ground truth)	
	PD	DCR
PD	12 (12.0%)	6 (6.0%)
DCR	28 (28.0%)	54 (54.0%)

In the 66% of cases the predicted DCR coincides with the ground truth after using the XAI (non-experts only).

Table 12. Association between DCR prediction success and XAI use for non-expert physicians. Univariable logistic regression model.

N = 200		
Phase		p = 0.1480
1	reference	
2	OR (95% CI) = 1.53 (0.86-2.60)	p = 0.1480
AUC (95% CI)	0.553 (0.482-0.624)	

Legend: N: number of predictions. OR: Odds Ratio. AUC: Area Under the Curve. CI: Confidence Interval.

NOTE: OR = 1.53 means that, for non-expert physicians, using the XAI increases the probability of success (i.e., predicting the true DCR) by 53%. However, this association is not statistically significant.

Table 13. Metrics summary for DCR predictions according to phase for non-expert physicians.

	Phase 1	Phase 2	p-value
Sensitivity (95% CI)	0.767 (0.640-0.866)	0.900 (0.795-0.962)	0.0209
Specificity (95% CI)	0.250 (0.127-0.412)	0.300 (0.166-0.465)	0.5637
Accuracy (95% CI)	0.560 (0.457-0.659)	0.660 (0.559-0.752)	0.0412
Precision (95% CI)	0.605 (0.487-0.716)	0.659 (0.546-0.760)	0.1967
F1-Score (95% CI)	0.676 (0.583-0.758)	0.761 (0.678-0.830)	-

We can reasonably infer that the sensitivity improved after the XAI use based on the non-inclusion in each other's 95% CI. This means that using the XAI changes the non-expert's opinion on false negatives and it increases the number of DCR patients to be treated. Based on the same interpretation, using the XAI also improves the accuracy (the overall percentage of correct evaluations) and the F1-score. The McNemar's test confirms the conclusions made on the sensitivity and the accuracy, since the differences are statistically significant.

2.2 OS

Table 14. Contingency table for OS predictions in phase 1 for all physicians.

Predicted OS	OS (Ground truth)				
	< 6 months	[6-12) months	[12-18) months	[18-24) months	≥24 months
< 6 months	4 (2.0%)	5 (2.5%)	2 (1.0%)	0 (0.0%)	4 (2.0%)
[6-12) months	20 (10.0%)	12 (6.0%)	8 (4.0%)	9 (4.5%)	18 (9.0%)
[12-18) months	13 (6.5%)	10 (5.0%)	7 (3.5%)	6 (3.0%)	21 (10.5%)
[18-24) months	9 (4.5%)	10 (5.0%)	3 (1.5%)	1 (0.5%)	14 (7.0%)
≥24 months	10 (5.0%)	1 (0.5%)	2 (1.0%)	2 (1.0%)	9 (4.5%)

Table 15. Contingency table for OS predictions in phase 2 for all physicians.

Predicted OS	OS (Ground truth)				
	< 6 months	[6-12) months	[12-18) months	[18-24) months	≥24 months
< 6 months	8 (4.0%)	1 (0.5%)	1 (0.5%)	3 (1.5%)	1 (0.5%)
[6-12) months	16 (8.0%)	15 (7.5%)	8 (4.0%)	3 (1.5%)	13 (6.5%)
[12-18) months	17 (8.5%)	11 (5.5%)	4 (2.0%)	9 (4.5%)	24 (12.0%)
[18-24) months	6 (3.0%)	8 (4.0%)	4 (2.0%)	2 (1.0%)	12 (6.0%)
≥24 months	9 (4.5%)	3 (1.5%)	5 (2.5%)	1 (0.5%)	16 (8.0%)

Table 16. Association between OS prediction success and XAI use for all physicians. Univariable logistic regression model.

N = 400		
Phase		p = 0.1440
1	reference	
2	OR (95% CI) = 1.36 (0.90-2.05)	p = 0.1440
AUC (95% CI)	0.538 (0.487-0.589)	

Legend: N: number of predictions. OR: Odds Ratio. AUC: Area Under the Curve. CI: Confidence Interval.

NOTE: ORR = 1.36 means that, overall, using the XAI increases the probability of success (i.e., predicting the true OS) by 36%. However, this association is not statistically significant.

Table 17. Total score for OS predictions correctness for all physicians.

Phase 1	Phase 2	Wilcoxon signed-rank test, p-value
134/400 (33.5%)	152/400 (38.0%)	0.1149

Using the XAI appears to improve the correctness of the OS prediction. However, from the Wilcoxon signed-rank test, no statistical differences between the scores' distributions were found at the 5% level, hence no definitive conclusion can be made about this improvement of the total score between phase 1 and phase 2.

Table 18. Contingency table for OS predictions in phase 1 for expert physicians.

Predicted OS	OS (Ground truth)

	< 6 months	[6-12) months	[12-18) months	[18-24) months	≥24 months
< 6 months	1 (1.0%)	2 (2.0%)	1 (1.0%)	0 (0.0%)	1 (0.0%)
[6-12) months	13 (13.0%)	6 (6.0%)	5 (5.0%)	5 (5.0%)	7 (7.0%)
[12-18) months	5 (5.0%)	6 (6.0%)	3 (3.0%)	2 (2.0%)	11 (11.0%)
[18-24) months	4 (4.0%)	5 (5.0%)	2 (2.0%)	0 (0.0%)	8 (8.0%)
≥24 months	5 (5.0%)	5 (0.0%)	2 (2.0%)	2 (2.0%)	6 (6.0%)

Table 19. Contingency table for OS predictions in phase 2 for expert physicians.

Predicted OS	OS (Ground truth)				
	< 6 months	[6-12) months	[12-18) months	[18-24) months	≥24 months
< 6 months	4 (4.0%)	1 (1.0%)	0 (0.0%)	1 (1.0%)	1 (1.0%)
[6-12) months	8 (8.0%)	7 (7.0%)	4 (4.0%)	1 (1.0%)	7 (7.0%)
[12-18) months	9 (9.0%)	5 (5.0%)	2 (2.0%)	5 (5.0%)	11 (11.0%)
[18-24) months	5 (5.0%)	6 (6.0%)	3 (3.0%)	1 (1.0%)	6 (6.0%)
≥24 months	2 (2.0%)	0 (0.0%)	1 (1.0%)	1 (1.0%)	8 (8.0%)

Table 20. Association between OS prediction success and XAI use for expert physicians. Univariable logistic regression model.

N = 200		
Phase		p = 0.6555
1	reference	
2	OR (95% CI) = 1.14 (0.64-2.05)	p = 0.6555
AUC (95% CI)	0.517 (0.443-0.590)	

Legend: N: number of predictions. OR: Odds Ratio. AUC: Area Under the Curve. CI: Confidence Interval.

NOTE: ORR = 1.14 means that, overall, using the XAI increases the probability of success (i.e., predicting the true OS) by 14%. However, this association is not statistically significant.

Table 21. Total score for OS predictions correctness for expert physicians.

Phase 1	Phase 2	Wilcoxon signed-rank test, p-value
72/200 (36.0%)	77/200 (38.5%)	0.5491

Using the XAI appears to improve the correctness of the OS prediction. However, from the Wilcoxon signed-rank test, no statistical differences between the scores' distributions were found at the 5% level, hence no definitive conclusion can be made about this improvement of the total score between phase 1 and phase 2.

Table 22. Contingency table for OS predictions in phase 1 for non-expert physicians.

Predicted OS	OS (Ground truth)				
	< 6 months	[6-12) months	[12-18) months	[18-24) months	≥24 months
< 6 months	3 (3.0%)	3 (3.0%)	1 (1.0%)	0 (0.0%)	3 (3.0%)
[6-12) months	7 (7.0%)	6 (6.0%)	3 (3.0%)	4 (4.0%)	11 (11.0%)
[12-18) months	8 (8.0%)	4 (4.0%)	4 (3.0%)	4 (4.0%)	10 (10.0%)
[18-24) months	5 (5.0%)	5 (5.0%)	1 (1.0%)	1 (1.0%)	6 (6.0%)
≥24 months	5 (5.0%)	1 (1.0%)	2 (2.0%)	0 (0.0%)	3 (3.0%)

Table 23. Contingency table for OS predictions in phase 2 for non-expert physicians.

Predicted OS	OS (Ground truth)				
	< 6 months	[6-12) months	[12-18) months	[18-24) months	≥24 months
< 6 months	4 (4.0%)	0 (0.0%)	1 (1.0%)	2 (2.0%)	0 (0.0%)
[6-12) months	8 (8.0%)	8 (8.0%)	4 (4.0%)	2 (2.0%)	6 (6.0%)
[12-18) months	8 (8.0%)	6 (6.0%)	2 (2.0%)	4 (4.0%)	13 (13.0%)
[18-24) months	1 (1.0%)	2 (2.0%)	1 (1.0%)	1 (1.0%)	6 (6.0%)
≥24 months	7 (7.0%)	3 (3.0%)	3 (3.0%)	0 (0.0%)	8 (8.0%)

Table 24. Association between OS prediction success and XAI use for non-expert physicians. Univariable logistic regression model.

N = 200		
Phase		p = 0.1072
1	reference	
2	OR (95% CI) = 1.61 (0.90-2.88)	p = 0.1072
AUC (95% CI)	0.559 (0.488-0.631)	

Legend: N: number of predictions. OR: Odds Ratio. AUC: Area Under the Curve. CI: Confidence Interval.

NOTE: ORR = 1.61 means that, overall, using the XAI increases the probability of success (i.e., predicting the true OS) by 61%. However, this association is not statistically significant.

Table 25. Total score for OS predictions correctness for non-expert physicians.

Phase 1	Phase 2	Wilcoxon signed-rank test, p-value
62/200 (31.0%)	75/200 (37.5%)	0.0759

Using the XAI appears to improve the correctness of the OS prediction. However, from the Wilcoxon signed-rank test, no statistical differences between the scores'distributions were found at the 5% level, hence no definitive conclusion can be made about this improvement of the total score between phase 1 and phase 2.

3. ANALYSIS 2: EXPERTS vs NON-EXPERTS (PHASE 1)

3.1 DCR

Table 26. Contingency table for DCR predictions by experts and non-experts in phase 1.

Predicted DCR by experts	Predicted DCR by non-experts	
	PD	DCR
PD	11 (11.0%)	25 (25.0%)
DCR	13 (13.0%)	51 (51.0%)
Cohen's Kappa	0.1105 (slight agreement)	
McNemar's test p-value	0.0516 (no significant difference between discordant predictions)	

Table 27. Association between DCR prediction success and physicians'expertise in phase 1. Univariable logistic regression model.

N = 200		
Level of expertise		p = 0.7752
Non-expert	reference	
Expert	OR (95% CI) = 1.09 (0.62-1.90)	p = 0.7752
AUC (95% CI)	0.510 (0.440-0.581)	

Legend: N: number of predictions. OR: Odds Ratio. AUC: Area Under the Curve. CI: Confidence Interval.

NOTE: ORR = 1.09 means that being an expert increases the probability of success (i.e., predicting the true DCR) by 9% in phase 1. However, this association is not statistically significant.

Table 28. Metrics summary for DCR predictions according to physicians'expertise in phase 1.

	Non-expert	Expert	p-value
Sensitivity (95% CI)	0.767 (0.640-0.866)	0.683 (0.550-0.797)	0.2752
Specificity (95% CI)	0.250 (0.127-0.412)	0.425 (0.270-0.591)	0.0896
Accuracy (95% CI)	0.560 (0.457-0.659)	0.580 (0.477-0.678)	0.7456
Precision (95% CI)	0.605 (0.487-0.716)	0.641 (0.511-0.757)	0.4054
F1-Score (95% CI)	0.676 (0.583-0.758)	0.661 (0.560-0.752)	-

Before using the XAI, the experts appear to present a better specificity based on the non-inclusion in each other's 95% CI. This means that an expert was better in identifying PD patients who should not be treated. However, from the McNemar test, this difference is not statistically significant.

3.2 OS

Table 29. Contingency table for OS predictions by experts and non-experts in phase 1.

Predicted OS by experts	Predicted OS by non-experts				
	< 6 months	[6-12) months	[12-18) months	[18-24) months	≥24 months
< 6 months	1 (1.0%)	2 (2.0%)	2 (2.0%)	0 (0.0%)	0 (0.0%)
[6-12) months	4 (4.0%)	10 (10.0%)	13 (13.0%)	7 (7.0%)	2 (2.0%)
[12-18) months	2 (2.0%)	13 (13.0%)	6 (6.0%)	5 (5.0%)	1 (1.0%)
[18-24) months	1 (1.0%)	4 (4.0%)	6 (6.0%)	5 (5.0%)	3 (3.0%)
≥24 months	2 (2.0%)	2 (2.0%)	3 (3.0%)	1 (1.0%)	5 (5.0%)
Cohen's Weighted Kappa	0.1426 (slight agreement)				

Table 30. Association between OS prediction success and physicians'expertise in phase 1. Univariable logistic regression model.

N = 200		
Level of expertise		p = 0.7618
Non-expert	reference	
Expert	OR (95% CI) = 1.10 (0.61-1.99)	p = 0.7618
AUC (95% CI)	0.512 (0.437-0.586)	

Legend: N: number of predictions. OR: Odds Ratio. AUC: Area Under the Curve. CI: Confidence Interval.

NOTE: ORR = 1.10 means that being an expert increases the probability of success (i.e., predicting the true OS) by 10% in phase 1. However, this association is not statistically significant.

Table 31. Total score for OS predictions according to physicians'expertise in phase 1.

Non-expert	Expert	Wilcoxon signed-rank test, p-value
62/200 (31.0%)	72/200 (36.5%)	0.2868

During phase 1 the experts appear to have a better OS prediction correctness. However, from the Wilcoxon signed-rank test, no statistical differences between the scores'distributions were found at the 5% level, hence no definitive conclusion can be made about this difference of the total score between experts and non-experts in phase 1.

4. ANALYSIS 3: EXPERTS vs NON-EXPERTS (PHASE 2)

4.1 DCR

Table 32. Contingency table for DCR predictions by experts and non-experts in phase 2.

Predicted DCR by experts	Predicted DCR by non-experts	
	PD	DCR
PD	12 (12.0%)	11 (11.0%)
DCR	6 (6.0%)	71 (71.0%)
Cohen's Kappa	0.4804 (moderate agreement)	
McNemar's test p-value	0.2253 (no significant difference between discordant predictions)	

Table 33. Association between DCR prediction success and physicians' expertise in phase 2. Univariable logistic regression model.

N = 200		
Level of expertise		p = 0.6576
Non-expert	reference	
Expert	OR (95% CI) = 0.88 (0.49-1.57)	p = 0.6576
AUC (95% CI)	0.516 (0.444-0.589)	

Legend: N: number of predictions. OR: Odds Ratio. AUC: Area Under the Curve. CI: Confidence Interval.

NOTE: ORR = 0.88 means that being an expert reduces the probability of success (i.e., predicting the true DCR) by 12% in phase 2. However, this association is not statistically significant.

Table 34. Metrics summary for DCR predictions according to physicians' expertise in phase 2.

	Non-expert	Expert	p-value
Sensitivity (95% CI)	0.900 (0.795-0.962)	0.833 (0.715-0.917)	0.1573
Specificity (95% CI)	0.300 (0.166-0.465)	0.325 (0.186-0.491)	0.7389
Accuracy (95% CI)	0.660 (0.559-0.752)	0.630 (0.528-0.724)	0.4669
Precision (95% CI)	0.659 (0.546-0.760)	0.649 (0.532-0.755)	0.4142
F1-Score (95% CI)	0.761 (0.678-0.830)	0.730 (0.641-0.808)	-

After using the XAI, there are no significant differences between experts and non-experts in terms of performance.

4.2 OS

Table 35. Contingency table for OS predictions by experts and non-experts in phase 2.

Predicted OS by experts	Predicted OS by non-experts				
	< 6 months	[6-12) months	[12-18) months	[18-24) months	≥24 months
< 6 months	2 (2.0%)	2 (2.0%)	0 (0.0%)	0 (0.0%)	3 (3.0%)
[6-12) months	4 (4.0%)	12 (12.0%)	9 (9.0%)	1 (1.0%)	1 (1.0%)
[12-18) months	1 (1.0%)	11 (11.0%)	12 (12.0%)	3 (3.0%)	5 (5.0%)
[18-24) months	0 (0.0%)	2 (2.0%)	9 (9.0%)	5 (5.0%)	5 (5.0%)
≥24 months	0 (0.0%)	1 (1.0%)	3 (3.0%)	2 (2.0%)	7 (7.0%)
Cohen's Weighted Kappa	0.3359 (fair agreement)				

Table 36. Association between OS prediction success and physicians'expertise in phase 2. Univariable logistic regression model.

N = 200		
Level of expertise		p = 0.3848
Non-expert	reference	
Expert	OR (95% CI) = 0.78 (0.44-1.37)	p = 0.3848
AUC (95% CI)	0.532 (0.460-0.603)	

Legend: N: number of predictions. OR: Odds Ratio. AUC: Area Under the Curve. CI: Confidence Interval.

NOTE: ORR = 0.78 means that being an expert reduces the probability of success (i.e., predicting the true OS) by 22% in phase 2. However, this association is not statistically significant.

Table 37. Total score for OS predictions according to physicians'expertise in phase 2.

Non-expert	Expert	Wilcoxon signed-rank test, p-value
75/200 (37.5%)	77/200 (38.5%)	0.8187

During phase 2 the experts appear to have a slightly better OS prediction correctness. However, from the Wilcoxon signed-rank test, no statistical differences between the scores'distributions were found at the 5% level, hence no definitive conclusion can be made about this difference of the total score between experts and non-experts in phase 2.

5. ANALYSIS 4: CONFUSION MATRIX

Table 38. Confusion matrix for DCR predictions after using the XAI for all physicians.

	Physician correct		Physician wrong	
XAI correct	0 (0.0%)	85 (42.5%)	35 (17.5%)	12 (6.0%)
XAI wrong	20 (10.0%)	9 (4.5%)	0 (0.0%)	39 (19.5%)
	Physician changes after XAI advice	Physician stays after XAI advice	Physician changes after XAI advice	Physician stays after XAI advice

Considering the 114 cases where a physician was right, 20 physicians (17.5%) changed opinion after using the XAI and were wrong, whereas 9 (7.9%) stayed with their initial evaluation and were right.
Considering the 29 subcases where a physician was right and the XAI was wrong, 20 physicians (69.0%) changed opinion after using the XAI and were wrong, whereas 9 (31%) stayed with their initial evaluation and were right.

With regard to the 86 cases where a physician was wrong, 35 physicians (40.7%) changed their opinion after using the XAI and were right, while 12 (14.0%) stayed with their wrong evaluation.
With regard to the 47 subcases a physician was wrong and the XAI was right, 35 physicians (74.5%) changed their opinion after using the XAI and were right, while 12 (25.5%) stayed with their wrong evaluation.

Table 39. Confusion matrix for DCR predictions after using the XAI for expert physicians.

	Physician correct		Physician wrong	
XAI correct	0 (0.0%)	40 (40.0%)	18 (18.0%)	8 (8.0%)
XAI wrong	13 (13.0%)	5 (5.0%)	0 (0.0%)	16 (16.0%)
	Physician changes after XAI advice	Physician stays after XAI advice	Physician changes after XAI advice	Physician stays after XAI advice

Considering the 58 cases where an expert was right, 13 experts (22.4%) changed opinion after using the XAI and were wrong, whereas 5 (8.6%) stayed with their initial evaluation and were right.
Considering the 18 subcases where an expert was right and the XAI was wrong, 13 experts (72.2%) changed opinion after using the XAI and were wrong, whereas 5 (2.8%) stayed with their initial evaluation and were right.

With regard to the 42 cases where an expert was wrong, 18 experts (42.9%) changed their opinion after using the XAI and were right, while 8 (19.0%) stayed with their wrong evaluation.
With regard to the 26 subcases a physician was wrong and the XAI was right, 18 experts (69.2%) changed their opinion after using the XAI and were right, while 8 (30.8%) stayed with their wrong evaluation.

Table 40. Confusion matrix for DCR predictions after using the XAI for non-expert physicians.

	Physician correct		Physician wrong	
XAI correct	0 (0.0%)	45 (45.0%)	17 (17.0%)	4 (4.0%)
XAI wrong	7 (7.0%)	4 (4.0%)	0 (0.0%)	23 (23.0%)
	Physician changes after XAI advice	Physician stays after XAI advice	Physician changes after XAI advice	Physician stays after XAI advice

Considering the 56 cases where a non-expert was right, 7 non-experts (12.5%) changed opinion after using the XAI and were wrong, whereas 4 (7.1%) stayed with their initial evaluation and were right.
Considering the 11 subcases where a non-expert was right and the XAI was wrong, 7 non-experts (63.6%) changed opinion after using the XAI and were wrong, whereas 4 (36.4%) stayed with their initial evaluation and were right.

With regard to the 44 cases where a non-expert was wrong, 17 non-experts (38.6%) changed their opinion after using the XAI and were right, while 4 (9.1%) stayed with their wrong evaluation.
With regard to the 21 subcases a non-expert was wrong and the XAI was right, 17 non-experts (81.0%) changed their opinion after using the XAI and were right, while 4 (19.0%) stayed with their wrong evaluation.

Supplementary Information 7

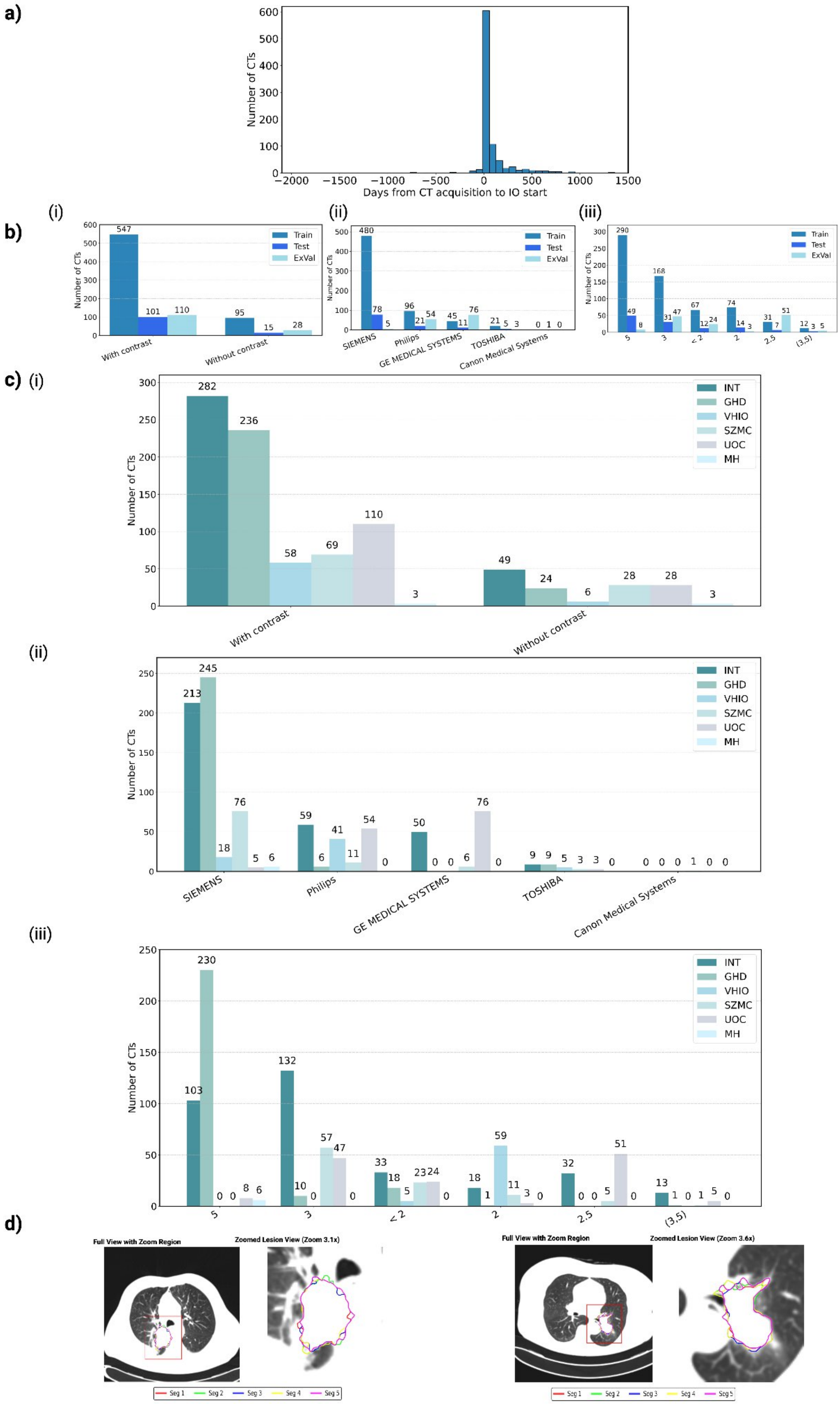

Figure 1. Metadata analysis for CT scans. a) Distribution of CT date of acquisition from IO start; b) (i) contrast information, (ii) manufacturer and (iii) slice thickness distributions across train, test

and ExVal; c) (i) contrast information (ii) manufacturer (iii) slice thickness grouped by center; d) example of CT segmentation with perturbations.

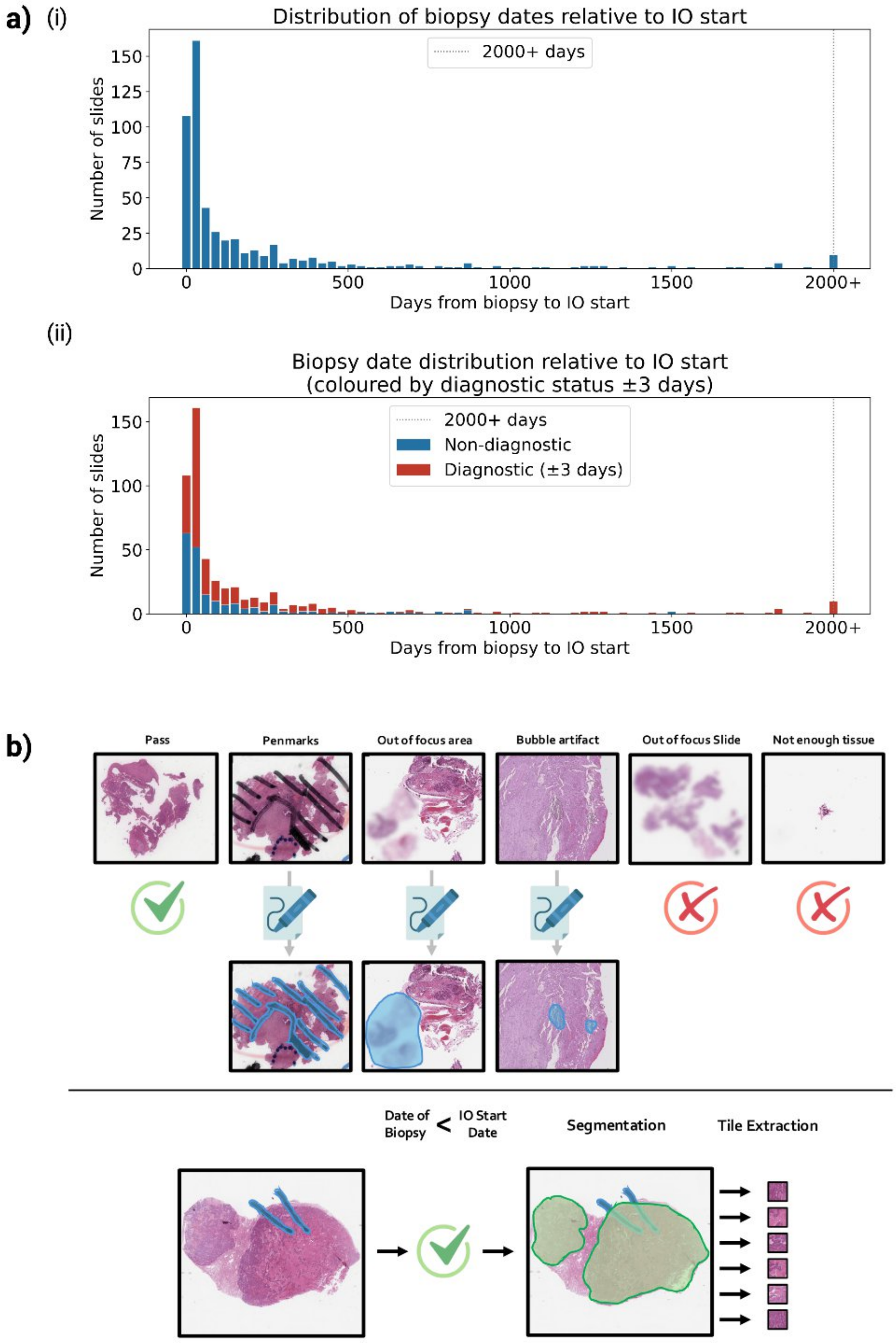

Figure 2. Metadata analysis for H&E slides. a) (i) distribution of biopsy date relative to IO start, (ii) distribution of biopsy date relative to IO start (coloured by diagnostic status ± 3 days); b) H&E slides quality control: first the slides are individually reviewed and artifacts and penmarks are manually annotated. Then, we exclude slides from biopsies that were not conducted after the IO start date. Finally the areas of the tumor are segmented. Only the tiles within the area of the tumor and outside the segmented artifacts are used for the analysis.

Table 1. a) MLEF classification results of full data-driven features on OS24, OS6, and DCR for CV, Test and ExVal; b) Cox-MLEF results on CV, test and ExVal

a)

Outcome	Dataset	F1-macro	AUC	Sensitivity	Specificity
OS24	CV	0.58 ± 0.06	0.68 ± 0.03	0.62 ± 0.05	0.64 ± 0.07
	Test	0.62	0.70 ± 0.09	0.65	0.66
	ExVal	0.55	0.63 ± 0.08	0.45	0.69
OS6	CV	0.63 ± 0.05	0.72 ± 0.03	0.67 ± 0.08	0.61 ± 0.13
	Test	0.64	0.70 ± 0.07	0.74	0.56
	ExVal	0.51	0.68 ± 0.08	0.92	0.12
DCR	CV	0.63 ± 0.04	0.71 ± 0.03	0.69 ± 0.13	0.58 ± 0.17
	Test	0.66	0.71 ± 0.03	0.68	0.63
	ExVal	0.40	0.55 ± 0.07	0.18	0.76

b)

Outcome	Dataset	C-INDEX
OS	CV	0.66 ± 0.02
	Test	0.66 ± 0.04
	ExVal	0.65 ± 0.05

Table 2. Sensitivity analysis removing EGFR as a driver. a) Sensitivity analysis for C2 (genomics); b) Sensitivity analysis for C2 (genomics); c) Sensitivity analysis for C23 (clinical + genomics); d) Sensitivity analysis for C2 (clinical + genomics).

We compared two approaches: Original, using the original composite driver (EGFR/ALK/ROS1/RET), and Modified, separating EGFR as an independent feature while retaining ALK/ROS1/RET as a composite. AUCs were compared via DeLong's test with 95% confidence intervals.

a)

Outcome	n	Original Driver CV AUC	Modified Driver CV AUC	p-value
OS6	1176	0.50 ± 0.00028	0.50 ± 0.0011	0.79
OS24	1003	0.51 ± 0.0014	0.52 ± 0.0012	0.86
DCR	1239	0.51 ± 0.00029	0.51 ± 0.001	0.84
CBR	1237	0.51 ± 0.00098	0.54 ± 0.001	0.3
ORR	1239	0.49 ± 0.001	0.55 ± 0.0011	1.49885e-09

b)

Outcome	n	Original Driver CV AUC	Modified Driver CV AUC	p-value
OS6	811	0.51 ± 0.0002	0.51 ± 0.00013	0.008

OS24	651	0.51 ± 0.0004	0.42 ± 0.002	0.001
DCR	870	0.45 ± 0.0012	0.55 ± 0.0012	0.018
CBR	869	0.54 ± 0.0012	0.57 ± 0.0012	0.36
ORR	870	0.54 ± 0.0012	0.50 ± 0.0002	0.04

c)

Outcome	n	Original Driver CV AUC	Modified Driver CV AUC	p-value
OS6	1176	0.69 ± 0.001	0.70 ± 0.001	0.49
OS24	1003	0.70 ± 0.0012	0.69 ± 0.0013	0.44
DCR	1239	0.67 ± 0.001	0.66 ± 0.001	0.27
CBR	1237	0.66 ± 0.001	0.66 ± 0.001	0.86
ORR	1239	0.61 ± 0.0011	0.63 ± 0.0011	0.27

d)

Outcome	n	Original Driver CV AUC	Modified Driver CV AUC	p-value
OS6	811	0.68 ± 0.0013	0.70 ± 0.0013	0.04
OS24	651	0.68 ± 0.0015	0.69 ± 0.0015	0.605
DCR	870	0.64 ± 0.0012	0.66 ± 0.0012	0.166
CBR	869	0.66 ± 0.0012	0.61 ± 0.0012	0.002
ORR	870	0.58 ± 0.0012	0.58 ± 0.0012	0.98

Comment: Where statistically significant differences occur, absolute performance remains uniformly low (AUC $\approx$ 0.5), indicating limited discriminative ability regardless of feature encoding. When including CB data, significant differences appear only for OS6 and CBR, while OS24, DCR, and ORR show no meaningful separation. Overall, the impact of EGFR stratification is modest and outcome-dependent. The decision to use a combined composite score is driven by substantial missingness in the available data; separating EGFR provides only minimal improvement in stratification. Full missing data analysis and EGFR separation experiments are reported in the Supplementary Information 1, Table 1.

Robustness analysis:

effect of contrast administration on feature robustness

Group definition

To assess whether a CT scan was performed with or without contrast, each scan was independently reviewed by radiologists, who classified them as contrast-enhanced or non-contrast. From the set used to perform PyRad feature selection we get that 640 CTs were contrast (C) enhanced and 100 CTs were non-contrast (NC).

Per-Group robustness metric

For each of the 128 selected features, the inverse coefficient of selection IS_f was recomputed independently within each subgroup $g \in \{C, NC\}$:

$$IS_f^{(g)} = \frac{\sigma_{intra,f}^{2(g)}}{\sigma_{inter,f}^{2(g)}}, g \in \{C, NC\}$$

where the group-specific intra-variance is:

$$\sigma_{intra,f}^{2(g)} = \frac{1}{L_g} \sum_{l=1}^{L_g} Var\left\{X_{l,p,f}\right\}_{p=1}^{P}$$

computed over the Lg lesions belonging to group g, and the group-specific inter-variance is:

$$\sigma_{inter,f}^{2(g)} = Var\left\{X_{l,p_0,f}\right\}_{l \in g}$$

where p_0 denotes the original (unperturbed) segmentation.

Hypotheses

To determine whether contrast administration systematically affects feature robustness, we computed the paired difference for each feature:

$$\Delta_f = IS_f^{(C)} - IS_f^{(NC)}, \ f = 1,\dots,128$$

A two-sided Wilcoxon signed-rank test was applied to the 128 paired differences. The hypotheses are:

$$H_0: median\left(\Delta_f\right) = 0$$
$$H_1: median\left(\Delta_f\right) \neq 0$$

Under H0, contrast administration does not introduce a systematic shift in feature robustness. The matched-pairs rank-biserial correlation r was reported as effect size.

Results

On the full (unbalanced) cohort (640 C vs 100 NC), the Wilcoxon signed-rank test was highly significant ($T = 1367$, $p < 0.0001$), with a large effect size ($r = 0.67$). The contrast group exhibited lower IS values (greater robustness) for 101 of 128 features (78.9%).

However, because the group sizes were highly unbalanced (640 C vs 100 NC), a subsampling analysis was performed to verify that the observed differences were not driven by the size asymmetry. A subsampling analysis was performed: in each of 1,000 iterations, 100 patients were randomly drawn

(without replacement) from the C group and the Wilcoxon test was repeated against the full NC group. Under balanced conditions ($n = 100$ vs 100), the effect attenuated substantially: the median p-value increased to 0.017, the median effect size decreased to $r = 0.24$, and the contrast group showed lower IS in only 60.9% of features (vs 78.9% in the full sample). The test reached significance at $\alpha = 0.05$ in 60.2% of iterations and at $\alpha = 0.01$ in 46.4%.

These results suggest that the strong effect observed on the full cohort is in part attributable to group-size imbalance. After correcting for this confound, the trend remains, but the effect is smaller ($r = 0.24$) and not completely consistent across resamples.
The 128 features were originally selected by ranking all features on IS_f computed over the entire pooled cohort. That cohort was ~86% contrast (640 out of 740). Both components of IS_f are dominated by the majority group: the intra-variance is a simple mean of per-patient standard deviations, so 640 of the 740 terms come from contrast patients; the inter-variance is the sample variance of the vanilla values across all patients, again overwhelmingly reflecting the contrast distribution. So, the feature selection was implicitly optimized for a population that is mostly contrast-enhanced.

Observational Study Protocol

Version n. 4.0 from 08/10/2025

PI: Prelaj Arsela, MD, Istituto Nazionale Dei Tumori di Milano

Title of the study	**I^{3}LUNG: Integrative science, Intelligent data platform for Individualized LUNG cancer care with Immunotherapy**
Background and Rationale	**BACKGROUND**<br><br>Lung cancer is the leading cause of cancer-related mortality worldwide. The global incidence in 2020 was an estimated 2.2 million cases with an estimated 1.8 million deaths. A 70% increase in the number of new cases of lung malignancy is expected over the next 2 decades (1). There are two major families of lung cancers: small cell lung cancer (SCLC), which accounts for approximately 15-20% of total cases, and non-small cell lung cancer (NSCLC), which accounts for approximately 80-85% (2). About 70% of patients with NSCLC, at the time of diagnosis, have advanced disease not amenable to surgical resection while SCLC is always considered a systemic disease (3). For several decades, cytotoxic chemotherapy has been the only treatment able to prolong survival in advanced patients with both SCLC and NSCLC. In recent years, in NSCLC, progress in cell biology have led to the identification of specific genetic alterations, known as "driver" alterations, at the basis of tumorigenesis, including mutations of EGFR, KRAS and BRAF and rearrangements of ALK, ROS1, RET genes. The use of "target therapies" is now the treatment choice in this subgroup of patients (4-11). The advent of immunotherapy has further changed the therapeutic landscape in both NSCLC without "driver" alteration (NSCLC-WT) and SCLC. This is a treatment modality based on mobilization of the immune system in order to recognize and destroy tumor cells. Immune Checkpoint Inhibitors (ICIs), have been developed with the intent to act on those pathways of "self-tolerance" used by tumors to escape recognition and destruction by the immune system. By blocking these inhibitory pathways, which physiologically control the immune response, ICIs reactivate and sustain the immune system response against tumors cells (12). Cytotoxic T-lymphocyte-associated-4 (CTLA-4) and programmed cell death protein 1 (PD-1) are receptors expressed on T cells that, by interacting with CD80/CD86 (13) and programmed cell death protein 1 (PD-L1) or PD-L2 ligands, respectively (14), can promote and foster immune evasion by tumor cells. PD-L1 evaluation on tumor remains the only used and approved predictor of response to immunotherapy (IO) treatment. Although the probability that a patient will benefit from IO treatment is higher in high PD-L1 expression, the role of PD-L1 remains unclear as a predictive role (15-20).<br><br>As a general guideline, the lack of alterations in target genes and PD-L1 expression > 50% allow to candidate a patient for first-line treatment, single-agent IO or its combination with chemotherapy based on platinum regimen (CHT). In patients with PD-L1 expression 1-49%, IO alone showed modest activity, while the CHT-IO therapy demonstrated to significantly prolong patient survival compared to CHT alone, in both squamous and non-squamous NSCLC (10).<br><br>To date, FDA and EMA approved for use in advanced NSCLC patients of nivolumab and pembrolizumab (antibodies against PD-1), and atezolizumab and durvalumab (antibodies against PD-L1). Due to the better efficacy and safety profile compared with chemotherapy, pembrolizumab, nivolumab, and atezolizumab were approved as monotherapies, pembrolizumab as first and second line treatment, whereas nivolumab and atezolizumab only in second line (15-19). Durvalumab is

instead indicated as maintenance therapy in patients with locally advanced NSCLC undergoing concomitant chemo-radiotherapy treatment (20).

Approximately 16% of NSCLC patients are still alive at 5 years compared to 5% with cytotoxic CHT. However, about 50% of patients do not obtain a real long-term benefit from this treatment modality. (21) Furthermore, in subjects carrying driver alterations, no significant advantage of single-agent IO over CHT has been reported (15-20). Lastly, there is a subgroup of 13-26% patients for whom IO treatment may even be detrimental, inducing a hyper-progressive disease (HPD) (22-26). Beyond PD-L1, several other biomarkers have been identified and used to profile patient prediction, including Tumor Mutational Burden (TMB) (27), tumor microenvironment (TME) (28), microRNA (miRNA) (29), immune gene signatures (30), gut microbiome (31), radiomics (32), or baseline clinical features or their combination in different scores (33,34).

Radiomics regards the quantitative analysis of digital imaging, informs future precision medicine, and supports objective decisions for cancer diagnosis and treatment. MAASTRO clinic has successfully experienced and validated fifty-six 3D-radiomic features. Recently, radiomics has demonstrated correlation between CT images and clinicopathologic features in NSCLC-IO patients (35). Finally, radiomics have been lately applied to PET/CT, in which context metabolic tumor volume (MTV) and total lesion glycolysis (TLG) in NSCLC demonstrated correlation with response to IO (36). There are several ongoing EU projects that use radiomics for NSCLC, but none of them is IO-specific and integrates multi- OMICs.

In the last decade, an impressive amount of knowledge has been achieved regarding the many other putative predictive factors, but no one has been applied in clinical practice. This is mainly due to unstructured and spared data among the different institutions, generating less consistent and therefore, less predictive datasets. Today, the medical community is claiming for an overall integration of clinical data and molecular biomarkers, in order to generate predictive information to address therapeutic administration in cancer patients, specifically in the immunotherapy era. In fact, it is unlikely that a single biomarker will profile prediction or prognosis with a high accuracy considering the role played by the immune system, and the complexity of the TME.

Artificial Intelligence (AI) frameworks and Machine Learning (ML) processes synthesize and correlate information from multiple sources and develop learning methods from it. These are a powerful, innovative, and potentially highly efficient instrument to construct decision-making (DM) tools providing individualized prediction of response to treatment and finally cope with the overmentioned complexity (37).

Therefore, the I³LUNG project supports a set of patient-centered ML tools designed and validated for the project, which make use of the novel virtual patient AVATAR entity for predicting progression and outcome. To maximize its impact, the use of Trustworthy Explainable AI (XAI) methodology will integrate the AI's inherent performances with the input of Human Intuition (HI) to construct a responsible AI application able to fully implement truly individualized treatment decisions in NSCLC interpretable and trustworthy for clinicians. (37) The final objective is the establishment of a European (potentially worldwide) Data Sharing and Elaboration Platform (DSEP), presented in Figure 1. The DSEP will provide guiding tools for patients, providing information to generate awareness on treatments. Lastly, it gives access to researchers and the general scientific community to the most up-to-date data sources on NSCLC.

Within the I3LUNG project, an ad-hoc Individual Patient Decision Aid System (IPDAS) for NSCLC patients will be developed. Patient decision aids are tools that might be used by patients either before

or within a consultation with physicians. Patient decision aids explicitly represent the decision to be made and provide patients with user-friendly information about each treatment option by focusing on harms and benefits. They allow patients to clarify what matters to them the most. These decisional support systems have been demonstrated to be effective in empowering patients, improving their knowledge, promoting their active participation in clinical decision-making about treatments, and improving overall patient satisfaction with care while decreasing decisional conflict and decisional regret (38-41) (see Psychological Study Annex I).

Finally, within the I^{3}LUNG project it will be assessed whether using the IPDAS during the clinical consultation would foster the quality of the shared decision-making as well as the quality of the doctor-patient communication. Alongside the evaluation of the impact of the IPDAS, it will be also evaluated whether the inclusion of the AI/ML predictive models in clinical practice will be added value in supporting oncologists' clinical decision-making and decreasing cognitive fatigue and decisional conflict (see Psychological Study Annex II).

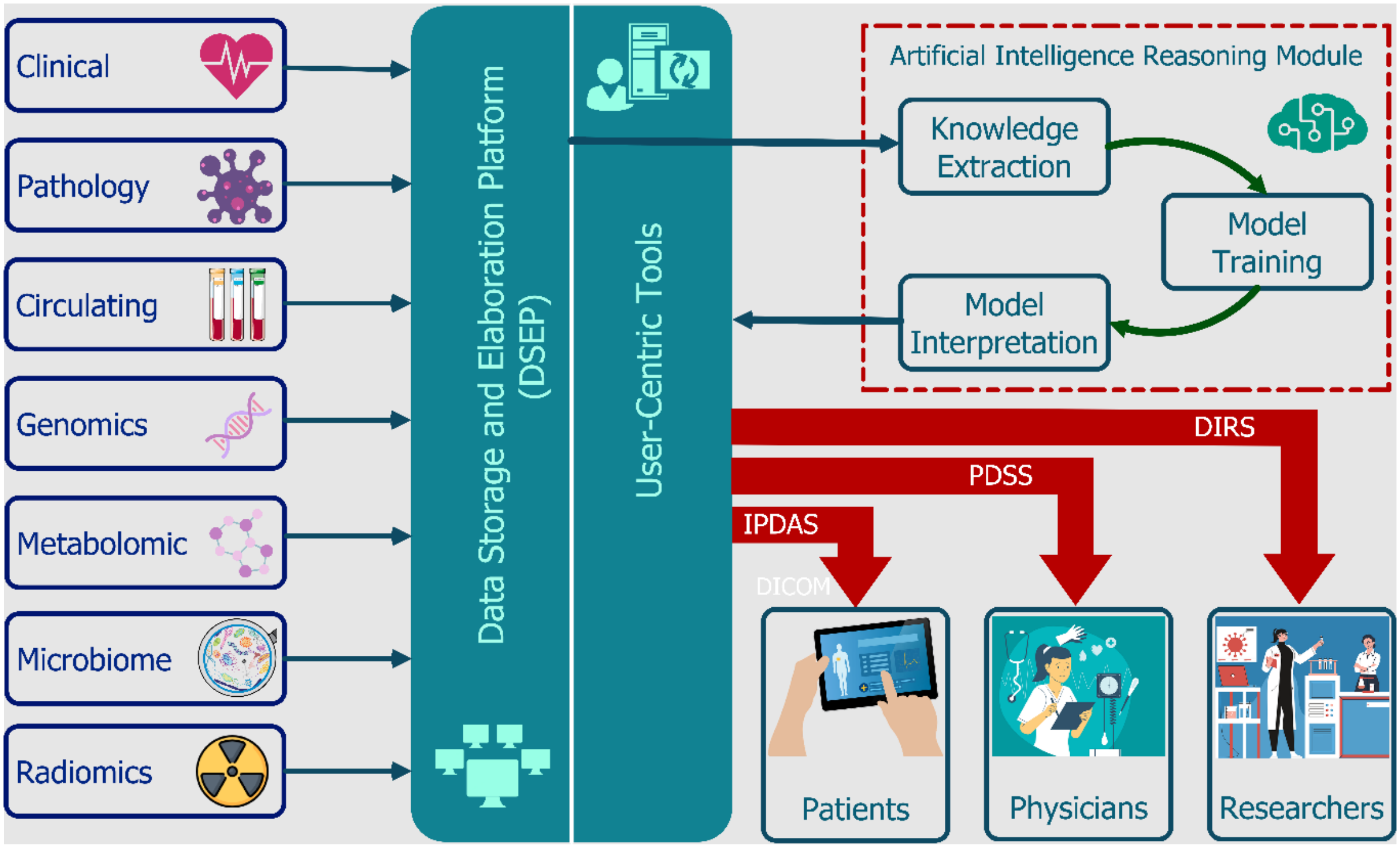

Figure 1. I^{3}LUNG platform, tools, and overall structure. The AVATAR based platform and the AI/ML based tools.

I^{3}LUNG will foresee two study phases: Retrospective and prospective.

I^{3}LUNG adopts a two-pronged approach to develop a medical device through the creation and validation of retrospective and prospective AI-based models to predict immunotherapy efficacy for NSCLC patients using the integration of multisource data (real word and multi-omics data) through a retrospective – setting up a transnational platform of available data from 2000 patients – and a prospective – multi-omics prospective data collection in 200 NSCLS patients – study phase.

The retrospective part of the I^{3}LUNG project includes the analysis of a multicentric retrospective cohort of more than 2,000 patients. This cohort will be used to perform a preliminary knowledge extraction phase and to build a retrospective predictive model for IO (R-Model), that will be used in the prospective study phase to create a first version of the Physician Decision Support System

	(PDSS) tool, an AI-based tool to provide an easy and ready-to-use access to predictive models, increasing care appropriateness, reducing the negative impacts of prolonged and toxic treatments on wellbeing and healthcare costs. Also, CT and PET scans will be collected and a first radiomic signature will be created to feed the R-Model.<br><br>**The prospective part** of the project includes the collection and the analysis of multi-OMICs data from a multicentric prospective cohort of about 200 patients. This cohort will be used to validate the results obtained from the retrospective model through the creation of a new model (P-Model), which will be used to create the final Physician Decision Support System (PDSS) tool.
Objectives and clinical relevance	**Overall Primary Objective**<br><br>● Prediction of response to immune checkpoint inhibitors in NSCLC by using clinical, biological, metabolomics, genomics, pathomics and radiomics data.<br><br>**Secondary Objectives**<br><br>● Prediction of Progression-free survival (PFS) in response to immune checkpoint inhibitors in NSCLC by using clinical, biological, metabolomics, genomics, circulomics, pathomics, microbiome and radiomics data.<br>● Prediction of Objective Response Rate (ORR) in response to immune checkpoint inhibitors in NSCLC by using clinical, biological, metabolomics, genomics, circulomics, pathomics, microbiome and radiomics data.<br>● Prediction of Overall survival (OS) in response to immune checkpoint inhibitors in NSCLC by using clinical, biological, metabolomics, genomics, circulomics, pathomics, microbiome and radiomics data.<br><br>**Exploratory Objectives**<br><br>● Added value of PET/CT into the prediction model for the Primary and Secondary Objectives.<br>● Prediction of adverse events to immune checkpoint inhibitors in NSCLC by using clinical, biological, genomics, pathomics and radiomics data.<br><br>Evaluate the addition of sarcopenia analysis into the prediction model to the response of immune checkpoint inhibitors in NSCLC.<br><br>**Specific Objectives:**<br><br>**Retrospective Study Phase**<br><br>The primary objective of the retrospective clinical study within the I3LUNG project is to create a model that will be used in the prospective phase to compare the predictive accuracy of ML methods (created using multi-OMICs analyses) with current validated biomarkers (e.g., PD-L1 or available scores). All available jeopardized data of 2000 aNSCLC patients treated at any point with any IO-based therapy will be collected in the first 18 months of the I3LUNG project, including all the data and radiomics detailed in the "Scientific Clinical Study Design" section. This model will be used to create a first version of the Physician Decision Support System (PDSS) tool.<br><br>**Prospective Study Phase**<br><br>The primary objective of the prospective phases to compare the predictive accuracy of ML methods

(created using multi-OMICs analyses) to current validated biomarkers (e.g., PD-L1). In this prospective part of the study, multi-OMICs data (pathology, genomics, transcriptomics, metabolomics, circulating immune profiling, microRNA signature classifier, liquid biopsy, microbiome) of 200 patients recruited by 5 cancer centers from the Consortium (INT, GHD, VHIO, MH and SZMC) will be collected and then integrated in the platform and used to feed the AI models learned on retrospective data, previously collected in the retrospective study phase.

Other objectives of the prospective phase in particular will include:

1. Integrate both retrospective and prospective data to improve and refine the predictive capabilities of the AI models compared to existing models;
2. Integrate radiomics signature originating from both Computed Tomography (CT) and Positron Emission Tomography–CT (PET-CT) images of the prospective cohort to refine and validate the retrospective radiomics signature;
3. Measure, within the prospective cohort new sensor-based QoL model and behavior data (e.g.: cough, physical and social activities, and mood states/emotions) that complements standard QoL measures (Patient Reported Outcome Measures or PROMs and Patient Reported Experience Measures or PREMs); the ad-hoc developed sensor- based monitoring system of patients' QoL and standardized PROMs and PREMs (ii) if co-shared decision-making impact QoL at least +10% compared to published prospective study data (Figure 1)
4. Use AI/ML to create new models in order to increase cognitive abilities of patients and physicians in shaping trust, usability, acceptability and attitude towards AI/ML;
5. Provide guidelines for an efficient collection of data to support diagnosis and select treatment in NSCLC will be developed thanks to the validated AI and Explainable AI (XAI) techniques;
6. Put in place preliminary tasks for the CE marking certification and future exploitation of the "medical device developed tools".

Clinical relevance

Currently, no clear data support the use of any biomarker, beyond PD-L1, to predict the patients benefit from IO and clinicians are often left alone to make decisions. The I³LUNG study aims to combine different types of markers (clinical, radiologic, genetic, molecular and immunological) through Artificial Intelligence (AI) frameworks and Machine Learning (ML) techniques. This will permit to fit it in a model that will be tested and integrated in the prospective phase of the study. In the final scenario, a pragmatic tool will be provided to clinicians in order to help predicting the benefit of IO, choosing the best candidate to immunotherapy and finally guide the therapeutic choice (IO alone vs IO-CHT combinations vs IO-IO combinations).

<u>Primary endpoint</u>

The predictive model will be developed through artificial intelligence and machine learning analysis of the integrated clinical, biological, genomics, metabolomics, circulomics, pathomics, microbiome and radiomics data.

<u>Secondary endpoints</u>

- Using the predictive model index as classificatory biomarker in Progression-free survival (PFS), defined as the time interval from the date of the first dose of the study treatment until the first date at which disease progression is objectively documented, or death due to any

Study Endpoints	cause, whichever occurs first and calculated through censored Kaplan-Meier analysis.<br>● Prediction of Objective response rate (ORR) classification through the predictive model and compared to the assessed by the investigator and defined as the proportion of the participants with confirmed complete response (CR) or partial response (PR) according to Response Evaluation Criteria in Solid tumors (RECIST) v1.1, after initiation of immune checkpoint inhibitors treatment.<br>● Using the predictive model index as classificatory biomarker in Overall survival (OS) calculated from diagnosis to death or last follow-up through censored Kaplan-Meier analysis.<br><br>**Exploratory endpoints**<br><br>● If available PET/CT will be added to the prediction model and the increment in the prediction performance will be analyzed for the primary and secondary endpoints and the adverse events categories.<br>● Machine learning algorithm will be used to predict adverse events categories in response to immune checkpoint inhibitors treatment through the integration of the clinical, biological, genomic, pathomics and radiomics.<br><br>Sarcopenia will be calculated through radiomics by measuring the fat and muscular mass on L1 slice of the chest CT scan and added to the prediction model for the Primary and Secondary Objectives.
Study population	RETROSPECTIVE PHASE:<br><br>The study cohort consists of aNSCLC patients treated with IO. Data from an estimated 2000 patients treated with IO-based therapy (any treatment line) in the period 2012 - until approval of the EC in each site (patients are already ongoing with immunotherapy or previously received or starting immunotherapy as second or further line) will be collected from all the clinical partners (INT, GHD, VHIO, MH, SZMC and UOC), patient inclusion and data registration starts at M3 until Jan 2026 . Inclusion criteria will be checked and informed consent for the study and for the re-use of prior collected medical data will be obtained before enrolment. If not feasible, i.e. patients not alive, the approval to Privacy Guarantee will be obtained.<br><br>**Inclusion criteria** retrospective phase:<br><br>1. Age ≥ 18 years<br>2. Presence of an Eastern Cooperative Oncology Group (ECOG) performance status ≤ 2;<br>3. Histologically confirmed diagnosis of stage III-IV Non-Small-Cell Lung Cancer patients receiving also maintenance therapy with durvalumab or other immune/combination are allowed;<br>4. Patients who received any line of IO-based therapy (immunotherapy monotherapy or immunotherapy in combination with any other treatments);<br>5. Patients with any CNS mets (stable or not) are allowed.<br>6. Patients with driver alteration (e.g., EGFR, BRAF, KRAS mutations or ALK, ROS1) who underwent an immunotherapy line will be included.<br><br>**Exclusion criteria** retrospective phase:<br><br>1. Patients without minimal treatment information data (start date, conclusion date (or

minimum 3 months follow up); best response (PD vs SD vs PR vs CR); setting (line of therapy, administration - alone vs combo).

PROSPECTIVE PHASE:

In the prospective phase, the study cohort consists of aNSCLC patients candidate for first-line IO-based therapy with available surgical samples (enough to perform OMICs). Baseline data of an estimated 200 patients from 5 clinical centers (INT, GHD, VHIO, MH and SZMC) will be collected including complete clinical, multi- OMICs analysis, imaging of CT and PET scan at baseline IO, behavioral, health economic, QoL measurements with based-sensor techniques and standard QoL. Data treated with IO-based therapy (any treatment line) will be collected. Inclusion criteria will be checked and informed consent for the study and for the re-use of prior collected medical data will be obtained before enrolment. Please refer to appendix 1 to see a workflow for the inclusion of these patients.

Inclusion criteria for prospective phase:

1. Age >/= 18 years.
2. Evidence of a personally signed and dated ICF indicating that the patient has been informed of and understands all pertinent aspects of the study before enrolment.
3. Eastern Cooperative Oncology Group (ECOG) performance status " 0-2.
4. Histologically confirmed diagnosis of stage IIIB/C-IV Non-Small-Cell Lung Cancer and large cell neuroendocrine carcinoma of the lung (LCNEC)
5. No activating EGFR mutations or ALK/ROS1 rearrangements.
6. No prior treatment for advanced disease.
7. Availability of at least 1 (one) FFPE block for -omics data generation (for cohort 200 pts).
8. Clinical indication for frontline treatment with immunotherapy (alone or in combination), as first line of treatment for metastatic disease or consolidation treatment for locally advanced disease.
9. Patients with untreated or unstable CNS brain metastases are allowed.

Exclusion criteria for prospective phase:

1. Diagnosed with small cell lung cancer histology, typical and untypical carcinoid.
2. Prior systemic treatment for NSCLC for advanced disease.
3. Absence of sufficient amount of tumor tissue for -omics data generation for OMIC cohort (200 pts).

The use of the app mobile and the completion of the psychological questionnaires should be offered to all prospective patients. However in case a patient does not accept to use it or to complete it, the patient can still be included in the study, if all criteria above are complied, in the corresponding cohort; omic or real world data.

Data for –omics analysis from patients considered screening-failure and, therefore, not eligible for

	the prospective phase of the protocol, will also be collected to feed the AI models using their real-world data generated during IO-based treatments. There is no limit for sample size in this phase and are not counted within the 200 patients who will be eligible for the multi-OMICs analysis.
Methodology and study design	**Scientific Clinical Study Design**<br><br>I[3]LUNG will conduct an observational retrospective/prospective multicenter international project on advanced Non-Small-Cell Lung Cancer patients treated with IO. Its main objective is to study, train and validate the predictive accuracy of ML methods created by using real world data and multi-OMICs analyses, compared to the current standard of care biomarkers (e.g., the PD-L1).<br><br>The retrospective study part enrolment and the registration of all the clinical data available will start at month 3 and will continue until August 2023. Patient diagnosis should have been from 2012 until EC approval.<br><br>The prospective enrolment will start at month 3 until January 2026. Once the recruitment has been finalized, there will be a follow-up period of 18 months with an expected end of study date in May 2027. Patients with NSCLC (n=200) candidates to first-line IO-based therapy with available surgical samples or biopsy samples (enough to perform -omics) will be enrolled.<br><br>For both phases, there are minimum required data to enroll/consider patients in the analysis.<br><br>**The minimum data required for Retrospective part is:**<br><br>- Immunotherapy treatment: start date, conclusion date (or a minimum follow-up of 3 months; best response (PD vs SD vs PR vs CR); setting (line of therapy, administration - alone vs combo)<br><br>- Clinical staging (AJCC VIII ed)<br><br>- Performance Status (if feasible)<br><br>- Histology<br><br>- Pathology images (if feasible)<br><br>- PET/CT scan at baseline (if feasible)<br><br>**The minimum data required for Prospective-OMIC is:**<br><br>- All biological samples collected (tumor, blood and stool)<br><br>- Immunotherapy treatment: start date, setting (line of therapy, administration - alone vs combo)<br><br>- Clinical staging (AJCC VIII ed)<br><br>- Performance Status<br><br>- Histology<br><br>- Pathology images: At IO baseline, a scanned image ofhematoxylin and PD-L1 staining. Please, check the Lab Manual for details.<br><br>- PET/CT scan at baseline, 1st evaluation, best response and progression<br><br>For those patients considered in the Prospective phase but eventually included in the RWD group,

the minimum required data is the same as for retrospective patients.

Additionally, the sites can collect the following data for Retrospective (when available) andProspective phases:

Baseline patients' data	Retrospective phase<br>Jeopardized	Prospective phase<br>Homogenized
**Demographic characteristics** (age, gender, height, body weight, ethnicity, family history of cancer, Smoking History, asbestos exposure, alcohol consumption, drug use history).	✓	✓
**Medical history** (personal medical history of cancer and other severe chronic diseases, allergies and concomitant medications)	✓	✓
**Health-economic details** (site of treatment administra. Ption, lost workdays, etc.)	If available	✓
**Lifestyle factors** (diet and use of supplements, physical activity, daily heart rate, sleep patterns, Q. oL)		✓
**Clinical findings**:<br>- Vital signs (blood pressure, oxygen saturation, (BP, SpO2), Temp, breaths/min, pulse).<br>- Main symptoms leading to diagnosis, main symptoms at initiation of immunotherapy. Presence of main disease-related symptoms (shortness of breath, cough, pain and fatigue).<br>- Body weight/height/BMI/BSA and weight loss prior to diagnosis and prior to initiation of IO (kg/months).<br>- Major findings at clinical examination at diagnosis.	✓	✓
**Laboratory findings**: Hb, WBC, Neutrophils, Lymphocytes, NLR (neutrophil/lymphocyte ratio) at IO initiation and post-treatment change in NLR, Platelets, Albumin, LDH, Ferritin, Creatinine, Creatinine	✓	✓

	clearance, Na, ALP, Glucose, CRP.		
	**Tumor characteristics**: Grade, Ki 67, mutational status (e.g. EGFR, BRAF KRAS, ALK, ROS1) and PD-L1 expression, TNM and/or stage, Sites/organs of metastases. Time from initial diagnosis to first metastatic disease.	✓	✓
	**Treatment Characteristics**:<br>- previous treatments, previous platinum use and response,<br>- 1st line treatment combination (IO vs CT+IO or other IO combinations).<br>- Regimen duration, number of cycles, details of maintenance treatment. Best response to 1st line. Duration of response, PFS, OS.<br>- Toxicity details (type of toxicity, grade). Use of supportive medication (growth factors, anti-emetics).	✓	✓
	**Radiomics**	✓ (CT +PET)<br>Required: Before IO treatment (baseline) CT-PET scan (if available)<br>Optional: diagnosis, 1st evaluation, best response and progression	✓ (CT + PET scan)<br>Required: Before IO treatment (baseline) CT-PET scan, 1st evaluation, best response and progression<br>Optional: diagnosis
	**Immunohistochemistry** data (e.g., CD3, CD4, CD8, CD20, CD11c, CD68, PD-1, PD-L1 and TIGIT)		✓
	**Citomic:** Immune phenotype and single cell analysis of circulating populations (i.e. PBMCs, PMNs)		✓
	**Cytokines and MicroRNA signature (MSC test)**		✓

**Cell free DNA and ctDNA sequencing**		✓
**Genomics** analysis: tumor mutational burden (TMB), somatic copy number alterations, whole genome sequencing, immune infiltration transcriptomic profile		✓
**Metabolomics and Microbiome**		✓
**Scanned hematoxylin/eosin and PDL1 FFPE tumor slides**	✓ (PDL1 slides only if available)	✓

The clinical/synthetic data will be collected within I3LUNG web-based platform. To each patient will be assigned a code. The CRF form will be filled in from each center with a dedicated account and all these workflows will be addressed using the Data Management Plan. For the OMIC patients (prospective phase) the data entry should be within 30 days.

In particular, for the prospective phase the following multi-OMICs data will be collected for all 200 patients with available tissue, blood and stool. For details see Table 2 and Lab manual.

**Omics type**	**Collection**	**Analyses**
**Tissue-based**<br><br>**(Tasks 3.2, 3.3, 3.4)**	Formalin-Fixed Paraffin Embedded (FFPE) tissue blocks from NSCLC patients who underwent surgery or large biopsy will be collected. Every 5 collected samples, FFPE blocks will be shipped to GHD. GHD will then extract samples for immune profiling/TME and will prepare 4 total cores, 2 for genomic profiling and 2 for metabolomics.	· TME and Tissue Immune Profiling - Reference tissue spots with known expression of the target proteins will be added to each microenvironment Tissue Microarrays (ME-TMA)<br>· Genomic profiling (DNA and RNA extraction) and transcriptomics profiling.<br>Metabolomics, metabolite extraction for targeted and untargeted metabolomics analysis

**Blood-based**<br><br>**(Task 3.5)**<br><br>(NB: PNMs isolation and Single Cell analysis and<br><br>will be performed only in INT, because PNMs lability will not permit freeze-thaw).	30 ml of WB samples will be collected from each patient only at baseline therapy.<br><br>1x2 ml WB tube will be used for DNA extraction<br><br>1x2 ml K2EDTA for cytokines assay<br><br>2x10 and 1x6 ml K2EDTA tubes for PBMC, PNMs, miRNA Signature Classifier (MSC) and cell-free DNA (cfDNA)	· Circulating Immune profiling PBMCs by flow cytometry staining. Assessment of membrane or intracellular markers (T-cells, T-regulatory cells, natural killer cells, monocytes, neutrophils, myeloid derived suppressor cells, B-cells and T-cell exhaustion, immune suppression, activation, proliferation, cytotoxicity, polarizations)<br>· Single cell analysis – T-cell receptor RNA sequencing<br>· Cytokines plasma analysis<br>· MSC test - panel of lung cancer related miRNAs will be measured in plasma samples<br>· cfDNA and ctDNA sequencing.
**Microbiota analysis**<br><br>**(Task 3.6)**	Baseline IO stool samples of all prospective patients enrolled will be collected and shipped to INT each 10 samples for microbiome analysis.	· NGS libraries sequencing<br>· Metagenomic DNA and RNA extraction<br>Organism-specific gene hits will be assigned to the Kyoto Encyclopaedia of Genes and Genomes (KEGG) Orthology (KO) DB, metagenomes for each sample will be reconstructed into metabolic pathways
**Advanced Imaging**<br><br>**(Task 3.7)**	CT scans in both in retrospective and prospective study phase and PET-CT in prospective phase scans are taken to perform the radiomic signature. The images will be anonymized and uploaded in a dedicated platform in order to be centralized to BGU.	· Tumour and lymph nodes/metastasis manual segmentation, and interactive segmentation, using algorithms of varying complexities, already evaluated.<br>· Extraction of four classes of image features describing tumour features: i) intensity, ii) shape, iii) texture and iv) Fourier or wavelet features (also MTV and TLG for PET-CT).<br>Radiomic signatures: unsupervised clustering to reveal clusters of patients with similar radiomic expression patterns

Transcriptomics

Added to the WP 3.3, a new task to implement the knowledge regarding spatial transcriptomics and tumor response to IO is here included. High-quality FFPE tumor biopsy/surgery sections of retrospective NSCLC patients (around 15 samples) will be collected to get information on single-cell transcriptomics, spatial transcriptomics and multiplexed IF analysis. The samples will be sent only from INT to UOC partner.

Tumor Microarrays

Added to the WP 3.2, a new task to implement the knowledge regarding spatial interrelationship between tumor and immune cells in TME is here included. High-quality FFPE tumor surgery sections of retrospective patients will be sent from INT to UKE partner to increase the number of manufactured Tissue Microrrays (TMAs).

QoL Monitoring

Within the prospective part of the study, among all the prospective enroller centers (INT, GHD, MH, VHIO and SZMC) we will evaluate whether making decisions using our prediction system would lead to an improvement in patient's QoL using (i) the ad-hoc developed sensor- based monitoring system of patients' QoL and standardized PROMs and PREMs (ii) if co-shared decision-making impact QoL at least +10% compared to published prospective study data (Figure 6).

App Mobile

Sensor-based monitoring system of QoL This task foresees the development of a mobile application for the transparent monitoring of the patient's quality of life. It will include: an automatic cough detector system, an assessment of patient's mood state and emotional functioning, a human activities and gait recognition system and an evaluation of smartphone usage to monitor changes of social interactions and habits. Smartphone-based features collected daily will be correlated with clinical outcomes (e.g., monitoring toxicity and clinical benefit) collected at specific time points during the prospective clinical study in order to evaluate their capability to predict temporal variations of QoL, physical, social, and emotional functioning. Data collection within preliminary controlled studies are foreseen in this task to validate the app accuracy.

To this end, the recruitment of 500 healthy participants is expected as a control group. Participants will record their forced cough to compare the acoustic features extracted from the pathological group with those of the control group. The main aim is to detect differences in terms of cough, to possibly obtain a tool for the early detection of lung cancer, and to train a machine learning model to better monitor the evolution of the cough of NSCLC patients during therapy.

In addition to forced cough recordings, the study will incorporate free speech collection from both NSCLC patients and the healthy control group to develop a comprehensive multi-modal classifier system. Free speech samples will be collected through structured text reading and vocalization tasks, providing complementary acoustic features that may reveal disease-related changes in vocal patterns, respiratory function, and speech dynamics. All recordings will be digitally saved for subsequent analysis. This multi-modal approach combining forced cough analysis with spontaneous speech assessment will create a more robust diagnostic framework, as different vocal tasks may emphasize distinct pathological features.

To strengthen the robustness of the early detection algorithm, the study will extend recruitment beyond the healthy control group to include 300 Non-Small Cell Lung Cancer (NSCLC) patients at resectable early and resectable locally advanced disease stages (Stage I-III), with a focus on recruiting patients prior to surgical intervention. This expanded patient cohort will provide acoustic data from individuals with early-stage malignancies, enabling the machine learning model to identify

subtle pathological features that may be present before advanced symptomatology develops. The inclusion of Stage I-III NSCLC patients will bridge the gap between healthy controls and advanced-stage patients, creating a more comprehensive dataset that captures the full spectrum of disease progression. This multi-tiered approach will enhance the algorithm's sensitivity for early detection by training it to recognize nuanced acoustic patterns that emerge in the initial phases of lung cancer development. For this group of patients, presurgery RWD data, PET and CT scan images will be collected.

Moreover, for the assessment of the emotional status of the patients, to cover the complete spectrum of emotions to have a more robust machine learning model and validate the set of acoustic features, the healthy control group and Stage I-III NSCLC patients will be asked to record and act out their emotions, following pre-defined scripts validated in previous studies (42).

Using self-reported QoL measures to identify trajectories over time

Patients' lifestyle factors, daily habits, and quality of life will be collected at baseline and at IO Baseline, M1, M3, M12 and when progressive disease occurs through validated and self-reported measures. Physical activity, metabolic equivalent of task, and QOL will be assessed with the WHO Global Physical Activity Questionnaire. Moreover, patients will be administered with reliable, valid, and standardized cancer QoL measures such as EORTC QLQ-C30 , EQ-5D and lung cancer-specific QoL measures such as QLQ-LC13.

Image analysis methodology

The analytical process for the medical images will include:

- Data acquisition
- Image segmentation
- Analysis and features extraction

Data acquisition

Digital Imaging and Communications in Medicine (DICOM) medical images will be exported from the PACS system pseudonymized and uploaded to the central cloud server for Radiomics analysis.

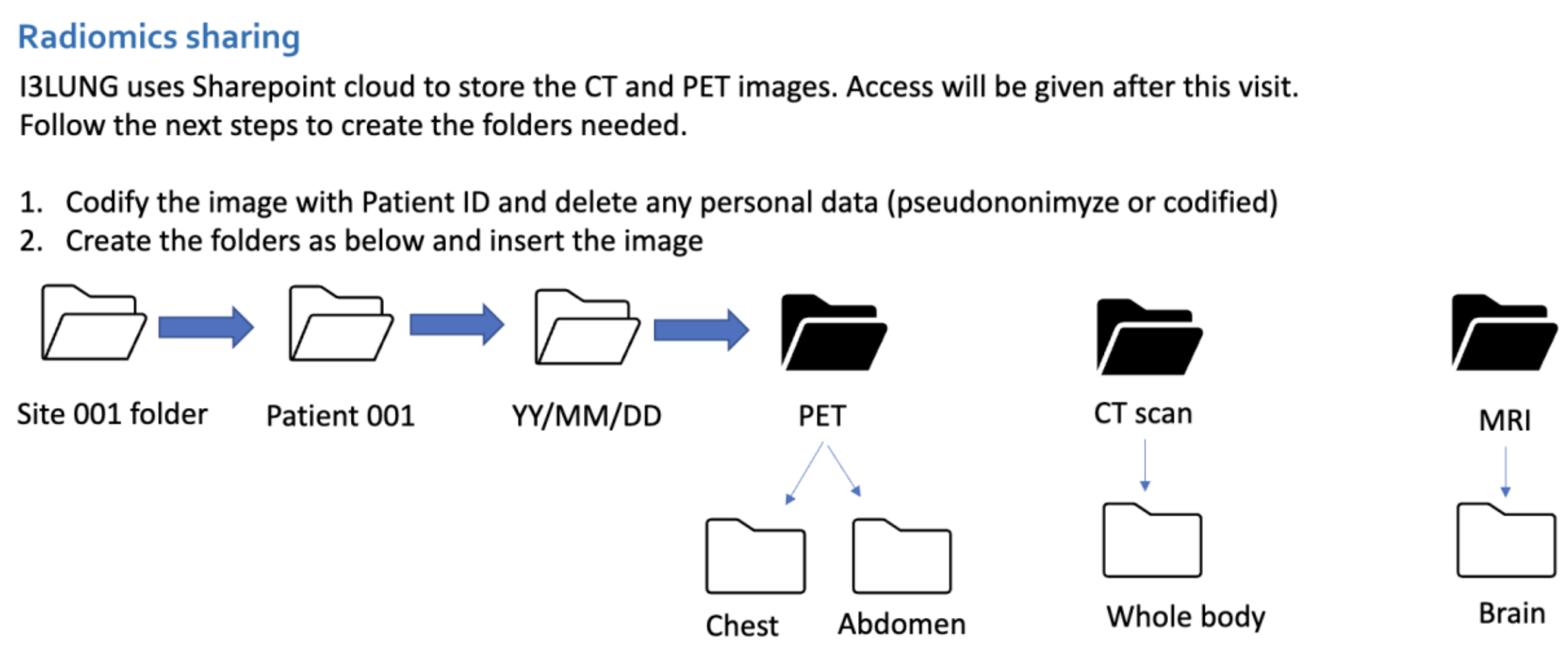

Figure 2. Storage naming in SharePoint cloud

Image segmentation

The pseudonymized DICOM images will be processed through automatic segmentation by artificial intelligence using deep learning algorithms to recognize the lung field and automatically detect and segment the lung tumor lesions.

An independent qualified radiologist will centrally review the results and performance of the tumor segmentation algorithm.

Image features extraction

The study will extract and analyze a list of radiomics features according to the Image Biomarker Standardization Initiative (IBSI) recommendations. Features extracted from the segmented lesions will characterize different properties of the images, including:

i) pixel intensity,
ii) lesion shape,
iii) lesion texture,
iv) Fourier or wavelet-based features.

The parameters set obtained by RFE will be used to create radiomic signature following three steps:

i) identification of cluster and possible expression patterns;
ii) evaluation of relationship between these patterns and tumor/patient's;
iii) generation of radiomic signature;

To remove redundancy within the radiomic information, one or two best-performing radiomic feature from each of the four-feature groups will be selected and combined into a multivariate Cox proportional hazards regression model for prediction of survival. For the training phase, feature stability will be assessed in inter-observer settings.

The radiomic signature will be developed in the first 1000 CT and PET-CT images and will be then validated in the ~1000 remaining patients. The generated and validated radiomic signature will be integrate to the R-model generated to improve its prediction and make the R-model.

Radiomic Signature

To assess the value of radiomic features to capture phenotypic differences of tumors, the features obtained by the RFE will be subjected to unsupervised clustering to reveal possible clusters of patients with similar radiomic expression patterns. For PET-CT metabolic tumor volume (MTV), total lesion glycolysis (TLG) and radiomics features such as volume and heterogeneity will be added to the analysis of PET-CT images.

The main clusters of patients will be related with other collected parameters, looking for association with tumor stage, histology etc. Finally, the generated radiomics signature will be validated and then integrated in the PDSS tool to improve prediction.

Artificial intelligence methodology

I[3]LUNG will develop a Data Sharing and Elaboration Platform (DSEP) that gathers and manages virtual entities, namely patient AVATARS, corresponding to real patients, and will provide a set of tools that integrate physician expertise and the knowledge extracted from the patient avatars data to create individualized predictions for NSCLC patients treated with IO. As depicted in Figure 2, data is gathered from the DB and a context is generated for each patient, which, in its turn, is fed to the ML models to make decisions.

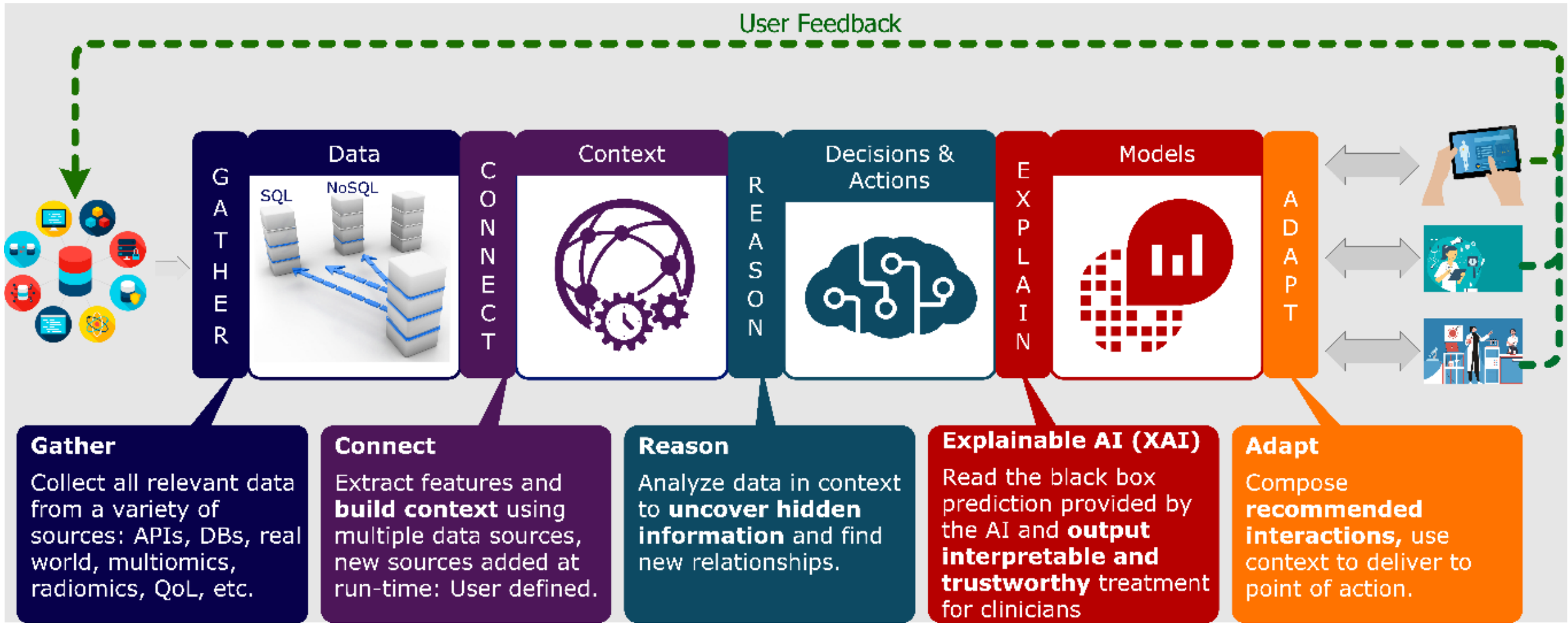

Figure 3. A summary of the ML pipeline we will adopt in the I[3]LUNG project.

Patient data collected through the I[3]LUNG platform will be utilized to prototype, train, and host validated ML predictive algorithms to help with prognosis and IO response predictions. A careful ML data preparation for each of the data types (real world, radiomics) is essential to build both independent and merged models. ML informed features will be derived from the raw data types.

Explainability of the AI Models

The aims of the Explainable AI (XAI) are to produce more explainable models while maintaining a high level of learning performance (e.g., prediction accuracy) and to enable humans to understand, appropriately trust, and effectively manage the emerging generation of AI partners.

In I[3]LUNG, a suite of XAI techniques will be used to make ML results interpretable and available to both clinicians and patients, comparing results with HI, so to support the clinical decision for the best IO-treatments for NSCLC, and the sharing of decisions with aware patients. XAI methodologies will be adopted and applied to the developed AI algorithms, such as the use of text, visual and local explanations, explanations by examples, and feature and input relevance (37). Moreover, simplifications to the model will be applied; for example, the creation of new models, based on the trained one, where part of the complexity is neglected without decreasing in the accuracy.

	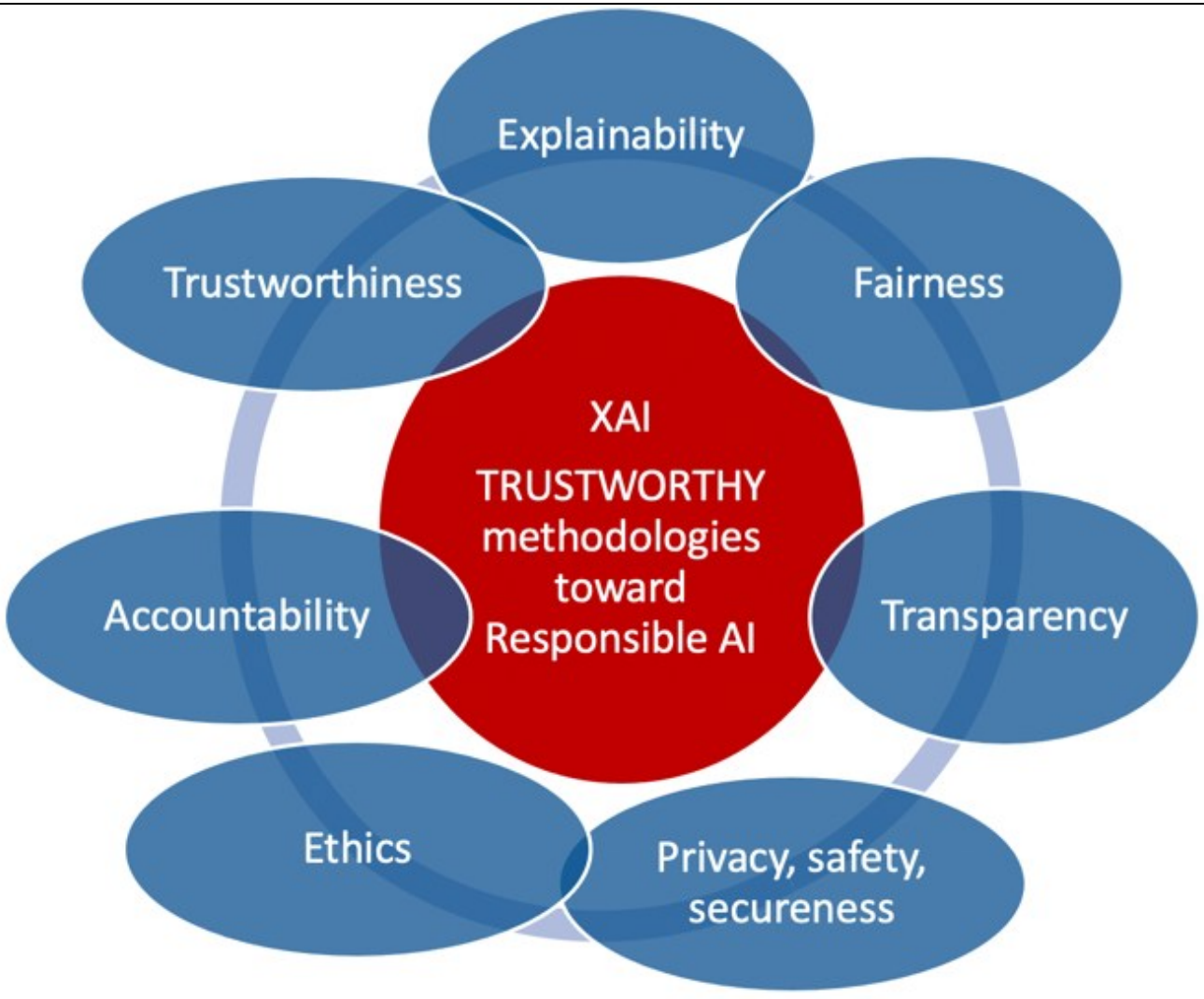<br><br>**Figure 4.** XAI methodology approaches.
Sample size and statistical analysis and AI analysis plan	For the retrospective phase, we have planned to enroll patients based on a first check availability of already treated patients with IO-based therapy among centres.<br><br>The primary objective of the study is to compare the predictive accuracy of ML methods (created using multi-OMICs analyses) with current validated biomarkers (e.g., PD-L1 or available scores, see Table 2). Considering the number of 200 prospective cases, the study will have at least 85% power to detect a 0.1 increase in predictive accuracy as measured by the area under the ROC curve (AUC), assuming an AUC for validated biomarker alone of at least 0.6, using a one-sided z-test at a significance level of 0,050. The data are assumed to be discrete (rating scale) responses. Stratification Factors: Country (Italy or others), Gender (male vs females), ECOG PS (0-1 vs 2), Histology (squamous vs non-squamous), Stage (III vs IV), PD-L1 status, and treatment administered (Chemo-Immuno vs immuno-alone vs others IO combinations) will be considered.<br><br>**AI analysis plan**<br><br>After Ethical approval in each Cancer Center, the third part of the study will start at month 3 and will continue up to 36 months. These periods will allow sufficient time for prospective enrolment and follow-up of patients for at least for 18 months. At month 30, an intermediate version of the P-Model will be released after the collection of the first batch (~100) of patients including multi-OMICs data. At months 54, after the predictive models are build using all the data from 200 patients, the final P-Model will allow to verify if: (i) new predictive system using multi-OMICs results perform better compared to current ones (e.g., PD-L1 or other state of the art biomarkers), (ii) if the model using -omics data perform better compared to the R model, (iii) if there is any specific -omics analysis which perform better compare to other -omics, which would give more value to the model (iv) if the P-Model perform better compared to current biomarker, and compare to the R-model when applied to specific subgroups of patients (e.g., non-squamous vs squamous patients). We will consider this phase successful if the new P-models outperforms current biomarkers prediction at least for 10% in terms of correct prediction of either 2-year PFS and 2-year OS, measured with Harrel's C statistic.
Ethical considerations	The study is conducted in accordance with the Declaration of Helsinki Ethical Principles and Good Clinical Practices and was approved at each site by an independent local ethics committee.<br><br>During the clinical visit, after receiving adequate study information, the patient may sign an

and Informed Consent	informed consent form, in accordance with the provisions of the local ethical regulations. regulations. The consent of the patient must be signed and personally dated by the patient and by the investigator/person designated to conduct the informed consent discussion. The signed and dated declaration of informed consent will remain at the researcher site and must be safely archived by the investigator so that the forms can be retrieved at any time for monitoring, auditing and inspection purposes. A copy of the signed and dated information and consent should be provided to the patient prior to participation.<br><br>In the retrospective part of the study, in case it is not possible to ask and obtain for the informed consent (due to death or inability to reach the patients), to process the patient data and samples, for ethical-administrative reasons, the study falls within the scope and purposes specified by the Regulation (EU) 2016/679 of the European Parliament and of the Council of 27 April 2016 on the protection of natural persons with regard to the processing of personal data and on the free movement of such data, and repealing Directive 95/46/EC (General Data Protection Regulation), in its article 89.1.<br><br>All clinical centers will be involved in the retrospective phase, while in the prospective phase only 5 out 6 centers will participate (INT, GHD, VHIO, MH and SZMC). The study will involve exporting of CT and PET images from both retrospective and prospective studies from EU cancer centers and US to Israel, while clinical data will be exported from all the EU cancer centers and Israel to US for AI analyses. Biological samples (tissue, blood and stool samples) will be collected from SZMC (Israel) and will be imported in EU countries (Italy, Germany, Spain) for pathology, genomic, circulating and microbiome analyses.
References	1. Globocan (2018) Available from http://globocan.iarc.fr/Default.aspx (Accessed on 25-Nov-2020).<br><br>2. Howlader N, et al. SEER Cancer Statistics Review, 1975–2012. National Cancer Institute, 2015 [resource on the Internet]. Available from: http://seer.cancer.gov/csr/1975_2012/http://seer.cancer.gov/csr/1975_2012/.<br><br>3. Sulpher JA, et al. Factors influencing a specific pathologic diagnosis of non-small-cell lung carcinoma. Clin Lung Cancer. 2013 May;14(3):238-44.<br><br>4. Siegel RL,et al. Cancer Statistics, 2017. CA Cancer J Clin 2017; 67:7–30.<br><br>5. Lynch TJ, et al. Activating mutations in the epidermal growth factor receptor underlying responsiveness of non-small-cell lung cancer to gefitinib. N Engl J Med 2004; 350: 2129-39.<br><br>6. Rosell R, et al. Screening for epidermal growth factor receptor mutations in lung cancer. N Engl J Med 2009; 361: 958-67.<br><br>7. Fukuoka M, et al. Biomarker analyses and final overall survival results from a phase III, randomized, open-label, first-line study of gefitinib versus carboplatin/paclitaxel in clinically selected patients with advanced non-small-cell lung cancer in Asia (IPASS). J Clin Oncol 2011; 29: 2866-74.<br><br>8. Maemondo M, et al. Gefitinib or chemotherapy for non-smallcell lung cancer with mutated EGFR. N Engl J Med 2010; 362: 2380-88.

9. Mitsudomi T, et al. Gefitinib versus cisplatin plus docetaxel in patients with non-small-cell lung cancer harbouring mutations of the epidermal growth factor receptor (WJTOG3405): an open label, randomised phase 3 trial. Lancet Oncol 2010; 11: 121-128.

10. Rosell R, et al. Erlotinib versus standard chemotherapy as first-line treatment for European patients with advanced EGFR mutation-positive nonsmall- cell lung cancer (EURTAC): a multicentre, open-label, randomised phase 3 trial. Lancet Oncol 2012; 13: 239-46.

11. Sequist LV, Yang et al. Phase III study of afatinib or cisplatin plus pemetrexed in patients with metastatic lung adenocarcinoma with EGFR mutations. J Clin Oncol 2013; 31: 3327-34.

12. Wu YL, et al. Afatinib versus cisplatin plus gemcitabine for first-line treatment of Asian patients with advanced non-small-cell lung cancer harbouring EGFR mutations (LUX-Lung 6): an open-label, randomised phase 3 trial. Lancet Oncol 2014; 15: 213-22.

13. Zhou C, et al. Erlotinib versus chemotherapy as first-line treatment for patients with advanced EGFR mutation-positive non-small-cell lung cancer (OPTIMAL, CTONG-0802): a multicentre, open-label, randomised, phase 3 study. Lancet Oncol 2011; 12: 735-42.

14. Soria JC, et al. Osimertinib in Untreated EGFR-Mutated Advanced Non–Small-Cell Lung Cancer. N Engl J Med 2018; 378:113-25

15. Mok TS, et al. Osimertinib or platinum–pemetrexed in EGFR T790M–positive lung cancer. N Engl J Med 2017;376:629-40.

16. Sullivan I, Planchard D. ALK inhibitors in non-small cell lung cancer: the latest evidence and developments. Ther Adv Med Oncol 2016;8:32-47.

17. Karachaliou N, , et al. Anaplastic lymphoma kinase inhibitors in phase I and phase II clinical trials for non-small cell lung cancer. Expert Opin Investig Drugs 2017;26:713-22.

18. Pardoll DM. The blockade of immune checkpoints in cancer immunotherapy. Nat Rev Cancer 2012;12:252–64.

19. Hathcock KS, et al. Identification of an alternative CTLA-4 ligand costimulatory for T cell activation. Science 1993; 262:905–7.

20. Thompson RH, et al. Indicator of tumor aggressiveness and potential therapeutic target. Proc Natl Acad Sci U S A 2004;101:17174–9.

21. Borghaei H, et al. Nivolumab versus docetaxel in advanced nonsquamous non-small-cell lung cancer. N Engl J Med. 2015; 373: 1627–39.

22. Brahmer J, et al. Nivolumab versus docetaxel in advanced squamous-cell non-small-cell lung cancer. New Engl J Med 2015

23. Herbst RS, et al. Pembrolizumab versus docetaxel for previously treated, PD-L1-positive, advanced non-small-cell lung cancer (KEYNOTE-010): a randomised controlled trial. Lancet. 2016; 387: 1540–50.

24. Fehrenbacher L, et al. Atezolizumab versus docetaxel for patients with previously treated non-small-cell lung cancer (POPLAR): a multicentre, open-label, phase 2 randomised

controlled trial. Lancet. 2016; 387: 1837–46.

25. Reck M, et al. Pembrolizumab versus Chemotherapy for PD-L1-Positive Non-Small-Cell Lung Cancer. N Engl J Med 2016;375:1823–33.

26. Antonia SJ, et al. Durvalumab after Chemoradiotherapy in Stage III Non–Small-Cell Lung Cancer. N Engl J Med 2017; 377:1919-29.

27. Gandhi L, et al. Pembrolizumab plus Chemotherapy in Metastatic Non-Small-Cell Lung Cancer. N Engl J Med 2018;378:2078-92.

28. Paz-Ares L, et al. Durvalumab plus platinum-etoposide versus platinum-etoposide in first-line treatment of extensive-stage small-cell lung cancer (CASPIAN): a randomised, controlled, open-label, phase 3 trial. Lancet 2019;394:1929-39.

29. Brahmer JR, et al: five-yer follow-up from the CA209-003 study of nivolumab in previously treated advanced non small cell lung cancer: clinical characteristics of long-term survivors. Presented at 2017 AACR Annual meeting Washington, DC, USA, 1-5 April 2017.

30. Adashek JJ, et al. Hyperprogression and Immune Checkpoint Inhibitors: Hype or Progress? Oncologist 2020; 25(2):94-98. doi: 10.1634/theoncologist.2019-0636.

31. Champiat Set al. Hyperprogressive disease: recognizing a novel pattern to improve patient management. Nat Rev Clin Oncol. 2018;15(12):748-762

32. Lo Russo G, et al. Antibody-Fc/FcR Interaction on Macrophages as a Mechanism for Hyperprogressive Disease in Non-small Cell Lung Cancer Subsequent to PD-1/PD-L1 Blockade Clin Cancer Res 2019; 25(3):989-999.

33. Ferrara R, et al. Hyperprogressive Disease in Patients With Advanced Non-Small Cell Lung Cancer Treated With PD-1/PD-L1 Inhibitors or With Single-Agent Chemotherapy JAMA Oncol 2018;4:1543–52.

34. Champiat S, et al. Hyperprogressive Disease Is a New Pattern of Progression in Cancer Patients Treated by Anti-PD-1/PD-L1. Clin Cancer Res 2017;23:1920–8.

35. Sun, Z. et al. Radiomics study for predicting the expression of PD-L1 in non-small cell lung cancer based on CT images and clinicopathologic features. J. X-Ray Sci. Technol. J Xray Sci Technol. 2020;28(3):449-459.

36. Polverari, G. et al. 18F-FDG Pet Parameters and Radiomics Features Analysis in Advanced Nsclc Treated with Immunotherapy as Predictors of Therapy Response and Survival. Cancers. 2020;12(5):1163.

37. Miller, T. Explanation in artificial intelligence: Insights from the social sciences. Artif. Intell. 2019;267:1–38.

36. Conforti, F. et al. Cancer immunotherapy efficacy and patients' sex: a systematic review and meta- analysis. Lancet Oncol. 2018;19:737–746.

37. Triberti, S., Durosini, I. & Pravettoni, G. A "Third Wheel" Effect in Health Decision Making Involving Artificial Entities: A Psychological Perspective. Front. Public Health 8, (2020)

38. Stacey D, Légaré F, Lewis K, Barry MJ, Bennett CL, Eden KB, et al. Decision aids for people facing health treatment or screening decisions. Cochrane Database Syst Rev. 2017;4:CD001431

39. Søndergaard, S. R., Madsen, P. H., Hilberg, O., Jensen, K. M., Olling, K., & Steffensen, K. D. (2019). A prospective cohort study of shared decision making in lung cancer diagnostics: impact of using a patient decision aid. Patient Education and Counseling, 102(11), 1961-1968.

40. Hoffman, A. S., Hempstead, A. P., Housten, A. J., Richards, V. F., Lowenstein, L. M., Leal, V. B., & Volk, R. J. (2018). Using a patient decision aid video to assess current and former smokers' values about the harms and benefits of lung cancer screening with low-dose computed tomography. MDM policy & practice, 3(1), 2381468318769886.

41. Shay LA, Lafata JE. Where is the evidence? A systematic review of shared decision making and patient outcomes. Med Decis Mak. 2014;35:114–31.

42. Anolli L, Ciceri R. La voce delle emozioni. FrancoAngeli, 2000. ISBN: 9788820499174.
