# Supplementary material for: Clinical usability of an explainable AI decision support tool and evaluation of multimodal models in NSCLC
Source: Nat Med. 2026 Sep 13;32(9):3235–47. doi: 10.1038/s41591-026-04488-2 (PMC13577906; doi:10.1038/s41591-026-04488-2)
Supplement: Supplementary file 2 [file 41591_2026_4488_MOESM2_ESM.pdf]

nature portfolio

Corresponding author(s): Arsela Prelaj

Last updated by author(s): May 19, 2026

# Reporting Summary

Nature Portfolio wishes to improve the reproducibility of the work that we publish. This form provides structure for consistency and transparency in reporting. For further information on Nature Portfolio policies, see our Editorial Policies and the Editorial Policy Checklist.

## Statistics

For all statistical analyses, confirm that the following items are present in the figure legend, table legend, main text, or Methods section.

| n/a | Confirmed | |
|---|---|---|
| ☐ | ☒ | The exact sample size (*n*) for each experimental group/condition, given as a discrete number and unit of measurement |
| ☐ | ☒ | A statement on whether measurements were taken from distinct samples or whether the same sample was measured repeatedly |
| ☐ | ☒ | The statistical test(s) used AND whether they are one- or two-sided<br>*Only common tests should be described solely by name; describe more complex techniques in the Methods section.* |
| ☐ | ☒ | A description of all covariates tested |
| ☐ | ☒ | A description of any assumptions or corrections, such as tests of normality and adjustment for multiple comparisons |
| ☐ | ☒ | A full description of the statistical parameters including central tendency (e.g. means) or other basic estimates (e.g. regression coefficient) AND variation (e.g. standard deviation) or associated estimates of uncertainty (e.g. confidence intervals) |
| ☐ | ☒ | For null hypothesis testing, the test statistic (e.g. *F*, *t*, *r*) with confidence intervals, effect sizes, degrees of freedom and *P* value noted<br>*Give P values as exact values whenever suitable.* |
| ☒ | ☐ | For Bayesian analysis, information on the choice of priors and Markov chain Monte Carlo settings |
| ☒ | ☐ | For hierarchical and complex designs, identification of the appropriate level for tests and full reporting of outcomes |
| ☐ | ☒ | Estimates of effect sizes (e.g. Cohen's *d*, Pearson's *r*), indicating how they were calculated |

*Our web collection on statistics for biologists contains articles on many of the points above.*

## Software and code

Policy information about availability of computer code

| | |
|---|---|
| Data collection | Clinical, laboratory, and genomic data were collected through electronic Case Report Forms (eCRFs) within the centralized I3LUNG platform. Computed tomography (CT) scans in DICOM format and hematoxylin and eosin (H&E) whole-slide images in SVS format were uploaded and managed through the same platform. The I3LUNG platform is hosted on Amazon Web Services (AWS) infrastructure and was developed according to data protection by design and by default cybersecurity principles. The hosting AWS servers comply with the ISO/IEC 27001:2022 standard. AWS incorporates its Data Processing Addendum into the AWS Service Terms (publicly available at https://aws.amazon.com/service-terms/). |
| Data analysis | Data analyses were performed using a combination of commercial and open-source software tools, including SAS software version 9.4 (SAS Institute), R (version 4.2.1.), Python (version 3.11.15 for MLEF and version 3.9.25 for DLIF), and 3D Slicer (version 5.2.2). Custom scripts developed for data preprocessing, and model development are publicly available on GitHub (https://github.com/AI-ON-Laboratory/I3LUNG_PDSS). |

For manuscripts utilizing custom algorithms or software that are central to the research but not yet described in published literature, software must be made available to editors and reviewers. We strongly encourage code deposition in a community repository (e.g. GitHub). See the Nature Portfolio guidelines for submitting code & software for further information.

# Data

Policy information about availability of data

All manuscripts must include a data availability statement. This statement should provide the following information, where applicable:

- Accession codes, unique identifiers, or web links for publicly available datasets
- A description of any restrictions on data availability
- For clinical datasets or third party data, please ensure that the statement adheres to our policy

De-identified data supporting the findings of this study are publicly available on Zenodo (https://zenodo.org/records/17535424) and will remain accessible for 20 years. The shared dataset includes clinical and blood (CB) variables, genomics data, and extracted features derived from medical imaging analyses, including radiomics and foundation model–based features from CT scans and digital pathology images (GigaPath and TITAN). These are necessary data to replicate the study. During the peer-review process, reviewers were provided with access to these data necessary to reproduce the study analyses.

The I3LUNG project is an ongoing EU-funded initiative, with additional analyses and related manuscripts planned through June 2027. Therefore, the complete raw multimodal dataset, including original imaging files, cannot currently be fully released. In accordance with the consortium's data-sharing policy and applicable European Commission regulations governing data access, sharing, and open science within EU-funded projects, the full raw datasets from all modalities will become available five years after the official completion of the project.

# Research involving human participants, their data, or biological material

Policy information about studies with human participants or human data. See also policy information about sex, gender (identity/presentation), and sexual orientation and race, ethnicity and racism.

| | |
|---|---|
| Reporting on sex and gender | Assigned sex is reported for all individuals included in the study. Sex data, together with other variables required to reproduce the analyses (including self-reported race), are publicly available on Zenodo (https://zenodo.org/records/17535424). During the peer-review process, these data were also accessible to the Editors and Reviewers. |
| Reporting on race, ethnicity, or other socially relevant groupings | Self-reported race for every individual where the data was available are reported and publicly available on Zenodo (https://zenodo.org/records/17535424). |
| Population characteristics | For I3LUNG cohorts population eligibility criteria included adults aged 18 years or older, and a histologically confirmed diagnosis of stage IIIC-IVB NSCLC according to the 8th edition of the TNM staging system. Patients were treated with first and further line (IO-based therapy).<br><br>Non-I3LUNG cohorts included only adult patients (≥18 years). EGFR cohorts: EGFR-mutant tsage IIIC-IVB NSCLC patients treated with EGFR-TKIs,  Chemotherapy cohort: Stage III NSCLC patients treated with neoadjuvant chemotherapy followed by surgery. |
| Recruitment | Participants were enrolled in the I3LUNG multicenter observational study (NCT05537922). This analysis includes adults (≥18 years) with stage III–IV NSCLC treated with immune checkpoint inhibitors, with or without chemotherapy, in routine clinical practice and identified from medical records according to protocol-defined eligibility criteria.<br><br>Potential sources of bias include selection bias due to the retrospective inclusion of patients with sufficient clinical and follow up data, center/referral bias related to specialized oncology centers, and heterogeneity arising from incomplete multimodal data availability across patients and sites, which may limit the generalizability of the models to broader NSCLC populations and to settings with different resource availability. |
| Ethics oversight | The study was conducted in accordance with the Declaration of Helsinki and Good Clinical Practice. Ethical approval was obtained from independent ethics committees at each participating site.<br><br>The full names of the approving committees are:<br>• Comitato Etico Fondazione IRCCS Istituto Nazionale dei Tumori di Milano (INT), Ethics Committee Code 147/22, approved on 25 July 2022.<br>• Ethik-Kommission Universität zu Lübeck (GHD), Ethics Committee Code 2022-439, approved on 17 October 2022.<br>• Scientific Committee Metropolitan Hospital (MH), Ethics Committee Code 30.08.2022, approved on 30 August 2022.<br>• Helsinki Committee Shaare Zedek Medical Center (SZMC), Ethics Committee Code 0240-22-SZMC, approved on 20 September 2022.<br>• Comité de Ética de Investigación con Medicamentos y Comisión de Proyectos de Investigación del Hospital Universitari Vall d'Hebron, approved on 17 January 2023.<br>• Institutional Review Board University of Chicago (UoC), Ethics Committee Code IRB22-1305, approved on 8 September 2022.<br><br>Written informed consent was obtained for all alive patients. For the retrospective cohort, when consent could not be obtained (e.g., deceased patients), data processing was performed in accordance with applicable data protection regulations, including GDPR provisions for scientific research, and local ethics approvals.<br><br>For Non I3LUNG Cohorts:<br>Cohort-EGFR-INT: these data have been collected within the APOLLO 11 study, approved by the ethical committee of INT |

(reference nr. Of the protocol) INT 128/22 by 29th June 2022.
Cohort-EGFR-UOC: Institutional Review Board University of Chicago (UoC), Ethic Committee code IRB24-2188, Approved on the date 23rd December 2024
Cohort-StageIII-CHT: For this cohort collected in INT the data protection impact assessment has been developed and is published at INT website https://dpm.istitutotumori.mi.it/public/documents/ called: Informativa-I3Lung Monocentrico, in compliance with applicable personal data protection laws, including Regulation (EU) 2016/679 (GDPR) and relevant national legislation. A distinct and appropriate legal basis was identified for each category of personal data processed, tailored to the nature and purpose of each specific processing activity. Suitable technical and organizational measures were put in place to ensure an adequate level of data security, in line with the principles of data minimization, purpose limitation, and access control, ensuring that only authorized individuals were granted access to personal information. The research was carried out in accordance with applicable ethical standards and under the supervision of the relevant institutional bodies. Throughout the entire research process, the accountability principle was upheld, meaning that the data controller was not only committed to complying with data protection requirements, but was also in a position to demonstrate such compliance at any time.

Note that full information on the approval of the study protocol must also be provided in the manuscript.

# Field-specific reporting

Please select the one below that is the best fit for your research. If you are not sure, read the appropriate sections before making your selection.

☒ Life sciences ☐ Behavioural & social sciences ☐ Ecological, evolutionary & environmental sciences

For a reference copy of the document with all sections, see nature.com/documents/nr-reporting-summary-flat.pdf

# Life sciences study design

All studies must disclose on these points even when the disclosure is negative.

| | |
|---|---|
| Sample size | No prior sample size calculation was performed. The sample sizes of final cohorts were chosen based on data availability, and inclusion criteria. |
| Data exclusions | All patients meeting the inclusion criteria were included in the study. Patients initially enrolled but who did not receive at least one cycle of immunotherapy, or who had missing endpoint data, were excluded from the analyses. |
| Replication | The primary analyses were replicated across leave-one-center-out cross-validation folds, an internal test set, and an external validation cohort (UOC). Model performance was additionally evaluated across multiple clinically relevant subgroups (details in Supplementary Information 2). Non-immunotherapy cohorts were further used to assess prognostic versus predictive effects. Replication of the main analyses is possible using the publicly available data deposited on Zenodo (https://zenodo.org/records/17535424) and the analysis code available on GitHub (https://github.com/AI-ON-Laboratory/I3LUNG_PDSS). Replication of analyses involving non-IO cohorts is not possible, as these datasets are not publicly available due to other related ongoing publications. |
| Randomization | Model development: Patients from UoC where used as external validation set, patients from remaining 5 centers were splitted into train (85%) and test (15%), randomly, but stratified by center and death event.<br><br>Clinical usability: 80 patients from the internal TEST set and 20 patients from the external validation (EXVAL) cohort were selected and assigned to 20 clinicians (10 experts and 10 non-experts). Patients from the TEST set were randomly assigned to clinicians from European centers, whereas patients from the EXVAL cohort were randomly assigned to clinicians from the USA. |
| Blinding | Blinding was used in the clinical usability study. During Phase 1, physicians were blinded to the XAI model predictions and explanations. This allowed unbiased baseline assessment before being exposed to the XAI-assisted outputs in Phase 2; No blinding was used during model development, as data labeling was based on existing clinical outcomes. |

# Reporting for specific materials, systems and methods

We require information from authors about some types of materials, experimental systems and methods used in many studies. Here, indicate whether each material, system or method listed is relevant to your study. If you are not sure if a list item applies to your research, read the appropriate section before selecting a response.

## Materials & experimental systems

| n/a | Involved in the study |
|---|---|
| ☒ | ☐ Antibodies |
| ☒ | ☐ Eukaryotic cell lines |
| ☒ | ☐ Palaeontology and archaeology |
| ☒ | ☐ Animals and other organisms |
| ☐ | ☒ Clinical data |
| ☒ | ☐ Dual use research of concern |
| ☒ | ☐ Plants |

## Methods

| n/a | Involved in the study |
|---|---|
| ☒ | ☐ ChIP-seq |
| ☒ | ☐ Flow cytometry |
| ☒ | ☐ MRI-based neuroimaging |

# Clinical data

Policy information about clinical studies

All manuscripts should comply with the ICMJE guidelines for publication of clinical research and a completed CONSORT checklist must be included with all submissions.

**Clinical trial registration**

NCT05537922

**Study protocol**

I3LUNG study protocol is submitted with the manuscript

**Data collection**

Clinical data were collected across six international oncology centers participating in the I3LUNG study:
- Istituto Nazionale dei Tumori (Italy)
- LungenClinic Grosshansdorf (Germany)
- Metropolitan Hospital (Greece)
- Shaare Zedek Medical Center (Israel)
- Vall d'Hebron Institute of Oncology (Spain)
- University of Chicago (USA)

The retrospective dataset includes patients treated with immunotherapy between September 2012 and October 2023, with data collected from routine clinical practice and recorded in standardized electronic case report forms within the I3LUNG. Data collection followed protocol-defined procedures, including predefined minimum datasets (e.g., treatment details, response, and follow-up), and centralized data curation and quality control processes across sites.

**Outcomes**

Outcomes were pre-defined in the study protocol prior to analysis. See study protocol.
Clinical endpoints included both response and survival outcomes. For the present analysis (retrospective cohorts) primary endpoints included overall survival (OS), OS at predefined landmark time points (for example, OS at 6 and 24 months), and disease control rate (DCR). Secondary endpoints included objective response rate (ORR) and clinical benefit rate (CBR)
Tumor response outcomes were assessed according to RECIST version 1.1, based on radiological evaluations performed in routine practice at each site. DCR was defined as stable disease (SD), partial response (PR), or complete response (CR) versus progressive disease (PD). ORR was defined as PR or CR. CBR incorporated both tumor response and a minimum progression free survival threshold, as pre specified in the protocol.
Survival outcomes were defined as follows: OS, measured from initiation of immunotherapy (with or without chemotherapy) to death from any cause or last follow up; and progression free survival (PFS), measured from treatment initiation to documented disease progression or death, whichever occurred first. For landmark analyses (OS6 and OS24), only patients with at least 6 or 24 months of potential follow up, respectively, were included, and those alive and uncensored at the landmark time were classified according to their survival status at that time point.

# Plants

**Seed stocks**

*Report on the source of all seed stocks or other plant material used. If applicable, state the seed stock centre and catalogue number. If plant specimens were collected from the field, describe the collection location, date and sampling procedures.*

**Novel plant genotypes**

*Describe the methods by which all novel plant genotypes were produced. This includes those generated by transgenic approaches, gene editing, chemical/radiation-based mutagenesis and hybridization. For transgenic lines, describe the transformation method, the number of independent lines analyzed and the generation upon which experiments were performed. For gene-edited lines, describe the editor used, the endogenous sequence targeted for editing, the targeting guide RNA sequence (if applicable) and how the editor was applied.*

**Authentication**

*Describe any authentication procedures for each seed stock used or novel genotype generated. Describe any experiments used to assess the effect of a mutation and, where applicable, how potential secondary effects (e.g. second site T-DNA insertions, mosiacism, off-target gene editing) were examined.*
