# Supplementary material for: Clinical usability of an explainable AI decision support tool and evaluation of multimodal models in NSCLC
Source: Nat Med. 2026 Sep 13;32(9):3235–47. doi: 10.1038/s41591-026-04488-2 (PMC13577906; doi:10.1038/s41591-026-04488-2)
Supplement: Supplementary file 3 [file 41591_2026_4488_MOESM3_ESM.pdf]

nature portfolio

Peer Review File

Clinical Usability of an Explainable AI Decision Support Tool and Evaluation of Multimodal Models in NSCLC

Corresponding Author: Dr Arsela Prelaj

This file contains all reviewer reports in order by version, followed by all author rebuttals in order by version.

Version 0:

Reviewer comments:

Reviewer #1

(Remarks to the Author)
In this manuscript NMED-A147165 entitled "I3LUNG: Clinical Validation of a Multimodal AI Tool to Support Immunotherapy Decisions in NSCLC", Dr. Prelaj and colleagues present the results of a robust international, real word and multimodal-based translational effort that integrates clinical, radiological, genomic, and pathology data from more than 2000 patients into AI/machine learning models in order to generate a tool that can aid the clinical decision making process and guide patient selection for effective immune-based therapy. The authors demonstrate that machine-learning approach performs consistently across outcomes and achieves improved results in FL setting, whereas multimodal models outperformed real world data in clinically relevant subgroups and AI models were superior to other prognostic and predictive established or emerging factors/biomarkers. These results provide a potential platform for clinical applicability as it appears providers could achieve, once these results are prospectically validated, improved prediction with the AI-based tool.

The authors must be commended for this high-risk, herculean undertaking that integrates a non-trivial amount of cross-institutional and multidisciplinary patient and disease information to provide the community with a sophisticated and less subjective tool that can ultimately increase the benefit of immunotherapy in the most appropriate population of patients. These aspects bring to the findings substantial novelty, impact and generalizability for clinical utility. The need for prospective validation is clear and emphasized by the authors and it is indeed ongoing in large cohorts. I have a few comments for the authors that could further strengthen the value of this work for the clinical and translational community, as discussed below:

Major issues:
1. The authors clearly describe that the current analyses are focused on C2 and C3. Although I could appreciate why a C3 including stage 3 pts where IO therapy as consolidation has a curative intent represents a cohort with a prognostically different population, I feel there is a missed opportunity here to look at the role of an AI-based multimodal approaches specifically in the curative-intent disease setting.
2. The authors should provide the results of analyses in the clinically relevant subgroups PD-L1<1%, 1-49% and ≥50%, in addition to the analysis with binary subgroups <50% or ≥50%.
3. I may have missed it, I do not see the min, max and median follow up time for survival analyses. Please provide, or bring to my attention, in text and figure legends.
4. Throughout the manuscript, it is complex and at times fatigue-inducing to follow the text depicting the corresponding panels in each figure because the text does not follow in the same order of each panel (for example, text describes Fig 1c, then 1d then 1b etc., similarly for Fig 2). Please consider reorganizing the text or, perhaps, would be more appropriate to reorganize the order of panels in the figures so that the order is aligned to the text describing each panel.
5. Table 1: I applaud the authors for a detailed amount of well-organized info. Please change the current header D" Disease control rate, n (%). What is shown is not the disease control rate but the RECIST categories. DCR is the proportion of patients with CR, PR and SD. Please rectify.
6. Table 1: Please consider including molecular/driver (EGFR/ALK/RET/ROS1) subsets and also include KRAS, STK11 and KEAP1 subgroups and co-mutation subgroups. as these are available from genomic data. Please clearly describe how was PFS defined, as done for OS.
7. Please provide performance of the multimodal approaches in molecularly defined subgroups, namely KRAS, STK11, KEAP1 mutant and in the co-mutant subgroups.
8. The Discussion is overall thoughtful and well-written. The authors discuss their findings in the context of what's known in

the literature and detail some of the limitations that are inherent to the nature of such study.
a. Can the authors expand in the Discussion on how they or the field should practically minimize the impact of heterogenous data quality from diverse centers for calibration and validation at each site before this tool can be deployed for clinical use?
b. It would be helpful to the lung cancer community if the authors could provide their thoughts and interpretation of the practical impact and utility of their results and the AI tool for daily clinical decisions by providers, implementing an approach in their description that is truly centered on clinically-relevant NSCLC subgroups in the advanced/metastatic setting.

Minor issues:
1. In the abstract the number of patients enrolled in this effort is reported to be 2365, but everywhere else (text and figures) I seem to depict 2396, what is the difference between these numbers?
2. Consider adding a description of clinical endpoints in Fig 1a.
3. I would suggest considering bringing the schema in Supp Fig 1 to Main or at least to Extended Data to provide the overview of cohorts utilized and undergoing validation.
4. In the Results text, “DCR responders” is not entirely accurate as R indicates a rate as proportion of patients with overall disease control so PR, CR and SD. For clarity, consider changing to simply “responders” (defined throughout including the methods to include also patients with SD) vs “non-responders” and remove DCR in front of responders, as you have basically done in Methods-Clinical Endpoints.
5. Consider changing the title of paragraph reporting on survival analyses findings. In its current form, “Survival analysis achieved moderate results”, this title is unclear and does not convey what the key results are in a nutshell, as instead the other subsection/paragraph titles attempt to do.

(Remarks on code availability)

Reviewer #2

(Remarks to the Author)
This study addresses a highly relevant clinical question, how to improve individualized immunotherapy selection for advanced NSCLC. A major strength of the work is the assembly of one of the largest real-world, international, multicenter cohorts integrating clinical data, CT imaging, H&E digital pathology, and genomics. The scale of the dataset is impressive and position this work as a potentially important contribution to the field. The comments below are intended to help strengthen the methodological rigor and the clinical interpretability of the findings.

Please consider the follwoing comments:

1. There is substantial heterogeneity in performance across centers and subgroups, which raises concerns about model stability. For example, in the fairness/center analysis of the RWD-only MLEF classifier, the TPR for OS6 is 0.96 in MH versus 0.58 (INT), 0.62 (GHD), 0.62 (VHIO), 0.61 (SZMC). For DCR, MH again shows TPR=0.96 versus 0.63 (INT) ($p \leq 0.05$). This magnitude of variability suggests that performance is strongly center-dependent and may reflect differences in practice patterns, data completeness or follow-up rather than robust generalization. Please provide subgroup sample sizes, calibration by center and an analysis of potential drivers of heterogeneity.

2. The use of a geographically distinct external validation (EXVAL) cohort is appropriate in principle. However, the EXVAL cohort differs substantially from the development cohorts in baseline characteristics and outcomes with a markedly longer median OS (22.2 months vs 12.5 months). This raise concerns that the EXVAL cohort represents a different-risk population rather than a true test of model generalitaton. Differences in line of therapy, histology type, sex and treatment composition (IO vs IO/chemo) may contribute to this survival gap. As a result, reduced performance in EXVAL for key clinical endpoints is difficult to interpret as either model failure or population shift. I recommend tempering claims of robust generalization.

3. Most of the reported gains from multimodal integration are derived from cross-validation rather than locked TEST or EXVAL analyses. While understandable given sample size constraints, this limits the strength of the conclusions, as cross-validation remains an internal validation strategy and may be influenced by center-specific effects or overfitting effect. I suggest reframing cross-validation findings as exploratory and prioritizing locked TEST and EXVAL results when making claims about clinical performance and multimodal benefit.

4. The MLEF approach excludes patients with missing modalities, resulting in potential complete-case bias. In real-world oncology practice, incomplete multimodal data are very common, making this issue central to clinical applicability. While DLIF is designed to address missing modalities, it does not consistently demonstrate improved performance with additional modalities, creating ambiguity about the practical value of multimodal integration.

Although the DLIF framework is designed to accommodate missing modalities, it does not consistently show improved performance with additional data, creating some ambiguity regarding the practical value of multimodal integration. This analysis would be strengthened by a clearer characterization of missingness (by modality, center, outcome) and by exploring strategies beyond complete-case analysis, including missingness indicators, multiple imputation and architectures explicitly designed for missing modalities.

It would be also helpful to clearly state which modeling paradigm (MLEF vs DLIF) is envisioned for real-world deployment and why.

5. The models are trained on patients receiving IO or IO/chemotherapy, which limits the ability to disentangle treatment-specific predictive value from general prognosis. From a clinical decision-making standpoint, prediction of benefit from immunotherapy relative to alternative treatments is far more impactful than prognosis alone. I would encourage inclusion of comparator cohorts treated with chemotherapy alone and/or EGFR-TKIs to assess treatment specificity.

6. Please provide clearer guidance on how the final model and decision thresholds were selected and fixed prior to evaluation in the EXVAL cohort.

7. Although the overall cohort is very large (2396 patients with clinical data), the effective sample size for multimodal analyses is substantially smaller and imbalanced. While CT scans are available for 1705/2396 patients (71%) in the full collected population, in the actual study population used for model development (C2-C3, n=2075), CT availability drops to 896/2075 patients (43%). The overlap across modalities is even more limited, 339 patients having all four modalities (clinical, CT, pathology, genomics). This is important as it may indicate bias selection of patients from specific centers with more complete diagnostic workflows and may correlate with prognosis, treatment patterns and follow-up intensity. As a result, observed multimodal performance may partially reflect complete-case selection bias rather than true incremental predictive value from additional modalities. To address this limitation, the study could benefit from: a systematic analysis of missingness by center, treatment and outcome; comparison of baseline characteristics and overall survival between multimodal-complete and modality-incomplete patients; and sensitivity analyses using approaches that can leverage partial modality availability, rather than restricting analyses to complete cases.

Minor comments:
- Font sizes in several main and supplementary figures are too small to be legible. This significantly difficult interpretability and should be corrected.
- Clarify censoring and label definition for OS6 and OS24 to avoid potential follow-up bias.
- The paper states baseline clinical features are collected before the first IO cycle. However, the acceptable timing window for CT/H&E relative to IO initiation is not clearly quantified in the main manuscript. Given disease dynamics, longer intervals could materially affect both radiomics and pathomics. Please report the distribution of scan/biopsy-to-treatment intervals.
- “RWD” vs “clinical data” is confusing. CT scans, H&E slides, and targeted genomics are also routine real-world clinical data. Using “RWD” to mean only structured clinical variables may mislead readers about what is “real-world” versus “research-grade.” Consider re-labelling as “clinical variables” (or similar) and describing imaging/pathology/genomics as routine clinical data modalities.
- The authors note that “contrast-enhanced scans were preferred when available,” and that contrast information varies by center. Because intra venous contrast status can strongly influence PyRadiomics features (and potentially FM embeddings), please report the proportion of contrast vs non-contrast scans by center and test robustness.
- DCR is defined with SD + PR + CR as “responders” vs PD as “non-responders”. Stable disease is heterogeneous, and “best response = SD” does not necessarily imply meaningful benefit, especially without duration. Consider reporting ORR (CR/PR) separately and/or a clinical benefit endpoint (e.g., CR/PR or durable SD ≥6 months), which is more decision relevant.

(Remarks on code availability)
A detailed review of the code would be time-consuming. I would be happy to review the code if the manuscript progresses further, provided that the key methodological concerns raised above are addressed.

Reviewer #3

(Remarks to the Author)
General comments

An elegantly-conceived, -conducted, and -reported observational study designed to develop and evaluate multivariable models for prediction of objective tumour control (disease control response = CR, PR, or SD), and for prediction of survival beyond 2 years, following treatment with an immune checkpoint inhibitor.

The work is highly original and innovative, particularly in its depth, breadth, rigour, and the sophistication of both development and evaluation.

The data, approaches, and methods are rigorous, well-described, and appropriate.

Statistical methods are sound, current, and appropriate.

The conclusions are suitably tempered and supported by the reported data.

I enjoyed reading it, and learnt much about the new methods, but it was hard work given the density and complexity of analyses and results.

The study raises many intersting unanswered questions about prognostication, decision-making, and doctor-patient communication.

The suggestions below are intended to help improve readability for Nature Medicine’s readership.

Specific Suggestions
1. Figure 1a bottom left panel, and elsewhere: please use appropriate numbers of icons (human symbols) to reflect the relative sizes of TRAIN vs TEST vs EXVAL, i.e. TRAIN ~ 6 x TEST and EXVAL. Current figures imply (exaggerate) that TEST and EXVAL are half the size of TRAIN.
2. Lines 173-181, 186-192, and 193-200: please include actual p-values (i.e. p = X), rather than ‘significance levels’ (i.e. p ≤ X).
3. Comparisons of multivariable models versus single biomarkers in isolation are not very persuasive. Would anyone recommend making decisions about therapy based solely on LDH, or NLR? PS is useful for identifying individuals unlikely to benefit from treatment, but not for selecting one treatment over another.
4. In many places, the abbreviation for confidence intervals is reversed (IC instead of CI).
5. Figure 4 c includes many interesting and innovative visualisations of unfamiliar indices, e.g. SHAP values, diagnostic test performance statistics. These need better explanation for the intended readership.
6. Line 318, please explain prediction of ‘DCR’ more clearly in the manuscript methods and results. The sentence is confusing, because it is easy to mistake that the categories for prediction of DCR were intervals of time in months. It took much backing and forthing, before I realised from the Figure 5a that the question was ‘DCR yes v no’. The R in DCR is defined as ‘rate’ earlier in the text. It would be clearer to use the abbreviation DCR for disease control Response (CR, PR, or SD). This needs to be clearer and used consistently throughout the manuscript.
7. Lines 323-326. The stated results indicate that predictions of non-experts were substantially better than those of experts (AUC 0.77 vs 0.68 without XAI, 0.90 vs 0.83 with XAI.) Is this correct? This warrants discussion. It raises questions about the design, phrasing, and interpretation of both the questions and the answers.
8. Given the differences between ‘experts’ and ‘non-expert’, it would be helpful for the definitions and/or characteristics of these 2 groups to be explained in the body of the manuscript, e.g. lung vs non-lung expert oncologists, as explained in 705-710, rather than expert vs non-expert.
9. Line 326. Clarify what is meant by a “37% increase in the likelihood” by defining and including the two numbers being compared.
10. Line 327 (p=0.1) is sufficient, no need to report p to 4 significant figures.
11. The improvement in line 326 is overemphasised given the p-value in line 327 indicates well within the play of chance.
12. The survival predictions are interesting, but again, somewhat naïve. It is unrealistic to expect a point-estimate of the predicted survival time. Even when perfectly calibrated, one would expect no more than a third of actual survival times to be within +/- one third of the expected survival time. A probabilistic prediction would have been more appropriate, e.g. probability of surviving more than x months, or estimated median survival time (in a group of similar individuals).
13. Sorry if I’ve missed, but what are the ‘correctness’ scores for the AI predictions of survival? Based on lines 718-19, I’m guessing that the survival time interval including the 50th percentile of the OS probability curve for each patient generated by AI would most closely correspond with the OS ‘prediction’ clinicians completed.
14. Line 442, please spell out and explain ‘NLP’
15. Lines 450-452. None of these factors, other than presence of targetable, oncogenic driver mutations, were ever ‘sufficient to capture the complexity of’ response to treatment.
16. Line 462, clarify in the text what the “>200” in parentheses refers to (participants?).
17. Line 595, KRAS mutations not counted as actionable, as appropriate from 2012-23. Should be mentioned in limitations, along with advent of new targets and treatments.
18. Please include in the body of the manuscript a sentence that specifies the 9 RWD predictive features selected (mentioned in lines 510 and 610).
19. Line 609, please clarify in the manuscript what is meant by “starting from 227”.
20. Please clarify in the manuscript whether you considered/included age, comorbidity measures, or frailty measures. And discuss these as either strengths or limitations.
21. Line 726, I think it should be “Similarly, two…”, not “Similar two…”
22. Line 726, prediction within 25% of actual is an unusually narrow margin for success; 33% is more commonly used. Prediction within 0.75 to 1.33 times actual would be even better.

(Remarks on code availability)

Reviewer #4

(Remarks to the Author)
1. Summary of the Key Results
This manuscript reports on the retrospective validation of the I3LUNG AI decision support system using a large cohort (n=2,396) of NSCLC patients across six international centers. The study compares two architectures, i.e., Machine Learning Early Fusion (MLEF) and Deep Learning Intermediate Fusion (DLIF), to integrate RWD, radiomics, pathology, and genomics. Notably, the MLEF models (AUC ~0.77) outperformed standard biomarkers. A key, albeit counter-intuitive, finding is that while multimodality helped in specific subgroups, models using RWD alone performed comparably to complex multimodal models in the general cohort. The inclusion of a usability study showing increased sensitivity for physicians (0.72 to 0.87) is a significant addition.
2. Originality and Significance
This work presents a large-scale, international multi-center study encompassing 2,396 patients from six countries, which shows undoubtable superiority against similar research. by highlighting the advantage of leveraging routine real-world data over rarer metrics like TMB. While the core concept of multimodal immuno-oncology prediction is not novel, the study

demonstrates clear novelty in its execution, scale, and translational focus. This is evidenced not only by its detailed comparative analysis of multiple ML/DL models but, more importantly, by the inclusion of a “Clinical Usability” study assessing human-AI interaction. This translational component, combined with the substantial dataset, elevates the work above many purely computational pathology papers and strongly justifies its high potential impact.
3. Data & Methodology
- 3.1 Validity of the Approach: The framework is generally sound, but I have reservations about the composite "driver" feature. Lumping EGFR, ALK, RET, and ROS1 into a single binary variable is methodologically risky. Biologically, EGFR often predicts resistance to IO, whereas other drivers may behave differently. Collapsing them risks washing out the signal. A sensitivity analysis separating EGFR is necessary. Regarding the clinical usability study, a key methodological detail requires clarification. The oncologists were provided with both global and local SHAP explanations. However, given that the underlying features range from clinically interpretable variables to abstract representations from foundation models, it is crucial to specify: (1) how these two distinct types of explanatory information were presented to clinicians (e.g., in an integrated or separate manner), and (2) how the meaning of the more abstract features was explained to ensure they were correctly interpreted.
- 3.2 Quality of the Data: The explicit handling of real-world heterogeneity is a strength. However, the sample size for full multimodal integration is relatively small (n=339). This makes it difficult to accept the conclusion that the DLIF approach failed to outperform MLEF; it is equally likely that the deep learning model simply lacked sufficient data to converge.
- 3.3 Quality of Presentation:
3.3.1. Quality and Consistency of Figures and Visualizations
The current presentation of figures significantly hinders the comprehension of the complex methodology and results.
(1) Please update the figures to vector graphics; currently, the text and numbers are pixelated and difficult to read.
(2) Inconsistent Notation and Ambiguous Labels:
1) The matrices representing the modalities in Figure 1a are confusing regarding their dimensions and shapes—these need to be labeled more clearly. I also noticed a notation discrepancy in Figure 3a. The schematic on the left labels the encoder input/output dimensions as d_i and d_z (and similarly for the MIL fusion), but the detailed component diagrams on the right use different labels for these same dimensions.
2) Figure 2f: The caption contains an error. "Each row represents one patient" should be corrected to "Each column represents one patient."
(3) The nomenclature for the four core data modalities is inconsistent across figures, creating unnecessary confusion. Figure 1a uses: Clinical, Radiomics, Pathomics, Genomics. Figure 1b uses: RW Data, Digital Pathology, Radiomics, Genomics. Figure 3a uses: Clinical, Digital Pathology, Radiology, Genomics. Please review and rigorously unify the terminology for these four input types across all figures and their captions in the main text.
(4) Optimization of Visual Encoding in Results Figures: Figures 2g & 3c: The current dual encoding (circle size and color hue) for representing sample size and model performance, respectively, is suboptimal for rapid interpretation. Suggestion:
1) Directly annotate the sample size (N) next to the model name on the y-axis (e.g., RWD-only (N=xxx)).
2) Use a single, consistent circle size for all data points.
3) Encode performance solely via a color gradient. Since no AUC values are below 0.5 in these plots, set the color map range from 0.5 to 1.0. This will enhance contrast and make performance differences across models and datasets more visually apparent.
3.3.2. Presentation and Interpretation of Results
(1) Simplification of Subgroup Analyses: the subgroup performance summaries are dense. Please consider simplifying these to highlight the most clinically actionable findings.
(2) Missing Baseline Analysis and Potential Model Bias:
1) Demographic Characteristics: Table 1 (or a supplementary table) must report the distribution of key demographic variables (e.g., sex, age) across the training, test, and external validation sets, as well as across the cohorts defined by different prediction tasks (OS24, OS6, DCR). A statistical comparison for differences is essential to assess dataset comparability and potential sampling bias.
2) Analysis of Subgroup Performance & Fairness: Figure 4a-b suggests potential biases that require discussion and clarification.
The notable variation in DLIF performance across centers and the substantial difference in TPR (>10%) between male and female subgroups for MLEF are concerning for model generalizability and fairness.
Please discuss these observations in the context of the model's potential limitations.
Crucially, the methods section should state whether and how mitigation strategies were employed to address potential biases related to sex or center effects during model development. If not, this should be acknowledged as a limitation.
3.3.3. Use and Definition of Abbreviations
There are also too much abbreviations used in the paper and some abbreviations are unclear and might cause trouble for reading, particularly non-intuitive ones like 'G' for genomics (provides no information actually). It would be helpful if the authors could: 1) improve their usage of abbreviations (and probably reduce the usage of unnecessary ones), 2) ensure that all abbreviations are clearly defined / referred upon first use.

4. Appropriate Use of Statistics and Treatment of Uncertainties
- External Validation Drop: The model performance drops in the external validation cohort (EXVAL, UOC-USA) compared to the test set (e.g., DCR AUC 0.71 in TEST vs 0.55 in EXVAL). The manuscript attributes this to "population shifts" (e.g., longer survival in UOC). This explanation is insufficient. The authors must perform a rigorous error analysis. Is the drop due to specific missing features in the US cohort, differences in CT acquisition, or biological heterogeneity?
- Fairness Audit: The inclusion of fairness metrics is commendable. However, significant variability in sensitivity was observed across centers (e.g., MH vs. INT). This center bias needs to be addressed before claiming the tool is ready for global deployment.
5. Conclusions: Robustness and Validity

The abstract claims multimodal models outperformed RWD in clinical-specific subgroups. While statistically true, the bigger story here seems to be the robustness of the RWD-only models. Highlighting the efficacy of RWD-only models could actually strengthen the paper's appeal for low-resource settings. Furthermore, the claim that this framework mitigates overfitting requires more evidence, especially given the degradation seen in the external validation cohort.
6. Reference, Clarity and Context
The abstract and introduction are lucid and provide excellent context. The distinction between MLEF (Early Fusion) and DLIF (Intermediate Fusion) is clearly articulated in the methods.

(Remarks on code availability)

Version 1:

Reviewer comments:

Reviewer #1

(Remarks to the Author)
The authors have appropriately addressed my comments and incorporated my suggestions accordingly in the revised manuscript which is clearly improved in strength and quality after thoughtful input from all referees. I have no further comments.

(Remarks on code availability)

Reviewer #2

(Remarks to the Author)
Thank you for your detailed and thoughtful responses, which have clearly improved the clarity and transparency of the manuscript. I particularly appreciate the additional analyses and the effort to better contextualize the findings.

As a remaining point, the performance decrease observed between TEST and EXVAL (OS24 AUC 0.74 vs 0.60; DCR 0.71 vs 0.55) is important and would benefit from a slightly more explicit discussion in the main text, particularly in terms of implications for model robustness across settings.

It may be helpful to more explicitly articulate the current stage of clinical applicability of the model, particularly how the outputs could be integrated into clinical decision-making.

(Remarks on code availability)

Reviewer #3

(Remarks to the Author)
Comments and suggestions addressed.
Methods and results reasonable.
Still hard work to wade through.
Claims exceed findings, comparators are poor, title is hybperbolic: not validated until evaluated in separate datasets.

(Remarks on code availability)

Reviewer #4

(Remarks to the Author)
This manuscript describes the retrospective arm of the I3LUNG project, an international real-world effort to develop AI models for immunotherapy decision support in advanced NSCLC using clinical data, CT, digital pathology and genomics. The study addresses an important clinical problem and benefits from a relatively large multicenter dataset. However, the manuscript currently overstates the level of validation and clinical readiness achieved. The strongest and most consistent results appear to come from the RWD-only models, whereas the multimodal advantage is limited, inconsistent, and largely confined to selected complete-case analyses. As written, the title, abstract and discussion go beyond what the data convincingly support.

Major:
1. Overstatement of validation level: The manuscript is framed as a “clinical validation” of a clinically relevant decision-support tool. In reality, this is a retrospective model-development and validation study with an exploratory usability component, while prospective validation remains ongoing. The title, abstract and discussion should be aligned more closely with the actual level of evidence.

2. The study is primarily prognostic rather than truly treatment-predictive: Although the manuscript is framed as supporting immunotherapy decision-making, the current analyses are conducted within IO/IO-CHT treated cohorts and mainly identify patients with better or worse outcomes. This is more consistent with prognostic modeling than with treatment-specific predictive modeling. Without an appropriate comparative treatment framework, it remains unclear whether the model identifies patients who specifically benefit from immunotherapy, rather than those with generally better prognosis.

3. Lack of consistent benefit from multimodal integration: A central claim of the manuscript is that multimodal integration improves prediction of immunotherapy outcomes. However, the results do not show a consistent or robust advantage over RWD-only models. Performance gains appear to be selective, endpoint-dependent, and often limited to specific subgroup analyses. As such, the current data support at most a conditional benefit of multimodality, rather than a broadly validated multimodal framework.

4. Early-fusion approach is not robust to missing modalities: The MLEF framework can only be applied to patients with complete multimodal data, which substantially reduces sample size and introduces potential selection bias. This is a major technical limitation, because missing modalities are common in real-world oncology datasets. A multimodal system that depends heavily on complete-case subsets has limited practical robustness and uncertain generalizability.

5. Complete-case restriction introduces substantial selection bias risk: Because early-fusion multimodal analyses require complete data, these results are based on a small and selected subset of patients. A matched RWD-only comparison on the same subset is useful, but it does not remove the concern that complete-case patients may be systematically different from the target population.

6. The amount of complete multimodal data is still limited: Although the total cohort is large, the number of patients with full multimodal coverage is relatively small. This limits the reliability of the multimodal analyses, particularly for deep learning models that require substantial data to learn stable cross-modal interactions. The reported multimodal gains may therefore reflect subset effects rather than true, generalizable multimodal synergy.

7. External validation is limited: The external validation cohort is valuable, but it comes from a single center and differs substantially from the development cohorts, including a notably longer median overall survival. The observed performance decline in EXVAL weakens claims of broad generalizability.

8. The comparison of model architectures is limited: The study contrasts only two integration strategies (MLEF and DLIF), without benchmarking against a broader range of contemporary multimodal architectures (e.g., transformer-based, late-fusion, or hybrid approaches). In addition, differences in data inclusion (e.g., complete-case vs. missing-modality settings) make the comparison less controlled. As a result, the conclusion that simpler machine learning approaches perform comparably to deep learning may be premature and not fully supported by a comprehensive architectural evaluation.

9. Pathology encoder evaluation is lacking: The digital pathology pipeline relies on one fixed encoder only, without comparison to other contemporary pathology backbones or slide-level aggregation methods. This substantially limits the technical rigor of the multimodal analysis, because the contribution of the pathology modality cannot be separated from the specific and unvalidated encoder choice.

10. Statistical testing is insufficiently transparent: The manuscript includes many subgroup analyses and pairwise comparisons, but the handling of multiplicity is not adequately described. In addition, the description of DeLong testing versus two-sided t-tests is unclear and should be clarified precisely.

Minor:
1. The genomics representation is relatively coarse, particularly the use of a composite actionable-driver variable, which may obscure biologically meaningful heterogeneity.
2. Radiomic analysis is restricted to the primary lesion and may miss clinically relevant information from metastatic sites and inter-lesional heterogeneity.
3. There are internal inconsistencies and editorial issues, including mismatch in reported total sample size across sections and visible formatting/reference-management artifacts.

In summary, this is a potentially impactful multicenter study addressing an important clinical problem. However, the present version makes claims that are stronger than the evidence currently supports. The clearest conclusion is that carefully curated clinical real-world data outperform common single biomarkers, while the incremental value of multimodal integration remains promising but not yet convincingly established.

(Remarks on code availability)

Open Access This Peer Review File is licensed under a Creative Commons Attribution 4.0 International License, which permits use, sharing, adaptation, distribution and reproduction in any medium or format, as long as you give appropriate credit to the original author(s) and the source, provide a link to the Creative Commons license, and indicate if changes were made.
In cases where reviewers are anonymous, credit should be given to 'Anonymous Referee' and the source.
The images or other third party material in this Peer Review File are included in the article's Creative Commons license, unless indicated otherwise in a credit line to the material. If material is not included in the article's Creative Commons license and your intended use is not permitted by statutory regulation or exceeds the permitted use, you will need to obtain permission directly from the copyright holder.
To view a copy of this license, visit https://creativecommons.org/licenses/by/4.0/

Dear Editor and Reviewers,

We thank you for the thorough evaluation of our manuscript and for the constructive and insightful comments. We have carefully considered all suggestions and have revised the manuscript extensively to address each point raised.

For clarity, reviewer comments are reported below, and our responses are provided in **blue**. All changes in the manuscript are reflected in the revised version.

Please note that for ease of readability, figures are embedded within the main text; however, high-resolution vector versions of all main and Extended Data figures have also been provided separately as PDF files.

We believe that these revisions have fully addressed the reviewers' comments and have significantly strengthened the manuscript. We hope that the revised version is now suitable for publication in *Nature Medicine*.

Reviewer 1

In this manuscript NMED-A147165 entitled "I3LUNG: Clinical Validation of a Multimodal AI Tool to Support Immunotherapy Decisions in NSCLC", Dr. Prelaj and colleagues present the results of a robust international, real word and multimodal-based translational effort that integrates clinical, radiological, genomic, and pathology data from more than 2000 patients into AI/machine learning models in order to generate a tool that can aid the clinical decision making process and guide patient selection for effective immune-based therapy. The authors demonstrate that machine-learning approach performs consistently across outcomes and achieves improved results in FL setting, whereas multimodal models outperformed real world data in clinically relevant subgroups and AI models were superior to other prognostic and predictive established or emerging factors/biomarkers. These results provide a potential platform for clinical applicability as it appears providers could achieve, once these results are prospectically validated, improved prediction with the AI-based tool.

The authors must be commended for this high-risk, herculean undertaking that integrates a non-trivial amount of cross-institutional and multidisciplinary patient and disease information to provide the community with a sophisticated and less subjective tool that can ultimately increase the benefit of immunotherapy in the most appropriate population of patients. These aspects bring to the findings substantial novelty, impact and generalizability for clinical utility. The need for prospective validation is clear and emphasized by the authors and it is indeed ongoing in large cohorts.

We sincerely thank the reviewer for this comment.

Major issues:

1. The authors clearly describe that the current analyses are focused on C2 and C3. Although I could appreciate why a C3 including stage 3 pts where IO therapy as consolidation has a curative intent represents a cohort with a prognostically different population, I feel there is a missed opportunity here to look at the role of an AI-based multimodal approaches specifically in the curative-intent disease setting.

Response to reviewer: We thank the reviewer for this comment. We would like to clarify that Cohort 3 (C3) does not include stage III patients treated with immuno-based therapy. C3 exclusively comprises pretreated patients who received IO-based therapy in the second or later metastatic lines of treatment in the advanced disease setting. Therefore, the analyses presented for C3 do not address curative-intent stage III disease.

Stage III patients treated with consolidation immunotherapy are included in the I3LUNG study within Cohort 1 (C1), which represents a biologically and prognostically distinct population. We agree that evaluating our models in the curative-intent setting is highly relevant. However, given the substantial clinical and biological differences between advanced pretreated disease and stage III, we deliberately focused the present analyses on the more homogeneous advanced-disease cohorts (C2 and C3) to avoid introducing additional heterogeneity. The results on C1 (stage III maintenance durvalumab) will be the subject of another upcoming publication. This is clarified in the main manuscript (line 122), online methods (724), and Figure 1a.

2. The authors should provide the results of analyses in the clinically relevant subgroups PD-L1<1%, 1-49% and ≥50%, in addition to the analysis with binary subgroups <50% or ≥50%.

Response to reviewer: We thank the reviewer for this important suggestion. In response, we have performed additional subgroup analyses stratified by the clinically relevant PD-L1 categories (<1%, 1–49%, and ≥50%). The results have the same trend as the other categories. The results are now included in Fig. 2g, Extended Data Fig. 2, Extended Data Fig. 3, Extended Data Fig.4.

3. I may have missed it, I do not see the min, max and median follow up time for survival analyses. Please provide, or bring to my attention, in text and figure legends.

Response to reviewer: We thank the reviewer for pointing this out. The median follow-up times (with ranges) for survival analyses are as follows:

TRAIN FU: 38.4 m (95%CI, 0.0-109.0)
TEST FU: 33.2 m (95%CI, 0.2-114.8)
EXVAL FU: 28.9 m (95%CI, 0.1-102.4)
CU FU: 39.7 (95%CI,0.2-63.6)

To improve clarity, we have now added these follow-up statistics to Table 1 in the revised manuscript.

4. Throughout the manuscript, it is complex and at times fatigue-inducing to follow the text depicting the corresponding panels in each figure because the text does not follow in the same order of each panel (for example, text describes Fig 1c, then 1d then 1b etc., similarly for Fig 2). Please consider reorganizing the text or, perhaps, would be more appropriate to reorganize the order of panels in the figures so that the order is aligned to the text describing each panel.

Response to reviewer: We sincerely thank the Reviewer for this constructive and helpful suggestion. Following this recommendation, we have carefully revised the manuscript to ensure full alignment between the text and the corresponding figure panels. Precisely, we have adapted the text to follow Figure 1, and for Figure 2 we have re-organised the subfigures to follow the text. In the revised manuscript, subfigures in Figure 3 are changed, and the text is adapted to follow the Figure. The remaining Figures (including extended data figures) and text have also been double-checked and adapted.

5. Table 1: I applaud the authors for a detailed amount of well-organized info. Please change the current header D" Disease control rate, n (%). What is shown is not the disease control rate but the RECIST categories. DCR is the proportion of patients with CR, PR and SD. Please rectify.

Response to reviewer: We thank the Reviewer. In the revised manuscript, Table 1 has been corrected by renaming "Disease control rate, n (%)" to "Best Response n (%)" to accurately reflect the data presented and be informative. Moreover, we added information on DCR (with categories yes or no), ORR (with categories yes or no), and CBR (with categories yes or no) based on the new endpoints.

6. Table 1: Please consider including molecular/driver (EGFR/ALK/RET/ROS1) subsets and also include KRAS, STK11 and KEAP1 subgroups and co-mutation subgroups. as these are available from genomic data. Please clearly describe how was PFS defined, as done for OS.

Response to reviewer: Thank you for the suggestion. In the revised manuscript, we have now added to Table 1 information regarding: Driver (ALK/ROS/RET/EGFR), EGFR alone, KEAP1, KRAS, and STK11.In addition, we have added the PFS definition to the online methods.

7. Please provide performance of the multimodal approaches in molecularly defined subgroups, namely KRAS, STK11, KEAP1 mutant and in the co-mutant subgroups.

Response to reviewer: We thank the Reviewer for this valuable suggestion. In the revised manuscript, we have performed the requested subgroup analysis using MLEF unimodal and bimodal, and we have provided the performance of the multimodal models within molecularly defined subgroups, including KRAS and P53 subgroups. These additional results have been included in Figure 2, and Supplementary Information 2 Figure 3 and Table 4. Unfortunately, we were unable to perform subgroup analyses of the

multimodal model in the STK11- and KEAP1-mutant populations due to the limited number of patients with available multimodal data in these subsets.

Subgroup analysis in CV C23 multimodal models were significant across outcomes.

8. The Discussion is overall thoughtful and well-written. The authors discuss their findings in the context of what's known in the literature and detail some of the limitations that are inherent to the nature of such study.
a. Can the authors expand in the Discussion on how they or the field should practically minimize the impact of heterogenous data quality from diverse centers for calibration and validation at each site before this tool can be deployed for clinical use?

Response to reviewer: We thank the Reviewer for this important and forward-looking comment. In the revised manuscript, we have expanded the discussion to address how heterogeneous data quality across centers may affect fair deployment. These clarifications have been added to the Discussion: "The reduced performance observed in the geographically distinct EXVAL cohort likely reflects differences in baseline clinical characteristics and outcomes, including longer OS and variations in treatment composition and histology distribution. Such distributional shifts can influence discrimination metrics even when the underlying predictive relationships remain valid. Therefore, to minimize the impact of heterogeneous data quality across centers, a prospective multicenter study with predefined calibration procedures will be essential to develop and validate model stability in real-world settings. Finally, continuous monitoring and periodic recalibration during deployment may further ensure reliable performance across institutions with different clinical workflows". (see lines 467 - 473 in the Discussion section of revised manuscript clean version).

b. It would be helpful to the lung cancer community if the authors could provide their thoughts and interpretation of the practical impact and utility of their results and the AI tool for daily clinical decisions by providers, implementing an approach in their description that is truly centered on clinically-relevant NSCLC subgroups in the advanced/metastatic setting.

Response to reviewer: We thank the reviewer for this important suggestion, which we believe strengthens the clinical framing of our work. Our preliminary results indicate that the I3LUNG tool may serve as a complementary decision-support instrument in advanced/metastatic NSCLC, particularly for junior clinicians or oncologists without dedicated thoracic oncology specialisation. Importantly, the tool is not intended to replace physician judgment and responsibility, but rather to support it by providing individualised, multimodal risk estimates that go beyond conventional subgroup analyses derived from registrational trials.

From a clinically relevant subgroup perspective:

- Patients with high PD-L1 expression (≥50%) and favourable predicted long-term survival (e.g., OS >24 months): In this subgroup, adding chemotherapy to immunotherapy may warrant careful

consideration. The tool may help identify patients for whom treatment de-escalation strategies could be explored, aligning with ongoing pragmatic de-escalation trial concepts.

- Patients in whom IO-based therapy may be insufficient: For patients predicted to have poorer outcomes with standard approaches, the tool may help identify candidates for intensified strategies, enrollment in clinical trials investigating novel combinations, or alternative therapeutic approaches.

- Prognostic information: The model may also help inform discussions with patients about prognosis, thereby potentially supporting shared decision-making. Ongoing research is evaluating how a tool dedicated to the patients may also inform patients about co-decision processes.

Within the I3LUNG framework, we are actively refining usability and physician experience using retrospective data. Final implementation will follow completion of the prospective phase. At present, the tool is not CE-certified and is not intended for immediate standalone clinical deployment. Rather, it should be viewed as a research-based decision-support model that complements current practice, which is still largely informed by subgroup analyses from pivotal trials rather than individualised multimodal prediction.

We have expanded the Discussion section (see lines 484- 489 in the Discussion section of revised manuscript clean version).

to more explicitly address the practical implications and subgroup-specific utility of the tool in advanced NSCLC.

Minor issues:

1. In the abstract the number of patients enrolled in this effort is reported to be 2365, but everywhere else (text and figures) I seem to depict 2396, what is the difference between these numbers?

Response to reviewer: We thank the reviewer for pointing this out. We have corrected the number in the abstract to 2396, as it is written everywhere else in the text.

2. Consider adding a description of clinical endpoints in Fig 1a.

Response to reviewer: We thank the Reviewer for this suggestion. In the revised manuscript, we have updated Figure 1a to include a clearer description of the clinical endpoints, including overall survival (OS).

3. I would suggest considering bringing the schema in Supp Fig 1 to Main or at least to Extended Data to provide the overview of cohorts utilized and undergoing validation.

Response to reviewer: Thank you for the suggestion. In the revised manuscript, we have moved the figure to the Extended Data Figure 1a.

4. In the Results text, “DCR responders” is not entirely accurate as R indicates a rate as proportion of patients with overall disease control so PR, CR and SD. For clarity, consider changing to simply “responders” (defined throughout including the methods to include also patients with SD) vs “non-responders” and remove DCR in front of responders, as you have basically done in Methods-Clinical Endpoints.

Response to reviewer: Thank you for the suggestion. We agree, and, therefore, we have changed ‘DCR responders’ to ‘responders' in the revised version of the manuscript.

5. Consider changing the title of paragraph reporting on survival analyses findings. In its current form, “Survival analysis achieved moderate results”, this title is unclear and does not convey what the key results are in a nutshell, as instead the other subsection/paragraph titles attempt to do.

Response to reviewer: We thank the Reviewer for this helpful suggestion. In the revised manuscript, we have modified the title of the paragraph reporting the survival analyses to better reflect the key findings and align it with the style of the other subsection titles (line 203). The title now is: **Survival analysis achieved consistent prediction across cohorts.**

Reviewer 2

This study addresses a highly relevant clinical question, how to improve individualized immunotherapy selection for advanced NSCLC. A major strength of the work is the assembly of one of the largest real-world, international, multicenter cohorts integrating clinical data, CT imaging, H&E digital pathology, and genomics. The scale of the dataset is impressive and position this work as a potentially important contribution to the field. The comments below are intended to help strengthen the methodological rigor and the clinical interpretability of the findings.

We sincerely thank the reviewer for this positive and balanced evaluation of our work.

1. There is substantial heterogeneity in performance across centers and subgroups, which raises concerns about model stability. For example, in the fairness/center analysis of the RWD-only MLEF classifier, the TPR for OS6 is 0.96 in MH versus 0.58 (INT), 0.62 (GHD), 0.62 (VHIO), 0.61 (SZMC). For DCR, MH again shows TPR=0.96 versus 0.63 (INT) ($p \leq 0.05$). This magnitude of variability suggests that performance is strongly center-dependent and may reflect differences in practice patterns, data completeness or follow-up rather than robust generalization. Please provide subgroup sample sizes, calibration by center and an analysis of potential drivers of heterogeneity.

Response to reviewer: We appreciate the reviewer’s thoughtful comments regarding the heterogeneity in performance across centers. We understand the concern that such variability may reflect differences

in practice patterns, data completeness, or follow-up rather than true model generalization. Below, we outline the steps we have taken to investigate and address these concerns.

- **Statistical Analysis Between Centers**

  To assess whether there is significant heterogeneity in model performance, we performed statistical analyses between centers by split (train/test) and outcome, and found that differences between centers do exist. The tables are included in **Supplementary Information 1, Tables 9-14.**

- **Mitigating Center Bias Using LOCO-CV**

  In the first version of the manuscript, to mitigate potential bias introduced by varying center-specific characteristics, we employed Leave-One-Center-Out Cross-Validation (LOCO-CV). This method allows us to train and validate the model on data from different centers while ensuring it does not overfit to any particular center. The results from LOCO-CV suggest that the observed performance differences across centres are not due to overfitting but likely reflect the underlying data characteristics of each center. This is specified in the **Online methods (line 722 and 737).**

- **Center-Specific Confusion Matrices for OS6**

  For further insight into the differences between centres, we provide a more detailed analysis of the predictions for the OS6 outcome, since the results are statistically different. These matrices demonstrate the number of true positives (TP), false positives (FP), true negatives (TN), and false negatives (FN) for each center. These results are added in the **Supplementary Information 5, Table 1.**

- **Calibration analysis**

  We performed a per-center calibration analysis for the OS6 MLEF CB-only model (LR C23). In this analysis, the model intercept was recalibrated separately for each center using its corresponding LOCO held-out training subset and subsequently evaluated on that center's independent test subset. **Table 2 in Supplementary Information 5** reports, for each of the five centers, the recalibrated intercept together with classification metrics computed before and after recalibration (TPR (sensitivity), FPR, and specificity). The recalibrated intercept values ranged from 0.272 (GHD) to 1.597 (SZMC). For MH, SZMC, and VHIO, post-calibration performance collapsed to TPR=1, FPR=1, and specificity=0. This behavior is most likely attributable to the extremely small number of negative samples in the respective LOCO test subsets. Under these highly imbalanced conditions, intercept recalibration resulted in a degenerate classifier that predicted all samples as positive. For GHD and INT, where class imbalance was less extreme, recalibration did not improve performance. These results suggest that center-specific intercept recalibration in this setting is unstable, particularly in the presence of limited sample size and severe class imbalance, and does not provide a consistent performance benefit.

In summary, the MH center's performance is significantly better than the other centers, with a very high TPR of 0.96. Specifically, MH has very few samples with OS < 6 months (class 0, only 2 patients), which could affect its model stability. This highlights the importance of having sufficient data from all classes to ensure reliable evaluation.

- **Center Prediction Model**

To assess how well the model predicts the center, we trained a multiclass logistic regression (LR) model on the OS6 dataset using the clinical features selected for the OS6 model. The model's performance on the OS6 test set was as follows:

- F1-macro Score: 0.222
- one-vs-all AUC: 0.633

Given the class imbalance, we also trained the same model to balance class weights. The model's performance on the OS6 test set was as follows:

- F1-macro Score: 0.282
- one-vs-all AUC: 0.624

These results suggest that the model struggles to distinguish between centers, indicating that the clinical features may not have sufficient discriminative power to reliably predict the center. This further suggests that the observed differences in TPR across centers are not driven by the model's ability to distinguish between centers, but rather by differences in data distribution. These results are included in the **Supplementary Information 5 Figure 1.**

- **SHAP Analysis Across Centers**

To investigate potential drivers of heterogeneity in model performance, we computed the mean SHAP feature differences across centers [1] **(Table 3 and Figure 2 Supplementary Information 5)**.

The results of this analysis indicate that the primary driver of the difference between MH and the other centers is the ECOG PS feature. Specifically, the SHAP value for ECOG PS in the MH cohort (0.32) is substantially higher than in the other centers (which range from -0.09 to 0.10), suggesting that the model relies much more heavily on this variable for its predictions within that specific center.
In contrast, other clinical features, including metastatic sites (bone, liver, brain) and smoking history, show minimal variations in SHAP values across the different sites, indicating a more stable contribution to the model's output. Furthermore, statistical analysis of baseline characteristics **(Supplementary Information 1, Table 3)** confirms that ECOG PS is significantly different across centers ($p<0.05$). This statistical heterogeneity in the dataset directly accounts for the observed divergence in SHAP values, identifying the underlying distribution of ECOG PS as the primary driver behind the model's center-specific internal logic and performance variations.

Conclusion

We acknowledge the heterogeneity in performance across centers and understand the concerns regarding model stability. To address these concerns, we have taken several steps, including: (a) statistical testing across centres and outcomes, (b) LOCO-CV (present also in the first version of the manuscript), and (b) model interpretability analysis using SHAP across centres. These analyses suggest that the observed differences in performance, particularly the higher TPR in MH (0.96), are likely due to factors such as class imbalance, insufficient data at
this centre, and differences in ECOG PS distributions across centres.
To better address and clarify this matter, we also added a small paragraph in the discussion (see lines 457-462 in the Discussion section of revised manuscript clean version).

1. Lundberg, Scott M. "Explaining quantitative measures of fairness." Fair & Responsible AI Workshop@ CHI2020. 2020.

In addition, we performed additional fairness analyses of the DLIF model across protected attributes and centers, the new analysis is reported in **Supplementary Information 5, Section 2, Figure 5.**

2. The use of a geographically distinct external validation (EXVAL) cohort is appropriate in principle. However, the EXVAL cohort differs substantially from the development cohorts in baseline characteristics and outcomes with a markedly longer median OS (22.2 months vs 12.5 months). This raise concerns that the EXVAL cohort represents a different-risk population rather than a true test of model generalitaton. Differences in line of therapy, histology type, sex and treatment composition (IO vs IO/chemo) may contribute to this survival gap. As a result, reduced performance in EXVAL for key clinical endpoints is difficult to interpret as either model failure or population shift. I recommend tempering claims of robust generalization.

Response to reviewer: We thank the Reviewer for this important observation. In the revised manuscript, we have expanded this concept (see lines 467- 473 in the Discussion section of revised manuscript clean version) to clarify that the reduced performance observed in the geographically distinct EXVAL cohort likely reflects substantial baseline and outcome differences compared with the development cohorts, including longer median OS, differences in treatment composition, line of therapy, histology distribution, and sex balance. These characteristics suggest a distinct risk profile rather than a pure test of model generalization. To further support this interpretation, we have performed additional comparative analyses of baseline characteristics across different outcomes, and these results are now provided in **Supplementary Information 1 Table 5-8.** In brief, the EXVAL test has significant differences in smoking status, PDL1, LDH, ECOG PS, Sex etc. We believe these additions help contextualize the modest decrease in AUC as a consequence of population heterogeneity rather than intrinsic model failure, reinforcing the need for prospective validation across diverse clinical settings.

3. Most of the reported gains from multimodal integration are derived from cross-validation rather than locked TEST or EXVAL analyses. While understandable given sample size constraints, this limits the

strength of the conclusions, as cross-validation remains an internal validation strategy and may be influenced by center-specific effects or overfitting effect. I suggest reframing cross-validation findings as exploratory and prioritizing locked TEST and EXVAL results when making claims about clinical performance and multimodal benefit.

Response to reviewer: In the new version of the manuscript, we prioritize reporting locked TEST and EXVAL if the tested patient cohorts are large enough. For instance, for the Logistic Regression C23 CB+DP+FMRAD model, given the limited number of patients with complete multimodal data in the TEST (n=47) and EXVAL (n=84) sets, we believe CV was the only statistically powered result. The small sample sizes in the TEST set yielded wide confidence intervals (0.27- 0.67 and 0.46-0.7) with p=0.47 and p=0.66, respectively, when comparing CB-only matched models, precluding meaningful conclusions about the multimodal benefit of TEST and EXVAL alone. All the results on the TEST and EXVAL cohorts for MLEF are now reported in **Supplementary Information 3.**

We agree that the lack of consistent replication in the TEST sets limits the strength of our multimodal conclusions. Accordingly, we have revised the manuscript to clearly frame the CV multimodal findings as exploratory.

That said, the CV analyses demonstrate that the models captured meaningful interactions between data modalities in specific subpopulations and were consistently superior to the CB-only (RWD-only) model within CV. These findings align with prior reports by Vanguri et al. 2022 and Captier et al. 2025, with the notable difference that our study included approximately 300 patients with complete multimodal data in CV, compared with around 80 patients at the intersection of all modalities in their works (Vanguri et al.2022 and Captier et al.2025).

For this reason, we emphasize that multimodal models require prospective validation in larger cohorts before clinical deployment. In fact, we selected the CB-only model as the basis for clinical deployment, explainable AI (XAI) analysis, fairness evaluation, and the clinical usability study, precisely because of its robustness and interpretability. Results for the CB-only model in the TEST and EXVAL cohorts are reported in the main text. To further clarify this distinction, we have structured the manuscript into separate sections discussing the CB-only and multimodal models' exploratory gains. We addressed this in different sections: Results (line 176-189, and 190-202) and Discussion in different points (line 401-408 and 422-426).

4. The MLEF approach excludes patients with missing modalities, resulting in potential complete-case bias. In real-world oncology practice, incomplete multimodal data are very common, making this issue central to clinical applicability. While DLIF is designed to address missing modalities, it does not consistently demonstrate improved performance with additional modalities, creating ambiguity about the practical value of multimodal integration.

Although the DLIF framework is designed to accommodate missing modalities, it does not consistently show improved performance with additional data, creating some ambiguity regarding the practical value of multimodal integration. This analysis would be strengthened by a clearer characterization of

missingness (by modality, center, outcome) and by exploring strategies beyond complete-case analysis, including missingness indicators, multiple imputation and architectures explicitly designed for missing modalities.

It would be also helpful to clearly state which modeling paradigm (MLEF vs DLIF) is envisioned for real-world deployment and why.

Response to reviewer: We thank the Reviewer for raising the critical issue of missing modalities and complete-case bias in multimodal modeling. We fully agree that real-world oncology data are commonly incomplete and that architectures explicitly designed to handle arbitrary missingness patterns are essential for clinical applicability.

To rigorously address this point and explore alternative intermediate-fusion paradigms, we evaluated two additional models specifically designed for missing-modality settings: DyAM (Vanguri et al. 2022) and MCVAE (Zanitti et al. 2025). Both models were evaluated on the same cohorts (C23, C2), outcomes (ORR, DCR, CBR, OS_6, OS_24), and evaluation splits (LOCO cross-validation, held-out test set, external validation) as MLEF.

DyAM (Vanguri et al. 2022). DyAM implements an intermediate-fusion framework that integrates radiology, pathology, and genomics through modality-specific hierarchical encoders followed by an attention-based joint representation. Missing modalities are handled via binary availability masks that gate each encoder's contribution at inference time, without requiring imputation.

MCVAE (Zanitti et al. 2025). MCVAE is a contrastive variational autoencoder designed for incomplete multimodal data. It incorporates modality-specific variational encoders, availability-aware gating, stochastic modality masking during training, and contrastive alignment to promote modality-agnostic representations. Full architectural details are provided in the Supplementary Methods.

Results: DyAM vs. DLIF. Across all outcomes and both cohorts (C23, C2), the two architectures showed similar CV AUC (~0.61–0.71). Performance at the test set was broadly consistent with CV for both models. Differences emerged at external validation and were outcome-dependent: for response and benefit endpoints (ORR, CBR), DyAM achieved higher EXVAL AUC; for survival endpoints (OS6, OS24), DLIF showed a consistent numerical advantage, though confidence intervals formally overlapped in most cases. In both models and across all outcomes, adding modalities beyond clinical variables did not yield consistent improvements in EXVAL; the clinical-only configuration was the most robust across all settings.

Results: MCVAE vs DLIF. Across all outcomes and both cohorts (C23, C2), MCVAE showed cross-validation AUCs broadly comparable to DLIF, consistent with equivalent in-distribution discriminative capacity. Performance at the held-out test set was similarly stable. On EXVAL, MCVAE showed outcome-dependent variability: for ORR, results were inconsistent across cohorts (C23: 0.38±0.07; C2: 0.54±0.08), with the C23 value falling below chance; for CBR and DCR, performance was comparable to DLIF with overlapping CIs; for survival endpoints, MCVAE performed below DLIF numerically (OS_6 C23: 0.65±0.08, C2

0.67±0.09) while OS_24 showed diverging cohort behavior (C23: 0.66±0.08; C2: 0.52±0.10). As with DyAM and DLIF, adding modalities beyond clinical variables did not yield consistent external validation improvements, and the clinical-only configuration remained the most robust setting throughout.

Full per-outcome, per-modality, per-cohort results and implementation details are provided in **Supplementary Information 4, Section 1, Figures 1-5**.

We also add this information to Results (line 251-253).

Conclusion. Across three architecturally distinct approaches to missing-modality fusion MLEF, DyAM, and MCVAE, results were consistent: multimodal integration did not yield systematic performance improvements over clinical-only models at external validation. This convergence across frameworks with fundamentally different missingness-handling strategies strengthens the robustness of our original conclusion and argues that the observed ceiling is driven by the data rather than by any single architectural choice.

5. The models are trained on patients receiving IO or IO/chemotherapy, which limits the ability to disentangle treatment-specific predictive value from general prognosis. From a clinical decision-making standpoint, prediction of benefit from immunotherapy relative to alternative treatments is far more impactful than prognosis alone. I would encourage inclusion of comparator cohorts treated with chemotherapy alone and/or EGFR-TKIs to assess treatment specificity.

Response to reviewer: We thank the reviewer for this important comment. We fully agree that distinguishing treatment-specific predictive value from general prognostic signal is critical for clinical decision-making. To address this point, we have collected and curated data from 4 additional cohorts, 3 of which are not part of the I3LUNG study. In detail:

1. **C-EGFR-INT**: EGFR-mutant IIIC-IV stage NSCLC patients treated with EGFR-TKIs (from INT).
2. **C-EGFR-UOC**: EGFR-mutant IIIC-IV stage NSCLC patients treated with EGFR-TKIs (from UOC).
3. **C-StageIII-CHT**: Stage IIIA-B NSCLC patients treated with neoadjuvant chemotherapy (from INT) and followed by surgery.
4. **C-LineI-CHT**: Patients from I3LUNG Cohort 3 who received first-line chemotherapy before second/further-line IO (baseline chemotherapy features were used for the model, while in the I3LUNG IO-Model, we used features at baseline immunotherapy).

We added these details in the online methods (line 580-588)

We were unable to retrieve a historical cohort of patients treated with first-line chemotherapy alone without subsequent immunotherapy, as these cases date back several years, and complete laboratory data were not consistently available or retrievable.

Given that only CB features were consistently available across these additional cohorts, we evaluated the MLEF CB model, which was the most robust and generalizable within the I3LUNG framework. For these analyses:

- Models trained on the combined C23 dataset were applied to the non-IO cohorts.
- For C-LineI-CHT, since these patients are already part of the original I3LUNG C3 (but evaluated at first-line chemotherapy), we applied the model trained exclusively on C2, to avoid overlap and potential information leakage.

The results of these treatment-specific analyses are now reported in **Figure 3e** and described in the main manuscript (line 278 - 288). Other more detailed results are in **Supplementary Information 2 Table 5.** These additional experiments allow a more rigorous assessment of whether the model captures signals specific to IO-treated populations versus broader prognostic information.

In particular, the results, across endpoints, were the best and with the most consistent performance in the IO test cohort (C23 TEST, I3LUNG IO cohorts), whereas performance was attenuated and/or more uncertain in both C-EGFR and C-StageIII-CHT, particularly for response endpoints (e.g., ORR AUC 0.57±0.10 in C-EGFR1; 0.50±0.13 in C-EGFR2; 0.58±0.12 in C-S3CH).

To further assess performance in a first-line chemotherapy-treated population, we extracted baseline chemotherapy features from Cohort 3 (C3) (see above). In this setting, OS discrimination remained moderate (AUC 0.72±0.09 for OS6; 0.64±0.08 for OS24), potentially reflecting the impact of downstream IO exposure on survival outcomes. In contrast, response endpoints remained lower (ORR/DCR AUC both 0.58), consistent with findings in other cohorts, suggesting that objective response under chemotherapy does not recapitulate the immunotherapy-specific predictive patterns captured by the model. This further supports the positioning of the I3LUNG model as a potential predictive, beyond prognostic tool.

We also compared the difference between the I3LUNG cohorts and the new ones, reported in **Figure 3e** and **Supplementary Information 2, Table 5.**

To summarize, the pattern of results demonstrates that the strongest and most consistent discrimination was achieved in the IO-treated test cohort (C23 TEST, I3LUNG), whereas performance attenuated and became less stable when the model was applied to cohorts treated with alternative therapies, including EGFR-TKIs and chemotherapy alone. If the model were capturing only general prognosis, we would expect similar discrimination across therapeutic contexts. Instead, the reduced performance observed in non-IO cohorts suggests that the model captures, at least in part, a treatment-associated predictive signal specific to IO exposure rather than a purely prognostic effect. We acknowledge that several non-IO cohorts had smaller sample sizes, class imbalance, and limited event numbers, resulting in wider confidence intervals and reduced statistical power. These limitations have been explicitly clarified in the revised manuscript. Nonetheless, the differential performance across treatment settings supports the presence of IO-specific predictive patterns beyond baseline prognosis.

We added a new paragraph in the discussion of the manuscript (line 448-455)

6. Please provide clearer guidance on how the final model and decision thresholds were selected and fixed prior to evaluation in the EXVAL cohort.

Response to reviewer: We thank the reviewer for this important point and agree that the process required a clearer explanation. In particular, no decision threshold was applied for the primary evaluation in the EXVAL cohort. All models were trained exclusively on the training cohort using LOCO and then applied directly to the TEST and the EXVAL cohort, without threshold adjustment.

The final model for the fairness, explainability, and clinical usability analyses was selected after comparing candidate models. The MLEF CB model was selected because, despite its relative simplicity (logistic regression using CB data only), it achieved performance comparable to the more complex multimodal approaches (MLEF multimodal and DLIF uni- and multimodal models), while offering greater interpretability and clinical implementability.

For the fairness, explainability, and clinical usability analyses only, a fixed decision threshold of 0.5 was used for the selected MLEF CB model (previously referred to as the RWD model). This threshold was not optimized on EXVAL data and was applied solely to facilitate binary classification analyses in those downstream evaluations.

We have clarified this workflow in the revised manuscript (see Online Methods, lines 736-741).

7. Although the overall cohort is very large (2396 patients with clinical data), the effective sample size for multimodal analyses is substantially smaller and imbalanced. While CT scans are available for 1705/2396 patients (71%) in the full collected population, in the actual study population used for model development (C2-C3, n=2075), CT availability drops to 896/2075 patients (43%). The overlap across modalities is even more limited, 339 patients having all four modalities (clinical, CT, pathology, genomics). This is important as it may indicate bias selection of patients from specific centers with more complete diagnostic workflows and may correlate with prognosis, treatment patterns and follow-up intensity. As a result, observed multimodal performance may partially reflect complete-case selection bias rather than true incremental predictive value from additional modalities. To address this limitation, the study could benefit from: a systematic analysis of missingness by center, treatment and outcome; comparison of baseline characteristics and overall survival between multimodal-complete and modality-incomplete patients; and sensitivity analyses using approaches that can leverage partial modality availability, rather than restricting analyses to complete cases.

Response to reviewer: We thank the Reviewer for this important observation regarding the potential for selection bias in the multimodal cohort. We have addressed this concern through three distinct analyses:

1. Baseline Characteristics and Selection Bias

A systematic comparison of baseline characteristics between patients with complete multimodal data and those with incomplete data revealed statistical differences in a limited subset of features, most notably

ECOG PS and smoking history. Importantly, while ECOG PS is a known prognostic factor, it is inherently subjective and prone to inter-observer variability, which may account for the observed statistical discrepancy. These tables are added to the **Supplementary Information 1, Tables 16-27.**

2. Model Robustness across Populations (Generalizability)

To determine if the multimodal population represents a "biased", easier-to-predict subset, we tested the performance of the CB-only (previously referred to as CB) MLEF model on both the multimodal-complete and the modality-incomplete populations.

- **Results:** Across the main cohorts (C2, C23) and outcomes (OS6, OS24, DCR), there were no statistically significant differences in AUC performance between the two groups (permutation test $p > 0.05$). These results are added in **Supplementary Information 2, Table 3.**
- **Interpretation:** In several instances, the CB-only MLEF model performed nominally better but not statistically significantly better in the *incomplete-data* population. This empirically shows that the multimodal cohort does not suffer from a significant positive selection bias that can influence the better predictive performance.

3. *Incremental Value (Sensitivity Analysis)*

To confirm the true incremental value of additional modalities, we performed an ablation analysis strictly within the multimodal-complete subset. This shows that the performance gain is due to data integration and not a change in the patient population.

- **Findings:** The transition from CB to CB+DP and CB+RAD showed consistent, statistically significant improvements in AUC (e.g., in C2 OS6: CB 0.689, CB+FMRAD 0.843, $p < 0.01$). These results are added to **the Supplementary Information 2, Table 2.**

We also performed a similar analysis for the DLIF model (reported in **Supplementary Information 4, Section 2, Fig. 6-19**), and results showed a similar trend, highlighting the fact that lack of data modalities are crucial for these architectures.
We referred to these analyses in the main manuscript and Online Methods (739-741)

Minor comments:

- Font sizes in several main and supplementary figures are too small to be legible. This significantly difficult interpretability and should be corrected.

Response to reviewer: We thank the Reviewer for highlighting this. In the revised manuscript, we have increased the font sizes across the main and supplementary figures to improve clarity and legibility.

- Clarify censoring and label definition for OS6 and OS24 to avoid potential follow-up bias.

Response to reviewer: We thank the reviewer for highlighting the importance of clearly defining censoring and these landmark endpoints. For both OS6 and OS24, we defined binary survival endpoints using a landmark approach:

1. OS6:
    a. Class 0: patients who died within 6 months from treatment initiation.
    b. Class 1: patients who survived ≥6 months.
2. OS24:
    a. Class 0: patients who died within 24 months.
    b. Class 1: patients who survived ≥24 months.

To avoid misclassification and follow-up bias, patients with follow-up shorter than the respective landmark time (i.e., <6 months for OS6 and <24 months for OS24) who were still alive at last contact were excluded from the corresponding analysis. This is also the reason for the different numbers in the TRAIN, TEST, and EXVAL datasets used for different outcomes. This ensured that survival status at the landmark time point could be determined with certainty for all included patients.

We have clarified this definition and the handling of censored cases in the revised manuscript to ensure transparency (Online methods lines 616 - 619).

- The paper states baseline clinical features are collected before the first IO cycle. However, the acceptable timing window for CT/H&E relative to IO initiation is not clearly quantified in the main manuscript. Given disease dynamics, longer intervals could materially affect both radiomics and pathomics. Please report the distribution of scan/biopsy-to-treatment intervals.

Response to reviewer: We thank the reviewer for raising this important methodological point. Clinical and blood variables were collected at baseline prior to initiation of IO. Genomic data and H&E slides were obtained at the time of diagnosis or at IO initiation; when biopsy material at IO start was available, it was preferred CT scans were collected mostly at the baseline of IO administration, but since this is a real world study, in those patients where CT was not available at baseline IO, we also included CT scans at diagnosis. In detail, almost all CT scans were performed at baseline, before IO initiation. In total, 110 patients (12% - 33 in C2 and 77 in C3) did not have available pre-IO CT scan and 41 (5% - 26 in C2 and 15 in C3) received surgery. For 23 patients (3% - 16 in C2 and 7 in C3), CT scans were collected after IO initiation.

To clarify this point, we have now added analyses reporting the distribution of days passed between biopsy/CT scans acquisition and start of IO, for digital pathology and CT scan data, respectively (**Supplementary Information 7, Figure 1a and 2a (i-ii**). This addition allows readers to assess the range and distribution of CT scan/biopsy-to-treatment intervals and their potential impact.

Finally, to assess whether this timing variability affected the multimodal model, we previously conducted CV analyses comparing models built with and without CT data, not at the IO baseline. No meaningful

performance differences were observed, and the multimodal models remained statistically superior to the matched CB-only models.

We have also clarified this in the main manuscript to improve transparency, Online Methods, line 627-630.

- "RWD" vs "clinical data" is confusing. CT scans, H&E slides, and targeted genomics are also routine real-world clinical data. Using "RWD" to mean only structured clinical variables may mislead readers about what is "real-world" versus "research-grade." Consider re-labelling as "clinical variables" (or similar) and describing imaging/pathology/genomics as routine clinical data modalities.

Response to reviewer: We thank the reviewer for this helpful suggestion, which has improved the clarity of the manuscript. We have replaced the term "RWD" with "Clinical+blood laboratory exams (CB)" throughout the manuscript, including in all figures and figure legends, to ensure consistency and avoid potential confusion

- The authors note that "contrast-enhanced scans were preferred when available," and that contrast information varies by center. Because intra venous contrast status can strongly influence PyRadiomics features (and potentially FM embeddings), please report the proportion of contrast vs non-contrast scans by center and test robustness.

Response to reviewer: We thank the reviewers for the suggestion. The graphs with contrast and non-contrast metadata information across centers are reported in **Supplementary Information 7, Figure 1b-c**. In brief, most CT scans were acquired with contrast (around 85%), and we have performed robustness analyses to assess PYRAD features extracted from contrast versus non-contrast CT scans. Differences were observed, likely due to the limited number of non-contrast images (***Supplementary Information 7, Robustness Analysis***). However, excluding non-contrast images did not impact performance in either unimodal or selected multimodal models using PYRAD features.

- DCR is defined with SD + PR + CR as "responders" vs PD as "non-responders". Stable disease is heterogeneous, and "best response = SD" does not necessarily imply meaningful benefit, especially without duration. Consider reporting ORR (CR/PR) separately and/or a clinical benefit endpoint (e.g., CR/PR or durable SD ≥6 months), which is more decision relevant.

Response to reviewer: We thank the reviewer for this clinically relevant suggestion. In response, we have expanded the analyses to include both objective response rate (ORR) and clinical benefit rate (CBR) as additional endpoints. The corresponding results are now provided in **Supplementary Information 2, Figures 1 and 2** for MLEF and **Supplementary Information 4, Section 1, Figures 1 and 2** for DLIF.

Specifically:

- **ORR** was defined as CR + PR versus SD + PD.

- **CBR** was defined as CR + PR + SD with PFS ≥6 months versus PD and SD with PFS <6 months, thereby incorporating a durability criterion for stable disease.

Reviewer #2 (Remarks on code availability):

A detailed review of the code would be time-consuming. I would be happy to review the code if the manuscript progresses further, provided that the key methodological concerns raised above are addressed.

Reviewer 3

General comments An elegantly-conceived, -conducted, and -reported observational study designed to develop and evaluate multivariable models for prediction of objective tumour control (disease control response = CR, PR, or SD), and for prediction of survival beyond 2 years, following treatment with an immune checkpoint inhibitor.

The work is highly original and innovative, particularly in its depth, breadth, rigour, and the sophistication of both development and evaluation.

The data, approaches, and methods are rigorous, well-described, and appropriate.

Statistical methods are sound, current, and appropriate.

The conclusions are suitably tempered and supported by the reported data.

I enjoyed reading it, and learnt much about the new methods, but it was hard work given the density and complexity of analyses and results.

The study raises many intersting unanswered questions about prognostication, decision-making, and doctor-patient communication.

We sincerely thank the reviewer for this encouraging evaluation of our work.

Specific Suggestions

1. Figure 1a bottom left panel, and elsewhere: please use appropriate numbers of icons (human symbols) to reflect the relative sizes of TRAIN vs TEST vs EXVAL, i.e. TRAIN ~ 6 x TEST and EXVAL. Current figures imply (exaggerate) that TEST and EXVAL are half the size of TRAIN.

Response to reviewer: Thank you for the suggestion. We have adapted **Figure 1a** to reflect the differences in dataset size. While the number of icons does not exactly reflect the true proportions due to space limitations, it now clearly conveys the much larger size of the TRAIN set.

2. Lines 173-181, 186-192, and 193-200: please include actual p-values (i.e. $p = X$), rather than 'significance levels' (i.e. $p \leq X$).

Response to reviewer: Thank you for the suggestion. We have now reported the exact p-values throughout the revised manuscript when $p >= 0.0001$; for smaller values, we report $p < 0.0001$ in accordance with standard reporting practices. See [2] for details.

[2] Mansournia, Mohammad Ali, and Maryam Nazemipour. "Recommendations for accurate reporting in medical research statistics." *The Lancet* 403.10427 (2024): 611-612.

3. Comparisons of multivariable models versus single biomarkers in isolation are not very persuasive. Would anyone recommend making decisions about therapy based solely on LDH, or NLR? PS is useful for identifying individuals unlikely to benefit from treatment, but not for selecting one treatment over another.

Response to reviewer: We thank the reviewer for this important comment. We agree that, in clinical practice, treatment decisions are rarely based solely on a single biomarker such as LDH or NLR. Our intention was not to suggest that therapy selection should rely on any of these individual markers. Rather, our objective was to contextualise the performance of the proposed multivariable models against biomarkers that are currently used more for routine clinical reasoning than for decision-making. One of the main motivations for our study was the lack of good biomarkers for clinical decisions. Therefore, the comparison with single-marker predictions is not in the sense of comparing with the gold standard, but rather to provide a measure of the incremental value of the ML models.

At present, most clinical decision-making relies either on PD-L1, composite scores such as LIPI, or subgroup analyses of individual biomarkers (e.g., LDH, NLR, ECOG PS) coming from Randomized Clinical Trials. Therefore, we included these single biomarkers as a reference to reflect the current standard of care and to provide a clinically meaningful context for the added value of our models.

Importantly, our goal was to demonstrate that the proposed ML models outperform commonly used individual clinical or blood-based markers, thereby supporting their incremental value over current practice. We have clarified this in the revised manuscript (line 396-399).

4. In many places, the abbreviation for confidence intervals is reversed (IC instead of CI).

Response to reviewer: Thank you. We have changed IC to CI everywhere in the text, table, and figures.

5. Figure 4 c includes many interesting and innovative visualisations of unfamiliar indices, e.g. SHAP values, diagnostic test performance statistics. These need better explanation for the intended readership.

Response to reviewer: We thank the Reviewer for this valuable comment. In the revised manuscript, we have expanded the Online Methods Explainability section to provide clearer explanations of these indices, their interpretation, and their clinical relevance.

6. Line 318, please explain prediction of 'DCR' more clearly in the manuscript methods and results. The sentence is confusing, because it is easy to mistake that the categories for prediction of DCR were intervals of time in months. It took much backing and forthing, before I realised from the Figure 5a that the question was 'DCR yes v no'. The R in DCR is defined as 'rate' earlier in the text. It would be clearer to use the abbreviation DCR for disease control Response (CR, PR, or SD). This needs to be clearer and used consistently throughout the manuscript.

Response to reviewer: We thank the Reviewer for this valuable comment. We revised the manuscript and corrected it (line 348-352 in revised clean manuscript).

7. Lines 323-326. The stated results indicate that predictions of non-experts were substantially better than those of experts (AUC 0.77 vs 0.68 without XAI, 0.90 vs 0.83 with XAI.) Is this correct? This warrants discussion. It raises questions about the design, phrasing, and interpretation of both the questions and the answers.

Response to reviewer: We thank the reviewer for carefully examining this section. We would like to clarify that the values reported in Lines 323–326 (in the original manuscript) are not AUCs; they correspond to prediction sensitivity, as reported in Table 28 (Phase 1) and Table 34 (Phase 2) of ***Supplementary Information 6*** and the main manuscript, line 353-355 in revised cleaned manuscript.

Importantly, the sensitivities of experts and non-experts were not statistically different in either Phase 1 or Phase 2 (see **Tables 28 and 34 in *Supplementary Information 6***). Therefore, the results do not indicate that non-experts outperformed experts. We agree that such a conclusion warrants careful analysis, but it is not supported by the statistical analysis.

What we observed instead was a within-group improvement when explainable AI (XAI) support was provided. Specifically:

- Non-experts' sensitivity increased from 0.77 to 0.90 (p = 0.021),
- Experts' sensitivity increased from 0.68 to 0.83 (p = 0.020)

- So, we can conclude that XAI significantly improved sensitivity in both groups, indicating a reduction in false negatives. However, there was no statistically significant difference between experts and non-experts in either phase.

We acknowledge that the previous phrasing may have led to confusion by suggesting a significantly higher sensitivity in non-experts. We have revised the manuscript (Line 353-359 in revised cleaned manuscript) to clearly state that the reported values refer to sensitivity, to emphasize the within-group improvements with XAI, and to clarify that no superiority between clinician groups was observed.

8. Given the differences between 'experts' and 'non-expert', it would be helpful for the definitions and/or characteristics of these 2 groups to be explained in the body of the manuscript, e.g. lung vs non-lung expert oncologists, as explained in 705-710, rather than expert vs non-expert.

Response to reviewer: Thank you for the suggestion. In the revised manuscript, we have changed the phrasing and renamed 'experts' to 'lung expert oncologists' and 'non-experts' to 'non-lung expert oncologists'.

9. Line 326. Clarify what is meant by a "37% increase in the likelihood" by defining and including the two numbers being compared.

Response to reviewer: We agree that the term "likelihood" was imprecise in this context. The reported 37% increase refers to an odds ratio of 1.37 from the logistic regression model comparing Phase 2 versus Phase 1 (**Supplementary Information 6, Table 4**). We have therefore replaced the term "likelihood" with "odds" to ensure statistical accuracy. In the revised manuscript, we explicitly report the odds ratio of 1.37 and clarify that it reflects an increase in the odds of the outcome in Phase 2 compared to Phase 1 (line 359-361).

10. Line 327 (p=0.1) is sufficient, no need to report p to 4 significant figures.

Response to reviewer: Thank you, we have changed it to p=0.1.

11. The improvement in line 326 is overemphasised given the p-value in line 327 indicates well within the play of chance.

Response to reviewer: Thank you for the comment. We have rephrased the sentence, line 359.

12. The survival predictions are interesting, but again, somewhat naïve. It is unrealistic to expect a point-estimate of the predicted survival time. Even when perfectly calibrated, one would expect no more than a third of actual survival times to be within +/- one third of the expected survival time. A probabilistic

prediction would have been more appropriate, e.g. probability of surviving more than x months, or estimated median survival time (in a group of similar individuals).

Response to reviewer: We thank the reviewer for this important comment. We would like to clarify that physicians were not asked to provide a numeric survival time. Instead, they were asked to assign each patient to one of five predefined OS intervals (≤6, 6–12, 12–18, 18–24, ≥24 months), clarified in the main text (line 348-352), online methods (line 849-851), and **Supplementary Information 6**. Importantly, the OS model generated a full predicted survival curve for each patient. For the purpose of the usability study and comparison with physician assessments, the continuous survival distribution was discretized into these predefined categories.

Therefore, the OS usability task should be interpreted as a coarse categorization of a probabilistic survival distribution rather than as an attempt to predict an exact individual survival time. We agree that probabilistic outputs (e.g., probability of surviving beyond a given time) are more appropriate representations of survival modeling, and this is indeed how the model operates internally.

13. Sorry if I've missed, but what are the 'correctness' scores for the AI predictions of survival? Based on lines 718-19, I'm guessing that the survival time interval including the 50th percentile of the OS probability curve for each patient generated by AI would most closely correspond with the OS 'prediction' clinicians completed.

Response to reviewer: Thank you for the question. Here is some clarification. The correctness score for the AI's survival prediction was defined in the online methods (line 887 893 in the revised manuscript).

"Due to the time-to-event nature of the OS endpoint (i.e., it is not possible to define "true positives" and "true negatives" when speaking of survival), it was not possible to compute any of the aforementioned metrics for the OS prediction. Therefore, we have developed a correctness score for the OS prediction defined for each patient, based on the true OS and the physician's predicted OS ranges as follows:

- 0 points were assigned in case of different and non-consecutive ranges for the true OS and the predicted OS by the physician;

- 1 point was assigned in case of different, but consecutive, ranges for the true OS and the predicted OS by the physician;

- 2 points were assigned in case of identical ranges for the true OS and the predicted OS by the physician.

The Wilcoxon signed-rank test for paired data was used to assess whether the distributions of the assigned scores differed significantly. A total correctness score was computed by summing all scores across phases and physicians' expertise."

14. Line 442, please spell out and explain 'NLP'

Response to reviewer: Thank you. We have now added a full name, i.e., "natural language processing".

15. Lines 450-452. None of these factors, other than presence of targetable, oncogenic driver mutations, were ever ‘sufficient to capture the complexity of’ response to treatment.

Response to reviewer: Thank you very much. We totally agree. Therefore, we have rephrased the sentence in the conclusion section of the Revised Manuscript:

“In conclusion, the first I3LUNG PDSS demonstrates that single biomarkers, composite scores, or subgroup analyses derived from randomised clinical trials are not sufficient to capture the complexity of IO effectiveness/response.”

16. Line 462, clarify in the text what the “>200” in parentheses refers to (participants?).

Response to reviewer: In the revised manuscript, we have clarified that this number refers to patients (line 524).

17. Line 595, KRAS mutations not counted as actionable, as appropriate from 2012-23. Should be mentioned in limitations, along with advent of new targets and treatments.

Response to reviewer: We thank the reviewer for this important observation. In our study, “actionable driver alterations” were defined as oncogenic alterations with established, guideline-recommended first-line targeted therapies during the study period (2012–2023), such as EGFR, ALK, ROS1, and RET. These alterations are supported by robust clinical evidence showing limited/uncertain benefit from immunotherapy in oncogene-addicted NSCLC, which was directly relevant to our modeling strategy. Moreover, for this driver we also have a first-line TKI therapy.

Importantly, the drivers included in our definition are generally considered negative predictors of response to immunotherapy and their negative role has been also confirmed in our models. In contrast, KRAS mutations were not classified as actionable in our analysis because, for most of the study period, KRAS-targeted therapies were not available as standard first-line treatment. Moreover, KRAS-mutant tumors, particularly in the absence of co-occurring STK11 or KEAP1 alterations, are often associated with relatively better responsiveness to immunotherapy than other oncogene-addicted subtypes. For these reasons, KRAS was conceptually and clinically distinct from the driver alterations included in our “actionable” category; for this reason we included KRAS as an individual feature in the model.

18. Please include in the body of the manuscript a sentence that specifies the 9 RWD predictive features selected (mentioned in lines 510 and 610).

Response to reviewer: Thank you for the suggestions. We have included this info in the main manuscript, specifically in the Results section, Overview of the Study Design paragraph (line 113-117).

19. Line 609, please clarify in the manuscript what is meant by “starting from 227”.

Response to reviewer: Thank you. In the revised manuscript, we have clarified this point by writing “starting from 227 features”.

20. Please clarify in the manuscript whether you considered/included age, comorbidity measures, or frailty measures. And discuss these as either strengths or limitations.

Response to reviewer: We thank the reviewer for raising this important point and for allowing us to explain it in more details. Age and comorbidity-related variables were collected within the I3LUNG framework. Age variables and comorbidity data had minimal missingness (e.g., 3.3% missing for the information on whether the patient had concomitant diseases), indicating good data availability.

To assess their contribution, we performed a fully ML data-driven modelling approach, including all 227 features, among which we also included:

- **Age variables:**
    - Age at diagnosis
    - Age at metastatic disease
    - Age at IO initiation

- **Comorbidity-related variables:**
    - Presence and number of concomitant diseases
    - Cardiovascular, renal, pulmonary, hepatic, autoimmune, endocrine, infectious, gastrointestinal, and neurological disorders
    - Diabetes, dyslipidemia
    - Prior/other malignancies (multiple site-specific categories)

These analyses are reported in **Supplementary Information 7, Table 1,** and described in the Online Methods.

Model performance using the data-driven approach was nearly identical to that of the hypothesis-driven model. Notably, for the MLEF C233 model, only endocrine disorders, but still not among the first 5 features, were selected (for OS24), while no age or other comorbidity variables were selected for OS6 or DCR.

Given the absence of meaningful performance improvement and in favour of interpretability, we retained the hypothesis-driven feature set as the primary modelling approach. We have clarified in the manuscript (online methods lines 566-568) that age and comorbidity measures were collected; a full list of the 227 features is in **Supplementary Information 1 Table 1**, while extended data analysis across centres is available in **Supplementary Information 1 Tables 3-4.**

21. Line 726, I think it should be “Similarly, two…”, not “Similar two…”

Response to reviewer: Thank you for pointing this out. In the revised manuscript, we have corrected it to “Similarly, two..”.

22. Line 726, prediction within 25% of actual is an unusually narrow margin for success; 33% is more commonly used. Prediction within 0.75 to 1.33 times actual would be even better.

Response to reviewer: We thank the reviewer for this important methodological observation.

We acknowledge that a ±33% margin is frequently used in studies evaluating clinicians’ temporal survival estimates. However, there is no universally accepted standard threshold, and the acceptable margin varies across studies depending on study design and clinical context.

In our analysis, the ±25% margin ([observed OS − 25% to observed OS + 25%]) was selected to ensure a consistent and comparable definition of accuracy between the model and physicians’ predictions. Because clinicians assigned patients to relatively broad predefined survival intervals, we aimed to adopt a criterion that would not artificially inflate predictive performance.

We agree that alternative margins (as the reviewer suggested, 0.75-1.33) are also reasonable and could be considered in future analyses. To address this point, we have already clarified the rationale for the chosen threshold in the revised manuscript in the online methods (line 858-861) to ensure transparency in our methodological choice.

Reviewer 4

1. Summary of the Key Results

This manuscript reports on the retrospective validation of the I3LUNG AI decision support system using a large cohort (n=2,396) of NSCLC patients across six international centers. The study compares two architectures, i.e., Machine Learning Early Fusion (MLEF) and Deep Learning Intermediate Fusion (DLIF), to integrate RWD, radiomics, pathology, and genomics. Notably, the MLEF models (AUC ~0.77) outperformed standard biomarkers. A key, albeit counter-intuitive, finding is that while multimodality helped in specific subgroups, models using RWD alone performed comparably to complex multimodal models in the general cohort. The inclusion of a usability study showing increased sensitivity for physicians (0.72 to 0.87) is a significant addition.

2. Originality and Significance

This work presents a large-scale, international multi-center study encompassing 2,396 patients from six countries, which shows undoubtable superiority against similar research. by highlighting the advantage of leveraging routine real-world data over rarer metrics like TMB. While the core concept of multimodal immuno-oncology prediction is not novel, the study demonstrates clear novelty in its execution, scale, and translational focus. This is evidenced not only by its detailed comparative analysis of multiple ML/DL models but, more importantly, by the inclusion of a “Clinical Usability” study assessing human-AI interaction. This translational component, combined with the substantial dataset, elevates the work above many purely computational pathology papers and strongly justifies its high potential impact.

We sincerely thank the reviewer for this thoughtful and encouraging assessment of our work.

3. Data & Methodology

- 3.1 Validity of the Approach: The framework is generally sound, but I have reservations about the composite "driver" feature. Lumping EGFR, ALK, RET, and ROS1 into a single binary variable is methodologically risky. Biologically, EGFR often predicts resistance to IO, whereas other drivers may behave differently. Collapsing them risks washing out the signal. A sensitivity analysis separating EGFR is necessary.

Response to reviewer: We thank the Reviewer for this important methodological concern. The rationale for the composite driver encoding stems from the marked imbalance and substantial missing data observed in our cohort for individual oncogenic drivers. Conversely, the composite driver variable addresses this by pooling rare events into a more robust binary predictor while maintaining biological interpretability. More information on the mutation distribution in c23 can be found in Table 15 in Supplementary Information 1.
To address the validity of our composite driver feature, we conducted a comprehensive sensitivity analysis comparing two approaches: (Model 1) the original composite driver (EGFR ∨ ALK ∨ ROS1 ∨ RET), and (Model 2) a stratified model separating EGFR as an independent feature while retaining ALK/ROS1/RET as a composite variable. We performed DeLong's test to compare AUC values across all clinical endpoints, with 95% confidence intervals. We report the CV results in **Table 2 in Supplementary Information 7**.
Some outcomes show statistically significant differences, but the absolute performance is uniformly low (AUCs close to 0.5). This indicates limited discriminative ability regardless of whether EGFR is separated or collapsed into the composite driver.
We repeated the sensitivity analysis for CB data + composite driver (EGFR, ALK, ROS1, RET) and CB data + driver with a separated EGFR feature.

Finally, SHAP analysis showed that EGFR mutations and ALK/ROS1/RET translocations features were associated with negative contributions to IO prediction, suggesting lower predicted benefit from immunotherapy and that their combination within the same feature is biologically justified.

Conclusion: the decision to propose a combined score is driven by the significant missingness in the data available to us. Experiments separating the EGFR driver from the other oncogenic drivers provide only minimal improvement in the model's performance.

Regarding the clinical usability study, a key methodological detail requires clarification. The oncologists were provided with both global and local SHAP explanations. However, given that the underlying features range from clinically interpretable variables to abstract representations from foundation models, it is crucial to specify: (1) how these two distinct types of explanatory information were presented to clinicians (e.g., in an integrated or separate manner), and (2) how the meaning of the more abstract features was explained to ensure they were correctly interpreted.

Response to reviewer: We thank the reviewer for highlighting this important point and apologise for the lack of clarity.

We would like to clarify that, in the clinical usability study, the model presented to clinicians was the MLEF Clinical+blood (CB, previously referred to as RWD) model (machine learning logistic regression trained exclusively on clinical and blood variables). This model does not include abstract representations derived from foundation models or any other image-based feature. All features used in this model are standard clinical or laboratory variables that are routinely interpreted by physicians in daily practice.

For each patient case, clinicians were provided with:

1. The individual feature values (including measurement units) for all features considered important for clinical practice, despite their relevance in the model.
2. An explicit indication when a value was missing.
3. CT slices with a segmented tumour region and an H&E slide, whenever available, even if not used by the model.
4. Local SHAP explanations showing the direction and magnitude of each CB feature's contribution to the prediction for that specific patient.
5. Global SHAP summaries describing overall feature importance across the cohort.
6. Tutorials on how to read these graphs.
7. Details about the model

Global and local explanations were presented in a complementary manner: the global explanations provided context for overall model behaviour, while the local explanations focused on patient-specific decision support. Because all features in the MLEF-CB model were clinically interpretable variables, no additional explanation of abstract or latent features was required, as reported in the online methods.

We have revised the manuscript to clarify that only the Clinical+blood model was used in the usability study and to explicitly describe how global and local SHAP explanations were presented (line 343-345 and 840-847).

- 3.2 Quality of the Data: The explicit handling of real-world heterogeneity is a strength. However, the sample size for full multimodal integration is relatively small (n=339). This makes it difficult to accept the conclusion that the DLIF approach failed to outperform MLEF; it is equally likely that the deep learning model simply lacked sufficient data to converge.

Response to reviewer: We thank the Reviewer for this thoughtful and fair comment. We agree that the relatively limited number of patients with complete multimodal data (n=339) represents an important constraint when interpreting the comparative performance of DLIF and MLEF.

To address the concern that DLIF may have underperformed due to insufficient data for convergence, we performed additional analyses. Specifically, we re-ran two alternative DLIF architectures to verify that the findings were not specific to a single deep learning implementation. Across these architectures, performance remained comparable and did not significantly improve over the RWD-only models (***Supplementary Information 4 Section 1***). In fact, this consistency suggests that the observed results are more likely driven by the extent and pattern of missing multimodal data coverage rather than by limitations of a specific AI architecture.

Beyond sample size considerations, several additional factors may explain the limited incremental benefit of full multimodal DLIF integration:

1. *High missingness burden:* Even within the multimodal subset, modality availability was uneven. DLIF architectures are designed to accommodate missing inputs, but when missingness is substantial or systematically patterned, the effective signal available for cross-modal representation learning may be reduced.
2. *Signal redundancy rather than complementarity:* Especially because deep learning models ingest the full set of extracted features from all modalities (e.g., ~4,000 features from FM-CT and ~750 from digital pathology), the inclusion of high-dimensional inputs in the context of incomplete coverage may introduce additional noise rather than incremental signal, especially when you have many more features than data.
3. *Strong baseline signal in CB:* Clinical and blood-derived variables already carried a robust prognostic and predictive signal. When a dominant modality captures a large proportion of explainable variance, additional modalities may yield only marginal incremental gain, particularly in real-world cohorts.

Taken together, these additional analyses and considerations suggest that the absence of a significant DLIF advantage reflects intrinsic data characteristics, heterogeneity, missingness patterns, and signal distribution, rather than a failure of deep learning convergence per se. We have clarified this interpretation in the revised Discussion and tempered claims regarding multimodal superiority accordingly (line 422-431).

- 3.3 Quality of Presentation:

3.3.1. Quality and Consistency of Figures and Visualizations

The current presentation of figures significantly hinders the comprehension of the complex methodology and results.

（1） Please update the figures to vector graphics; currently, the text and numbers are pixelated and difficult to read.

Response to reviewer: We thank the reviewer. We have submitted the high-resolution vector graphic format to ensure optimal readability of text, numbers, and annotations across formats and zoom levels.

（2） Inconsistent Notation and Ambiguous Labels:

1) The matrices representing the modalities in Figure 1a are confusing regarding their dimensions and shapes—these need to be labeled more clearly. I also noticed a notation discrepancy in Figure 3a. The schematic on the left labels the encoder input/output dimensions as d_i and d_z (and similarly for the MIL fusion), but the detailed component diagrams on the right use different labels for these same dimensions.

Response to reviewer: We thank the Reviewer. We have revised the figures to improve clarity and ensure consistent notation throughout.

Specifically, in **Figure 1a** we have fixed the dimensions and shapes to the modality matrices. In addition, in **Figure 3a**, we have added zi inside the boxes to make it clearer.

2) Figure 2f: The caption contains an error. "Each row represents one patient" should be corrected to "Each column represents one patient."

Response to reviewer: We thank the reviewer. The caption has been corrected in the revised manuscript.

(3) The nomenclature for the four core data modalities is inconsistent across figures, creating unnecessary confusion. Figure 1a uses: Clinical, Radiomics, Pathomics, Genomics. Figure 1b uses: RW Data, Digital Pathology, Radiomics, Genomics. Figure 3a uses: Clinical, Digital Pathology, Radiology, Genomics. Please review and rigorously unify the terminology for these four input types across all figures and their captions in the main text.

Response to reviewer: We thank the Reviewer for pointing out this inconsistency. We have now rigorously harmonized the nomenclature of the four core input modalities across all figures, captions, and the main text. A single, consistent terminology is now used throughout to avoid ambiguity and improve readability.

All previous discrepancies (e.g., “Clinical” vs “RW Data,” “Radiomics” vs “Radiology,” “Pathomics” vs “Digital Pathology”) have adopted the following nomenclature: CB - clinical+blood, FMRAD and PYRAD - radiology, DP - digital pathology, and G - genomics in the revised manuscript.

(4) Optimization of Visual Encoding in Results Figures: Figures 2g & 3c: The current dual encoding (circle size and color hue) for representing sample size and model performance, respectively, is suboptimal for rapid interpretation. Suggestion:

1) Directly annotate the sample size (N) next to the model name on the y-axis (e.g., RWD-only (N=xxx)).
2) Use a single, consistent circle size for all data points.
3) Encode performance solely via a color gradient. Since no AUC values are below 0.5 in these plots, set the color map range from 0.5 to 1.0. This will enhance contrast and make performance differences across models and datasets more visually apparent.

Response to reviewer: We thank the Reviewer for these constructive and detailed suggestions regarding visual encoding. We have carefully revised **Figures 2g and 3c** and implemented at our best the proposed improvements to enhance interpretability.

Specifically, we set the circle size across all data points to avoid dual encoding ambiguity. Model performance is now represented exclusively by a color gradient, and the colormap range has been adjusted to span from 0.5 to 1.0, thereby improving visual contrast and facilitating clearer comparison across models and datasets. We have added * when the multimodal model outperformed the CB-only for the same subset. Moreover, we have selected just some subgroup analyses to present in these plots as part of the main manuscript; the rest were moved to the Extended data.

These modifications substantially improve figure readability.

3.3.2. Presentation and Interpretation of Results

(1) Simplification of Subgroup Analyses: the subgroup performance summaries are dense. Please consider simplifying these to highlight the most clinically actionable findings.

Response to reviewer: We thank the Reviewer for this helpful suggestion. To improve clarity and clinical relevance, we have simplified the subgroup analyses and now focus specifically on the high PD-L1 population treated with IO monotherapy. The rest of the subgroup analysis has been moved to the **Extended Data Figure 2-4.**

(2) Missing Baseline Analysis and Potential Model Bias:

1) Demographic Characteristics: Table 1 (or a supplementary table) must report the distribution of key

demographic variables (e.g., sex, age) across the training, test, and external validation sets, as well as across the cohorts defined by different prediction tasks (OS24, OS6, DCR). A statistical comparison for differences is essential to assess dataset comparability and potential sampling bias.

Response to reviewer: We thank the Reviewer for this important suggestion, which aligns with reviewer 1 request. We have now added the distribution of key demographic variables (including sex and age) across the training, test, and external validation sets, as well as across the cohorts defined by the different prediction tasks (OS24, OS6, DCR).

In addition, we performed appropriate statistical comparisons to assess differences between sets and to evaluate potential sampling bias, as well as a detailed analysis by the center as requested by Reviewer 1. These results are now reported in **Table 1** and **Supplementary Information Table 1-12**, ensuring transparency regarding dataset comparability.

2) Analysis of Subgroup Performance & Fairness: Figure 4a-b suggests potential biases that require discussion and clarification.

The notable variation in DLIF performance across centers and the substantial difference in TPR (>10%) between male and female subgroups for MLEF are concerning for model generalizability and fairness. Please discuss these observations in the context of the model's potential limitations. Crucially, the methods section should state whether and how mitigation strategies were employed to address potential biases related to sex or center effects during model development. If not, this should be acknowledged as a limitation.

Response to reviewer: We acknowledge the observed disparity in TPR between sexes, with sensitivity consistently higher in the female subgroup by more than 10% across all outcomes (e.g., 96% vs 77% for OS 24 and 80% vs 68% for DCR), although the differences are not statistically significant (p=0.054, p=0.15). To investigate whether this performance gap stems from algorithmic bias, we conducted a SHAP-based analysis examining feature-level contributions across sex subgroups in OS 24 and DCR test set patients (**Figure 3 and 4, and Table 4 and 5, Supplementary Information 5**).

In the OS 24 model, sex is selected by the LASSO algorithm and, as expected, sex itself shows the largest mean SHAP difference between male and female (**Figure 3a, Supplementary Information 5**). No other clinical feature, including NLR, ECOG PS, metastasis sites, or PDL1 category, shows a statistically significant difference in mean SHAP contribution between male and female patients (i.e., all the 95% CIs cross zero). This indicates that the model applies the same decision logic to other clinical variables. This is not surprising as no formal bias mitigation strategies for sex were applied during model development (in particular, in the OS24 case, sex was selected as a feature).

To strengthen our conclusions, we further analyzed the feature contributions using SHAP bar plots across the train, test, and EXVAL sets **(Figure 3b-d, Supplementary 5)**. In each of these sets, sex consistently ranked among the least contributing features to the model's predictions. This provides additional evidence that, despite the observed disparity in TPR between sexes, sex does not appear to play a significant role in the model's decision-making process across different stages of model evaluation.

Regarding the DCR model, the sex feature was not selected by LASSO, and SHAP analysis showed no concerning differences in other features between females and males, **Figure 4, Supplementary Information 5**.

3.3.3. Use and Definition of Abbreviations

There are also too much abbreviations used in the paper and some abbreviations are unclear and might cause trouble for reading, particularly non-intuitive ones like 'G' for genomics (provides no information actually). It would be helpful if the authors could: 1) improve their usage of abbreviations (and probably reduce the usage of unnecessary ones),

2) ensure that all abbreviations are clearly defined / referred upon first use.

Response to reviewer: Thank you. We have revised the manuscript to reduce unnecessary abbreviations to improve overall clarity. In particular, we have replaced non-intuitive shorthand (e.g., “G” for genomics) with clearer terminology where appropriate. Additionally, we have carefully reviewed the manuscript to ensure that all abbreviations are explicitly defined at first use.

4. Appropriate Use of Statistics and Treatment of Uncertainties

- External Validation Drop: The model performance drops in the external validation cohort (EXVAL, UOC-USA) compared to the test set (e.g., DCR AUC 0.71 in TEST vs 0.55 in EXVAL). The manuscript attributes this to "population shifts" (e.g., longer survival in UOC). This explanation is insufficient. The authors must perform a rigorous error analysis. Is the drop due to specific missing features in the US cohort, differences in CT acquisition, or biological heterogeneity?

Response to reviewer: We thank the reviewer for this comment. To further investigate the performance drop in the EXVAL cohort, we performed a systematic statistical comparison of the clinical variables used in our CB models between the TRAIN, TEST, and EXVAL cohorts using chi-squared tests for categorical variables, Mann–Whitney U tests for non-normally distributed continuous variables between 2 groups comparison and Kruskal-Wallis Test for Medians between 3 group comparisons. This analysis showed that several key clinical features are significantly imbalanced in EXVAL compared with both TRAIN and TEST,

including sex, ECOG PS, LDH, smoking status (current and former), PD-L1 expression category, and overall survival distributions (all $p < 0.05$) (**Table 5-8, Supplementary Information 1**). These results indicate that the EXVAL cohort represents a clinically distinct population, and support our interpretation that the performance drop is largely driven by a shift in the underlying clinical feature distributions rather than by model instability. We have expanded the discussion to reflect this point.

- Fairness Audit: The inclusion of fairness metrics is commendable. However, significant variability in sensitivity was observed across centers (e.g., MH vs. INT). This center bias needs to be addressed before claiming the tool is ready for global deployment.

Response to reviewer: Thank you for pointing this out. We understand the concern that such variability may reflect differences in practice patterns, data completeness, or follow-up rather than true model generalization. Below, we outline the steps we have taken to investigate and address these concerns.

- **Statistical Analysis Between Centers**

  To assess whether there is significant heterogeneity in model performance, we performed statistical analyses between centers by split (train/test) and outcome, and found that differences between centers do exist. The tables are added to the **Supplementary Information 1 Table 9-14.**

- **Mitigating Center Bias Using LOCO-CV**

  In the first version of the manuscript, to mitigate potential bias introduced by varying center-specific characteristics, we employed Leave-One-Center-Out Cross-Validation (LOCO-CV). This method allows us to train and validate the model on data from different centers while ensuring it does not overfit to any particular center. The results from LOCO-CV suggest that the observed performance differences across centres are not due to overfitting but likely reflect the underlying data characteristics of each center. This is specified in the **Online methods (line 722 and 738).**

- **Center-Specific Confusion Matrices for OS6**

  For further insight into the differences between centres, we provide a more detailed analysis of the predictions for the OS6 outcome, since the results are statistically different. These matrices demonstrate the number of true positives (TP), false positives (FP), true negatives (TN), and false negatives (FN) for each center. These results are added in the **Supplementary Information 5 table 1.**

- **Calibration analysis**
-

  We performed a per-center calibration analysis for the OS6 MLEF CB-only model (LR C23). In this analysis, the model intercept was recalibrated separately for each center using its corresponding LOCO held-out training subset and subsequently evaluated on that center's independent test

subset. **Table 2 in Supplementary Information 5** reports, for each of the five centers, the recalibrated intercept together with classification metrics computed before and after recalibration (TPR (sensitivity), FPR, and specificity). The recalibrated intercept values ranged from 0.272 (GHD) to 1.597 (SZMC). For MH, SZMC, and VHIO, post-calibration performance collapsed to TPR=1, FPR=1, and specificity=0. This behavior is most likely attributable to the extremely small number of negative samples in the respective LOCO test subsets. Under these highly imbalanced conditions, intercept recalibration resulted in a degenerate classifier that predicted all samples as positive. For GHD and INT, where class imbalance was less extreme, recalibration did not improve performance. These results suggest that center-specific intercept recalibration in this setting is unstable, particularly with limited sample size and severe class imbalance, and does not provide a consistent performance benefit.

The MH center's performance is significantly better than the other centers, with a very high TPR of 0.96. Specifically, MH has very few samples with OS < 6 months (class 0, only 2 patients), which could affect its model stability. This highlights the importance of having sufficient data from all classes to ensure reliable evaluation.

- **Center Prediction Model**

To assess how well the model predicts the center, we trained a multiclass logistic regression (LR) model on the OS6 dataset using the clinical features selected for the OS6 model. The model's performance on the OS6 test set was as follows:

- F1-macro Score: 0.222
- one-vs-all AUC: 0.633

Given the class imbalance, we also trained the same model to balance class weights. The model's performance on the OS6 test set was as follows:

- F1-macro Score: 0.282
- one-vs-all AUC: 0.624

These results suggest that the model struggles to distinguish between centers, indicating that the clinical features may not have sufficient discriminative power to reliably predict the center. This further suggests that the observed differences in TPR across centers are not driven by the model's ability to distinguish between centers, but rather by differences in data distribution. These results are added in the **Supplementary Information 5, Figure 1.**

- **SHAP Analysis Across Centers**

To investigate potential drivers of heterogeneity in model performance, we computed the mean SHAP feature differences across centers [1] **(Table 3 and Figure 2, Supplementary Information 5)**.

The results of this analysis indicate that the primary driver of the difference between MH and the other centers is the ECOG PS feature. Specifically, the SHAP value for ECOG PS in the MH cohort (0.32) is

substantially higher than in the other centers (which range from -0.09 to 0.10), suggesting that the model relies much more heavily on this variable for its predictions within that specific center.

In contrast, other clinical features, including metastatic sites (bone, liver, brain) and smoking history, show minimal variations in SHAP values across the different sites, indicating a more stable contribution to the model's output. Furthermore, statistical analysis of baseline characteristics **(Supplementary Information 1)** confirms that ECOG PS is significantly different across centers (p<0.05). This statistical heterogeneity in the dataset directly accounts for the observed divergence in SHAP values, identifying the underlying distribution of ECOG PS as the primary driver behind the model's center-specific internal logic and performance variations.

Conclusion

We acknowledge the heterogeneity in performance across centers and understand the concerns regarding model stability. To address these concerns, we have taken several steps, including: (a) statistical testing across centres and outcomes, (b) LOCO-CV (present also in the first version of the manuscript), and (b) model interpretability analysis using SHAP across centres. These analyses suggest that the observed differences in performance, particularly the higher TPR in MH (0.96), are likely due to factors such as class imbalance, insufficient data at this centre, and differences in ECOG PS distributions across centres.

In order to better address and clarify this matter, we also added a small paragraph in the discussion (line 456-466).

1. Lundberg, Scott M. "Explaining quantitative measures of fairness." Fair & Responsible AI Workshop@ CHI2020. 2020.

5. Conclusions: Robustness and Validity

The abstract claims multimodal models outperformed RWD in clinical-specific subgroups. While statistically true, the bigger story here seems to be the robustness of the RWD-only models. Highlighting the efficacy of RWD-only models could actually strengthen the paper's appeal for low-resource settings. Furthermore, the claim that this framework mitigates overfitting requires more evidence, especially given the degradation seen in the external validation cohort.

Response to reviewer: We thank the Reviewer for this thoughtful and strategic comment. We agree that the robustness of the CB (previously RWD-only) models represents an important and clinically meaningful finding, particularly in real-world and low-resource settings where multimodal data may not be consistently available. Across both TEST and EXVAL cohorts, the CB-only models demonstrated stable and reproducible performance for OS6, OS24, and DCR endpoints. We have revised the manuscript to more clearly emphasize this robustness, positioning it as a key strength of the framework. Regarding overfitting, we agree that this claim requires careful framing. In the revised manuscript, we have tempered the

language and clarified that the performance degradation observed in EXVAL likely reflects a population shift.

6. Reference, Clarity and Context

The abstract and introduction are lucid and provide excellent context. The distinction between MLEF (Early Fusion) and DLIF (Intermediate Fusion) is clearly articulated in the methods.

Response to reviewer: We thank the Reviewer for this positive and encouraging feedback.

Dear Editor and Reviewers,

We thank the Editor and the Reviewers for the thorough evaluation of our manuscript and for the constructive and insightful comments provided during this second round of revision.

We thank the Editor for his suggestions. We have carefully revised the manuscript to address the reviewers' comments, with particular attention to moderating claims regarding clinical validity and real-world readiness. These revisions have been implemented throughout the title, abstract, results, discussion and conclusion to ensure that the results appropriately support the conclusions.

For clarity, reviewer comments are reported below, and our responses are provided in **blue**. All corresponding changes have been incorporated into the revised manuscript.

We believe that these revisions have addressed the reviewers' comments and that the manuscript may now be suitable for publication in *Nature Medicine*.

Figures

For ease of readability, figures are embedded within the main text. High-resolution vector versions of all main and Extended Data figures have also been provided separately as PDF files.

Figures 1a, 3e and 5a were created with BioRender.com while the rest of the figures were arranged using BioRender.com, and we confirm that the first author has a valid licence.

Data and Code Availability

We confirm that the Data Availability Statement is included in the main manuscript.

Data availability

The I3LUNG project is an ongoing EU-funded initiative, with related analyses and manuscripts expected through June 2027. Therefore, the full raw multimodal dataset cannot be released at this time. However, de-identified data supporting the findings of this study, specifically, CB and genomics data and the extracted features from medical images (radiomics, FM for CT scans and digital pathology, both from GigaPath and TITAN) will be made available upon the acceptance for publications on ZENODO, and the data will be available for 20 years.

During the review process, reviewers can access the data needed to reproduce the study by downloading the zip folder data.zip. In line with the consortium's data-sharing policy, the full raw datasets from all modalities (including images) will become available five years after the official end of the project.

Code availability

The code used for preprocessing, model training, and statistical analyses is available on GitHub. For now, the repository is private (https://github.com/AI-ON-Laboratory/I3LUNG_PDSS), but reviewers can access (and clone) the repository with the extended code, pipelines, and models using this link: https://gitfront.io/r/CristinaLicciardello/ueXioAMbCAAt/I3LUNG-PDSS/. Upon acceptance, the repository will be made publicly available.

Sex and Gender reporting:

1. If the research findings apply to only one sex or gender, that must be indicated in the title and/or abstract.

Response: Our research does not apply only to one sex or gender.

2a. For studies involving vertebrates animal and cell lines. The Reporting Summary should include whether sex was were considered in the study design.

Response: Our research does not involve vertebrates animal or cell lines.

2b. For studies involving human research participants- The Reporting Summary should include whether sex and/or gender was considered in the study design and whether sex and/or gender of participants was determined based on self-report or assigned (and methodology used).

Response: During data collection, we have collected information about the assigned sex of the patients. This is included in the reporting summary and is clearly stated in the main manuscript.

3. Data should be reported disaggregated for sex and gender where this information has been collected and consent has been obtained for reporting and sharing individual-level data; disaggregated numbers for individual experiments must be provided in the source data as appropriate whereas overall numbers may be provided in the Nature Portfolio Reporting Summary.

Response: Sex data will be made publicly available for all patients, together with other information required to replicate the study (including self-reported race), upon acceptance. During the review process, these data are available to the Editors and Reviewers.

We have also submitted the updated Nature Research Reporting Summary and the Nature Research Software Policy checklist.

Reviewer #1 (Remarks to the Author):

The authors have appropriately addressed my comments and incorporated my suggestions accordingly in the revised manuscript which is clearly improved in strength and quality after thoughtful input from all referees. I have no further comments.

Response to Reviewer: We thank the reviewer for the positive and constructive feedback. We are pleased that the revisions have improved the quality of the manuscript, and we appreciate the reviewer's acknowledgment.

Reviewer #2 (Remarks to the Author):

Thank you for your detailed and thoughtful responses, which have clearly improved the clarity and transparency of the manuscript. I particularly appreciate the additional analyses and the effort to better contextualize the findings.

As a remaining point, the performance decrease observed between TEST and EXVAL (OS24 AUC 0.74 vs 0.60; DCR 0.71 vs 0.55) is important and would benefit from a slightly more explicit discussion in the main text, particularly in terms of implications for model robustness across settings.

Response to Reviewer: We thank the reviewer for this important point. We have now explicitly addressed the observed performance decrease in the EXVAL cohort in the revised manuscript, in lines 390-395 of the discussion.

It may be helpful to more explicitly articulate the current stage of clinical applicability of the model, particularly how the outputs could be integrated into clinical decision-making.

Response to Reviewer: We thank the reviewer for this important point. We have now explicitly added a new paragraph outlining the current and next steps needed for integrating I3LUNG in the clinic (lines 462-465 in the conclusion).
In particular, at its current stage, in the I3LUNG ongoing project, the model's clinical applicability is evolving from retrospective validation toward real-world implementation. A usability study is ongoing within the I3LUNG platform for the multimodal model (CB+Rad+DP model), together with an implementation analysis to assess integration into clinical workflows and decision-making (and costs to be useful for low and middle-income countries). In parallel, a silent prospective validation is being conducted to evaluate performance in a real-world setting without influencing clinical decisions. In addition, a pragmatic randomized clinical trial is under design to formally assess prospective clinical utility and impact on outcomes as a registrational trial. This pathway is aimed at supporting regulatory approval and CE marking, with expected clinical deployment within the next 2-3 years

Reviewer #3 (Remarks to the Author): Stats expert

Comments and suggestions addressed.

Response to Reviewer: We thank the reviewer for the positive and constructive feedback. We are pleased that the revisions have addressed the suggestions.

Methods and results reasonable.

Response to Reviewer: We thank the reviewer for the positive assessment of the methods and results. We are pleased that the work is considered sound and appropriate.

Still hard work to wade through.

Response to Reviewer: We thank the reviewer for this important comment. We have carefully revised the manuscript to improve readability, including simplifying the text, reducing redundancy, and improving the flow in each section (abstract, results, discussion, and conclusion).

Claims exceed findings, comparators are poor, title is hybperbolic: not validated until evaluated in separate datasets.

Response to Reviewer: We thank the reviewer for this important comment. We have carefully tempered our claims throughout the manuscript, including revisions to the title, abstract, discussion and conclusion. In particular, we have clarified the scope of the findings, reduced any potentially overstated conclusions, and explicitly acknowledged the need for prospective validation in independent datasets before clinical deployment.

Reviewer #4 (Remarks to the Author):

This manuscript describes the retrospective arm of the I3LUNG project, an international real-world effort to develop AI models for immunotherapy decision support in advanced NSCLC using clinical data, CT, digital pathology and genomics. The study addresses an important clinical problem and benefits from a relatively large multicenter dataset. However, the manuscript currently overstates the level of validation and clinical readiness achieved. The strongest and most consistent results appear to come from the RWD-only models, whereas the multimodal advantage is limited, inconsistent, and largely confined to selected complete-case analyses. As written, the title, abstract and discussion go beyond what the data convincingly support.

Major:

1. Overstatement of validation level: The manuscript is framed as a “clinical validation” of a clinically relevant decision-support tool. In reality, this is a retrospective model-development and validation study with an exploratory usability component, while prospective validation remains ongoing. The title, abstract and discussion should be aligned more closely with the actual level of evidence.

Response to Reviewer: We thank the reviewer for this important observation and agree with the concern. In response, we have revised the title, abstract, discussion and conclusion to better reflect the actual level of evidence. Specifically, we have removed any implication of formal clinical validation and clarified that this study represents a retrospective model development and validation effort and added the clinical usability study, with a prospective validation study and clinical implementation currently

ongoing. These changes ensure a more accurate and appropriately cautious representation of the study's scope and maturity.

2. The study is primarily prognostic rather than truly treatment-predictive: Although the manuscript is framed as supporting immunotherapy decision-making, the current analyses are conducted within IO/IO-CHT treated cohorts and mainly identify patients with better or worse outcomes. This is more consistent with prognostic modeling than with treatment-specific predictive modeling. Without an appropriate comparative treatment framework, it remains unclear whether the model identifies patients who specifically benefit from immunotherapy, rather than those with generally better prognosis.

Response to Reviewer: We thank the reviewer for this important and insightful comment. We agree that distinguishing between prognostic and truly treatment-predictive modeling is critical. As correctly noted, our analyses are primarily conducted in cohorts treated with IO or IO-CHT, and therefore largely capture outcome stratification within these treatment groups.

However, we would like to note that, in the current clinical landscape, first-line chemotherapy alone is no longer a standard comparator, which makes it inherently challenging to construct an appropriate untreated or alternative-treatment control group for formal predictive modeling. This limitation is especially relevant for the prospective component of the study, as, for ethical reasons, you cannot consider CH alone as a comparator for these patients.

To partially address this point, we have expanded the analyses by including additional cohorts, incorporating CT-based data and EGFR-mutant populations, as detailed in the revised manuscript Version 1 (V1) (i.e., section entitled: *"Machine-learning models showed greater predictive performance in IO cohorts compared to CHT and EGFR-cohorts" in the main manuscript and corresponding* Figure 3e). These analyses were intended to provide preliminary insight into treatment-specific behavior beyond purely prognostic associations.

We have now clarified this aspect more explicitly in the manuscript, emphasizing that the current results should be interpreted primarily as prognostic (lines 426-430 Discussion). Importantly, we would like to highlight that prognostic stratification within immunotherapy-treated populations remains highly clinically relevant and can meaningfully support decision-making. For instance, identifying patients with a low likelihood of benefit from IO-based therapies (IO or CHT/IO) may help clinicians consider alternative strategies based on the patient's clinical condition, such as referral to clinical trials or early consideration of best supportive care. Conversely, the ability to identify patients with a high likelihood of benefit (maybe to IO or IO-CHT), particularly among those with high PD-L1 expression, may support more personalized approaches, including potential de-escalation strategies (e.g., IO alone versus CHT-IO) (lines 442-447 discussion).

3. Lack of consistent benefit from multimodal integration: A central claim of the manuscript is that multimodal integration improves prediction of immunotherapy outcomes. However, the results do not

show a consistent or robust advantage over RWD-only models. Performance gains appear to be selective, endpoint-dependent, and often limited to specific subgroup analyses. As such, the current data support at most a conditional benefit of multimodality, rather than a broadly validated multimodal framework.

Response to Reviewer: We thank the reviewer for this important comment and agree that the benefit of multimodal integration is not consistently demonstrated across all analyses. This concern was carefully addressed in the revised manuscript V1. Specifically, we have prioritized reporting results from the locked TEST and EXVAL cohorts when sample sizes are sufficient, and we have moved additional results to the Supplementary Information to ensure transparency.

Importantly, we acknowledge that the limited number of patients with complete multimodal data in the TEST and EXVAL sets (e.g., n=47 and n=84 for selected models) results in wide confidence intervals and limited statistical power, precluding definitive conclusions regarding the added value of multimodality in these cohorts. As such, we explicitly framed in the revised manuscript V1 that the multimodal findings derived from CV were exploratory, rather than definitive.

At the same time, the CV analyses consistently showed that multimodal models captured meaningful interactions between data types and outperformed CB-only (first version of the manuscript, RWD-only) models within specific subpopulations. These findings are in line with prior studies (e.g., Vanguri et al., 2022; Captier et al., 2025), while our study benefits from a larger multimodal sample size (~350 patients with complete data in CV and ~400 without genomics: CB+RAD+DP, which appears to be the best model in CV).

To reflect these considerations, we have revised the manuscript, and, in the revised manuscript V1, clearly present multimodality as providing conditional and context-dependent benefits, rather than a universally robust advantage. Furthermore, given its consistency, robustness, and interpretability, we selected the CB-only (RWD-based) model as the primary model for clinical deployment, including explainability analyses, fairness evaluation, and the usability study.

These revisions are explicitly detailed in the Results (section *Machine learning-based CB-only models provide robust IO outcomes prediction* and *Multimodal integration showed exploratory gains*) and Discussion sections (lines 396-399), ensuring a more balanced and accurate interpretation of the role of multimodal integration.

4. Early-fusion approach is not robust to missing modalities: The MLEF framework can only be applied to patients with complete multimodal data, which substantially reduces sample size and introduces potential selection bias. This is a major technical limitation, because missing modalities are common in real-world oncology datasets. A multimodal system that depends heavily on complete-case subsets has limited practical robustness and uncertain generalizability.

Response to Reviewer: We fully agree with the limitation highlighted. We have revised the manuscript to better rephrase and clarify this point. In particular, we now explicitly acknowledge that early-fusion approaches such as MLEF require complete-case data and are therefore inherently limited in real-world settings, where missing modalities are common and may introduce selection bias.

At the same time, we emphasize that while MLEF-type approaches can be useful to explore the potential value of fully integrated multimodal data in well-characterized subsets, their applicability in routine clinical practice remains limited. In real-world datasets, more flexible methods that can explicitly handle missing modalities are essential and will need to be integrated for robust clinical deployment. (Discussion Line 399-403, 450-452).

5. Complete-case restriction introduces substantial selection bias risk: Because early-fusion multimodal analyses require complete data, these results are based on a small and selected subset of patients. A matched RWD-only comparison on the same subset is useful, but it does not remove the concern that complete-case patients may be systematically different from the target population.

Response to Reviewer: We thank the reviewer for this important comment and agree with the concern regarding potential bias introduced by complete-case analyses. We have also explicitly added this point as a limitation in the revised manuscript V1.

At the same time, as part of the previous revision, we performed additional analyses to assess potential differences between patients with complete multimodal data and those with missing modalities. These analyses aimed to evaluate whether the complete-case subset is systematically different from the broader study population. The results of these comparisons are reported in the revised manuscript V1, **Supplementary Information 1, Tables 16-27**. While these analyses provide some reassurance, we acknowledge that they do not fully eliminate the risk of bias. Accordingly, we emphasize this limitation and the need for future approaches that can robustly handle missing data in real-world settings.

In the previous revised manuscript V1: we have addressed this concern through three distinct analyses:

1. Baseline Characteristics and Selection Bias

*A systematic comparison of baseline characteristics between patients with complete multimodal data and those with incomplete data revealed statistical differences in a limited subset of features, most notably ECOG PS and smoking history. Importantly, while ECOG PS is a known prognostic factor, it is inherently subjective and prone to inter-observer variability, which may account for the observed statistical discrepancy. These tables are added to the **Supplementary Information 1, Tables 16-27.***

2. Model Robustness across Populations (Generalizability)
To determine if the multimodal population represents a "biased", easier-to-predict subset, we tested the performance of the CB-only (previously referred to as RWD) MLEF model on both the multimodal-complete and the modality-incomplete populations.

- ***Results:*** *Across the main cohorts (C2, C23) and outcomes (OS6, OS24, DCR), there were no statistically significant differences in AUC performance between the two groups (permutation test p > 0.05). These results are added in* ***Supplementary Information 2, Table 3.***
- ***Interpretation:*** *In several instances, the CB-only MLEF model performed nominally better but not statistically significantly better in the incomplete-data population. This empirically shows that the multimodal cohort does not suffer from a significant positive selection bias that can influence the better predictive performance.*

3. *Incremental Value (Sensitivity Analysis)*

To confirm the true incremental value of additional modalities, we performed an ablation analysis strictly within the multimodal-complete subset. This shows that the performance gain is due to data integration and not a change in the patient population.

- ***Findings:*** *The transition from CB to CB+DP and CB+RAD showed consistent, statistically significant improvements in AUC (e.g., in C2 OS6: CB 0.689, CB+FMRAD 0.843, p < 0.01). These results are added to* ***the Supplementary Information 2, Table 2.***

We also performed a similar analysis for the DLIF model (reported in ***Supplementary Information 4, Section 2, Fig. 6-19****), and results showed a similar trend, highlighting the importance of the lack of data modalities for these architectures.*

We referred to these analyses in the main manuscript, Discussion (lines 399-403, 449-450) and Online Methods (680-710)

6. The amount of complete multimodal data is still limited: Although the total cohort is large, the number of patients with full multimodal coverage is relatively small. This limits the reliability of the multimodal analyses, particularly for deep learning models that require substantial data to learn stable cross-modal interactions. The reported multimodal gains may therefore reflect subset effects rather than true, generalizable multimodal synergy.

Response to Reviewer: We thank the reviewer for this important comment and fully agree with this limitation. We have now explicitly included this point as a key limitation in the revised manuscript and discussed it in detail (see Discussion, sections 399-403 and 449-452). In particular, we acknowledge that the relatively small number of patients with complete multimodal data may limit the robustness of the multimodal analyses, especially for DL approaches to limit the reduction of the dataset, and that the observed gains could reflect subset-specific effects rather than fully generalizable multimodal synergy. This may also explain why DLIF does not show additional benefit from multimodal integration

7. External validation is limited: The external validation cohort is valuable, but it comes from a single center and differs substantially from the development cohorts, including a notably longer median overall survival. The observed performance decline in EXVAL weakens claims of broad generalizability.

Response to Reviewer: We thank the reviewer for this important comment and agree with the concern. In the revised manuscript V1, we performed additional analyses to better understand the performance decrease in the EXVAL cohort. Specifically, we conducted a systematic comparison of key clinical variables across TRAIN, TEST, and EXVAL cohorts, which showed significant imbalances in several features (including

sex, ECOG PS, LDH, smoking status, PD-L1 expression, and overall survival distributions; all $p < 0.05$). These findings indicated that the EXVAL cohort represents a clinically distinct population (**Supplementary Information 1 Table 5-8**). Accordingly, we have clarified in the revised manuscript that the observed performance drop is likely driven by differences in underlying patient characteristics rather than model instability. This point has been further elaborated in the Discussion (lines 391-395). Furthermore, in the previous revision V1, we addressed concerns regarding generalizability by moderating our claims and removing references to broad generalizability, a prospective silent trial is ongoing.

8. The comparison of model architectures is limited: The study contrasts only two integration strategies (MLEF and DLIF), without benchmarking against a broader range of contemporary multimodal architectures (e.g., transformer-based, late-fusion, or hybrid approaches). In addition, differences in data inclusion (e.g., complete-case vs. missing-modality settings) make the comparison less controlled. As a result, the conclusion that simpler machine learning approaches perform comparably to deep learning may be premature and not fully supported by a comprehensive architectural evaluation.

Response to Reviewer: We thank the reviewer for this important comment. In previous revision we have clarified this point as follow:

Specifically, to address the limitations related to missing modalities and complete-case bias, we incorporated and evaluated two additional fusion models explicitly designed to handle missing data: ***DyAM (Vanguri et al., 2022)*** *and* ***MCVAE (Zanitti et al., 2025)****. Both models were systematically assessed using the same cohorts (C23, C2), clinical endpoints (ORR, DCR, CBR, OS_6, OS_24), and evaluation strategy (LOCO cross-validation, held-out test set, and external validation) as MLEF.*

These additional analyses enabled us to provide a more comprehensive and controlled comparison across different modeling paradigms, including approaches tailored to real-world settings with incomplete data. We have clarified this in the revised manuscript and tempered our conclusions, avoiding overinterpretation regarding the comparative performance of machine learning versus deep learning approaches.

DyAM (Vanguri et al. 2022). *DyAM implements an intermediate-fusion framework that integrates radiology, pathology, and genomics through modality-specific hierarchical encoders followed by an attention-based joint representation. Missing modalities are handled via binary availability masks that gate each encoder's contribution at inference time, without requiring imputation.*

MCVAE (Zanitti et al. 2025). *MCVAE is a contrastive variational autoencoder designed for incomplete multimodal data. It incorporates modality-specific variational encoders, availability-aware gating, stochastic modality masking during training, and contrastive alignment to promote modality-agnostic representations. Full architectural details are provided in the Supplementary Methods.*

Results: DyAM vs. DLIF. *Across all outcomes and both cohorts (C23, C2), the two architectures showed similar CV AUC (~0.61–0.71). Performance at the test set was broadly consistent with CV for both models. Differences emerged at external validation and were outcome-dependent: for response and benefit endpoints (ORR, CBR), DyAM achieved higher EXVAL AUC; for survival endpoints (OS6, OS24), DLIF showed*

a consistent numerical advantage, though confidence intervals formally overlapped in most cases. In both models and across all outcomes, adding modalities beyond clinical variables did not yield consistent improvements in EXVAL; the clinical-only configuration was the most robust across all settings.

Results: MCVAE vs DLIF. *Across all outcomes and both cohorts (C23, C2), MCVAE showed cross-validation AUCs broadly comparable to DLIF, consistent with equivalent in-distribution discriminative capacity. Performance at the held-out test set was similarly stable. On EXVAL, MCVAE showed outcome-dependent variability: for ORR, results were inconsistent across cohorts (C23: 0.38±0.07; C2: 0.54±0.08), with the C23 value falling below chance; for CBR and DCR, performance was comparable to DLIF with overlapping CIs; for survival endpoints, MCVAE performed below DLIF numerically (OS_6 C23: 0.65±0.08, C2 0.67±0.09) while OS_24 showed diverging cohort behavior (C23: 0.66±0.08; C2: 0.52±0.10). As with DyAM and DLIF, adding modalities beyond clinical variables did not yield consistent external validation improvements, and the clinical-only configuration remained the most robust setting throughout.*

Finally, further transformer-based multimodal foundation models will be considered for the future as the next technological advancement on the I3LUNG EU project, which continues for another 2 years, and this was explicitly highlighted in the discussion point.

9. Pathology encoder evaluation is lacking: The digital pathology pipeline relies on one fixed encoder only, without comparison to other contemporary pathology backbones or slide-level aggregation methods. This substantially limits the technical rigor of the multimodal analysis, because the contribution of the pathology modality cannot be separated from the specific and unvalidated encoder choice.

Response to Reviewer: We thank the reviewer for this important comment and agree that the evaluation of the pathology encoder is a relevant aspect of the multimodal framework. In the revised analysis, we have included additional experiments using an alternative and more recent pathology backbone (TITAN, published after our first submission), to better assess the contribution of this modality.

While the TITAN-based models showed a slightly improved performance at the unimodal level compared to GigaPath, this did not translate into a meaningful improvement in the overall multimodal model performance. This finding suggests that the limited added value observed for multimodal integration is not solely driven by the choice of pathology encoder, but may instead reflect constraints related to sample size and the availability of complete multimodal data.

We have clarified this point in the revised manuscript and highlighted that larger datasets will likely be required for deep learning-based multimodal approaches (such as DLIF, DyAM and MCVAE) to fully capture cross-modal interactions and demonstrate robust added value.

TITAN: Ding, T., Wagner, S.J., Song, A.H. et al. A multimodal whole-slide foundation model for pathology. Nat Med 31, 3749–3761 (2025). https://doi.org/10.1038/s41591-025-03982-3

In Figure 2b (main manuscript) you can see the results on the C23 full set on unimodal MLEF. We have also incorporated the results in the **Supplementary Information 4 Section S3.**

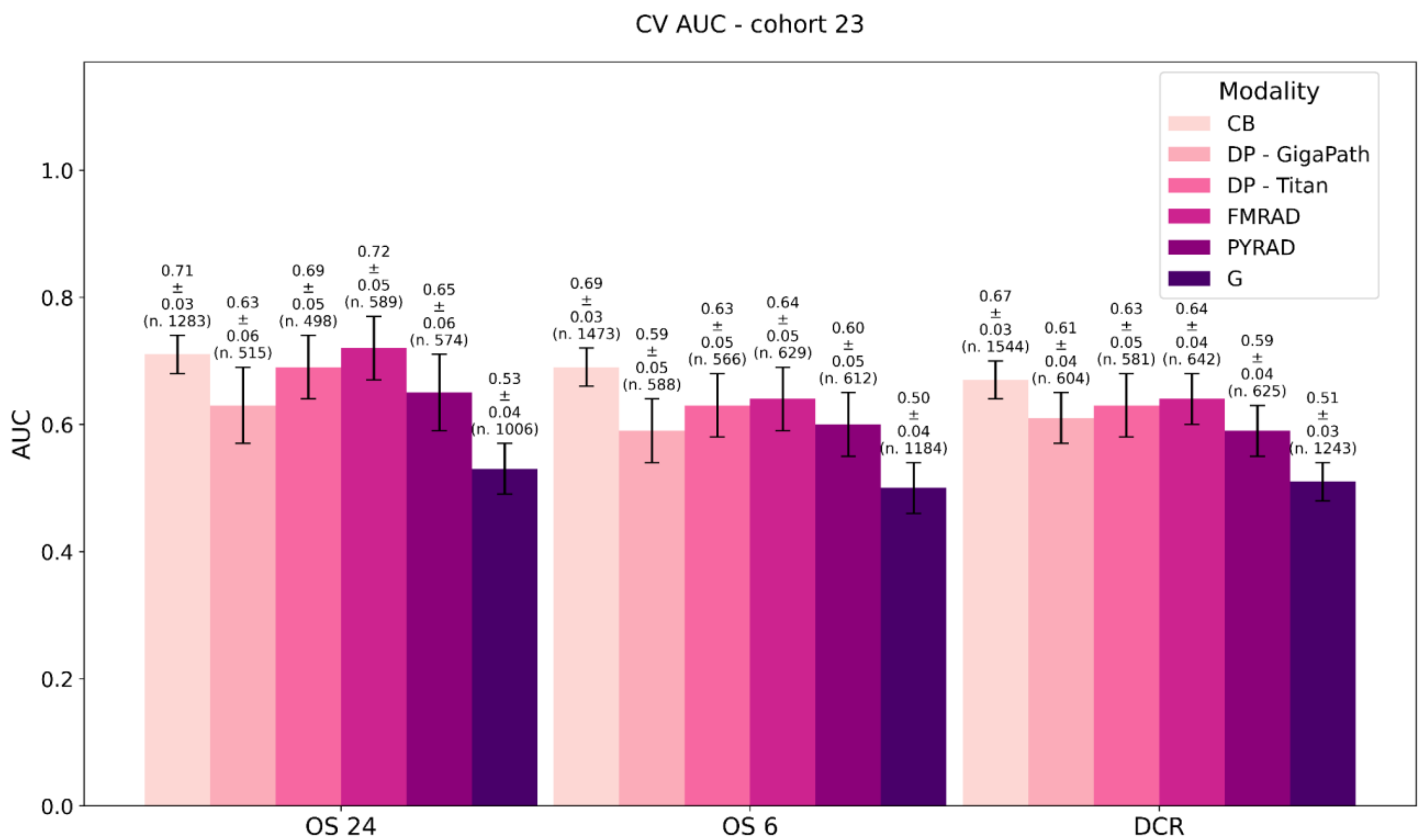

Figure 2b Unimodal MLEF performance across outcomes.

Multimodal Comparison with TITAN Digital Pathology Features

To determine whether results were driven by the choice in DP feature extractor, we re-extracted 768-dimensional slide embedding features using TITAN (Ding et al., Nature Medicine 2025), a multimodal whole-slide foundation model pretrained using self-supervised learning on digital pathology whole slide images and vision-language alignment with pathology reports. We compared our results using Prov-GigaPath digital pathology features to those extracted by TITAN across all three response outcomes and modality combinations.

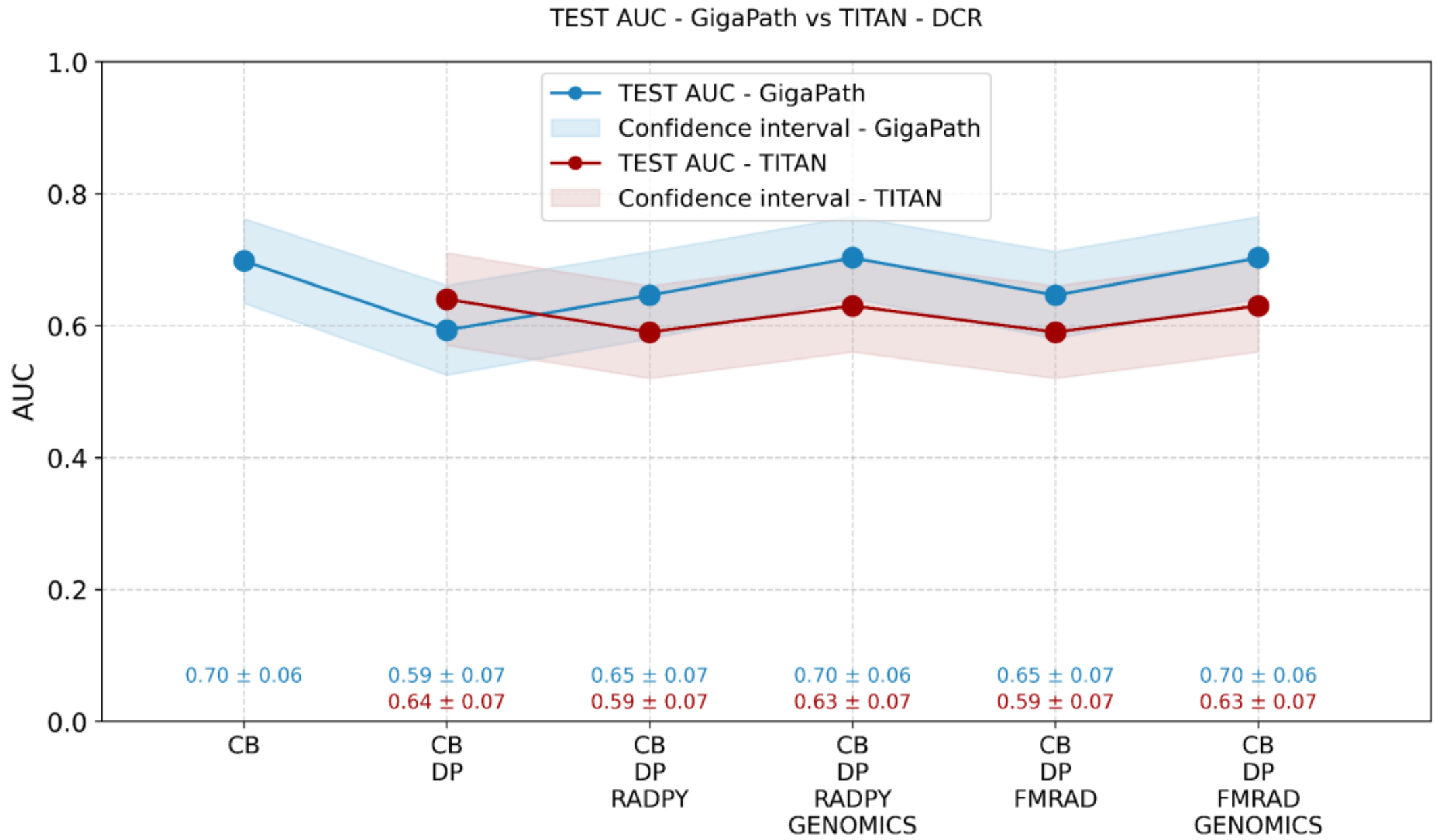

Figure 22 (Supp Info 4). DLIF results in our original Prov-GigaPath-based model vs. the TITAN-based model in Test for outcome DCR.

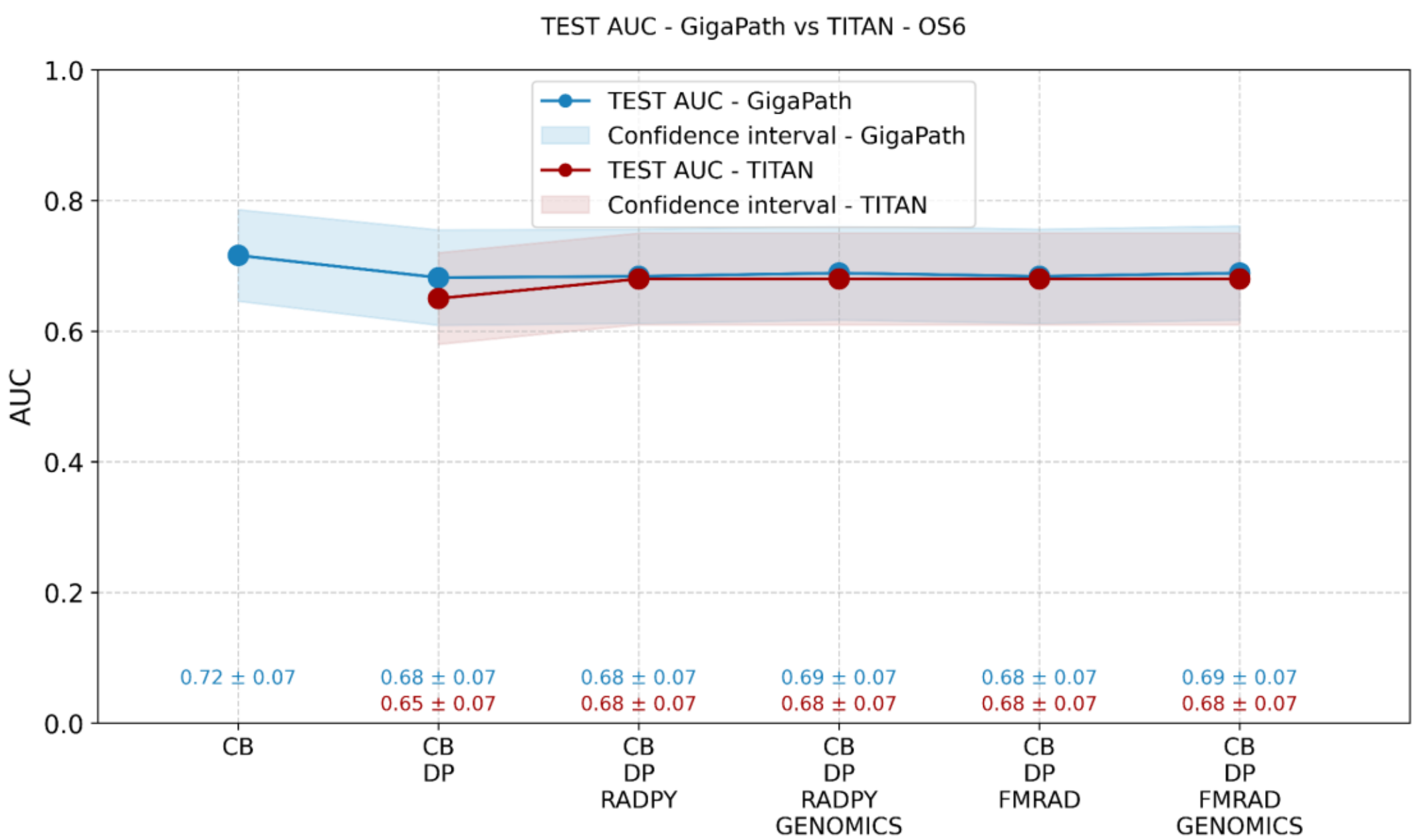

Figure 23 (Supp Info 4). DLIF results in our original Prov-GigaPath-based model vs. the TITAN-based model in Test for outcome OS6.

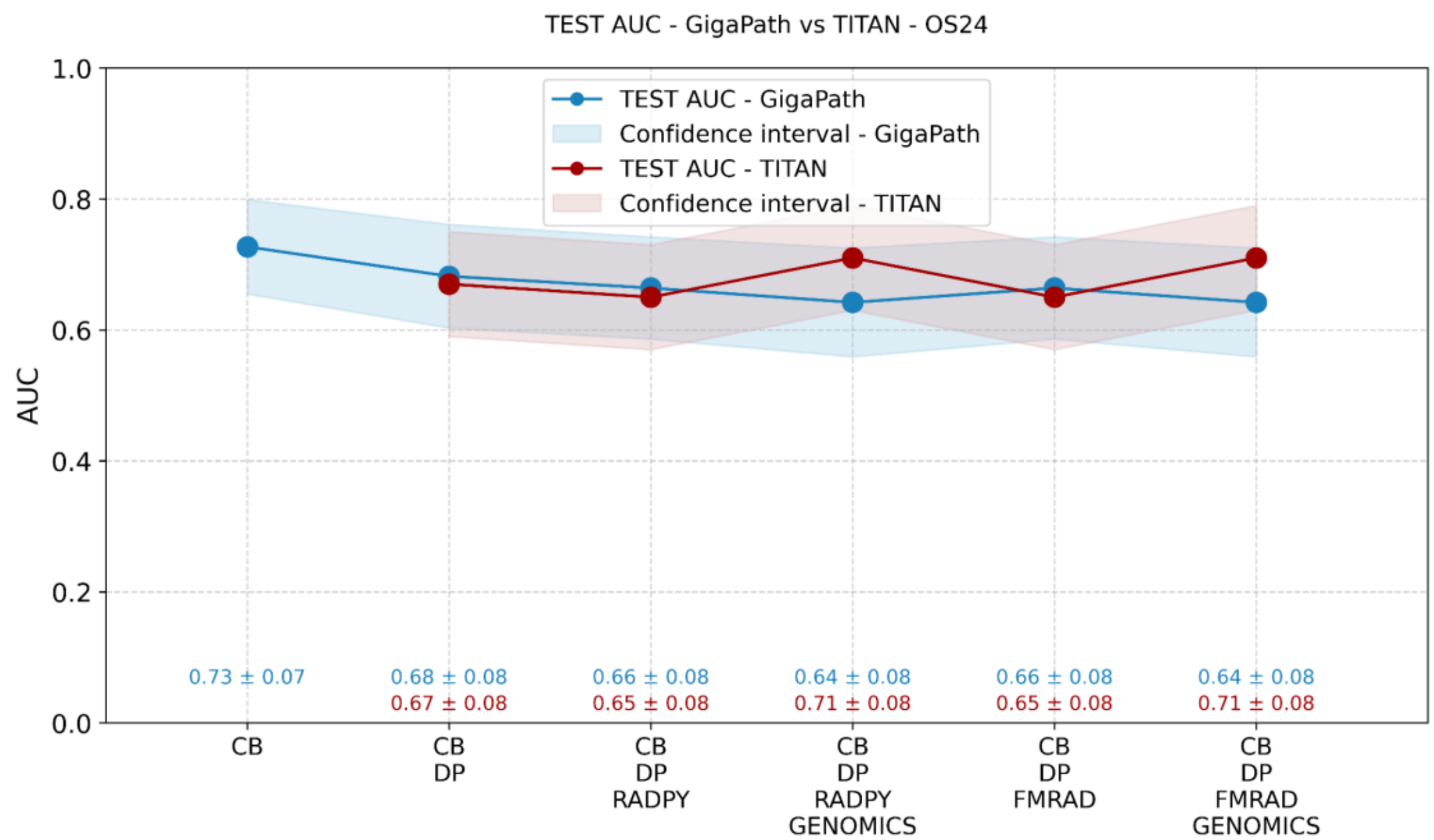

Figure 24 (Supp Info 4). DLIF results in our original Prov-GigaPath-based model vs. TITAN-based model in Test for outcome OS24.

Finally, we compare our original model results with digital pathology feature extractor Prov-GigaPath (Xu et al., Nature 2024), and a newer model, TITAN (Ding et al., Nature Medicine 2025), also for MLEF. Results can be found in **Supplementary Information 2, Figure 3-5.**

10. Statistical testing is insufficiently transparent: The manuscript includes many subgroup analyses and pairwise comparisons, but the handling of multiplicity is not adequately described. In addition, the description of DeLong testing versus two-sided t-tests is unclear and should be clarified precisely.

Response to Reviewer: We thank the reviewer for this important comment and agree that greater clarity in the description of the statistical methods is needed. We clarified in Online Methods lines 696-710. Specifically, we have corrected the corrected description of the DeLong test based on a normal approximation and produced a z-test.

In addition, we have clarified the approach used for comparing C-index values in survival analyses. We now explicitly state that differences in C-index were assessed using bootstrapping (n=1000) with two-sided p-values, and we emphasize that the DeLong test was not used for survival outcomes, as it is only appropriate for binary endpoints.

Regarding multiplicity, we agree with the reviewer that formal correction methods may not be appropriate given the exploratory nature of this study. Therefore, we have added a statement in the Online Methods clarifying that all subgroup analyses and pairwise comparisons are hypothesis-generating, that no adjustment for multiple comparisons was applied, and that all reported p-values

should be considered nominal and interpreted in the context of multiple testing. We also specify that results from the independent TEST and EXVAL cohorts represent the primary evidence supporting model robustness.

Minor:

1. The genomics representation is relatively coarse, particularly the use of a composite actionable-driver variable, which may obscure biologically meaningful heterogeneity.

Response to reviewer:We thank the reviewer for this comment and agree with this important point. We have expanded the Discussion and the Online Methods to clarify the rationale and limitations of the composite actionable-driver variable, including its potential to mask biological heterogeneity in the revised manuscript V1. See below for details and explanations.

Revision V1, reviewer 4 comment 3, discussion, and online methods:

The rationale for the composite driver encoding stems from the marked imbalance and substantial missing data observed in our cohort for individual oncogenic drivers. Conversely, the composite driver variable addresses this by pooling rare events into a more robust binary predictor while maintaining biological interpretability. More information on the mutation distribution in c23 can be found in ***Table 15 in Supplementary Information 1.***

To address the validity of our composite driver feature, we conducted a comprehensive sensitivity analysis comparing two approaches: (Model 1) the original composite driver (EGFR ∨ ALK ∨ ROS1 ∨ RET), and (Model 2) a stratified model separating EGFR as an independent feature while retaining ALK/ROS1/RET as a composite variable. We performed DeLong's test to compare AUC values across all clinical endpoints, with 95% confidence intervals. We report the CV results in ***Table 2 in Supplementary Information 7****.*

Some outcomes show statistically significant differences, but the absolute performance is uniformly low (AUCs close to 0.5). This indicates limited discriminative ability regardless of whether EGFR is separated or collapsed into the composite driver. We repeated the sensitivity analysis for CB data + composite driver (EGFR, ALK, ROS1, RET) and CB data + driver with a separated EGFR feature.

Finally, SHAP analysis showed that EGFR mutations and ALK/ROS1/RET translocation features were associated with negative contributions to IO prediction, suggesting lower predicted immunotherapy benefit and that their combination within the same feature is biologically justified.

Conclusion: *The decision to propose a combined score is driven by the significant missingness in the data available to us. Experiments separating the EGFR driver from the other oncogenic drivers provide only minimal improvement in the model's performance.*

2. Radiomic analysis is restricted to the primary lesion and may miss clinically relevant information from metastatic sites and inter-lesional heterogeneity.

Response to Reviewer: We thank the reviewer for this important comment and agree with this limitation. We have now explicitly highlighted this limitation in the revised manuscript, acknowledging that restricting radiomic analysis to the primary lesion may miss relevant information from metastatic sites and inter-lesional heterogeneity, which could influence model performance (lines 453-454 in discussion).

We have also clarified that future steps within the I3LUNG project will include the development and evaluation of comprehensive multi-lesion segmentation approaches, to better capture whole-disease heterogeneity and potentially improve predictive performance (CT scans sections Online Methods, lines 621-623).

3. There are internal inconsistencies and editorial issues, including mismatch in reported total sample size across sections and visible formatting/reference-management artifacts.

Response to Reviewer: We thank the reviewer for this comment. We have carefully reviewed the manuscript to address editorial inconsistencies, including small discrepancies in reported numbers (e.g., Table 1), and to correct formatting and reference-related issues. These revisions have been implemented to improve clarity and consistency throughout the manuscript.

In summary, this is a potentially impactful multicenter study addressing an important clinical problem. However, the present version makes claims that are stronger than the evidence currently supports. The clearest conclusion is that carefully curated clinical real-world data outperform common single biomarkers, while the incremental value of multimodal integration remains promising but not yet convincingly established.

Response to Reviewer: We thank the reviewer for this thoughtful and balanced summary. We have revised the title, abstract, discussion, and conclusions to better align our claims with the strength of the evidence. In particular, we now emphasize that the most robust finding is the strong performance of curated clinical real-world data compared to single biomarkers. We emphasize the role of the clinical usability study in demonstrating the benefit of clinicians by using the XAI CB-only tool while the added value of multimodal integration is presented as promising but still preliminary and requiring further validation.
